# Performance of tropical forest seedlings under shade and drought: an interspecific trade-off in demographic responses

**DOI:** 10.1038/s41598-019-55256-x

**Published:** 2019-12-11

**Authors:** Stefan J. Kupers, Christian Wirth, Bettina M. J. Engelbrecht, Andrés Hernández, Richard Condit, S. Joseph Wright, Nadja Rüger

**Affiliations:** 1 German Centre for Integrative Biodiversity Research (iDiv) Halle-Jena-Leipzig, Deutscher Platz 5e, 04103 Leipzig, Germany; 2 Systematic Botany and Functional Biodiversity, Institute of Biology, Leipzig University, Johannisallee 21-23, 04103 Leipzig, Germany; 3 Max-Planck-Institute for Biogeochemistry, Hans-Knöll Str. 10, 07745 Jena, Germany; 4 Department of Plant Ecology, Bayreuth Center of Ecology and Environmental Research (BayCEER), University of Bayreuth, 95447 Bayreuth, Germany; 5 Smithsonian Tropical Research Institute, Apartado 0843-03092, Balboa, Ancón Panama; 6 Field Museum of Natural History, 1400 S Lake Shore Dr., Chicago, IL 60605 USA; 7 Morton Arboretum, Lisle, IL 60532-1293 USA

**Keywords:** Ecology, Forest ecology, Tropical ecology

## Abstract

Seedlings in moist tropical forests must cope with deep shade and seasonal drought. However, the interspecific relationship between seedling performance in shade and drought remains unsettled. We quantified spatiotemporal variation in shade and drought in the seasonal moist tropical forest on Barro Colorado Island (BCI), Panama, and estimated responses of naturally regenerating seedlings as the slope of the relationship between performance and shade or drought intensity. Our performance metrics were relative height growth and first-year survival. We investigated the relationship between shade and drought responses for up to 63 species. There was an interspecific trade-off in species responses to shade versus species responses to dry season intensity; species that performed worse in the shade did not suffer during severe dry seasons and vice versa. This trade-off emerged in part from the absence of species that performed particularly well or poorly in both drought and shade. If drought stress in tropical forests increases with climate change and as solar radiation is higher during droughts, the trade-off may reinforce a shift towards species that resist drought but perform poorly in the shade by releasing them from deep shade.

## Introduction

Differential performance of plant species along resource gradients affects species composition and contributes to species diversity^1–3^. Light and water are key resources for plants and a lack of light and water (i.e. shade and drought) strongly limits plant performance^4–6^. Yet, it remains unclear how shade and drought interact to shape performance of natural plant communities^7,8^.

Smith and Huston^9^ were the first to propose an interspecific trade-off between shade and drought tolerance, i.e. a shade tolerant species should be intolerant to drought and vice versa. They proposed various trade-offs in plant adaptations to cope with shade and drought, such as a trade-off in allocation to aboveground structures to increase light capture versus allocation to belowground structures to increase water uptake. Shade and drought tolerance traded off in a landmark study that determined shade and drought tolerance scores of species across the northern hemisphere^10^. However, other studies suggest that shade and drought tolerance may be unrelated^11,12^ because traits that determine these tolerances do not require high resource allocation. For example, tolerance to shade is not directly related to high aboveground allocation but is instead promoted by slow growth and low specific leaf area^13^. Other traits reduce demand for light and water simultaneously (e.g. low respiration rates or low leaf nitrogen concentration), allowing for high shade and drought tolerance^14,15^. These traits contrast with traits promoting fast resource acquisition (e.g. low tissue density or high photosynthetic capacity), leading to the well-established fast–slow plant economic spectrum that predicts that traits related to shade and drought tolerance are positively related^16^.

Various studies have evaluated the relationship between shade and drought tolerance in different ecosystems, but there is no conclusive answer as to which relationship emerges under which environmental conditions (see Table 1). Most of these studies used functional traits or species distributions as proxies for shade and drought tolerance (Table 1), even though whole-plant performance finally determines population dynamics^17^. The focus on traits is partly due to the lack of small-scale data on light and water availability, which hinders evaluation of performance differences within plant communities. This is particularly true for soil water potential, which is the relevant measure of water status for plant performance^18^ because plants draw water from the soil along the soil–plant–atmosphere continuum of water potential^19^. As a result, attempts to evaluate relationships between performance in shade and drought have been limited to experiments (e.g.^11,14^). These experiments can only include a few species, making it difficult to generalize performance trade-offs to species-rich natural communities.

**Table 1:** Studies that tested the interspecific relationship between tolerances to shade and drought.

Study	Vegetation type	Life stage	Nr. spp.	Approach	Shade tolerance definition	Drought tolerance definition	Support for hypothesis*
Suding *et al*.^56^	Lake-plain prairie	Seedlings	11	Experimental performance	Growth in low versus high light	Growth in low versus high soil moisture	Trade-off^†^
Niinemets and Valladares^10^	Temperate forest	Seedlings and saplings	806	Species distributions/traits	Subjective species occurrence indices compiled across sources	Subjective species occurrence indices compiled across sources	Trade-off
Stahl *et al*.^93^	Temperate forest	Seedlings and saplings	305	Species distributions/traits	Subjective species occurrence indices compiled across sources	Subjective species occurrence indices compiled across sources	Trade-off^†^
Poorter and Markesteijn^24^	Tropical dry and moist forest	Seedlings	38	Species distributions	Juvenile crown exposure	Relative abundance of species in dry versus moist forest site	Trade-off
Brenes‐Arguedas *et al*.^55^	Tropical moist forest	Seedlings	24	Experimental performance	Leaf area growth in the understory	Survival in control versus irrigated conditions	Trade-off^‡^
Martínez‐Tillería *et al*.^94^	Arid scrubland	Seedlings	6	Experimental performance	Growth and survival in low, medium and high light	Growth and survival in control versus irrigated conditions	Independence^†^
Sack and Grubb^14^	Temperate forest	Seedlings	4	Experimental performance	Growth in high versus low light treatment	Growth in high versus low watering treatment	Independence^†^
Sack^11^	Temperate forest	Seedlings	13	Experimental performance	Growth and survival in high versus low light treatment	Growth and survival in high versus low watering treatment	Independence
Sánchez‐Gómez *et al*.^12^	Mediterranean forest	Seedlings	8	Experimental performance	Growth in high versus low light treatment	Growth in high versus low watering treatment	Independence
Markesteijn and Poorter^13^	Tropical dry and moist forest	Seedlings	62	Species distributions	Juvenile crown exposure	Relative abundance of species in dry versus moist forests	Independence
Engelbrecht *et al*.^95^	Tropical moist forest	Seedlings and adult trees	28	Species distributions/Experimental performance	Percentage of recruits in high light conditions	Species distributions along rainfall and soil moisture gradients, survival in dry versus irrigated conditions	Independence
Sterck *et al*.^25^	Tropical dry forest	Saplings	13	Model parametrized with functional traits	Simulated light compensation point	Simulated water compensation point	Acquisitive vs. conservative^‡^
Sterck *et al*.^96^	Tropical dry forest	Saplings	37	Model parametrized with functional traits	Simulated light compensation point	Simulated water compensation point	Acquisitive vs. conservative^‡^
Markesteijn *et al*.^26^	Tropical dry forest	Seedlings	40	Species distributions, functional traits	Juvenile crown exposure	Midday leaf water potential	Acquisitive vs. conservative
Ouédraogo *et al*.^97^	Tropical moist forest	Trees ≥10 cm dbh	229	Field performance/species guilds	Maximum growth rate and regeneration guild	Growth responses to climatological drought and modelled soil water content	Acquisitive vs. conservative^†^

*Support for the ‘trade-off’ or ‘acquisitive versus’ conservative hypothesis was found when a correlation between tolerances was significantly negative or positive, respectively (*p* < 0.05), and support for the ‘independence’ hypothesis was found when the correlation was not significant.

^†^Relationship between tolerances was evaluated other than through a correlation between tolerances, e.g. through relating shade and drought tolerance to a principle coordinate analysis of functional traits, comparison of performance of individual species/guilds among treatments, or among natural conditions varying in shade or drought intensity.

^‡^Hypothesis was partly supported, correlation between shade and drought tolerance was marginally significant (0.05 ≤ *p* < 0.10).

The combined pressure of shade and drought is particularly evident in tropical forests^8^. As in other ecosystems, the relationship between light and water availability gradients in these forests determines the adaptive pressures acting on plant communities. This relationship varies depending on the scale at which the gradients are compared and local climatic conditions. In wet and seasonal moist tropical forests, open vegetation or large gaps have lower soil moisture than denser patches due to higher evaporation rates^20,21^. Similarly, less densely vegetated hilltops tend to be drier than shaded valleys^22,23^. Thus, in these forests species would either need to cope with low light or low soil moisture availability. Since these environmental differences are relatively modest, one would expect a relatively weak interspecific trade-off between performance in shaded versus dry conditions. When comparing relatively closed moist forests with relatively open dry forests on a regional scale, there is a stronger contrast in environmental conditions which should result in a stronger trade-off, i.e. in moist forests species are well adapted to shade but poorly to drought, while in dry forests species are well adapted to drought but poorly to shade^24^. Within tropical dry forests there is a pronounced division between evergreen species that specialize on the dark understory and deciduous species that specialize on the bright canopy or gaps^25^. Here there is a positive relationship between adaptation to shade and drought, i.e. a division between conservative evergreen species that specialize on coping with shade and drought and acquisitive deciduous species that avoid shade and take advantage of optimal growing conditions in the wet season^25,26^.

Temporal variation in shade and drought intensity also affects plant performance in tropical forests. Light variation caused by gap dynamics are crucial for the establishment and growth of many species^27^. Pronounced dry seasons and occasional, severe droughts strongly limit growth and increase mortality^28,29^. Light availability in tropical forests is higher during droughts due to increased solar radiation^30,31^, which may also interactively affect performance.

Our objective was to study the relationship between demographic responses (growth and survival) of naturally regenerating seedlings to spatiotemporal variation in shade and drought in a moist tropical forest. Seedlings are particularly vulnerable to shade and drought because their low biomass limits resource capture above and belowground^28,32^. We evaluated species responses to shade and drought as the slope between seedling performance (growth or survival) and shade or drought intensity for a large community of woody seedlings on Barro Colorado Island (BCI), Panama. To this end, we determined shade intensity at 200 seedling census sites across years (i.e. spatiotemporal variation in shade). We determined spatial variation in drought by measuring a detailed spatial gradient of soil water potential at the seedling sites (i.e. spatial drought) and temporal variation in drought by determining dry season severity (inter-annual drought). We then correlated shade responses to drought responses for growth, survival and, finally, growth versus survival.

We hypothesise that there is an interspecific trade-off (i.e. a negative correlation) between performance in shade versus drought, because we expect a trade-off in plant adaptations to cope with shade and drought^9,10^. Additionally, we expect higher light availability in drier habitats and during droughts (and vice versa)^20–23,30,31^, allowing species to be adapted to either shade or drought because they would temporarily be released from the other stress. In order to understand how performance of species in shade and drought is linked to broader demographic strategies, we related shade and drought responses to an independently assessed fast–slow continuum based on demographic rates (recruitment, growth and survival) ranging from conservative to acquisitive species^16^. On BCI, conservative species with slower growth and lower mortality have traits that confer shade tolerance, such as high wood density^33^. Thus, we hypothesize that more conservative species perform better in the shade than acquisitive species. On the other hand, acquisitive species should cope better with drought, based on the expected trade-off between shade and drought responses (see above).

## Results

### Responses to shade and drought

Ninety-one species fulfilled the sample size requirements for growth and/or survival analyses. For growth, we estimated shade responses for 63 species and spatial and inter-annual drought responses for 84 species (62,973 observations in total). For survival, we estimated shade and drought responses for 27 and 45 species, respectively (31,560 observations in total). Fewer species fulfilled the larger sample size requirements for estimating survival responses (≥100 observations) compared to growth responses (≥50 observations). Similarly, fewer species fulfilled sample size requirements for analyses of light responses because the canopy measurements used to estimate light availability took place in 12 of 20 years (see *Methods: Estimating shade and drought responses* for details).

**Figure 1:**
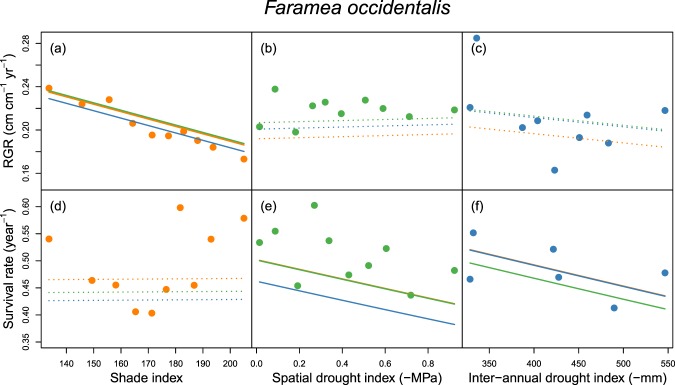
Relationship between observed and fitted relative growth rate (RGR, upper panels) and survival rate (lower panels) and shade (**a,d**), spatial drought (**b,e**) and inter-annual drought (**c,f**) of the abundant treelet *Faramea occidentalis*. Growth decreased significantly in deeper shade (**a**) and survival decreased significantly in drier sites (**e**, spatial drought) and years (**f**, inter-annual drought). Large dots represent mean observed growth or survival for ten shade or drought classes, each containing 10% of the individuals of the species (only six classes in (**f**), due to high abundance in one year). Lines show fitted growth and survival with increasing shade (**a,d**, orange), spatial drought (**b,e**, green) and inter-annual drought (**c,f**, blue), at mean values of the other independent variables. Solid and dotted lines indicate significant and non-significant responses, respectively. Lines whose colour differs from the large dots within each panel represent 1 SD increase in shade (orange), spatial drought (green) or inter-annual drought (blue). Figure S1 presents responses to shade and drought for all analysed species.

There was at least one significant growth or survival response to shade or drought for 31% of the species included in the analyses (28 of 91 of species, Supplementary Tables S1 and S2, Fig. S1). Figure 1 illustrates shade and drought responses of growth and survival for *Faramea occidentalis*, the most common species in our study, which grew significantly slower in the shade and had lower survival during drought. Most, but not all, of the significant responses to shade or drought were negative, i.e. weaker performance, with increasing shade or drought (Supplementary Table S1.1). As reported earlier from these seedling data^34^, relative growth rates decreased and survival increased significantly with height for the large majority of species (86% and 76% of species, respectively, see Supplementary Table S1.1). Explained variance (R^2^) was 0.24 in the growth model and 0.12 in the survival model.

### Relationship between shade and drought responses

**Figure 2:**
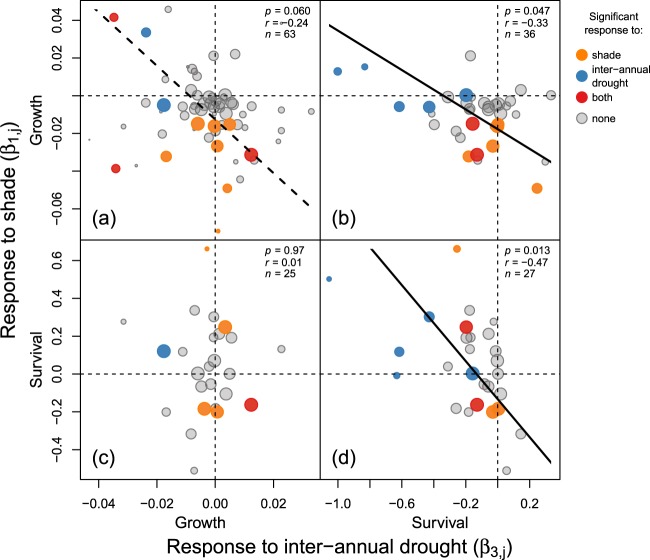
Relationships between species responses to shade and inter-annual drought (i.e. dry season severity) for growth (**a**), survival (**d**), or growth versus survival (**b,c**). Solid and dashed lines indicate significant (*p* < 0.05) and marginally significant (0.05 ≤ *p* < 0.10) relationships, respectively. Negative relationships indicate a trade-off between shade and drought responses. Correlations are weighted by the uncertainty in species tolerances (smaller dots have higher uncertainty and lower weight, see equation (5) in text). Colours identify species with insignificant (grey) or significant responses to shade (orange), inter-annual drought (blue) or both (red).

There was a trade-off (i.e. a significant negative correlation) between growth responses to shade and survival responses to inter-annual drought (β_1,gr_ ~ β_3,su_, Fig. 2b) and between survival responses to shade and inter-annual drought (β_1,su_ ~ β_3,su_, Fig. 2d). There was also a marginally significant negative correlation between growth responses to shade and inter-annual drought (β_1,gr_ ~ β_3,gr_, Fig. 2a). Survival responses to shade and growth responses to inter-annual drought were unrelated (β_1,su_ ~ β_3,gr_, Fig. 2c). Results were robust when we included individuals that resprouted, were visually damaged or infected by pathogens (Supplementary Fig. S1.1). We found no significant relationships between responses to shade and spatial drought (β_1,su_ ~ β_2,gr_, Supplementary Fig. S1.2).

### Responses in relation to the fast–slow continuum

**Figure 3:**
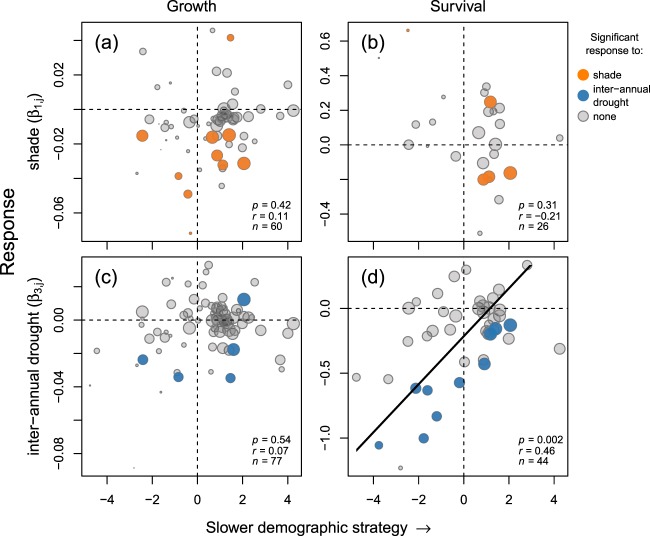
Relationships between the fast–slow continuum and responses to shade (**a,b**) and dry season severity (i.e. inter-annual drought) (**c,d**) for growth (left) and survival (right). The position of species along the continuum was quantified by a weighted PCA of demographic rates (growth, survival, number of sapling recruits) of trees ≥1 cm dbh recorded in the BCI 50-ha plot^33^. Low and high scores correspond to species with fast and slow demographic strategies, respectively. Colours identify species with insignificant (grey) or significant responses to shade (orange) or inter-annual drought (blue). Relationships were consistent when the fast–slow continuum was calculated using seedling performance and/or seed number additionally (see Supplementary Table S1.2).

Survival responses to inter-annual drought (β_3_) increased with species’ scores on the fast–slow continuum, with scores on the fast–slow continuum from an independent analysis of the performance of trees ≥1 cm dbh in the 50-ha plot^33^. Species at the fast end of the fast–slow continuum (low PCA score, fast-growing species with low survival rates) suffered large reductions in survival in years with severe dry seasons, while species at the slow end of the continuum had little reduction in survival (Fig. 3d). In contrast, other responses (growth to inter-annual drought, and growth or survival to shade or spatial drought) were unrelated to the fast–slow continuum (Fig. 3a–c, Supplementary Fig. S1.3). These results were robust when the fast–slow continuum was calculated with seedling growth and survival in addition to performance of trees ≥1 cm dbh and with additionally including seed number and seedling recruitment (Supplementary Table S1.2).

## Discussion

We assessed the interspecific relationship of species responses to shade and drought in a naturally regenerating tropical seedling community. We found relatively few significant responses of species to shade or drought (Supplementary Table S1.1), in part because many species had modest sample sizes and were limited to part of the observed shade and drought gradients. Yet, there was an interspecific trade-off in responses to shade and dry season severity (Fig. 2), indicating that the ability to cope with (or perform better under) shade or drought comes at the expense of coping with the other stress. Weaker performance during severe dry seasons was also related to a faster demographic strategy (Fig. 3d). Future increases in drought length or severity may come with decreases in shade intensity, which would reinforce a shift towards more drought tolerant and less shade tolerant species.

### Responses to shade and drought

The proportion of species responding significantly to shade or drought was relatively low (Supplementary Table S1.1), which contrasts with reported seedling sensitivity to shade and drought (e.g.^35–37^). This is likely caused by high uncertainty in responses for the many species with low sample sizes, decreasing the chance of finding significant responses for these rare species (Supplementary Fig. S1.4). In addition, many rare species that we could not include are habitat specialists that likely respond more strongly to shade or drought. Second, dispersal or recruitment limitation curtail seedling distributions along light and moisture gradients to sites near successful adults. Seeds of moisture sensitive species rarely disperse to dry microsites^38,39^, and if they do and they germinate, many seedlings die during dry spells before inclusion in the annual census^40^. Likewise, seeds of light-demanding species fail to germinate in low light^41^. There were particularly few significant responses to spatial drought. This is likely due to the relatively shallow gradient in spatial drought in the 50-ha plot^34^, particularly when compared to other tropical forests^42^. Finally, there may be fewer significant responses to shade than expected because our shade index could not capture ephemeral sunflecks, which are important sources of light for understory plants^43,44^.

Unexpectedly, some species performed better in shade or drought (Supplementary Table S1.1). Species may have directly suffered from excessive light (photoinhibition) or water (waterlogging)^45,46^. Alternatively, shade may reduce drought stress, especially during severe dry seasons^47–49^. There was a negative correlation across the 200 seedling census sites between our indices of shade and spatial drought (Supplementary Fig. S1.5, *r* = −0.26, *p < *0.001), which is consistent with this possibility. Additionally, shade or spatial drought may release seedlings from other stresses. Shade may release seedlings from strong competition for space in gaps^50^. Drought may release seedlings from pathogens^51^ or damage from overland water flow on wet slopes during heavy rains^52^. In sum, the positive responses of some species to shade and drought highlight that species responses are not strictly synonymous with shade or drought tolerance. We studied natural variation in shade and drought conditions that incorporate various other biotic and abiotic influences on plants^11,53^. Thus, our approach allows for a more holistic understanding of the ecological mechanisms that affect seedling performance under natural shade and drought conditions, where their relevance should emerge.

### Trade-off between shade and drought responses

As expected, we found an interspecific trade-off between responses of species to shade and inter-annual drought; species that performed worse in the shade were not affected (or even performed better) during intense dry seasons and vice versa (Fig. 2a,b,d). The trade-off between shade and inter-annual drought resulted in part from the relative lack of doubly poorly adapted species and ‘superspecies’. Only four species performed significantly worse in both shade and drought (i.e. red dots in bottom-left quadrants of Fig. 2a,b,d). Such species would be outcompeted by species that are well-adapted to shade or drought, and hence would be unlikely to persist in the local community^6,54^. In contrast, although some species performed significantly better in either shade or drought, no species performed significantly better in both (i.e., no red dots in top-right quadrants of Fig. 2a,b,d). Such species would be akin to ‘superspecies’ (cf. Tilman^6^) that would dominate the community. However, many of the species in our study had responses that deviated considerably from the trade-off relationship, indicating that the trade-off is not absolute^10,11^.

Correlations between environmental conditions likely also contributed to the trade-off between responses to shade and inter-annual drought. Species that performed worse in the shade sometimes benefitted during severe dry seasons (bottom-right quadrant of Fig. 2). Reduced cloud cover and increased solar irradiance during severe dry seasons^30,31^ might contribute to this effect. Conversely, species that performed worse in years with severe dry seasons tended to have slightly (and sometimes significantly) better performance in the shade (top-left quadrants of Fig. 2a–d). The negative correlation between spatial variation in light and drought (Supplementary Fig. S1.5) might also contribute, with drought-sensitive species protected during drought by wetter conditions in the shade. In sum, the temporary release from shade during drought and from drought pressure in shaded sites may have constrained the evolution of combined tolerance to shade and drought^8^.

The mechanisms leading to the observed trade-off remain unknown. Experiments found interspecific trade-offs in seedling traits that may underlie a trade-off in species performance in shade versus drought^24,55,56^. For example, there was a trade-off in biomass allocation to leaves and roots^24^ as proposed by Smith and Huston^9^. However, low biomass allocation to leaves or roots does not preclude tolerance to shade or drought. For example, shade tolerant species can compensate for low aboveground biomass allocation by producing thinner leaves or reducing growth rates^13^. Other adaptations increase both shade and drought tolerance, including high wood and vessel density^16^. Still other traits increase shade or drought tolerance, but the effect of a trait that increases shade tolerance on drought tolerance and vice versa is unknown. For example, investment in carbohydrate storage and defence against herbivores and pathogens are associated with high seedling survival in the shade^5,57,58^. As another example, species may avoid drought through deciduousness^13,24^. These adaptations have metabolic costs^59–61^ and could contribute to the trade-off in shade and drought responses.

### Relationship between shade and drought responses and the fast–slow continuum

We hypothesised that responses to shade would correlate with the fast–slow continuum, which was not the case (Fig. 3a,b). This is surprising given the abundant evidence that fast species (with high growth and low survival rates) tend to be light-demanding, while slow species (with high survival and low growth rates) tend to be shade tolerant (e.g.^62,63^). As discussed previously, our ability to detect interspecific variation in shade responses is limited because the most light-demanding species were generally too rare to be included in our analyses (i.e. the paucity of species on the left side of Fig. 3a,b). In addition, species may respond differently to shade in terms of height growth versus diameter growth, for example if seedlings prioritize height growth until they capture enough light for diameter growth as saplings^64^. Species may also undergo ontogenetic shifts in shade tolerance^65^, although such ontogenetic shifts have proven to be very rare among species present in the BCI 50-ha plot^66–68^.

Species with a slow demographic strategy were more tolerant to severe dry seasons in terms of survival than species with a fast strategy (Fig. 3d). This is likely due to the high cost of drought adaptations (see *Discussion: Trade-off between shade and drought responses*). We did not expect a conservative strategy of drought-tolerant species, because we hypothesised that slow species would be shade tolerant and that shade and drought responses traded off. Yet, similar results have been found in northern hemisphere species that exhibited a trade-off in shade and drought tolerance; drought tolerance corresponded with conservative traits (long leaf life span and high leaf dry mass), while shade tolerance did not correlate with a fast–slow continuum because it involved both conservative (long leaf life span) and acquisitive (low leaf dry mass) traits^69^. Trait comparisons of the slow and fast species in our study can help to understand whether shade or drought tolerance affects the position of species on the fast–slow continuum most.

### Implications of the trade-off between shade and inter-annual drought tolerances

The trade-off between responses to shade and drought may have significant consequences for the future dynamics of tropical forests. As the climate changes, droughts are becoming more frequent and severe^70^, which is expected to cause a shift in species composition to species that perform better during drought^71,72^. As tropical forests receive more solar radiation during droughts^30,31^, species performing well during drought would be released from shaded conditions. Thus, the drought-shade trade-off could reinforce a shift to more drought-adapted and less shade-adapted species as observed in Ghana^73,74^.

It remains unclear how the trade-off may interact with other factors that may cause performance differences among species (e.g. nutrient availability or pest pressure). Evaluating trade-offs among responses to multiple stresses simultaneously can significantly improve our understanding of life-history strategies of species^33^. Thus, a next step would be to evaluate if responses of species to shade and drought are related to their responses to other factors, or if the latter responses form independent dimensions of species strategies in coping with multiple stresses.

## Methods

### Study site

We conducted this study in old-growth, lowland, moist tropical forest in the 50-ha Forest Dynamics Plot (FDP) on Barro Colorado Island (BCI), Panama (9.15°N, 79.85°W). Annual rainfall averages 2660 mm, with a pronounced dry season from mid-December until early May^75^. Approximately 10% of the crown area in the plot is deciduous during the dry season^76^. Severe dry seasons tend to occur during El Niño events, when the dry season generally starts early and ends late^31,77^. Soil water availability varies spatially with topography in the plot, with plateaus generally being drier than slopes^78,79^.

### Seedling censuses

We monitored height growth along the main stem and survival annually in the dry season (January until March) from 1994 to 2014 for all seedlings (no minimum size) at 200 permanent seedling census sites (see^80^ for details). Each site included three 1-m^2^ seedling plots (see^81^ for methods). The sites are situated along 2.7 km of trails in the 50-ha plot and cover all topographic habitats (cf.^82^) except stream sides. We excluded individuals that had resprouted or were visually damaged or infected by pathogens because this damage likely had a larger effect on performance than variation in shade or drought. We also excluded growth for individuals that were more than 200 cm tall, as their height was measured inaccurately. Finally, we excluded observations for census intervals that deviated more than 30 days from a full year.

### Quantification of shade and drought

We quantified shade intensity for all 200 sites using a shade index based on annual canopy censuses conducted in 12 years (1995–1996 and 2003–2012, data from Condit^83^). The canopy censuses were conducted on a 5 m grid across the 50-ha plot^84^. In each grid cell, presence or absence of vegetation was recorded with an ocular range finder in six height intervals: 0–2 m, 2–5 m, 5–10 m, 10–20 m, 20–30 m and ≥30 m. We assumed that vegetation casts shade as a 5 m diameter circle at the average height of the intervals (at 35 m height for the highest interval). We estimated the amount of shade cast in the understory (at 0.5 m height) using Beer’s law (i.e. a constant proportion of light removed by each layer present) and trigonometry (i.e. the angle of sky overshadowed by vegetation, see Rüger *et al*.^84^ for more details). The relative shade index (S) ranged from 109 to 218 (unitless, mean = 169, SD = 23) with increasing values representing deeper shade, i.e. lower light availability.

We quantified dry season intensity using the maximum cumulative water deficit (MCWD) of the dry season preceding the growth or survival observations (1994–2013, published in Condit *et al*.^85^). MCWD is the best predictor of species distributions along a regional rainfall gradient in Central Panama and strongly affects tree growth and mortality on BCI^85,86^. To derive MCWD, we calculated a daily water balance as rainfall minus potential evapotranspiration (PET). We used daily rainfall records from BCI and the average daily PET on BCI from the period 1994–2007, which we assumed to hold across years^85^. We summed daily balances for every possible set of consecutive days between 1 September and 1 July of the next year (encompassing one dry season). The most negative value, i.e. the extreme deficit equalled the MCWD of that year. MCWD ranged from −618 to −328 mm in the years with the most and least severe dry seasons, respectively (mean = −464 mm, SD = 95 mm). We multiplied MCWD by −1 so that larger values correspond to more severe drought. This index of dry season intensity (D_I_) captures inter-annual drought variation.

To quantify spatial drought variation, we measured dry season soil water potential (SWP) at the 200 seedling census sites at 15 cm depth (WP4C Dewpoint PotentiaMeter, Decagon Devices, Inc, Pullman WA, USA). As rooting depth of the seedlings was unknown, we took additional samples at 40 and 100 cm depth at 36 seedling sites and 66 sites along the border of the 50-ha plot, and confirmed that SWP at 15, 40 and 100 cm depth were positively correlated (*p* < 0.001, Supplementary Fig. S1.6). We completed SWP measurements three times in a moderate dry season (February, March and April 2015) and once in a severe dry season (March 2016). The latter dry season occurred during the 2015–16 El Niño event and was the third longest dry season recorded on BCI since 1954^75^. We excluded samples taken after a rain in April 2015, and outliers identified using soil water retention curves for a subsample of sites (see Supplementary Information and Fig. S2.1 for details). SWP measurements taken at the same sites were positively correlated among all four sampling rounds, indicating that spatial differences in SWP were consistent over time (*p* < 0.001, Supplementary Fig. S1.7). We calculated the median SWP per site across the four sampling periods to quantify spatial variation in water availability. The median SWP across the 200 sites ranged from −1.57 to 0.00 MPa at the driest and wettest site, respectively (mean = −0.39 MPa, SD = 0.27 MPa). We multiplied median SWP by −1 so that again larger values corresponded to drier conditions for our spatial drought index (D_S_).

### Estimating shade and drought responses

We analysed annual relative height growth rates (RGR), because it decreased monotonically with seedling height whereas absolute height growth varied nonlinearly with height. We calculated RGR as:

1 $${\rm{RGR}}=\frac{\mathrm{ln}\,({{\rm{height}}}_{2})-\,\mathrm{ln}\,({{\rm{height}}}_{1})}{{{\rm{t}}}_{2}-{{\rm{t}}}_{1}}$$

where height_2_ and height_1_ are the height measurements at times t_2_ and t_1_, respectively. As RGR was strongly right-skewed and contained negative values (preventing the use of a log transformation), we transformed RGR using a modulus transformation^87^:

2 $${{\rm{RGR}}}_{t}({\rm{\lambda }})=\{\begin{array}{cc}{{\rm{RGR}}}^{{\rm{\lambda }}} & {\rm{RGR}}\ge 0\\ -\{{(-{\rm{RGR}})}^{{\rm{\lambda }}}\} & {\rm{RGR}} < 0\end{array}$$

where RGR_*t*_ is the transformed RGR. This transformation effectively reduced skewness with λ values between 0.3 and 0.6 in a recent study of diameter growth of saplings and trees in the BCI 50-ha plot^85^. We used λ = 0.6, as this reduced skewness most effectively (i.e. it resulted in the smallest difference between median and mean RGR). Additionally, we excluded extreme RGR outliers using a modified z-score. This score indicates outliers using the distance of an observation from the median, divided by the median absolute deviation of all observations from the median (see Iglewicz and Hoaglin^88^ for details).

We quantified first-year survival for the year after a seedling was first recorded, discarding all seedlings present in the initial 1994 census because their ages are unknown. Our first-year survival estimates were not affected by the ephemeral mortality spike that follows immediately after germination, because seedlings were already three to seven months post germination at the start of the censuses in January. Using first-year survival rather than older seedlings allowed for larger sample sizes per species and more species to meet the minimum sample size to be included in the analyses (see below). This is especially relevant, because survival rates were low for many species (often below 50% in the first year, see Fig. S1).

We used a Bayesian approach to quantify species growth and survival responses to shade and drought. We modelled the transformed RGR_*t*_ of individual *i* of species *j* in site *s* and year *y* (*G*_*i,j,s,y*_) (cm cm^−1^ year^−1^) using a normal distribution with predicted growth g_*i,j,s,y*_ and standard deviation σ_*j*_:

3 $${{\rm{G}}}_{i,j,s,y} \sim {\rm{normal}}\,({{\rm{g}}}_{i,j,s,y},{{\rm{\sigma }}}_{j})$$

Independent variables were the shade (S_*s,y*_), spatial drought (D_S,*s*_) and inter-annual drought (D_I,*y*_) indices for site *s* and year *y* and seedling height (cm) of individual *i* at the beginning of the census interval in year *y* (H_*i,y*_):

4 $${g}_{i,j,s,y}={{\rm{\beta }}}_{0,j}+{{\rm{\beta }}}_{1,j}\times {{\rm{S}}}_{s,y}+{{\rm{\beta }}}_{2,j}\times {{\rm{D}}}_{{\rm{S}},s}+{{\rm{\beta }}}_{3,{\rm{j}}}\times {{\rm{D}}}_{{\rm{I}},y}+{{\rm{\beta }}}_{4,j}\times \,\mathrm{ln}\,({{\rm{H}}}_{i,y})+{{\rm{u}}}_{i}+{{\rm{u}}}_{s}+{{\rm{u}}}_{y}$$

where the species-specific coefficients describe the mean *RGR*_*t*_ (β_0,*j*_), the responses to shade (β_1,*j*_), spatial drought (β_2,*j*_) and inter-annual drought (β_3,*j*_) and the effect of height (β_4,*j*_). We did not impose hyperdistributions on the β parameters to prevent the abundant species from dominating the results. We included random effects for individual (u_*i*_), site (u_*s*_) and year (u_*y*_). We tested for an interaction between responses to shade and spatial drought (β_5,*j*_ × S_*s,y*_ × D_S,*s*_) and between responses to shade and inter-annual drought (β_6,*j*_ × S_*s,y*_ × D_S,*s*_), but we found few significant positive or negative interactions per parameter (≤5 species).

We modelled survival using a Bernoulli distribution in Eq. (3) and a logistic adaptation of Eq. (4). The survival model did not include a random effect for individual because we evaluated survival once per individual. As we did not have prior information, we used flat (non-informative) priors. We fitted models with the Bayesian inference software package RStan version 2.16.2^89^. We assessed the overall predictive power of the growth and survival models by calculating the proportion of explained variance (R^2^) following Gelman and Hill^90^. The Supplementary Information includes the Stan code, implementation procedures and model diagnostics.

For both models, the shade (β_1,*j*_) and drought responses (β_2,*j*_, and β_3,*j*_) represent the slope of the relationship between performance (growth or survival) and S, D_S_ and D_I_, respectively. Species performed significantly worse (negative slope) or better (positive slope) in higher shade or drought when the 95% credible interval (CI) excluded 0. We analysed responses for all freestanding, woody species that were abundant enough to estimate reliable parameter values. We included species with ≥50 growth observations or ≥100 survival observations. We used data from the 12 years with canopy censuses to quantify shade responses and data from all 20 years to quantify drought responses. For the eight years lacking canopy measurements, we estimated growth or survival responses to drought only by removing the shade response term (β_1,*j*_ × S_*s,y*_) from Eq. (4) (see model code in Supplementary Information for details). Due to the inclusion criteria and measurement constraints, we could estimate more growth than survival responses and more drought than shade responses (see *Results: Responses to shade and drought*).

### Testing the relationship between responses

We tested the relationships of species-specific responses to shade (β_1_) versus spatial (β_2_) and inter-annual drought (β_3_) using weighted Pearson correlations. We evaluated correlations for growth responses, survival responses and growth versus survival responses. For each species *j* and parameter *p*, we used the 95% credible interval of ${\beta }_{p,j}$ ($C{I}_{p,j}$) as a weight (weight_p,*j*_) as follows:

5 $${{\rm{weight}}}_{{\rm{p}},j}=1-\frac{{{\rm{CI}}}_{{\rm{p}},j}}{{\rm{\max }}\,({{\rm{CI}}}_{{\rm{p}}})}$$

The species with the widest CI_p,*j*_ (i.e. largest uncertainty) had weight zero, which we reset to half the weight of the species with the second widest CI_p,*j*_. As the weighted correlations required one weight per species, we used the mean of the weights of their respective shade (β_1,*j*_) and spatial (β_2_) or inter-annual drought response (β_3,*j*_).

### Relating responses to the fast–slow continuum

To test whether responses to shade and drought were related to demographic strategies, we evaluated relationships between responses to shade (β_1_), spatial drought (β_2_) or inter-annual drought (β_3_) and species positions along an independently quantified fast–slow continuum^33^. A low score on the first principal component axis of Rüger *et al*. (2018) corresponds to species with fast growth and low survival (i.e. fast species), and a high score corresponds to species with slow growth and high survival (i.e. slow species). We evaluated these relationships using weighted Pearson correlations as described above (see *Methods: Testing the relationship between responses*).We conducted all analyses in R version 3.4.1^91^.

## Supplementary information

Supplementary Information

## Data Availability

Seedling and inter-annual drought data are available on request via ForestGEO^80^ and Condit *et al*.^85^, respectively. Shade and soil water potential data are available from Condit^83^ and Kupers *et al*.^92^, respectively.
