# Supplementary material for: Performance of tropical forest seedlings under shade and drought: an interspecific trade-off in demographic responses
Source: Sci Rep. 2019 Dec 11;9:18784. doi: 10.1038/s41598-019-55256-x (PMC6906455; doi:10.1038/s41598-019-55256-x)
Supplement: Supplementary file 1 — Supplementary Information [file 41598_2019_55256_MOESM1_ESM.pdf]

# Performance of tropical forest seedlings under shade and drought: an interspecific trade-off in demographic responses

Stefan J. Kupers[1], Christian Wirth[1,2,3], Bettina M.J. Engelbrecht[4,5], Andrés Hernández[4], Richard Condit[6,7], S. Joseph Wright[4], Nadja Rüger[1,4]

[1] German Centre for Integrative Biodiversity Research (iDiv) Halle-Jena-Leipzig, Deutscher Platz 5e, 04103 Leipzig, Germany

[2] University of Leipzig, Systematic Botany and Functional Biodiversity, Johannisallee 21-23, 04103 Leipzig, Germany

[3] Max-Planck-Institute for Biogeochemistry, Hans-Knöll Str. 10, 07745 Jena, Germany

[4] Smithsonian Tropical Research Institute, Apartado 0843-03092, Balboa, Ancón, Panama

[5] Department of Plant Ecology, Bayreuth Center of Ecology and Environmental Research (BayCEER), University of Bayreuth, 95447 Bayreuth, Germany

[6] Field Museum of Natural History, IL 60605, USA

[7] Morton Arboretum, Lisle, IL 60532, USA

## SUPPLEMENTARY INFORMATION

## Contents

Supplementary figures and tables ........ 2

Soil water retention curves ........ 11

Implementation procedures and Stan code ........ 13

3.1 Model implementation and diagnostics ........ 13

3.2 Stan code ........ 14

References ........ 18

All species responses ........ 19

## 1. Supplementary figures and tables

Tables S1-S2 and Figure S1 present the responses of all 91 species in the study to shade and drought, and are included in this file from page 19 onwards.

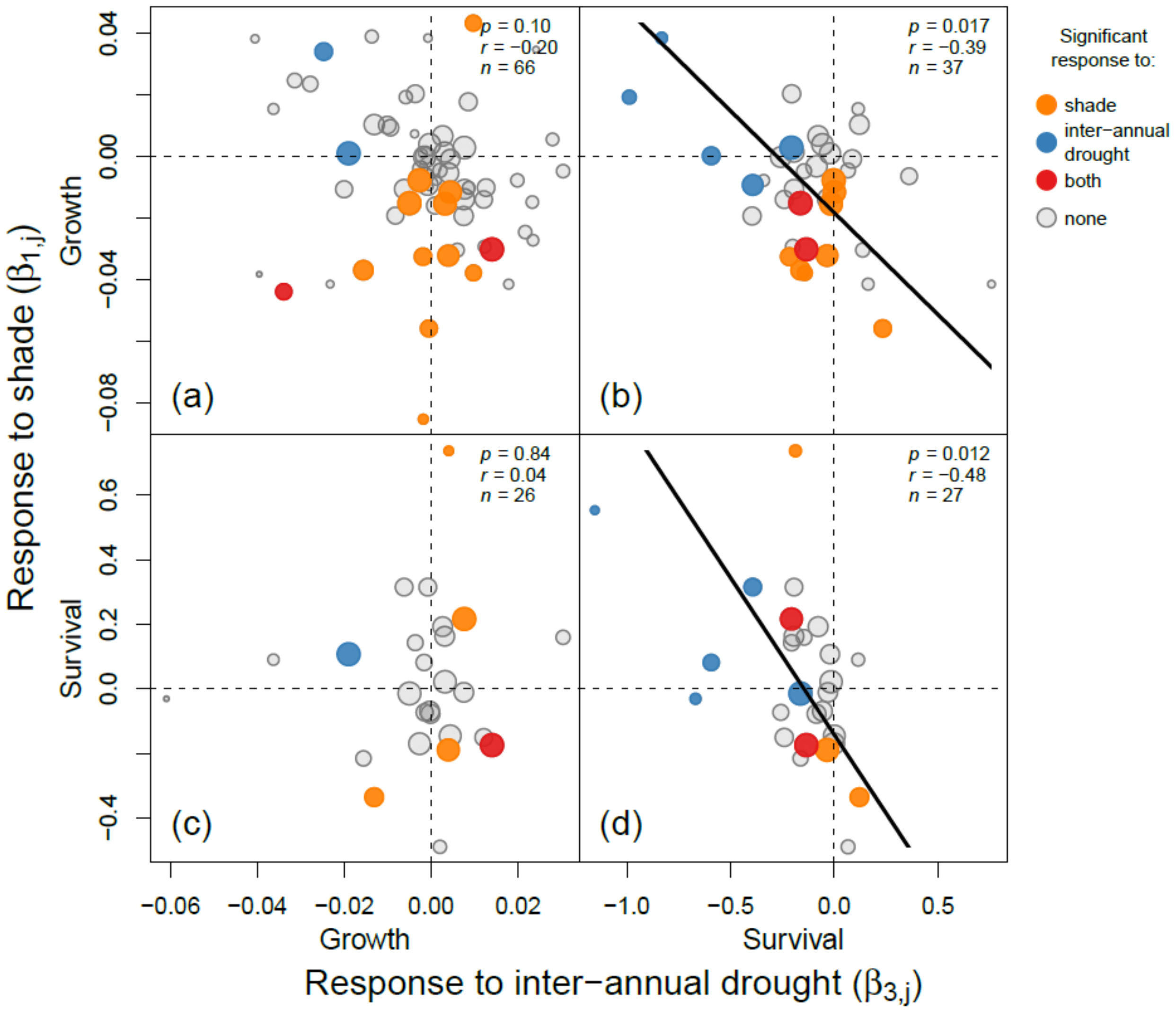


**Figure S1.1 Relationships between species responses to shade and inter-annual drought (i.e. dry season severity) for growth (a), survival (d), or growth versus survival (b,c) when individuals that resprouted, were visually damaged or infected by pathogens were included in the analysis. Solid lines indicate significant relationships ($p < 0.05$). Negative relationships indicate a trade-off between shade and drought responses. Correlations are weighted by the uncertainty in species tolerances (smaller dots have higher uncertainty and lower weight, see equation (5) in the main text). Colours identify species with insignificant (grey) or significant responses to shade (orange), inter-annual drought (blue) or both (red).**

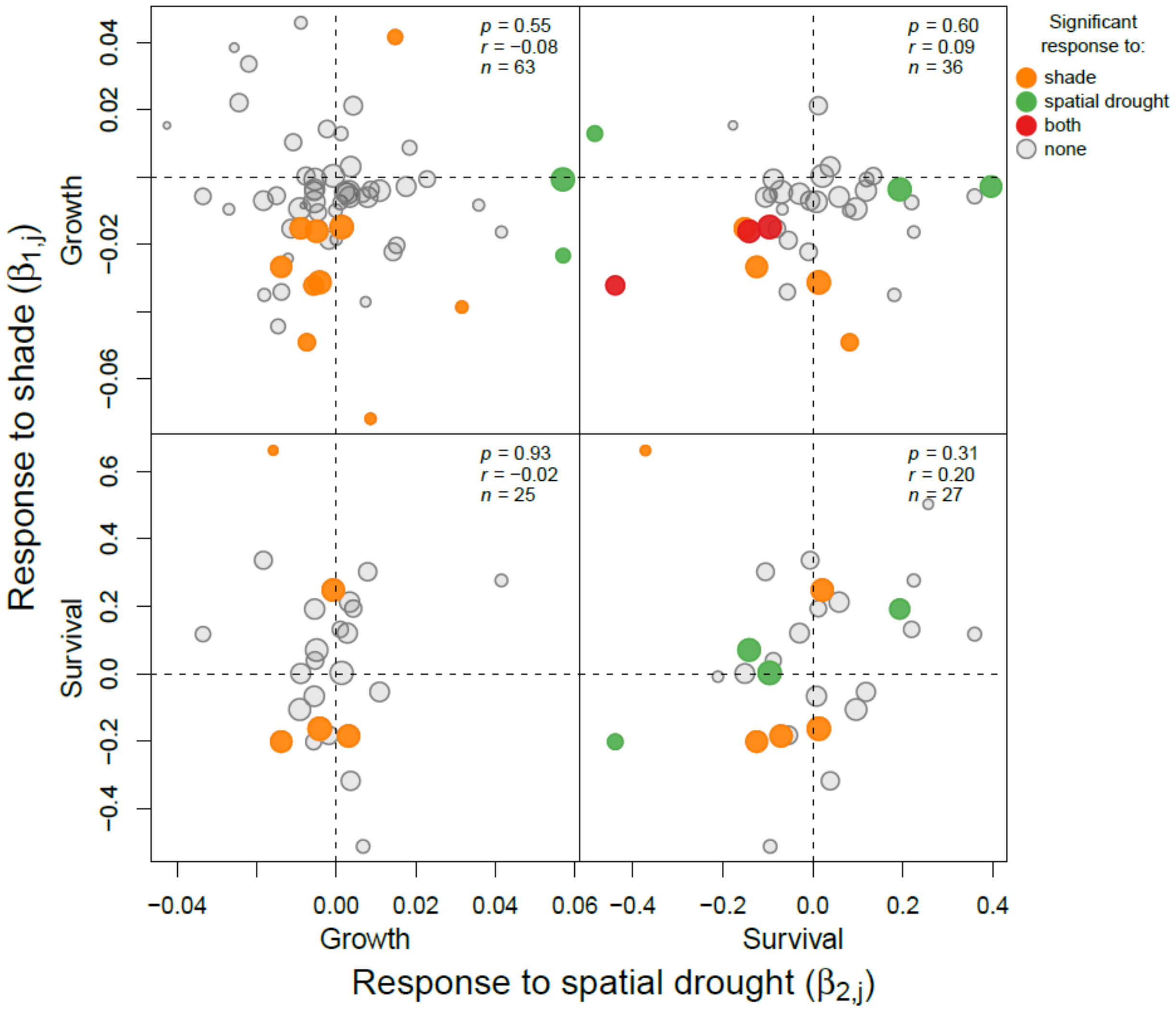


**Figure S1.2 Relationships between species responses to shade and spatial drought (i.e. the inverse of soil water potential) for growth (a), survival (d), or growth versus survival (b,c). None of the relationships was significant. Correlations are weighted by the uncertainty in species tolerances (smaller dots have higher uncertainty and lower weight, see equation (5) in the main text). Colours identify species with insignificant (grey) or significant responses to shade (orange), spatial drought (green) or both (red).**

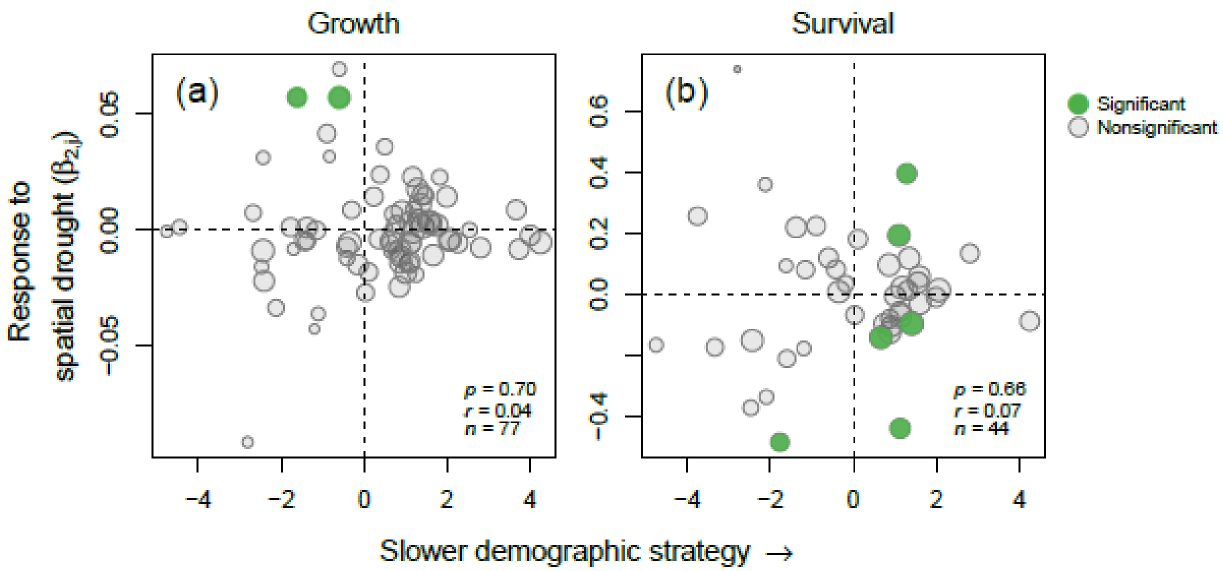


**Figure S1.3 Relationships between the fast-slow continuum and responses to spatial drought for (a) growth and (b) survival. The position of species along the continuum was quantified based on a weighted PCA of demographic rates (growth, survival, number of sapling recruits) of trees ≥ 1 cm dbh recorded in the BCI 50-ha plot[1]. Low and high scores correspond to species with fast and slow demographic strategies, respectively. Colours identify species with insignificant (grey) or significant responses to shade (orange) or spatial drought (green). Relationships were consistent when the fast-slow continuum was calculated using seedling performance and/or seed number additionally (see Table S1.2).**

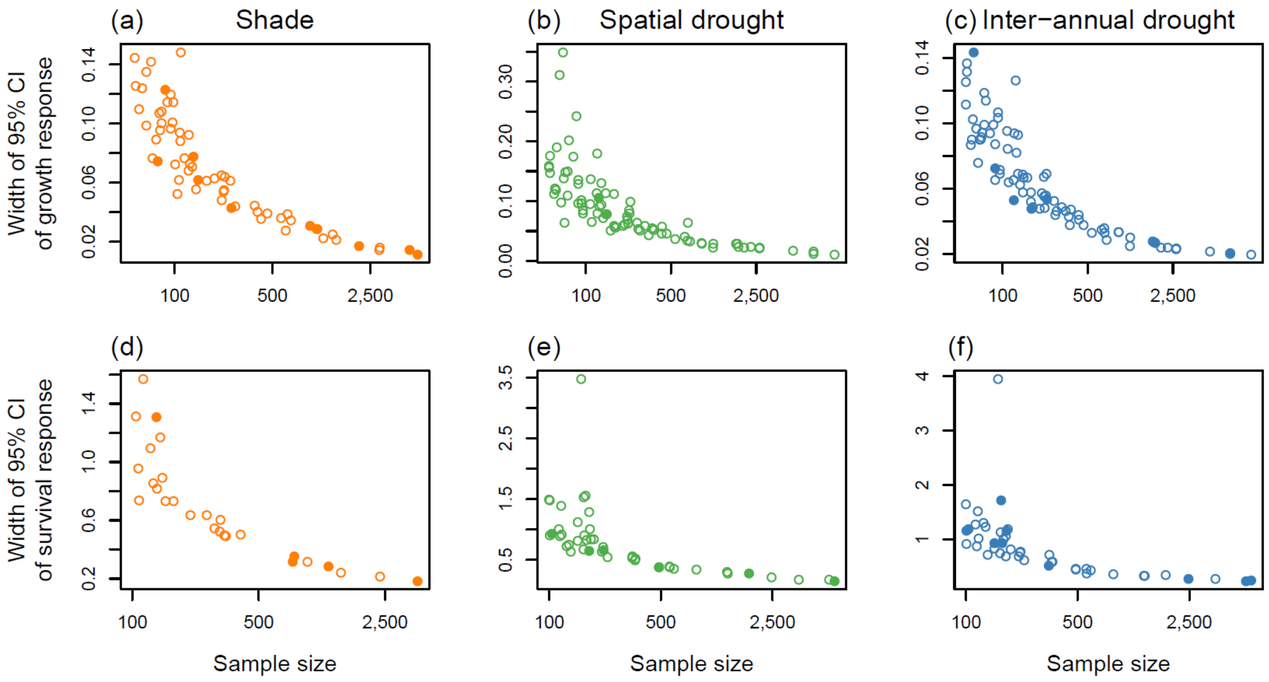


**Figure S1.4 Width of the 95% credible interval of growth responses (upper panels) and survival responses (lower panels) of species to shade (a,d), spatial drought (b,e) and inter-annual drought (c,f) versus sample size (i.e. the number of seedling observations of species in the respective models). Filled and unfilled circles represent species with significant and nonsignificant responses, respectively.**

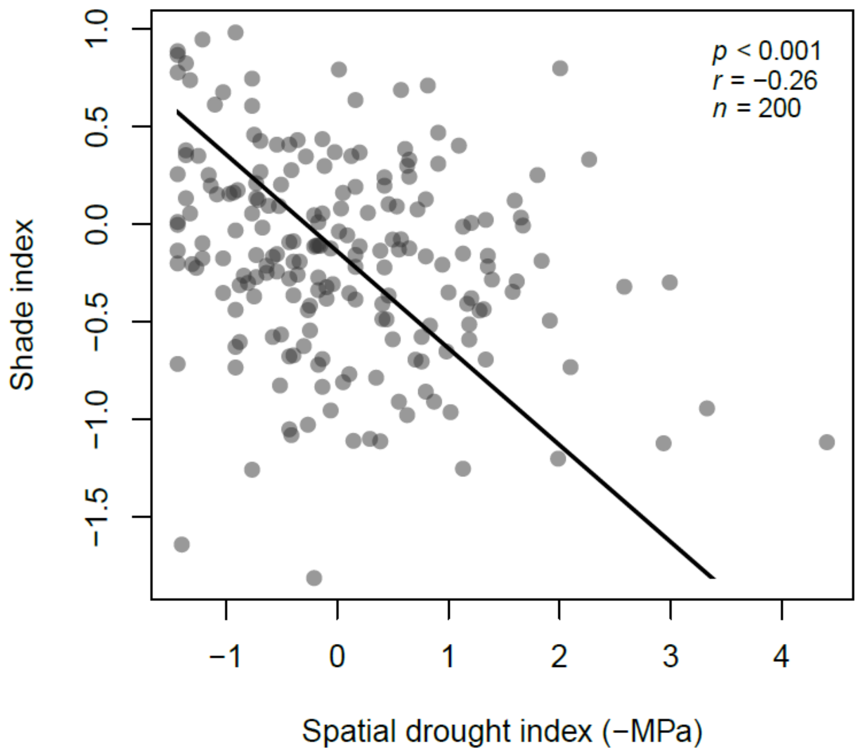


**Figure S1.5 Pearson correlation between shade index (means over all years) and spatial drought index at the 200 seedling census sites.**

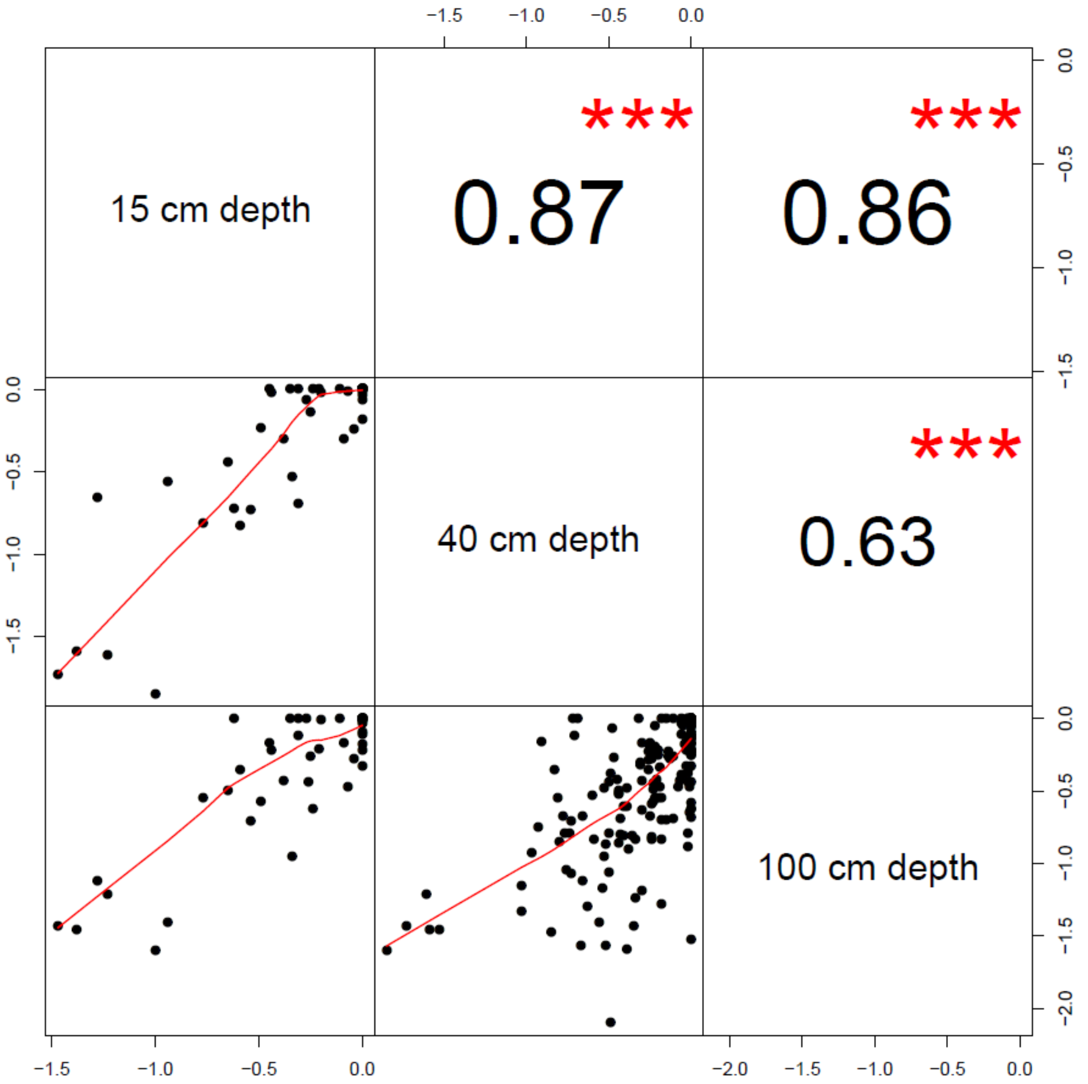


**Figure S1.6 Pearson correlations of soil water potentials (MPa) at 15, 40 and 100 cm depth. Samples were taken at 36 seedling census sites and 66 sites along on the border of the 50-ha plot and in a 10-ha plot bordering the full northern side of the 50-ha plot. *** All correlations are significant at $p$ < 0.001.**

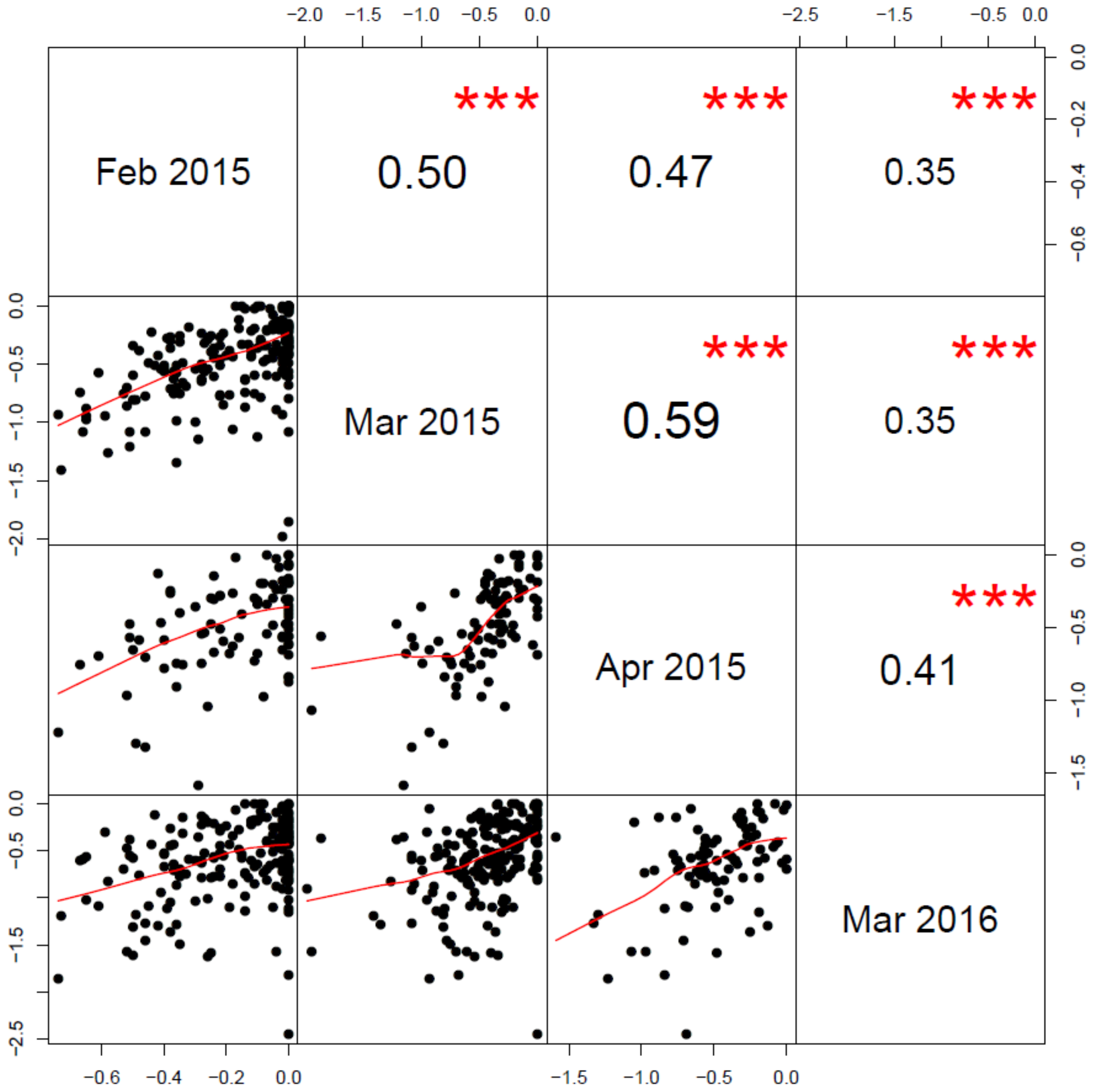


**Figure S1.7 Pearson correlations of soil water potentials (MPa) measured at the 200 seedling census sites among the four soil moisture sampling periods. *** All correlations are significant at *p* < 0.001.**

**Table S1.1 Numbers and percentages of species (in parentheses) with significant growth or survival responses to shade, drought and ln(height).**

| | Growth responses | | Survival responses | |
|---|---|---|---|---|
| | **Negative** | **Positive** | **Negative** | **Positive** |
| Shade ($\beta_1$) | 9 (16%) | 1 (2%) | 3 (11%) | 2 (7%) |
| Spatial drought ($\beta_2$) | 0 (0%) | 2 (2%) | 4 (9%) | 2 (4%) |
| Inter-annual drought ($\beta_3$) | 6 (7%) | 1 (1%) | 10 (22%) | 0 (0%) |
| ln(Height) ($\beta_4$) | 73 (86%) | 0 (0%) | 0 (0%) | 34 (76%) |

**Table S1.2 Correlation between shade and drought responses and the fast-slow continuum in the 50-ha plot on BCI[1]. Bold values indicate significant relationships.**

| Response | Model | Fast-slow axis* | *r* | *p* | *n* |
|---|---|---|---|---|---|
| Shade ($\beta_{1,j}$) | Growth | Trees | 0.11 | 0.42 | 60 |
| | | Trees + seedlings | 0.09 | 0.52 | 60 |
| | | Trees + seedlings + seeds | 0.05 | 0.73 | 50 |
| | Survival | Trees | -0.21 | 0.31 | 26 |
| | | Trees + seedlings | -0.18 | 0.38 | 26 |
| | | Trees + seedlings + seeds | -0.21 | 0.32 | 24 |
| Spatial drought ($\beta_{2,j}$) | Growth | Trees | 0.04 | 0.70 | 77 |
| | | Trees + seedlings | 0.04 | 0.74 | 77 |
| | | Trees + seedlings + seeds | 0.12 | 0.40 | 56 |
| | Survival | Trees | 0.07 | 0.66 | 44 |
| | | Trees + seedlings | 0.09 | 0.56 | 44 |
| | | Trees + seedlings + seeds | -0.23 | 0.20 | 34 |
| Inter-annual drought ($\beta_{3,j}$) | Growth | Trees | 0.07 | 0.54 | 77 |
| | | Trees + seedlings | 0.07 | 0.55 | 77 |
| | | Trees + seedlings + seeds | 0.07 | 0.61 | 56 |
| | Survival | Trees | 0.46 | **0.002** | 44 |
| | | Trees + seedlings | 0.48 | **0.001** | 44 |
| | | Trees + seedlings + seeds | 0.45 | **0.007** | 34 |

* Trees: recruitment, growth and survival of saplings and trees (≥ 1 cm dbh) in four canopy layers

Seedlings: growth and survival of seedlings (30-100 cm height)

Seeds: Seedling recruitment and seed number

## 2. Soil water retention curves

To identify outliers in soil water potential (SWP) measurements, we constructed soil water retention curves for 25 of the 200 seedling sites. After measuring SWP of each sample, we determined soil water content (SWC) from fresh mass (f) and dry mass (d) determined after 72 hours at 105°C (SWC = (f–d)/d). We then selected 25 samples from different seedling sites that covered all soil types (cf. Baillie *et al.*[2]) and topographic habitats (cf. Harms *et al.*[3]) of the 50-ha plot to construct soil water retention curves. By selecting sites with different edaphic characteristics, we ensured that the curves represented different possible combinations of SWP and SWC that can be expected at the sites. Thus, substantial deviation from the curves likely indicate measurement error.

To construct the retention curves, we first added distilled water to the soil sample until saturation (0 MPa). The soil was then gradually dried for approximately 30 minutes, weighted and measured for SWP with a WP4C Dewpoint PotentiaMeter (Decagon Devices, Inc, Pullman WA, USA). This was repeated 6-13 times until SWP was lower than -7 MPa. After this, gravimetric SWC was again determined as stated above. Finally, we fitted a third-order polynomial line through the observed SWP vs. SWC to construct each curve, correcting the line to 0 MPa when SWP was predicted to be positive.

To determine SWP outliers, we calculated standard deviation (SD) of the SWC (horizontally) and SWP (vertically) that were measured across all sampling rounds (excluding the observations used to construct the curves). Six SWP samples deviated by more than 1 SD from the most extreme retention curves (Fig. S2.1). These samples were considered outliers and excluded from the analysis. With the remaining samples, we calculated the median SWP per site and multiplied these by -1 to obtain our spatial drought index ($D_s$).

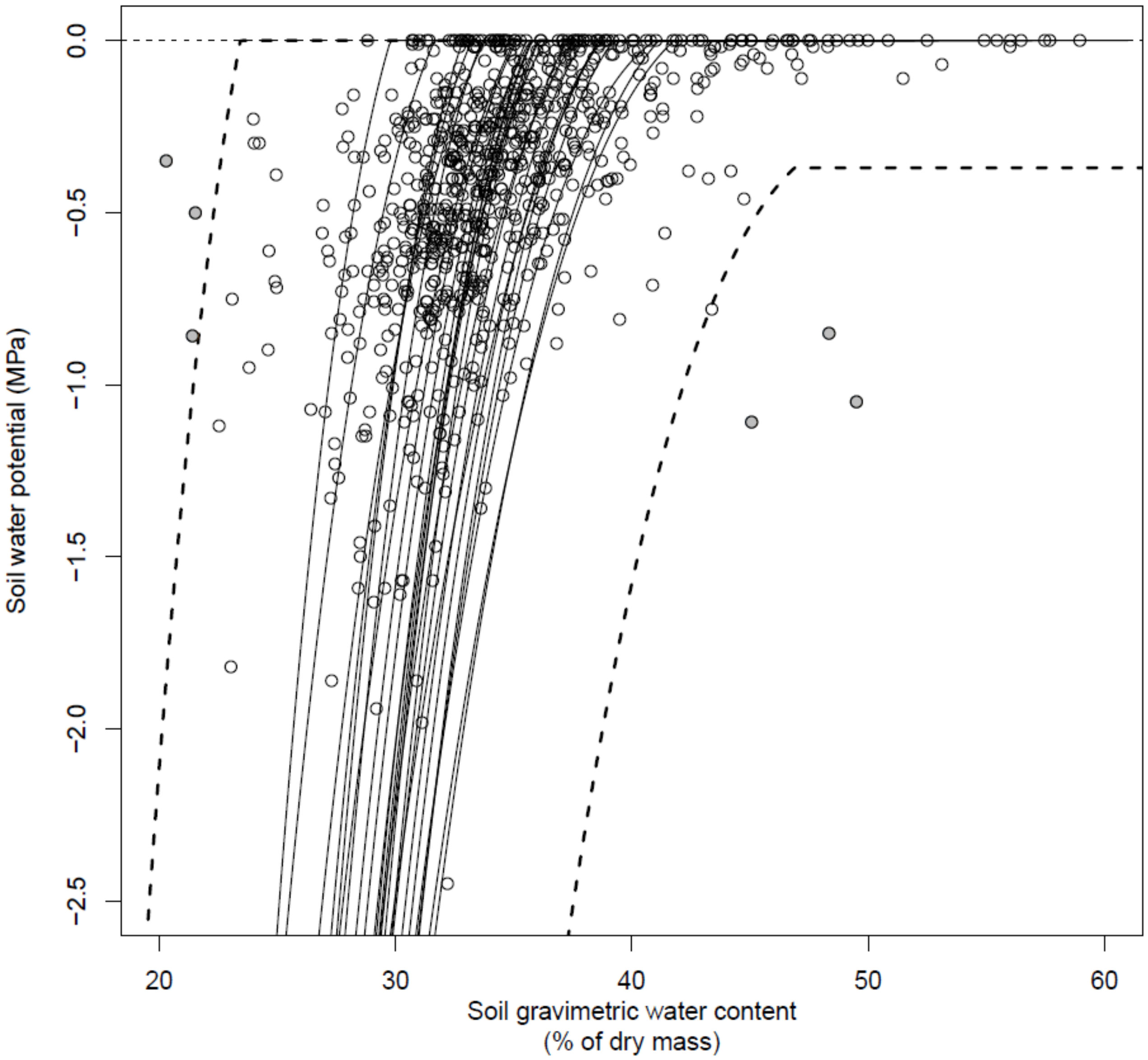


**Figure S2.1 Soil water retention curves (solid lines) constructed for 25 of the 200 seedling sites were used to determine outliers in observed SWP and SWC of all measurement rounds (dots). Outliers (grey dots) deviate more than 1 SD from the most extreme curves (dashed lines).**

## 3. Implementation procedures and Stan code

### *3.1 Model implementation and diagnostics*

Posterior distributions and error components were modelled using the Bayesian inference software package RStan version 2.16.2[4] in R version 3.4.1[5]. Convergence was monitored by running four chains with different starting values. We also used the potential scale reduction factor (*Rhat*) to check for convergence of the model. *Rhat* did not exceed 1.1 for any of the parameters in any model, indicating that the models converged[6]. To prevent divergent transitions, we adapted the initial step size, target acceptance rate and maximum treedepth where necessary. We centred and scaled all independent variables to mean 0 and standard deviation 1 to speed up convergence.

Chains of all models that we ran mixed well and converged in less than 100 iterations. For the main models, we used a burn-in period of 1000 iterations and an additional 2,500 iterations after burn-in per chain, giving a total of 10,000 iterations (2500 per chain) for the analyses.

For each model, the proportion of explained variance ($R^2$) was calculated following Gelman and Hill[6]:

$$\mathrm{R}^2 = 1 - \frac{\mathrm{E}(\mathrm{Var}(\epsilon))}{\mathrm{E}(\mathrm{Var}(y))}$$

where $\varepsilon$ are the model residuals (including all samples after warmup) and $y$ are the observed growth or survival of all observations.

***3.2 Stan code***

Growth model

```
data {
  int<lower=0>                              N_obs;
  int<lower=0>                              N_ind;
  int<lower=0>                              N_year;
  int<lower=0>                              N_sp;
  int<lower=0>                              N_trap;

  vector[N_obs]                             obs;

  vector[N_obs]                             cspatdrought;
  vector[N_obs]                             cinterannualdrought;
  vector[N_obs]                             cshade;
  vector[N_obs]                             clogheight;

  int<lower=0>                              species[N_obs];
  int<lower=0>                              ind[N_obs];
  int<lower=0>                              spind[N_ind];
  int<lower=0>                              repind[N_ind];

  int<lower=0>                              year[N_obs];
  int<lower=0>                              trap[N_obs];
}
parameters {
  vector<lower=-10,upper=10>[N_sp]      b0;
  vector<lower=-10,upper=10>[N_sp]      b1;
  vector<lower=-10,upper=10>[N_sp]      b2;
  vector<lower=-10,upper=10>[N_sp]      b3;
  vector<lower=-10,upper=10>[N_sp]      b4;

  vector<lower=-10,upper=10>[N_year]   a_year;
  vector<lower=-10,upper=10>[N_ind]     a_ind;
  vector<lower=-10,upper=10>[N_trap]     a_trap;

//hyperparameters
  vector<lower=0,upper=2>[N_sp]          sigma_p;
  vector<lower=0,upper=2>[N_sp]          sigma_aind;

  real<lower=0,upper=2>                  sigma_year;
  real<lower=0,upper=2>                  sigma_trap;
  real<lower=0,upper=2>                  sigma_sigp;
  real<lower=0,upper=2>                  sigma_sigaind;
}
model{
  vector[N_obs]                             pred;

  a_trap ~ normal(0,sigma_trap);
  a_year ~ normal(0,sigma_year);

  for( i in 1:N_ind){
    if(repind[i] == 1){
      a_ind[i] ~ normal(0,sigma_aind[spind[i]]);
    }
```

```
  }

  sigma_aind ~ normal(mu_sigaind, sigma_sigaind);
  sigma_p ~ normal(mu_sigp,sigma_sigp);


   for( i in 1:N_obs ){
        if(cshade[i]==-9){
                if( repind[ind[i]] == 1 ){
                        pred[i] = a_ind[ind[i]] + a_year[year[i]] + a_trap[trap[i]] + b0[species[i]] +
                                                b2[species[i]] * cspatdrought[i] +
                        b3[species[i]] * cinterannualdrought[i] + b4[species[i]] * clogheight[i];
                }
                else{
                        pred[i] =                a_year[year[i]] + a_trap[trap[i]] + b0 [species[i]] +
                                                b2[species[i]] * cspatdrought[i] +
                        b3[species[i]] * cinterannualdrought[i] + b4[species[i]] * clogheight[i];
                }
        }
        else{
            if( repind[ind[i]] == 1 ){
                        pred[i] = a_ind[ind[i]] + a_year[year[i]] + a_trap[trap[i]] + b0[species[i]] +
                        b1[species[i]] * cshade[i] + b2[species[i]] * cspatdrought[i] +
                        b3[species[i]] * cinterannualdrought[i] + b4[species[i]] * clogheight[i];
                }
                else{
                        pred[i] =                a_year[year[i]] + a_trap[trap[i]] + b0[species[i]] +
                        b1[species[i]] * cshade[i] + b2[species[i]] * cspatdrought[i] +
                        b3[species[i]] * cinterannualdrought[i] + b4[species[i]] * clogheight[i];
                }
         }
        obs[i] ~ normal(pred[i], sigma_p[species[i]]);
        }
}
```

Survival model

```
data {
  int<lower=0>                          N_ind;
  int<lower=0>                          N_year;
  int<lower=0>                          N_trap;
  int<lower=0>                          N_sp;

  int<lower=0,upper=1>                  alive[N_ind];

  vector[N_ind]                         cspatdrought;
  vector[N_ind]                         cinterannualdrought;
  vector[N_ind]                         cshade;
  vector[N_ind]                         clogheight;

  int<lower=0>                          species[N_ind];
  int<lower=0>                          year[N_ind];
  int<lower=0>                          trap[N_ind];
}
parameters {
  real<lower=-10,upper=10>              b0[N_sp];
  real<lower=-10,upper=10>              b1[N_sp];
  real<lower=-10,upper=10>              b2[N_sp];
  real<lower=-10,upper=10>              b3[N_sp];
  real<lower=-10,upper=10>              b4N_sp];

  real<lower=-10,upper=10>              a_year[N_year];
  real<lower=-10,upper=10>              a_trap[N_trap];

//hyperparameters
  real<lower=0,upper=2>                 sigma_year;
  real<lower=0,upper=2>                 sigma_trap;
}
transformed parameters {
vector[N_ind]                           theta;
  real s;

  for (i in 1:N_ind){
        if(cshade[i]==-9){
                s[i] =  a_year[year[i]] + a_trap[trap[i]] + b0[species[i]] +
                b2[species[i]] * cspatdrought[i] + b3[species[i]] * cinterannualdrought[i] +
                b4[species[i]] * clogheight[i];
    }else{
                s[i] =  a_year[year[i]] + a_trap[trap[i]] + b0[species[i]] + b1[species[i]] * cshade[i] +
                b2[species[i]] * cspatdrought[i] + b3[species[i]] * cinterannualdrought[i] +
                b4[species[i]] * clogheight[i];
        }
        theta[i] = 1/(1+exp(-s));
  }
}
model {
  for(i in 1:N_trap){
```

```
        a_trap[i] ~ normal(0,sigma_trap);
        }

  for(i in 1:N_year){
        a_year[i] ~ normal(0,sigma_year);
        }

  for (i in 1:N_ind){
    alive[i] ~ bernoulli(theta[i]);
  }
}
```

## References


1 Rüger, N. *et al.* Beyond the fast–slow continuum: demographic dimensions structuring a tropical tree community. *Ecology Letters* **21**, 1075-1084 (2018).

2 Baillie, I., Elsenbeer, H., Barthold, F., Grimm, R. & Stallard, R. Semi-detailed soil survey of Barro Colorado Island, Panama. 54 pp., https://biogeodb.stri.si.edu/bioinformatics/bci_soil_map/ (Smithsonian Tropical Research Institute, 2007).

3 Harms, K. E., Condit, R., Hubbell, S. P. & Foster, R. B. Habitat associations of trees and shrubs in a 50-ha Neotropical forest plot. *Journal of Ecology* **89**, 947-959 (2001).

4 Stan Development Team. RStan: the R interface to Stan, version 2.16.2. http://mc-stan.org (2017).

5 R Core Team. R: A language and environment for statistical computing. R Foundation for Statistical Computing: Vienna, Austria, https://www.R-project.org/ (2017).

6 Gelman, A. & Hill, J. Data analysis using regression and multilevel/hierarchical models. 625 pp. (Cambridge University Press, 2007).

## 4. All species responses

Here we provide Figure S1, which visualises all species responses to shade and drought. We also provide Tables S1-S2 with the parameter estimates and 95% credible intervals for all species.

**Figure S1 The following pages show observed and fitted relative growth rate (RGR, upper panels) and survival rate (lower panels) against shade (a,d), spatial drought (b,e) and inter-annual drought (c,f) for all species at their mean seedling height. Solid and dotted lines indicate significant and non-significant responses of species to shade or drought, respectively. Large dots represent mean observed growth or survival for ten shade or drought classes, each containing 10% of the individuals of the species (fewer than 10 dots indicate that a species occurs in a limited set of sites or years). Lines show fitted growth and survival with increasing shade (a,d, red), spatial drought (b,e, yellow) and inter-annual drought (c,f, blue), at mean values of the other predictors. Lines with a different colour than the large dots represent 1 SD increase in shade (red), spatial drought (yellow) or inter-annual drought (blue). Panels without data indicate that a species did not reach the selection criteria for that particular relationship (see *Methods: Estimating shade and drought responses*).**

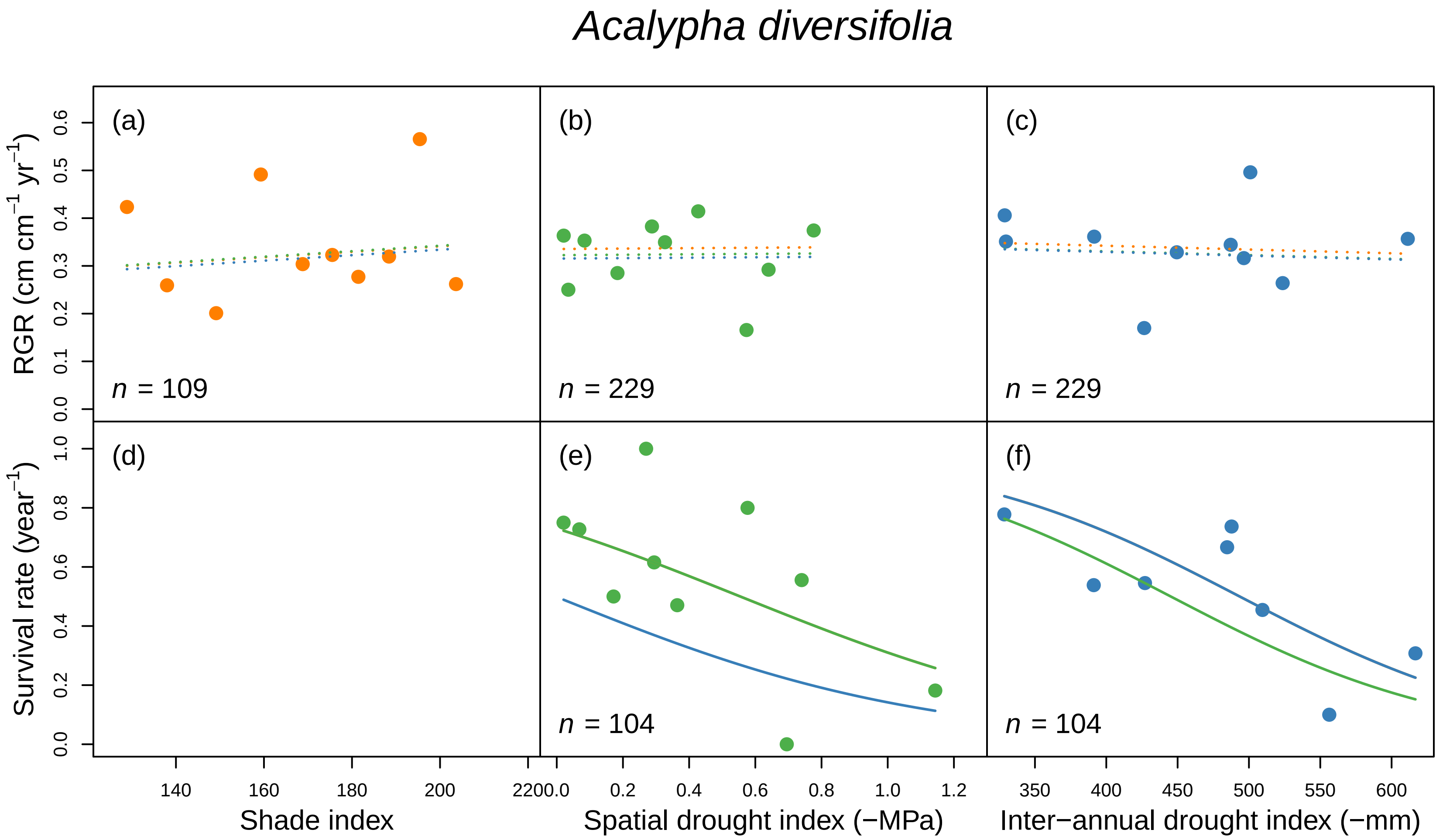
Acalypha diversifolia
(a)
(b)
(c)
(d)
(e)
(f)
n = 109
n = 229
n = 229
n = 104
n = 104
RGR (cm cm−1 yr−1)
Survival rate (year−1)
Shade index
Spatial drought index (−MPa)
Inter−annual drought index (−mm)

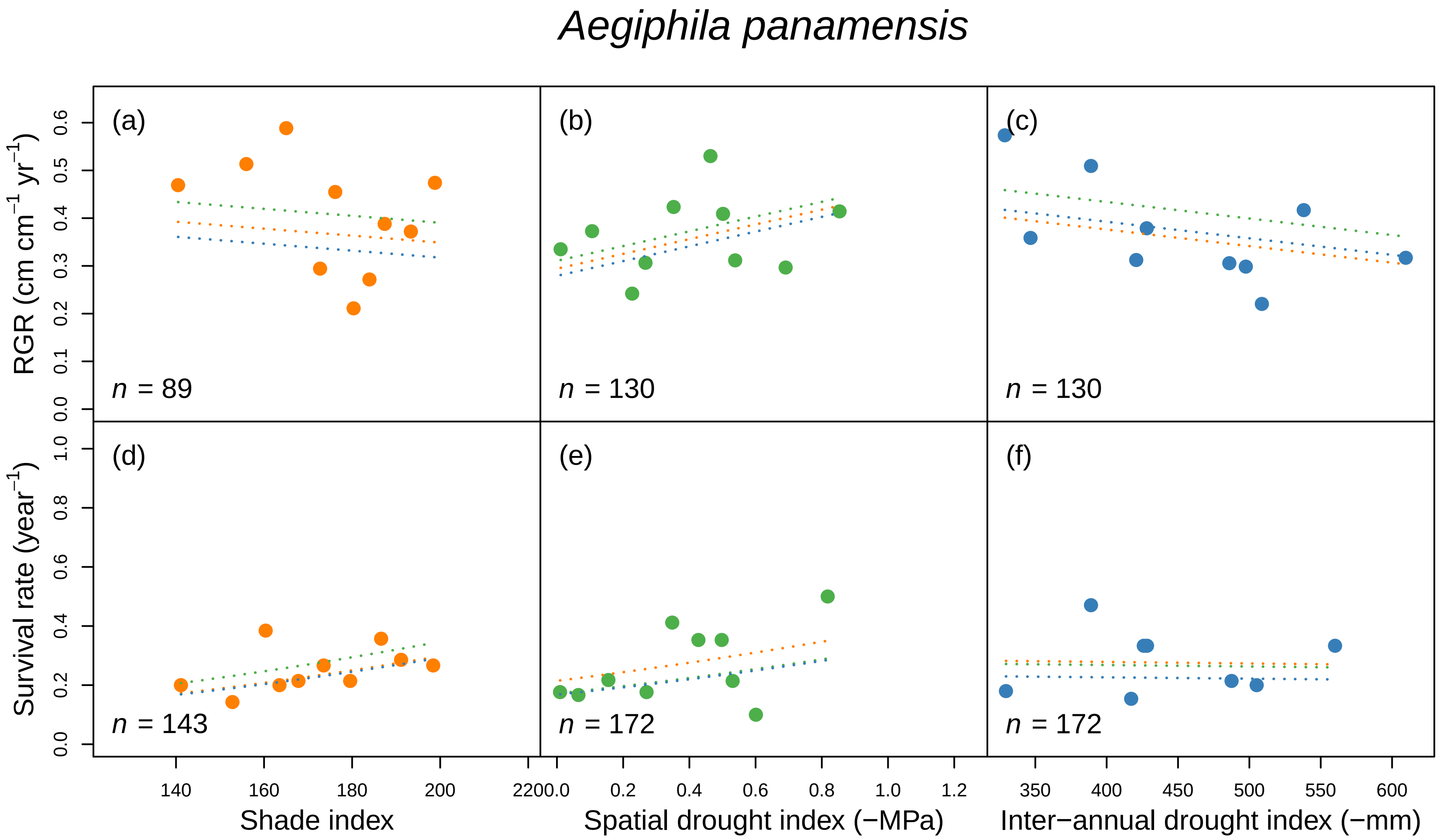

Aegiphila panamensis
(a)
(b)
(c)
(d)
(e)
(f)
n = 89
n = 130
n = 130
n = 143
n = 172
n = 172
RGR (cm cm−1 yr−1)
Survival rate (year−1)
0.0
0.1
0.2
0.3
0.4
0.5
0.6
0.0
0.2
0.4
0.6
0.8
1.0
140
160
180
200
220
0.0
0.2
0.4
0.6
0.8
1.0
1.2
350
400
450
500
550
600
Shade index
Spatial drought index (−MPa)
Inter−annual drought index (−mm)

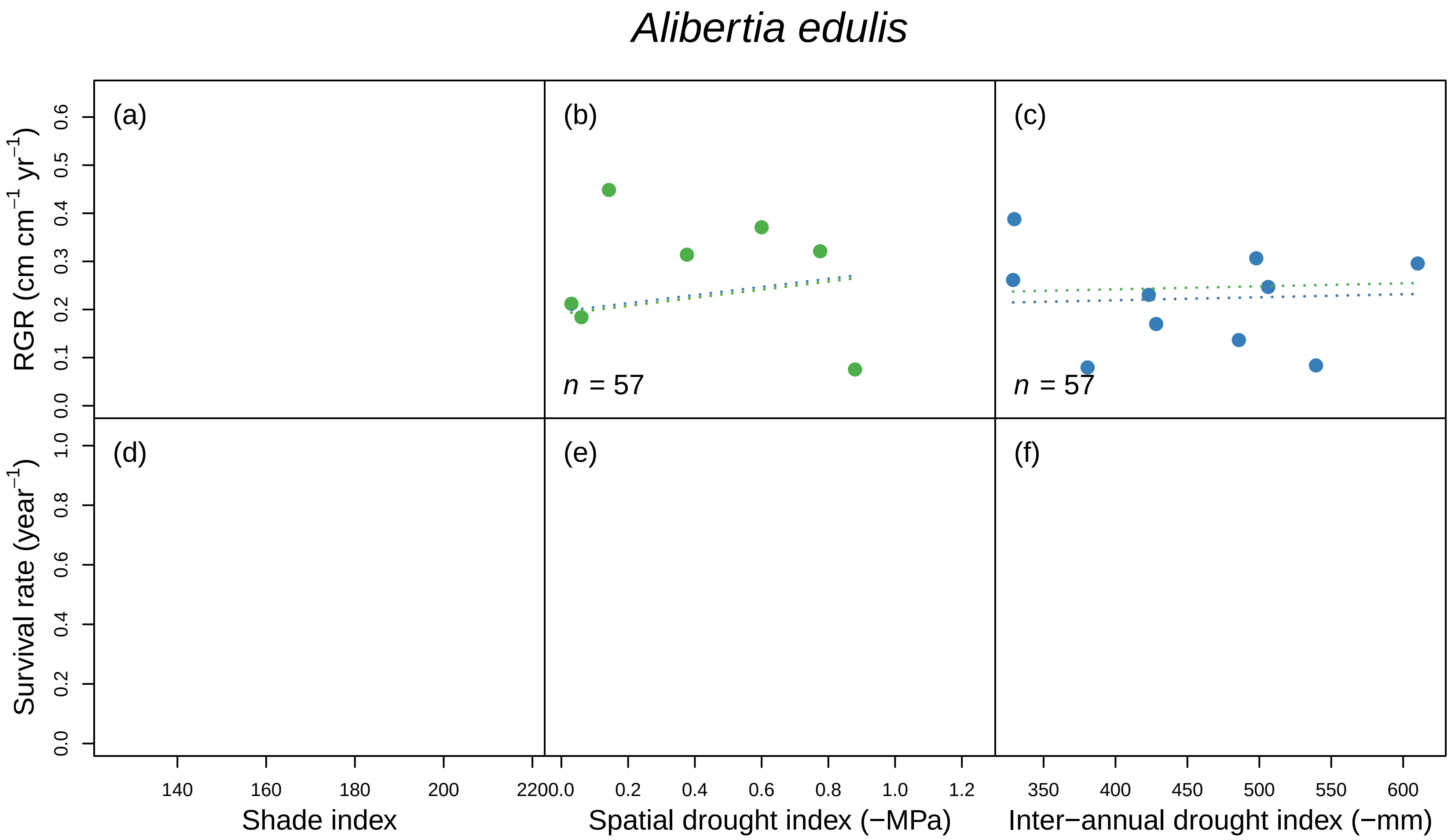

Alibertia edulis
(a)
(b)
(c)
(d)
(e)
(f)
n = 57
n = 57
RGR (cm cm−1 yr−1)
Survival rate (year−1)
0.0
0.1
0.2
0.3
0.4
0.5
0.6
0.0
0.2
0.4
0.6
0.8
1.0
140
160
180
200
220
0.0
0.2
0.4
0.6
0.8
1.0
1.2
350
400
450
500
550
600
Shade index
Spatial drought index (−MPa)
Inter−annual drought index (−mm)

*Allophylus psilospermus*

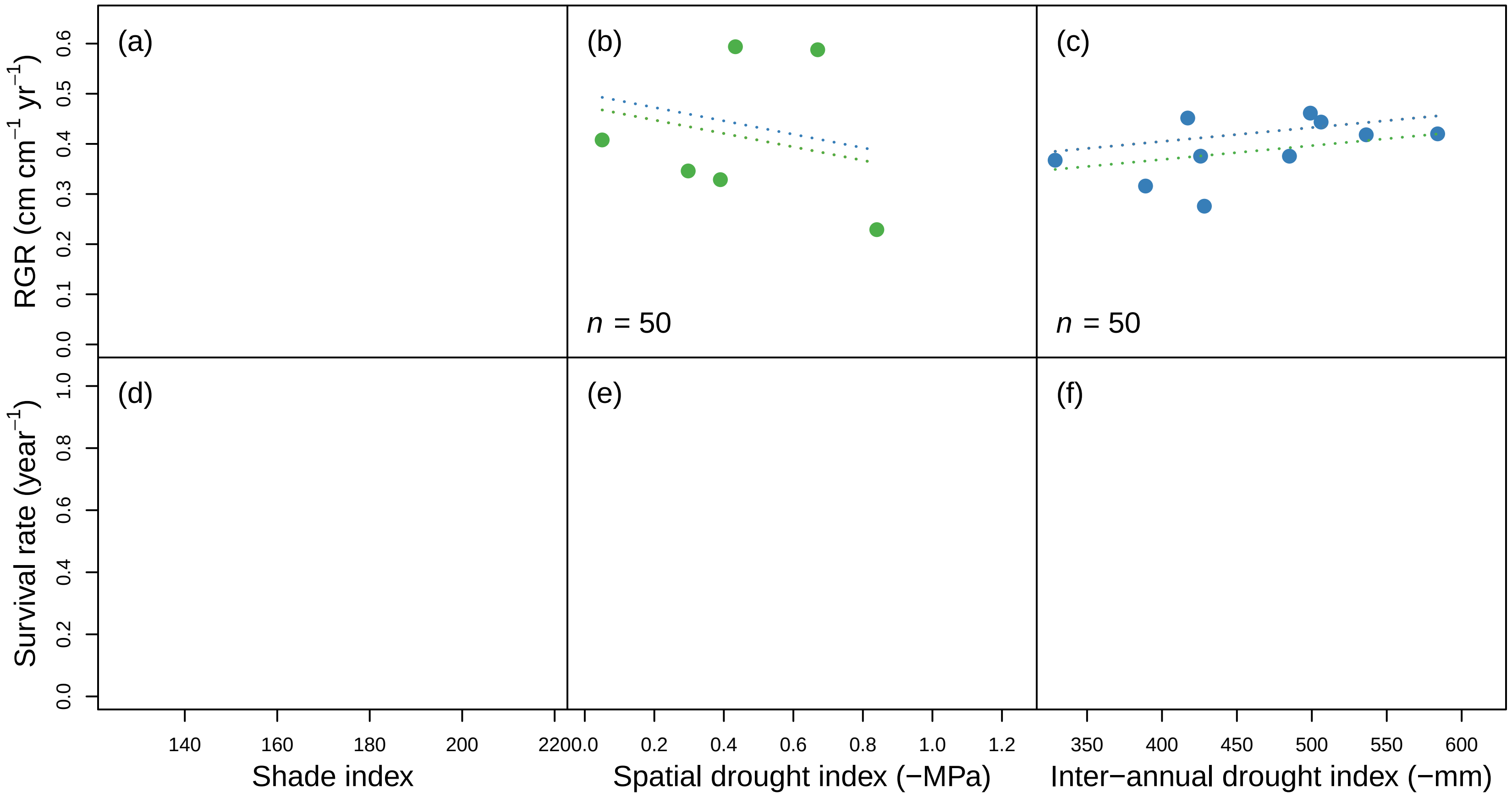

# *Alseis blackiana*

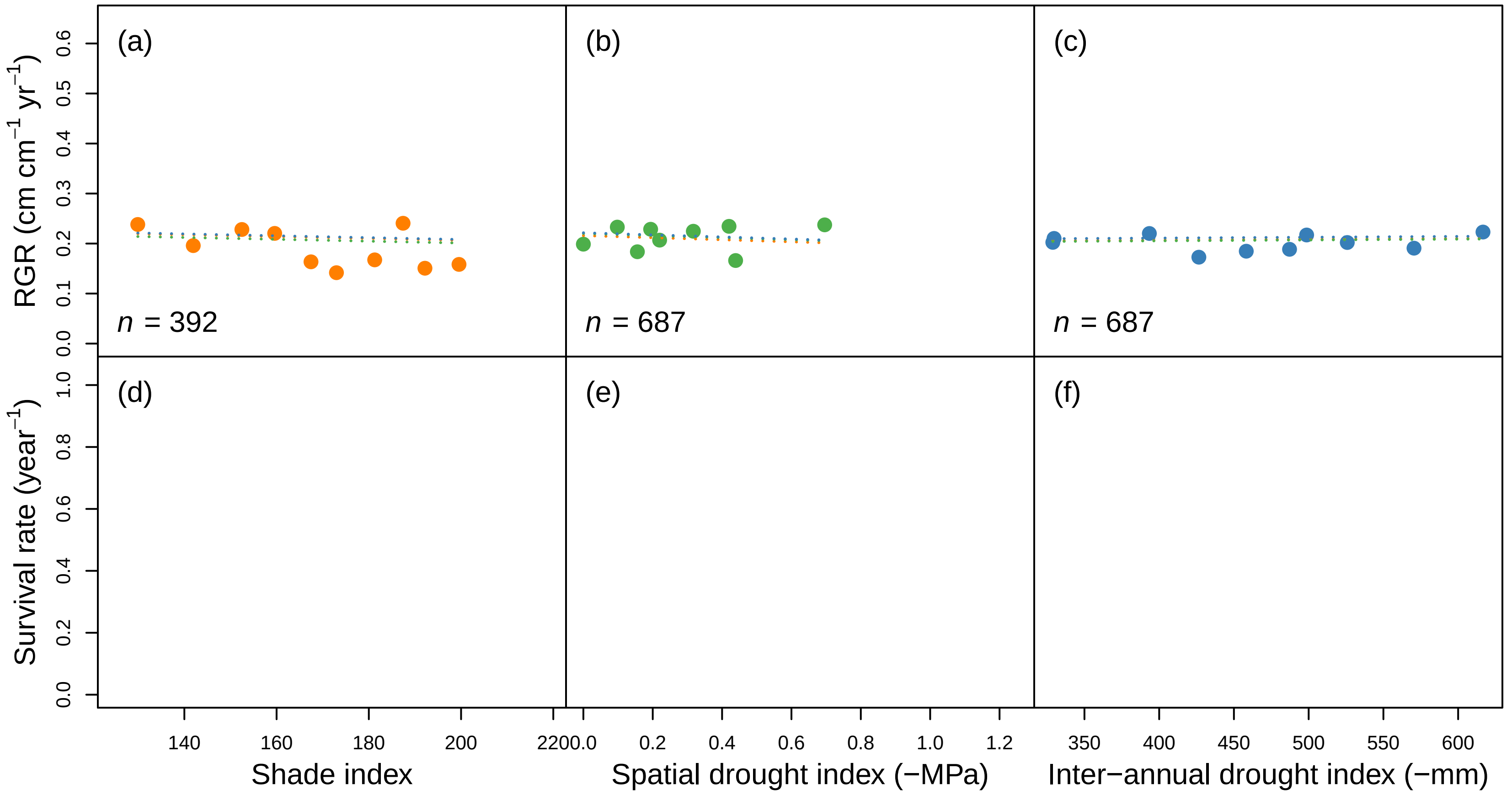

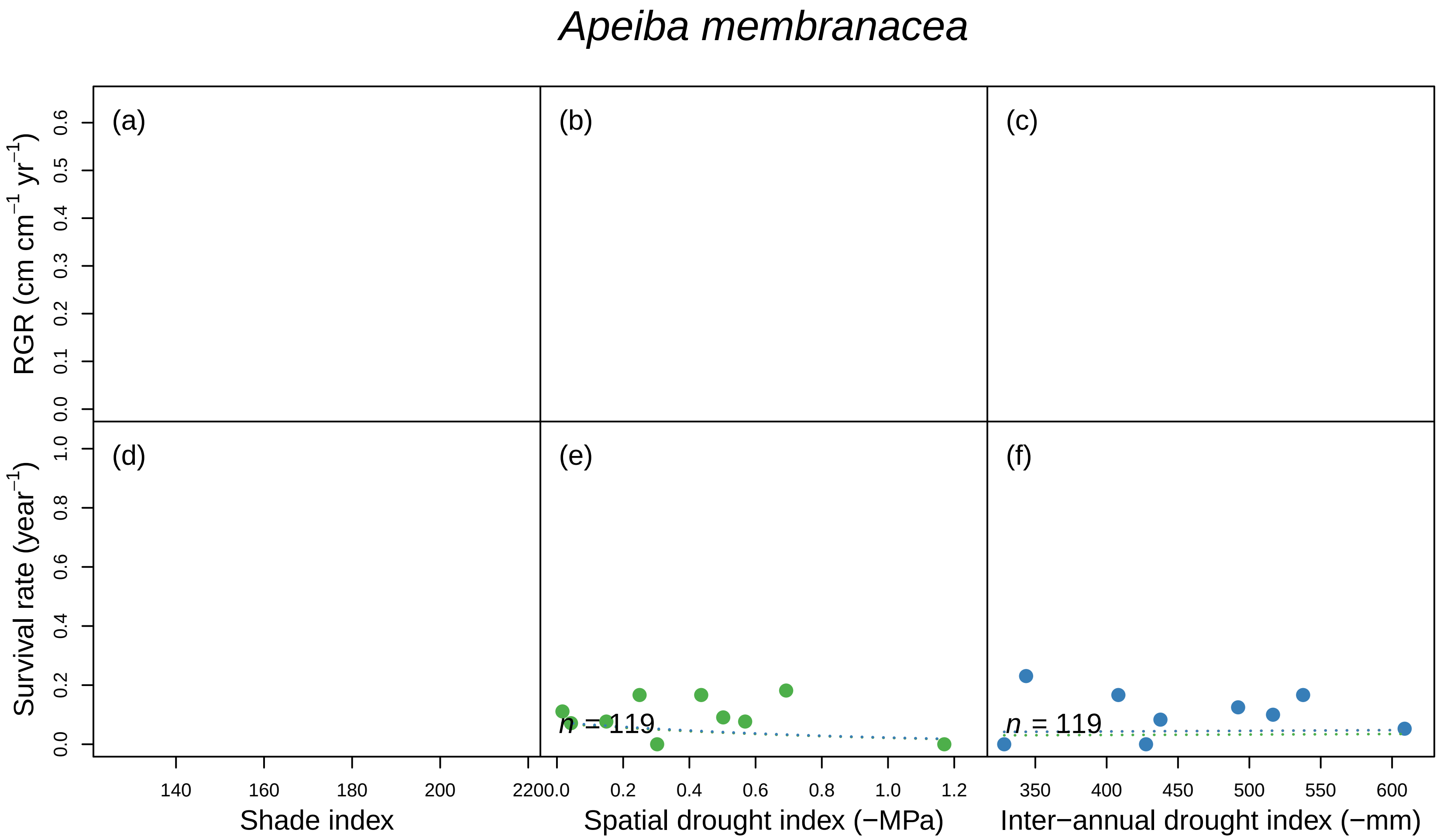
Apeiba membranacea
(a)
(b)
(c)
(d)
(e)
(f)
RGR (cm cm−1 yr−1)
Survival rate (year−1)
n = 119
n = 119
Shade index
Spatial drought index (−MPa)
Inter−annual drought index (−mm)

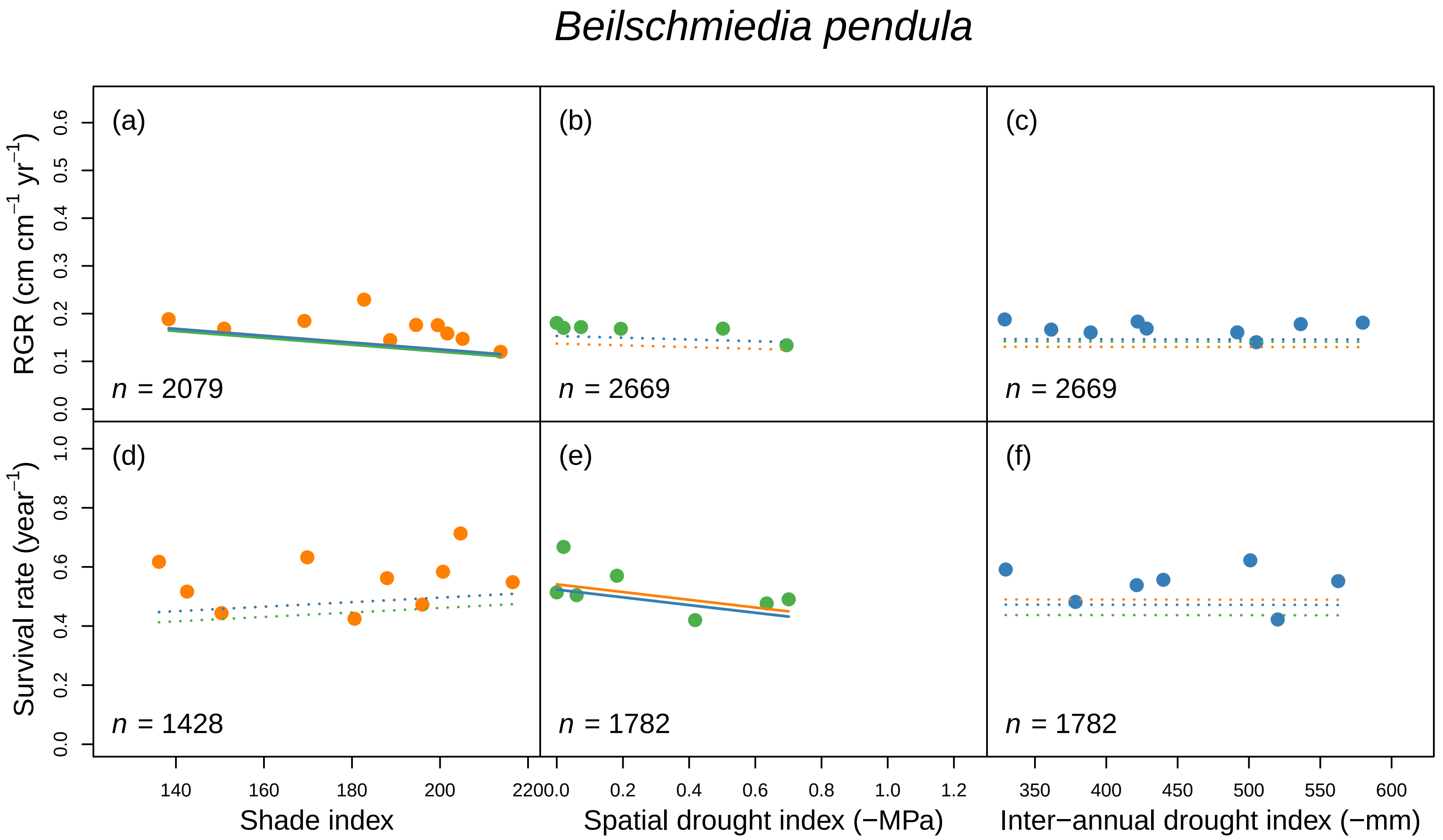
Beilschmiedia pendula
RGR (cm cm−1 yr−1)
Survival rate (year−1)
(a)
(b)
(c)
(d)
(e)
(f)
n = 2079
n = 2669
n = 2669
n = 1428
n = 1782
n = 1782
Shade index
Spatial drought index (−MPa)
Inter−annual drought index (−mm)

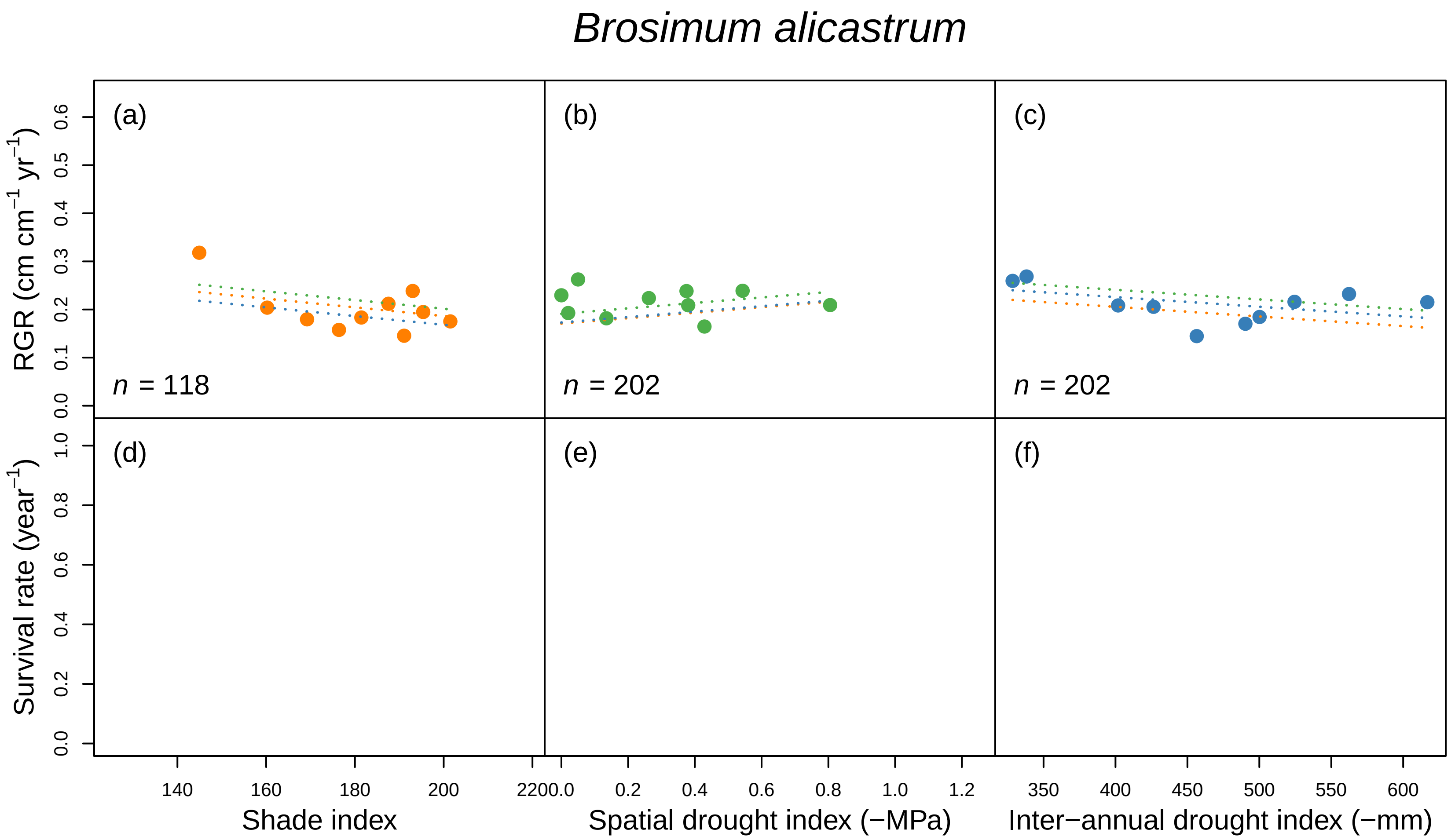

Brosimum alicastrum
(a)
(b)
(c)
(d)
(e)
(f)
n = 118
n = 202
n = 202
RGR (cm cm−1 yr−1)
Survival rate (year−1)
0.0
0.1
0.2
0.3
0.4
0.5
0.6
0.0
0.2
0.4
0.6
0.8
1.0
140
160
180
200
220
0.0
0.2
0.4
0.6
0.8
1.0
1.2
350
400
450
500
550
600
Shade index
Spatial drought index (−MPa)
Inter−annual drought index (−mm)

# *Calophyllum longifolium*

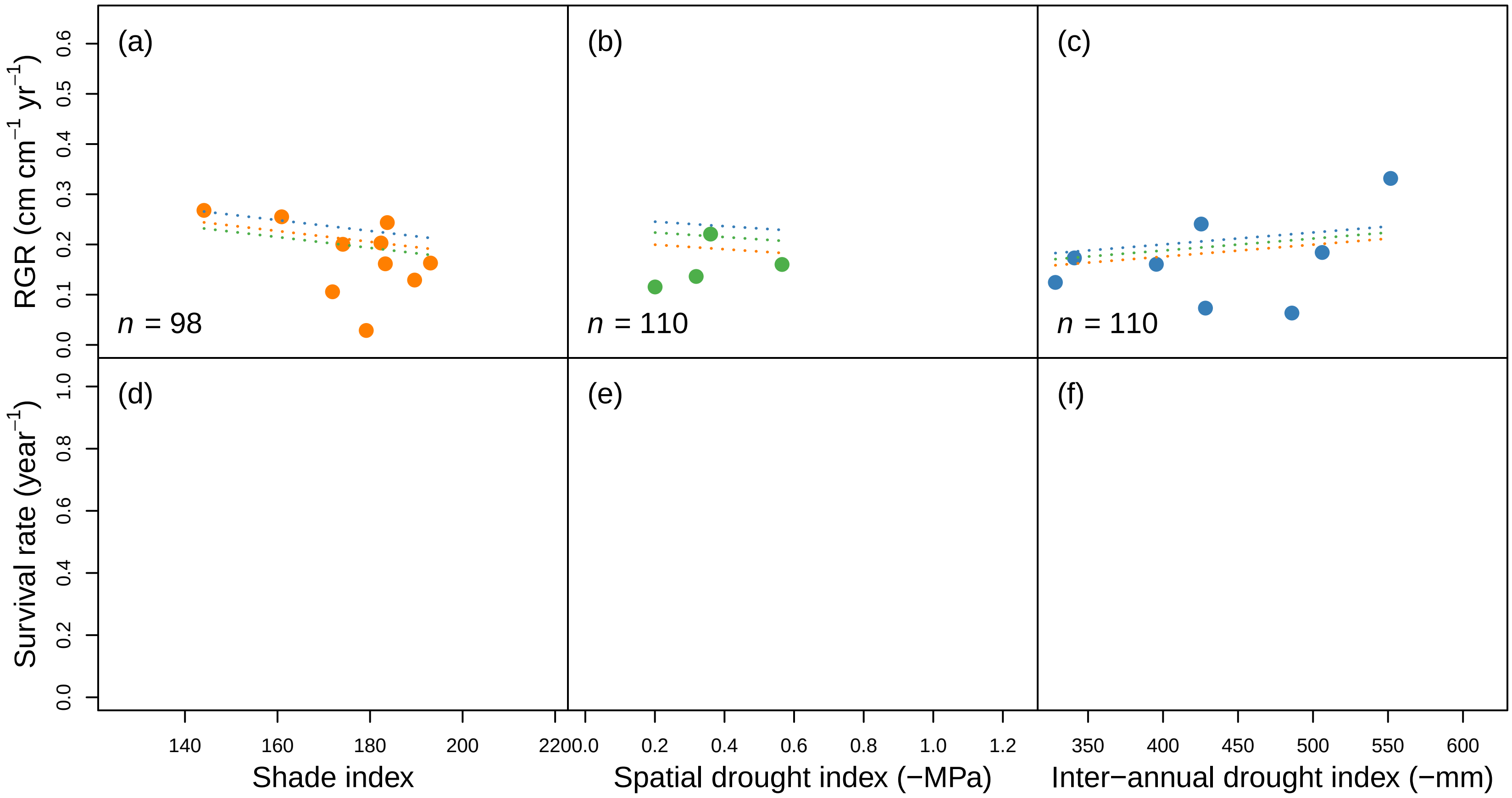

# *Capparis frondosa*

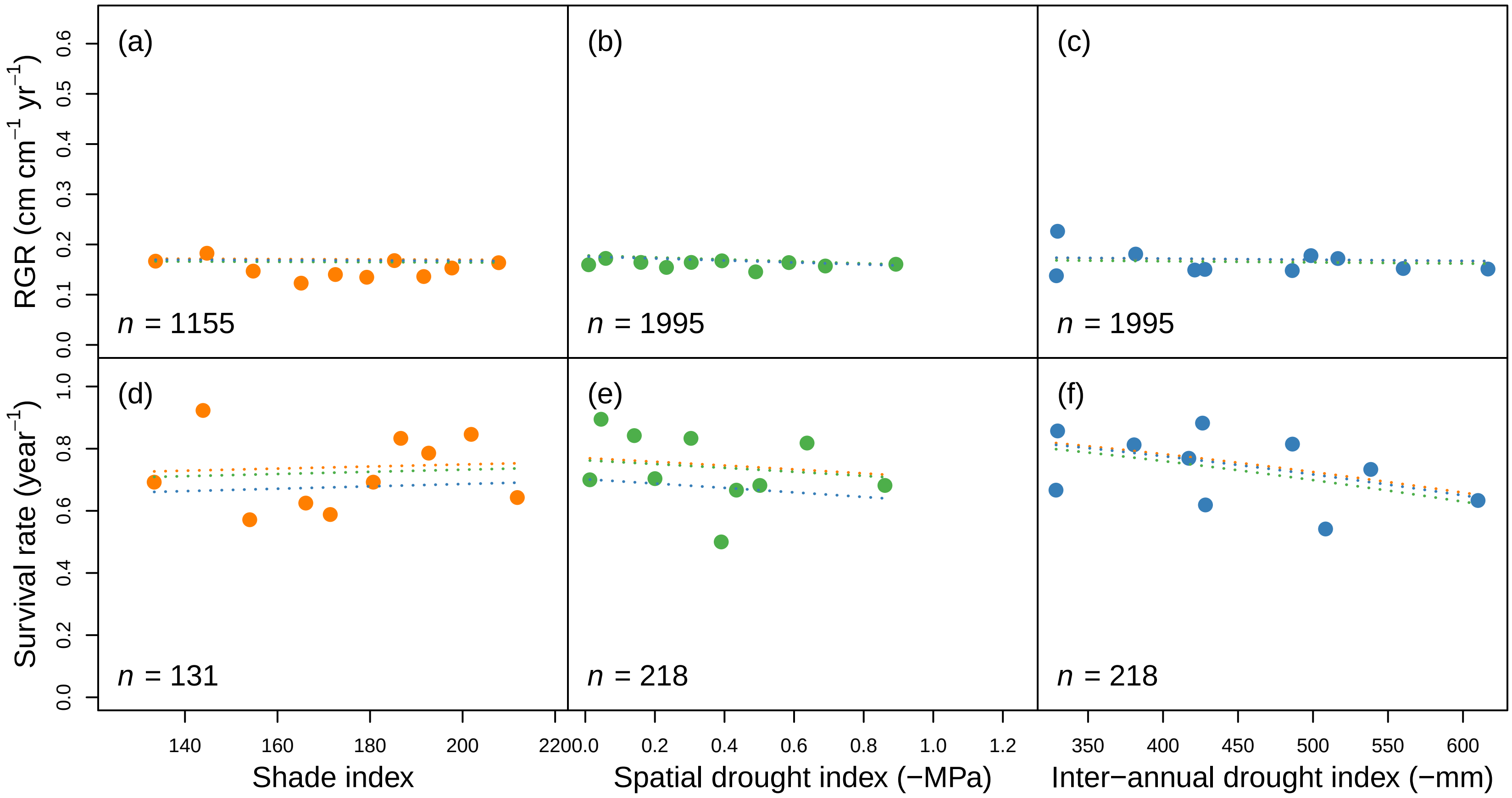

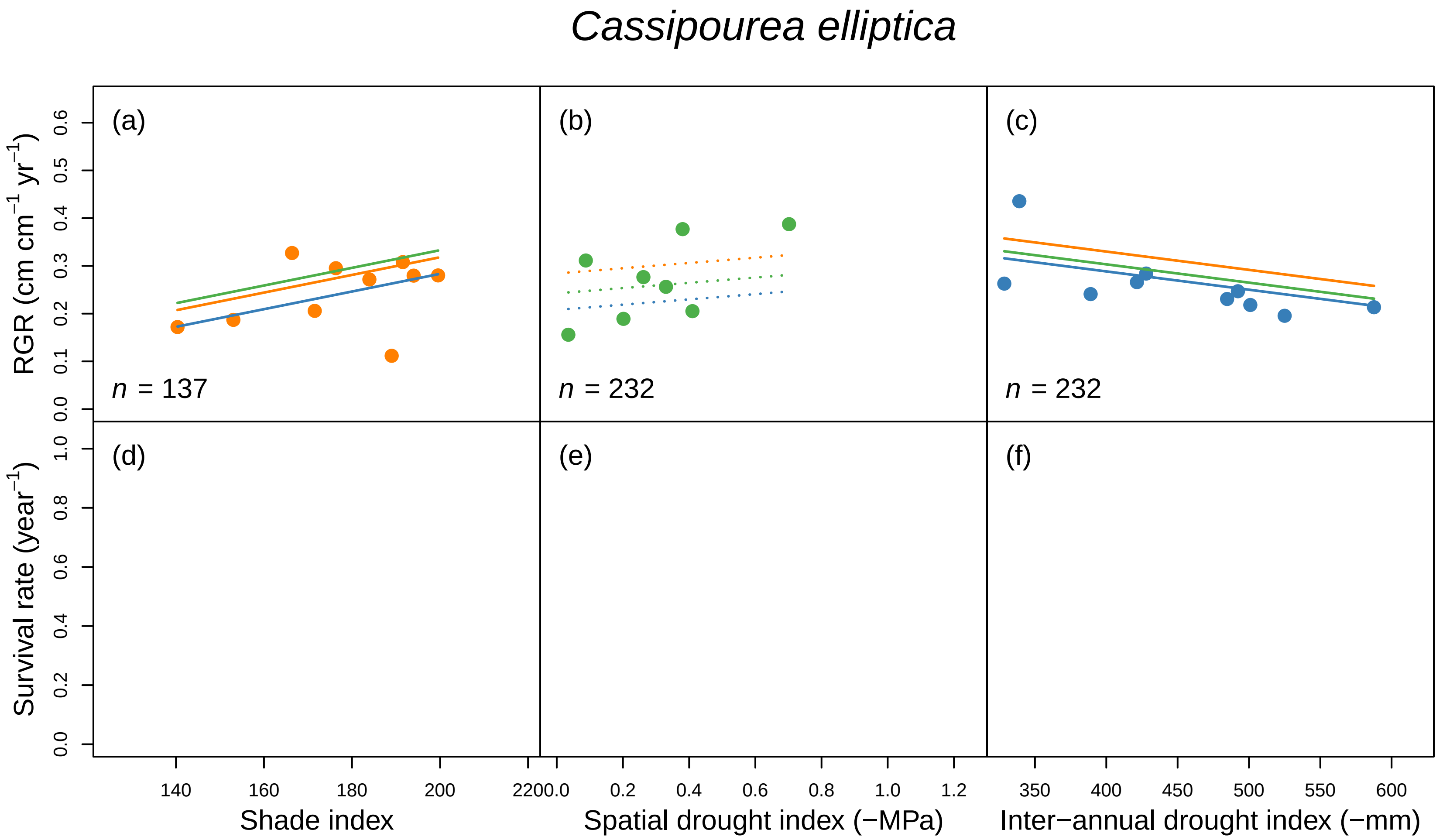

*Cassipourea elliptica*
(a)
(b)
(c)
(d)
(e)
(f)
*n* = 137
*n* = 232
*n* = 232
RGR (cm cm$^{-1}$ yr$^{-1}$)
Survival rate (year$^{-1}$)
0.0
0.1
0.2
0.3
0.4
0.5
0.6
0.0
0.2
0.4
0.6
0.8
1.0
140
160
180
200
220
0.0
0.2
0.4
0.6
0.8
1.0
1.2
350
400
450
500
550
600
Shade index
Spatial drought index (−MPa)
Inter−annual drought index (−mm)

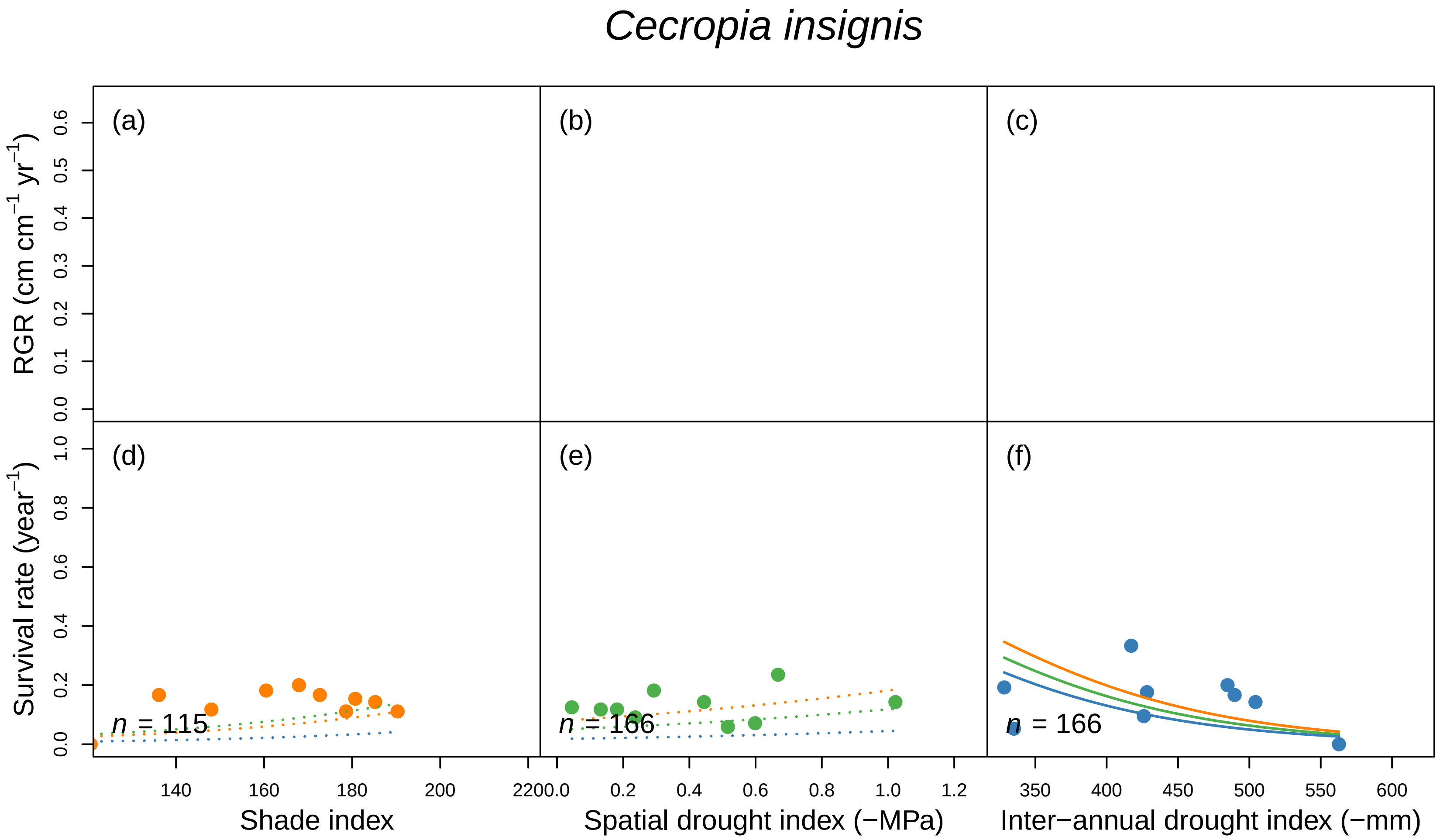

Cecropia insignis
RGR (cm cm$^{-1}$ yr$^{-1}$)
0.0
0.1
0.2
0.3
0.4
0.5
0.6
(a)
(b)
(c)
Survival rate (year$^{-1}$)
0.0
0.2
0.4
0.6
0.8
1.0
(d)
(e)
(f)
n = 115
n = 166
n = 166
140
160
180
200
220
0.0
0.2
0.4
0.6
0.8
1.0
1.2
350
400
450
500
550
600
Shade index
Spatial drought index (−MPa)
Inter−annual drought index (−mm)

# *Chrysophyllum argenteum*

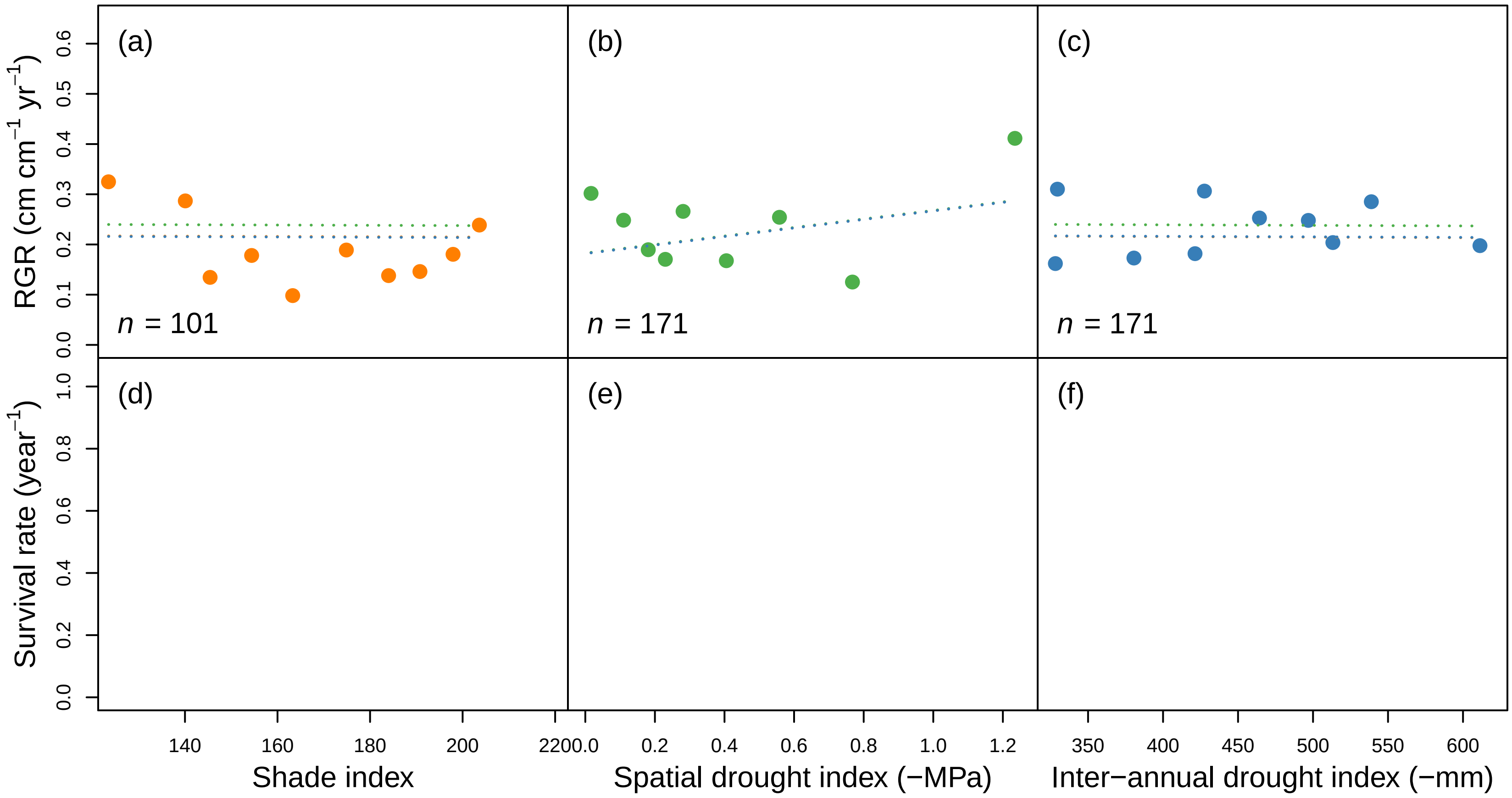

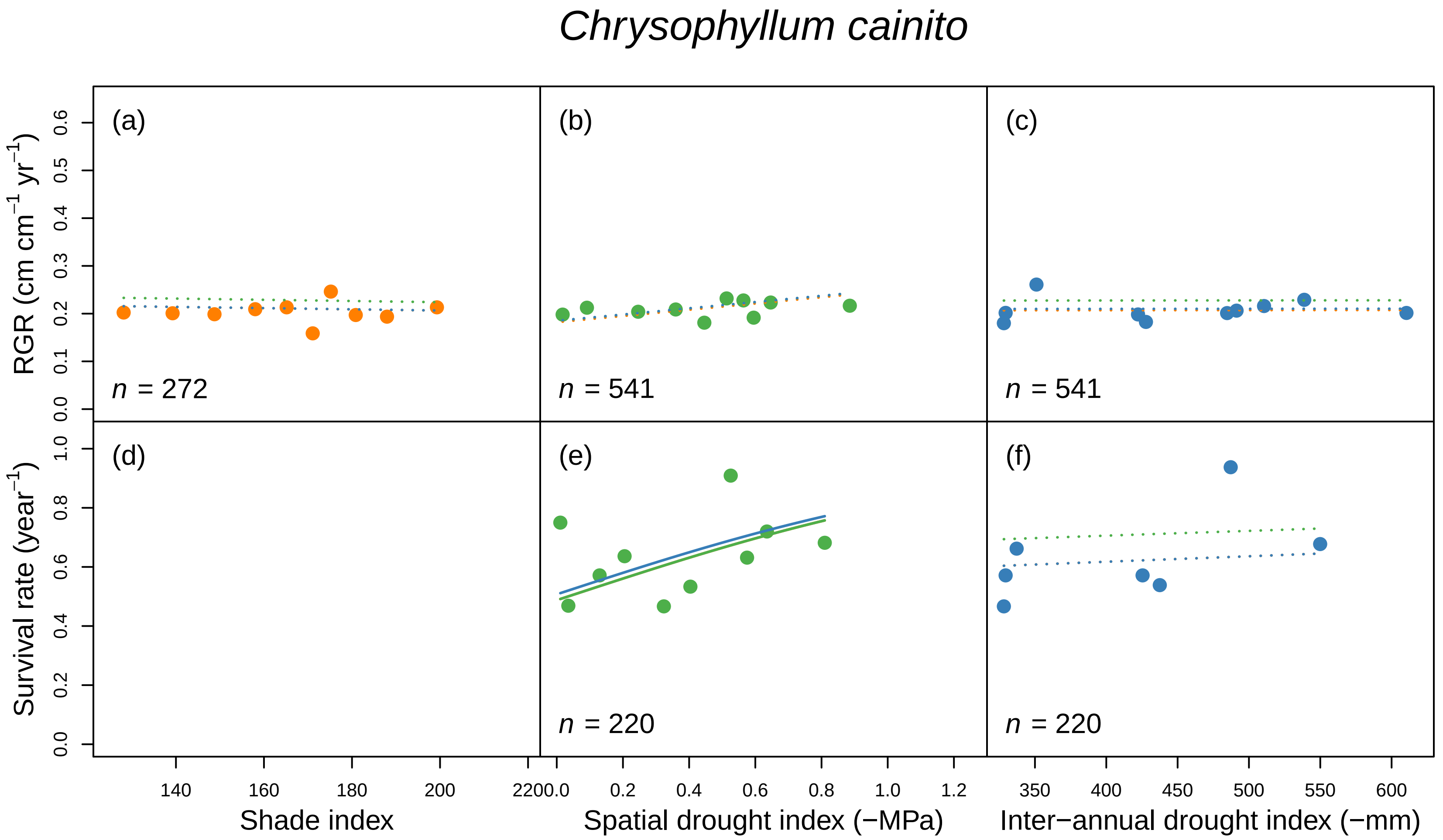

Chrysophyllum cainito
(a)
(b)
(c)
(d)
(e)
(f)
n = 272
n = 541
n = 541
n = 220
n = 220
RGR (cm cm−1 yr−1)
Survival rate (year−1)
0.0
0.1
0.2
0.3
0.4
0.5
0.6
0.0
0.2
0.4
0.6
0.8
1.0
140
160
180
200
220
0.0
0.2
0.4
0.6
0.8
1.0
1.2
350
400
450
500
550
600
Shade index
Spatial drought index (−MPa)
Inter−annual drought index (−mm)

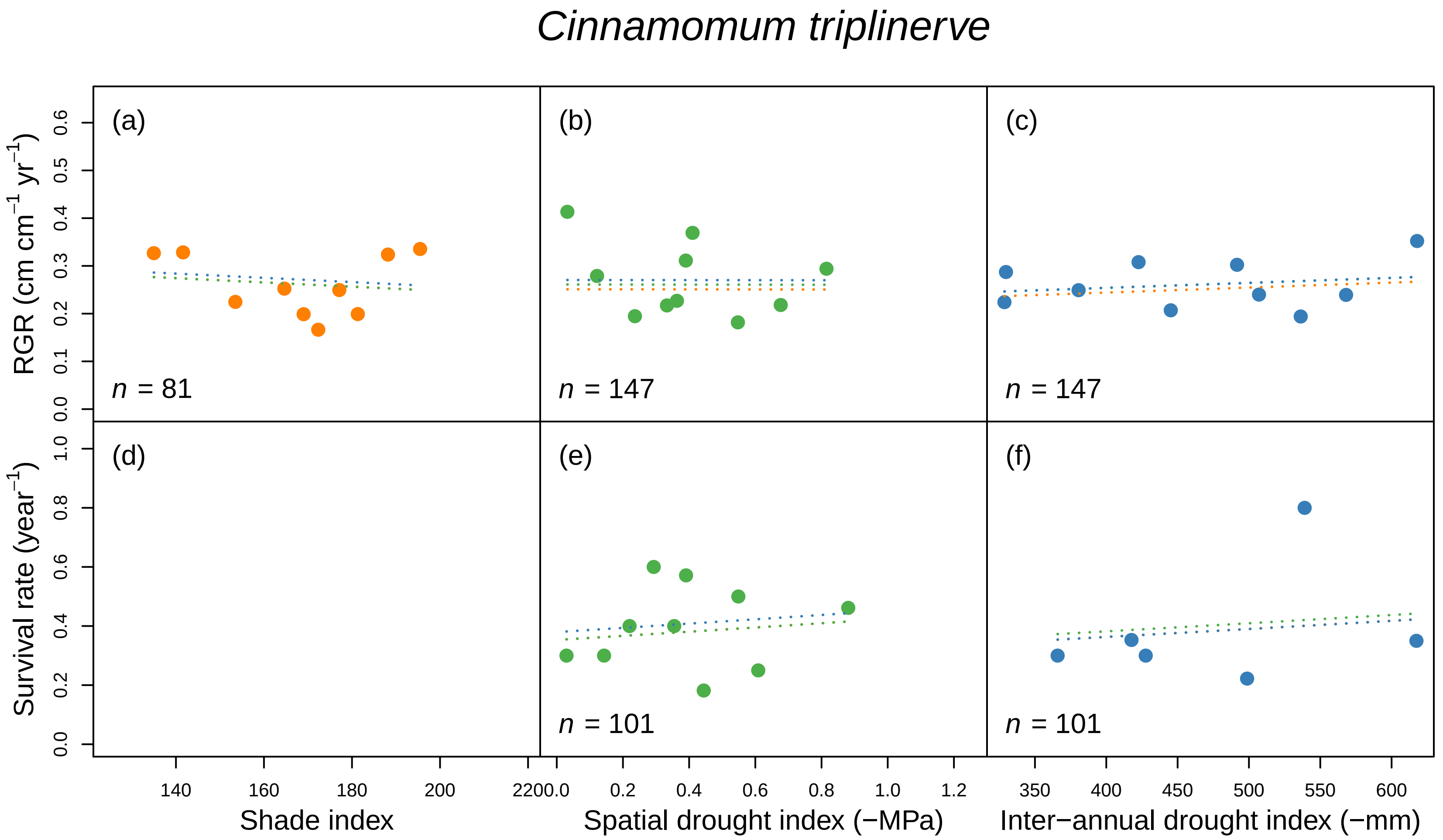

Cinnamomum triplinerve
(a)
(b)
(c)
(d)
(e)
(f)
n = 81
n = 147
n = 147
n = 101
n = 101
RGR (cm cm−1 yr−1)
Survival rate (year−1)
0.0
0.1
0.2
0.3
0.4
0.5
0.6
0.0
0.2
0.4
0.6
0.8
1.0
140
160
180
200
220
0.0
0.2
0.4
0.6
0.8
1.0
1.2
350
400
450
500
550
600
Shade index
Spatial drought index (−MPa)
Inter−annual drought index (−mm)

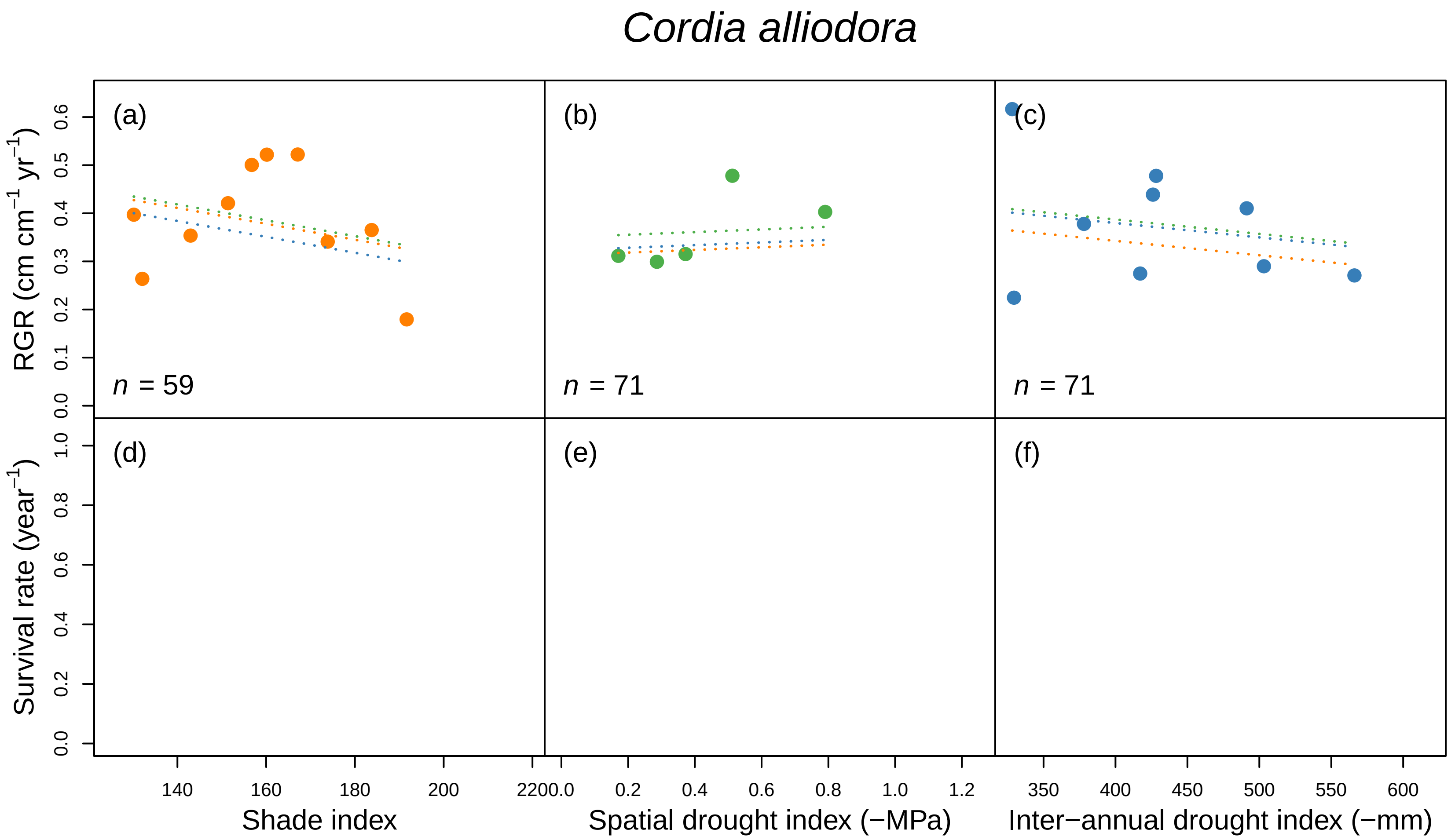

Cordia alliodora
(a)
(b)
(c)
(d)
(e)
(f)
n = 59
n = 71
n = 71
RGR (cm cm−1 yr−1)
Survival rate (year−1)
0.0
0.1
0.2
0.3
0.4
0.5
0.6
0.0
0.2
0.4
0.6
0.8
1.0
140
160
180
200
220
0.0
0.2
0.4
0.6
0.8
1.0
1.2
350
400
450
500
550
600
Shade index
Spatial drought index (−MPa)
Inter−annual drought index (−mm)

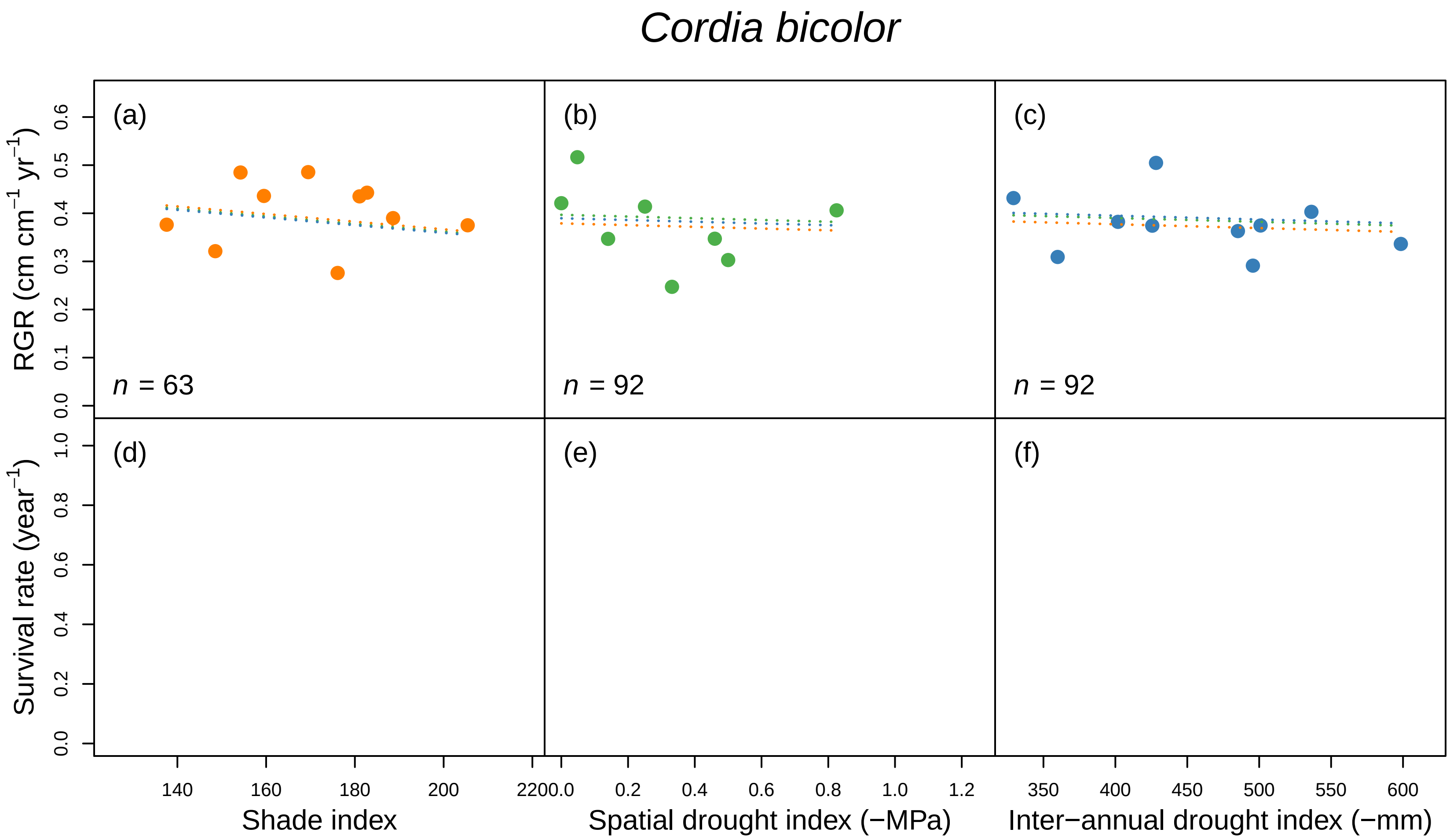
Cordia bicolor
(a)
(b)
(c)
(d)
(e)
(f)
n = 63
n = 92
n = 92
RGR (cm cm−1 yr−1)
Survival rate (year−1)
0.0
0.1
0.2
0.3
0.4
0.5
0.6
0.0
0.2
0.4
0.6
0.8
1.0
140
160
180
200
220
0.0
0.2
0.4
0.6
0.8
1.0
1.2
350
400
450
500
550
600
Shade index
Spatial drought index (−MPa)
Inter−annual drought index (−mm)

# *Cordia lasiocalyx*

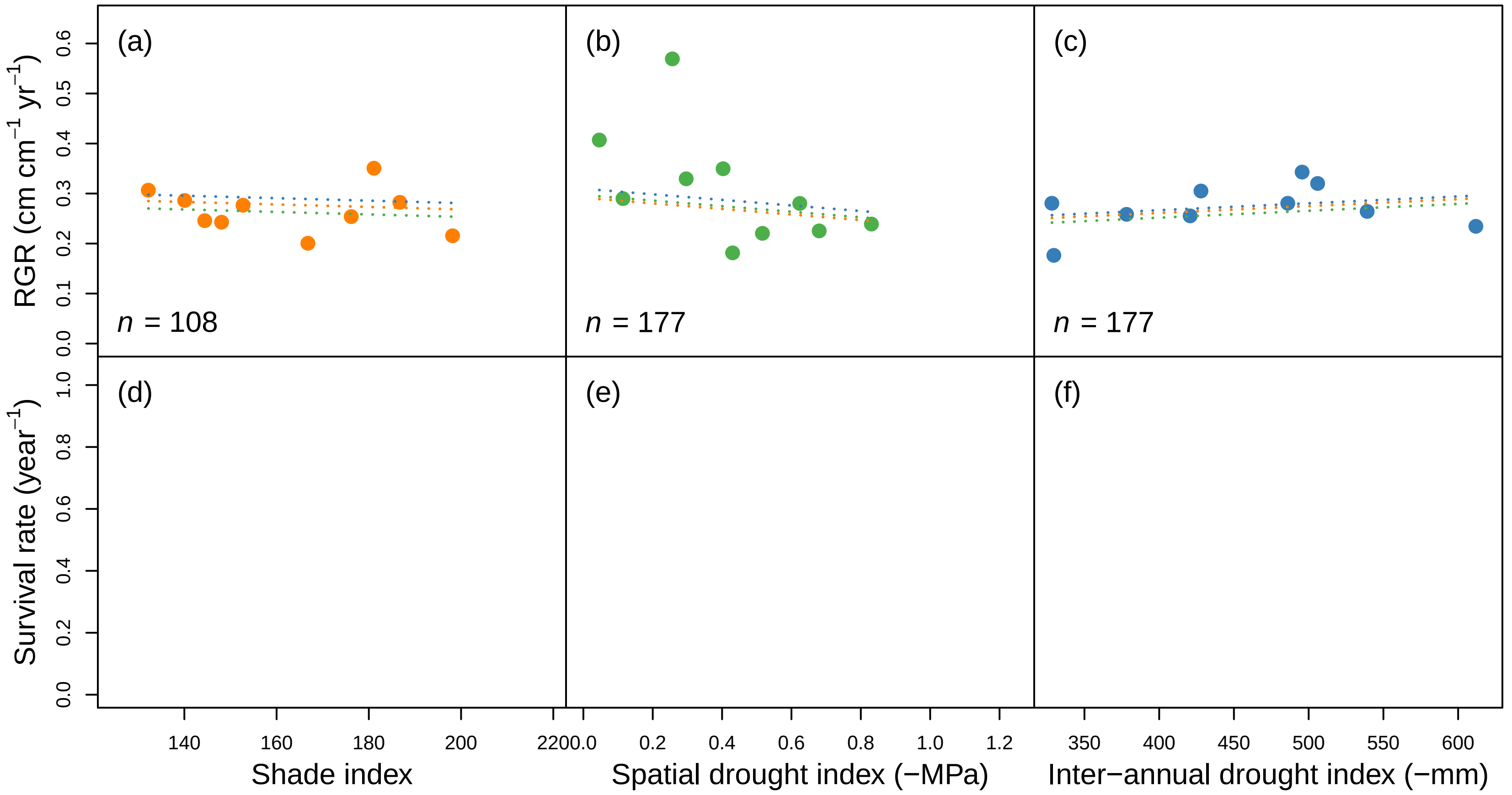

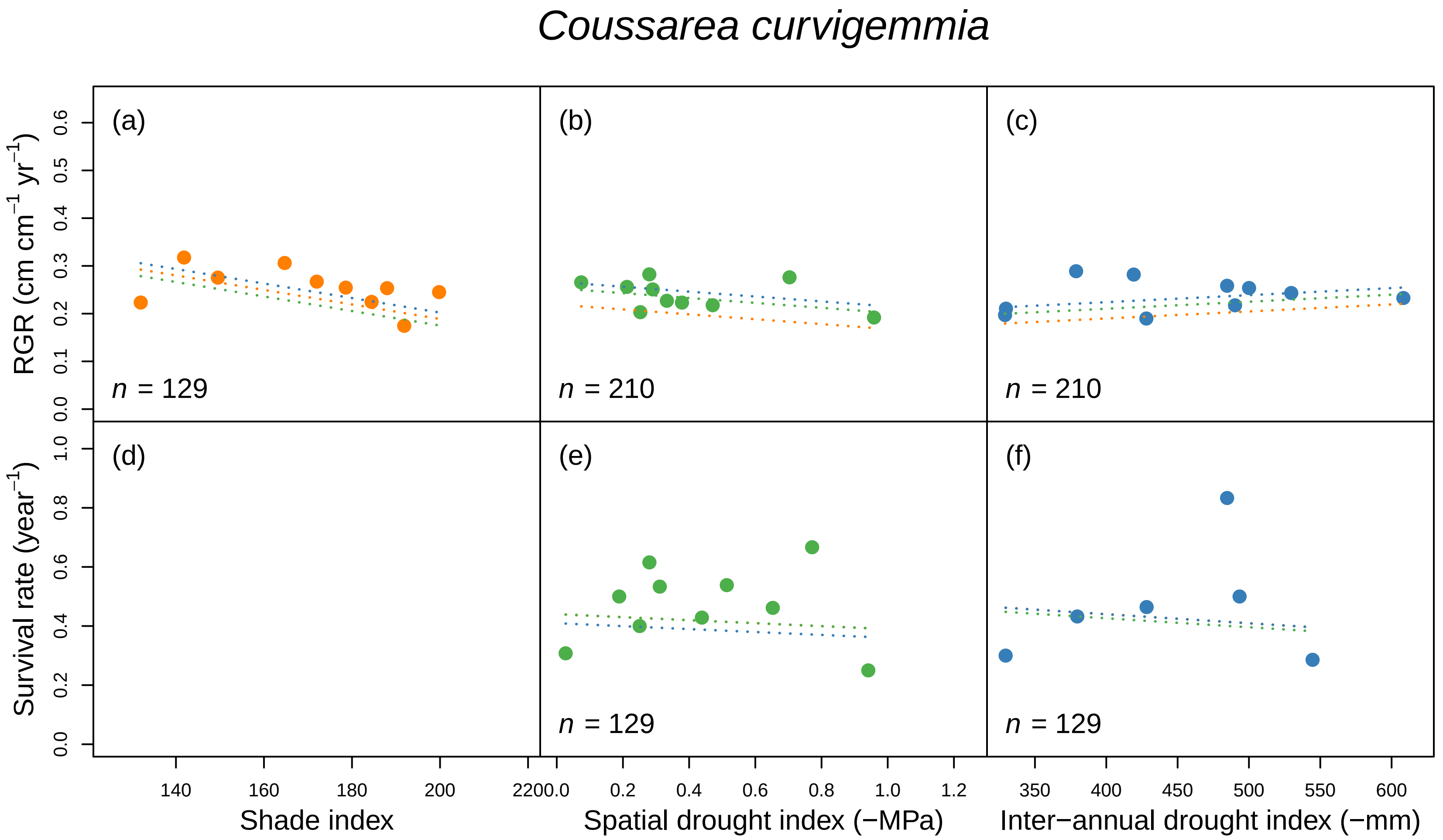

Coussarea curvigemmia
(a)
(b)
(c)
(d)
(e)
(f)
n = 129
n = 210
n = 210
n = 129
n = 129
RGR (cm cm−1 yr−1)
Survival rate (year−1)
Shade index
Spatial drought index (−MPa)
Inter−annual drought index (−mm)

# *Croton billbergianus*

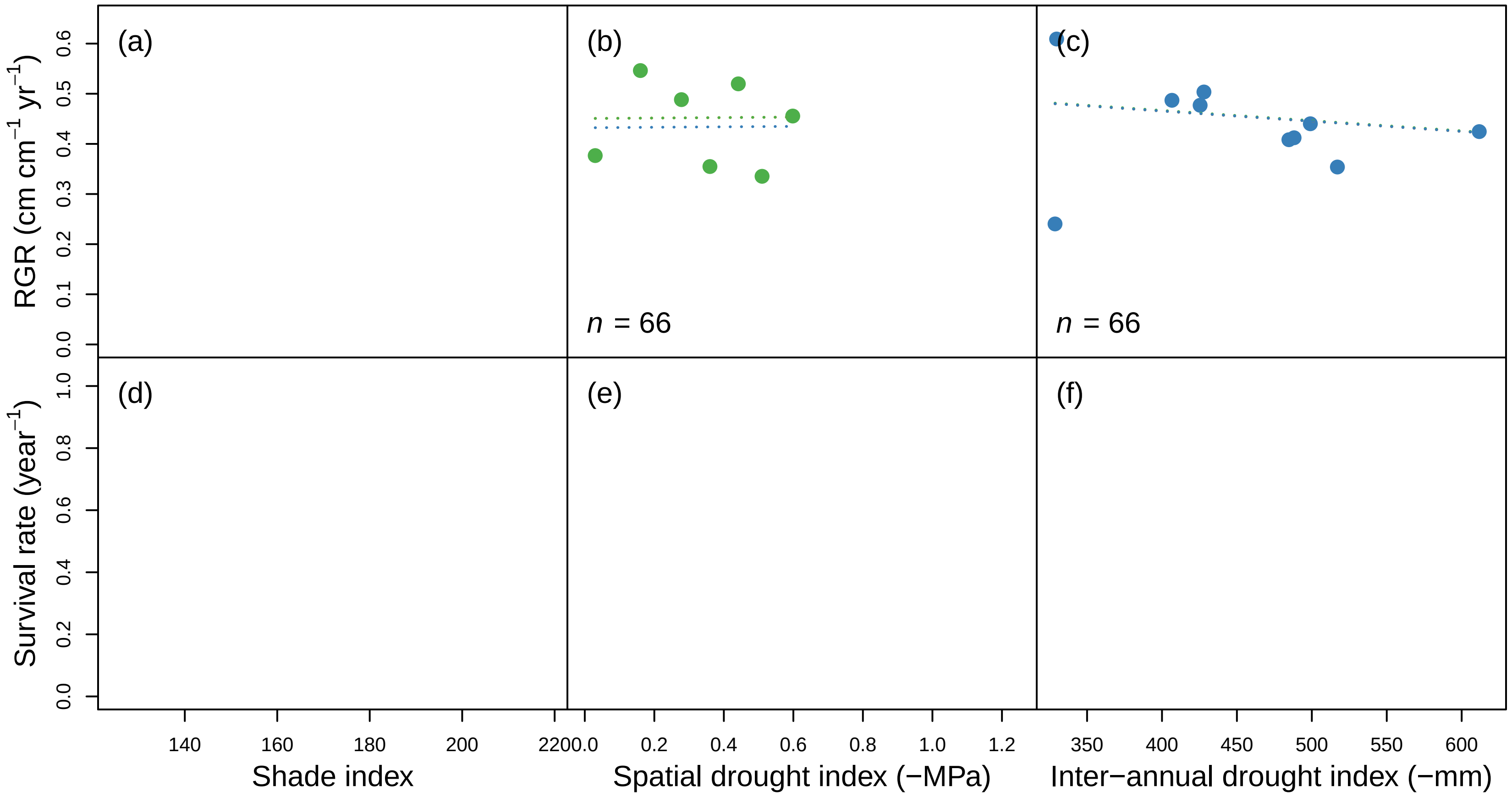

# *Cupania rufescens*

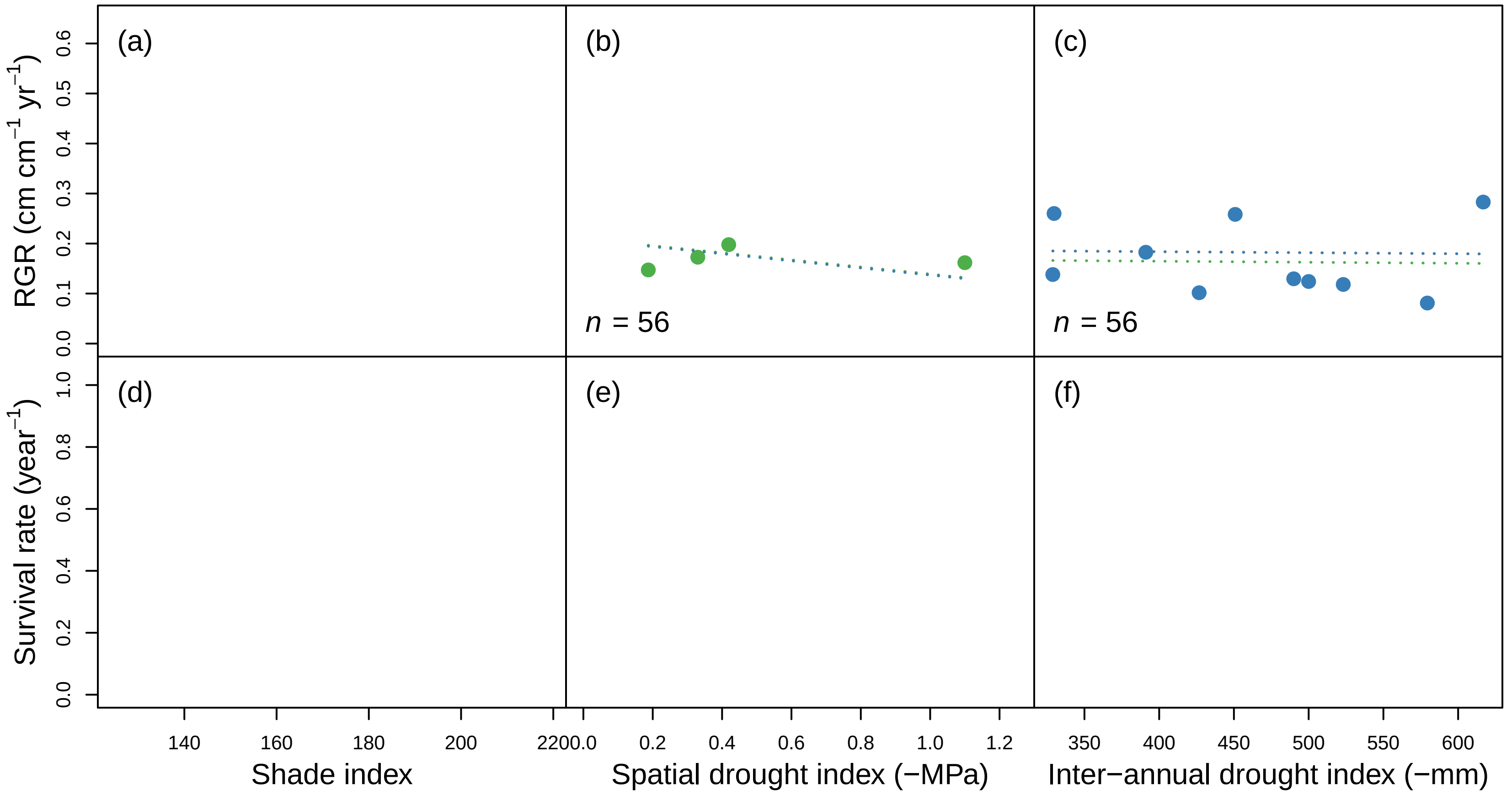

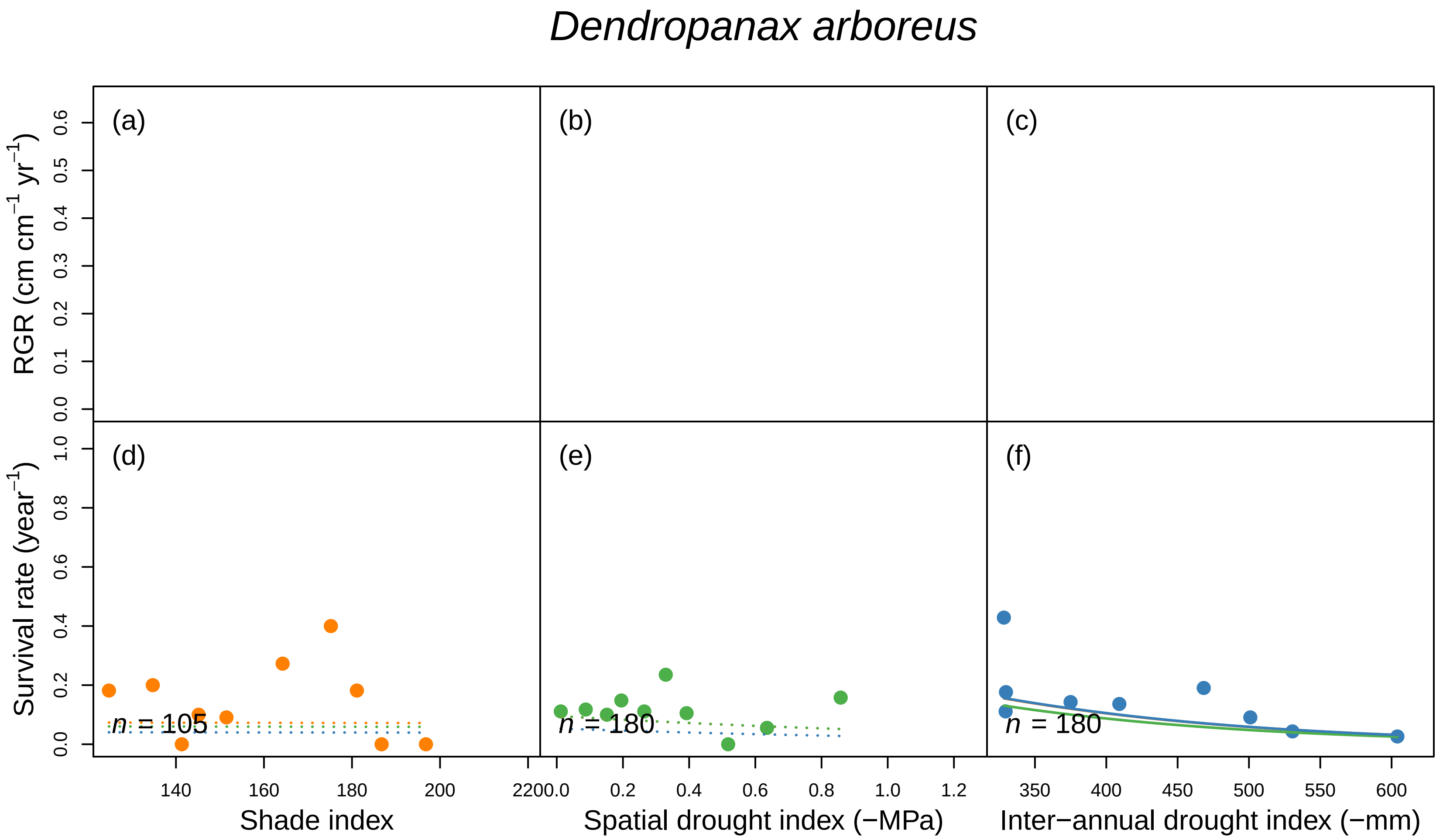
Dendropanax arboreus
(a)
(b)
(c)
(d)
(e)
(f)
RGR (cm cm−1 yr−1)
Survival rate (year−1)
0.0
0.1
0.2
0.3
0.4
0.5
0.6
0.0
0.2
0.4
0.6
0.8
1.0
n = 105
n = 180
n = 180
140
160
180
200
220
0.0
0.2
0.4
0.6
0.8
1.0
1.2
350
400
450
500
550
600
Shade index
Spatial drought index (−MPa)
Inter−annual drought index (−mm)

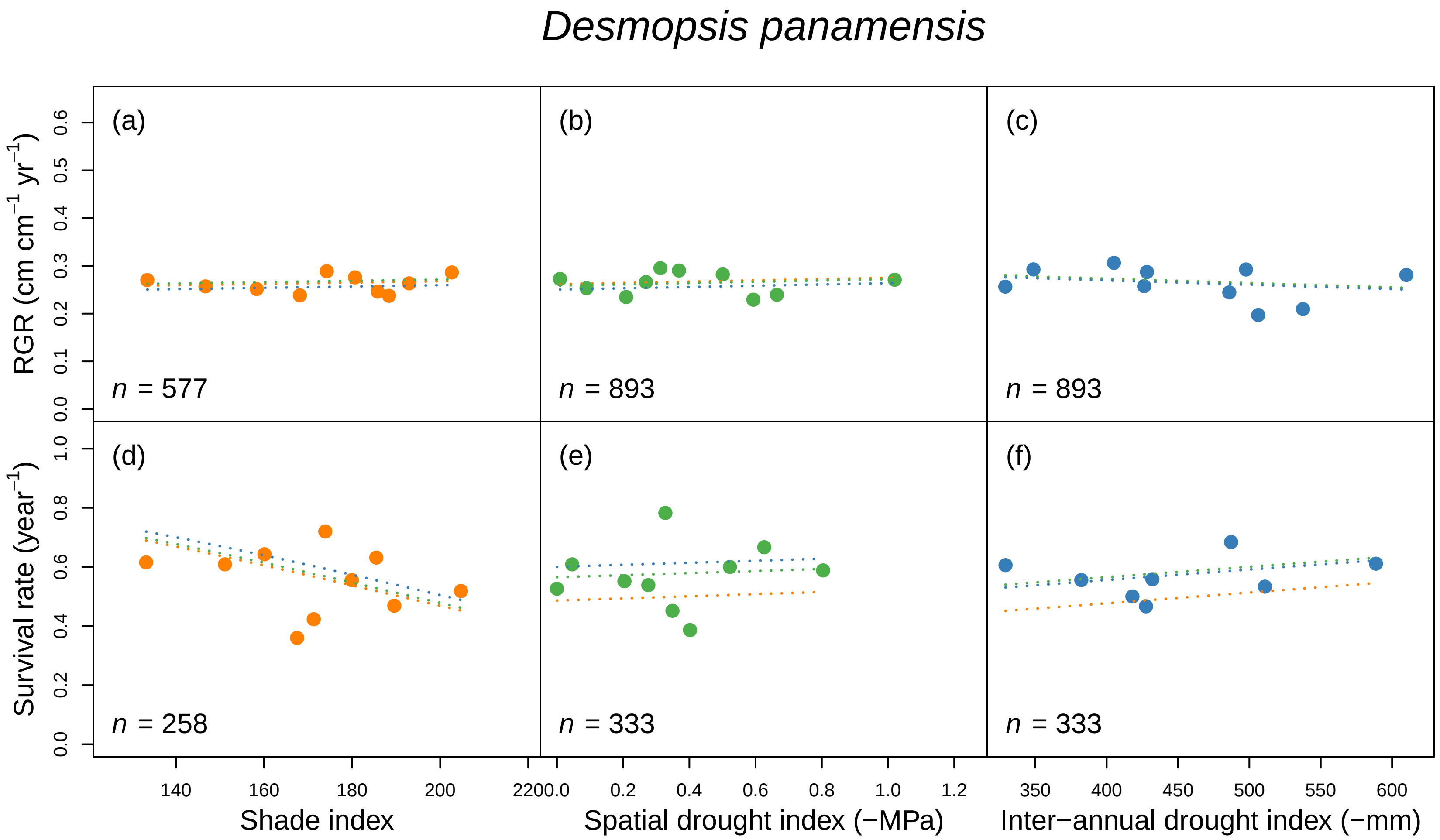
Desmopsis panamensis
(a)
(b)
(c)
(d)
(e)
(f)
n = 577
n = 893
n = 893
n = 258
n = 333
n = 333
RGR (cm cm−1 yr−1)
Survival rate (year−1)
0.0
0.1
0.2
0.3
0.4
0.5
0.6
0.0
0.2
0.4
0.6
0.8
1.0
140
160
180
200
220
0.0
0.2
0.4
0.6
0.8
1.0
1.2
350
400
450
500
550
600
Shade index
Spatial drought index (−MPa)
Inter−annual drought index (−mm)

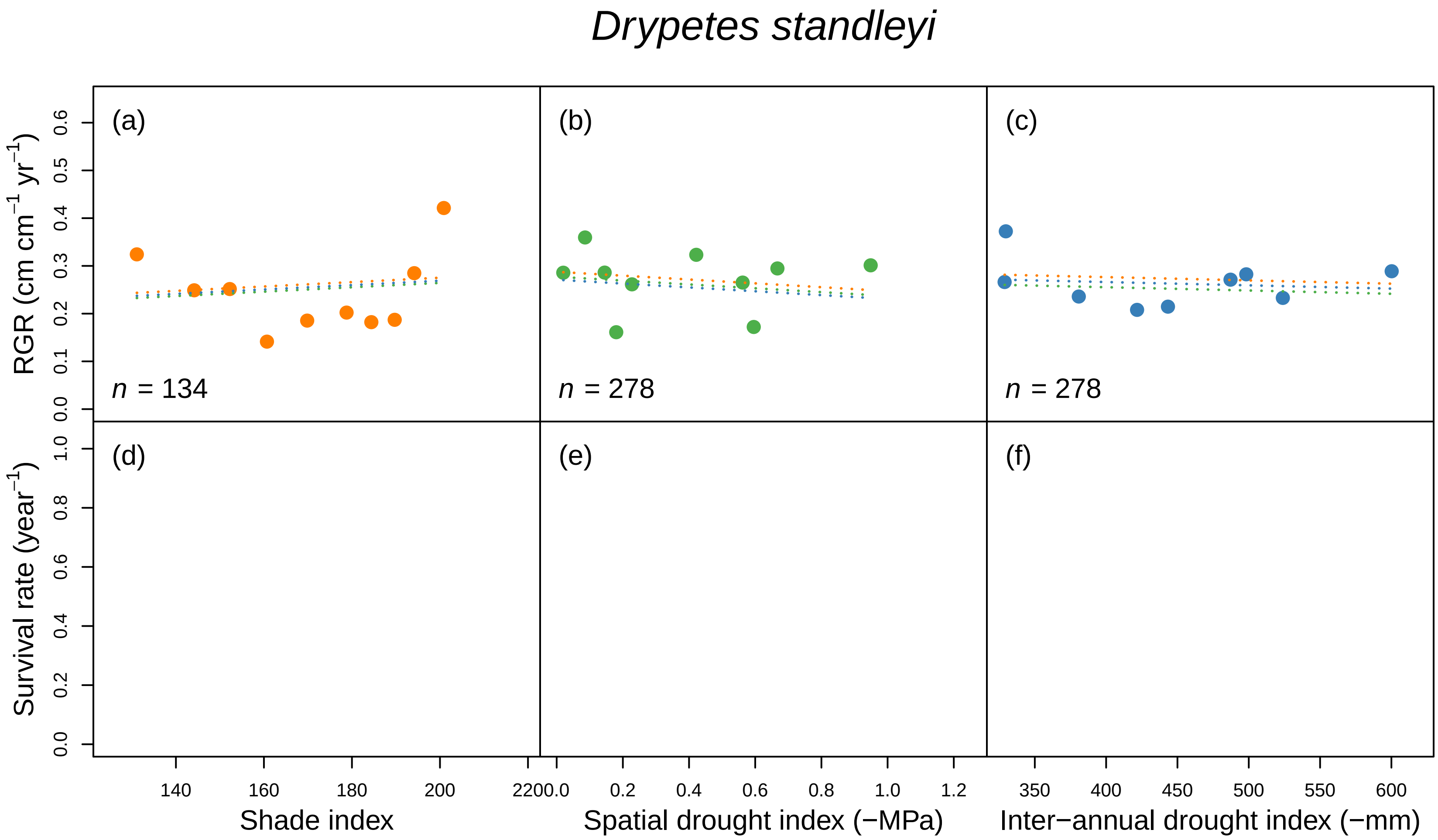

Drypetes standleyi
(a)
(b)
(c)
(d)
(e)
(f)
n = 134
n = 278
n = 278
RGR (cm cm−1 yr−1)
Survival rate (year−1)
0.0
0.1
0.2
0.3
0.4
0.5
0.6
0.0
0.2
0.4
0.6
0.8
1.0
140
160
180
200
220
0.0
0.2
0.4
0.6
0.8
1.0
1.2
350
400
450
500
550
600
Shade index
Spatial drought index (−MPa)
Inter−annual drought index (−mm)

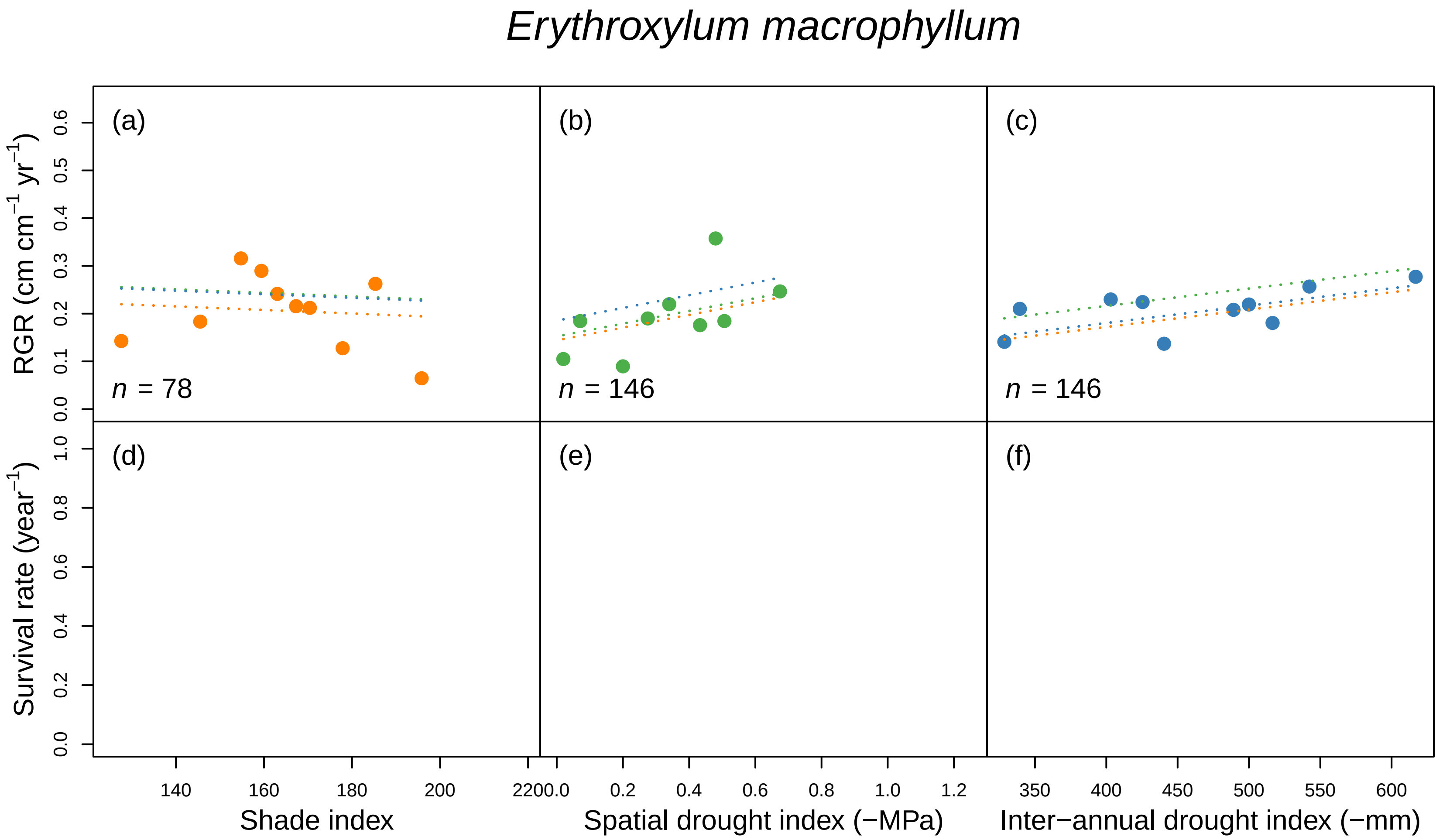
Erythroxylum macrophyllum
(a)
(b)
(c)
(d)
(e)
(f)
n = 78
n = 146
n = 146
RGR (cm cm−1 yr−1)
Survival rate (year−1)
Shade index
Spatial drought index (−MPa)
Inter−annual drought index (−mm)

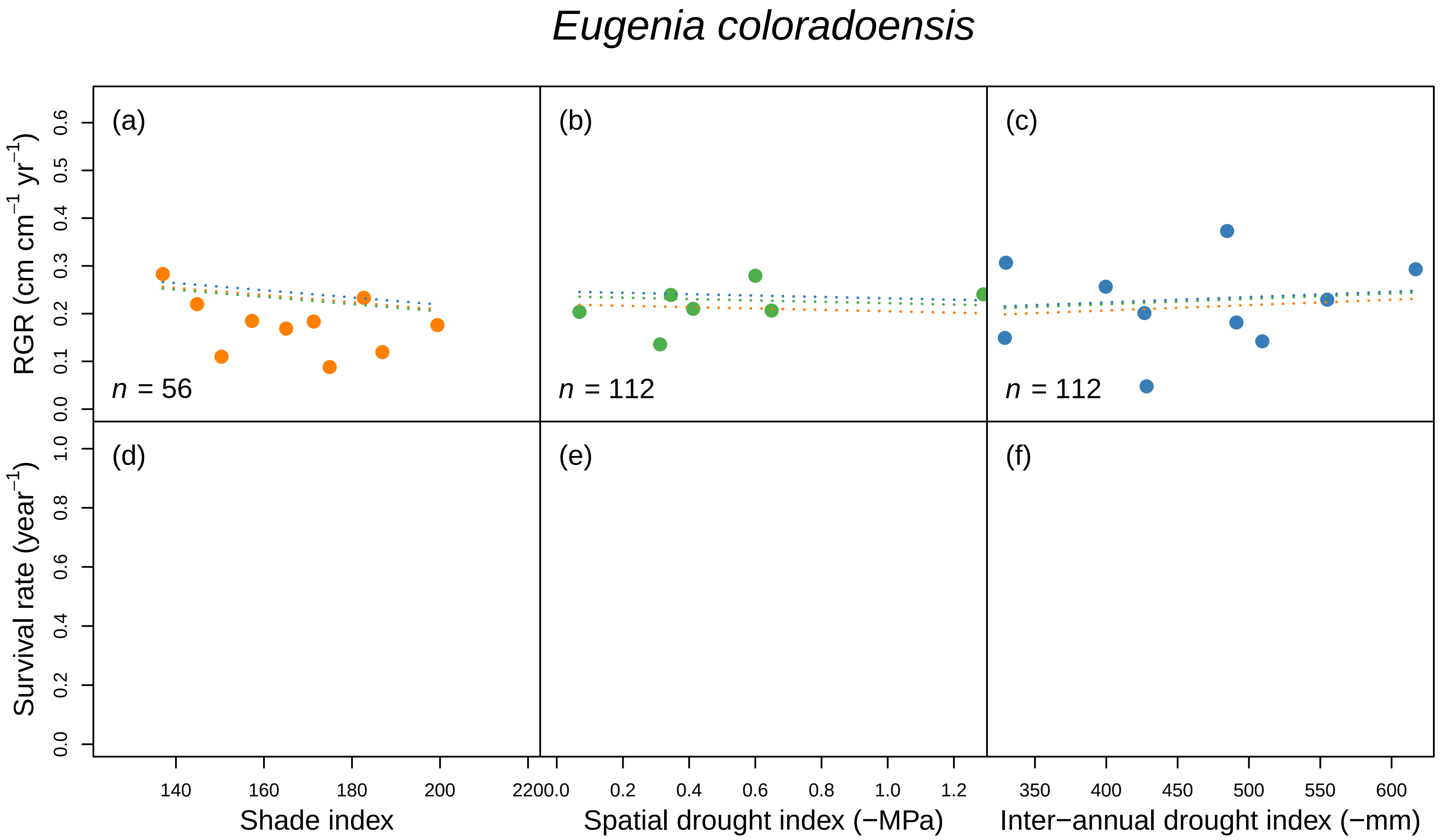

Eugenia coloradoensis
RGR (cm cm$^{-1}$ yr$^{-1}$)
Survival rate (year$^{-1}$)
(a)
(b)
(c)
(d)
(e)
(f)
n = 56
n = 112
n = 112
0.0
0.1
0.2
0.3
0.4
0.5
0.6
0.0
0.2
0.4
0.6
0.8
1.0
140
160
180
200
220
0.0
0.2
0.4
0.6
0.8
1.0
1.2
350
400
450
500
550
600
Shade index
Spatial drought index (−MPa)
Inter−annual drought index (−mm)

## *Eugenia galalonensis*

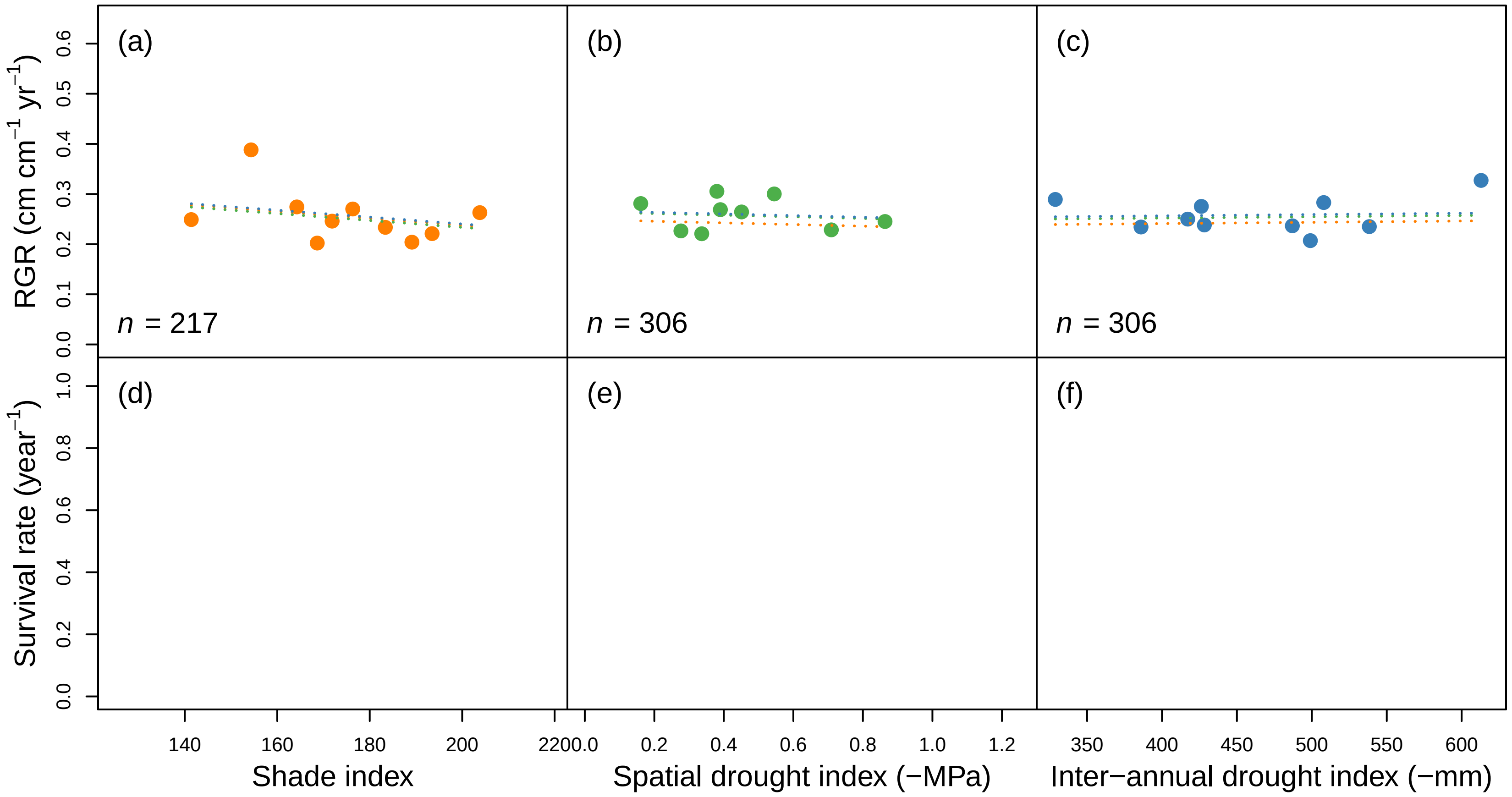

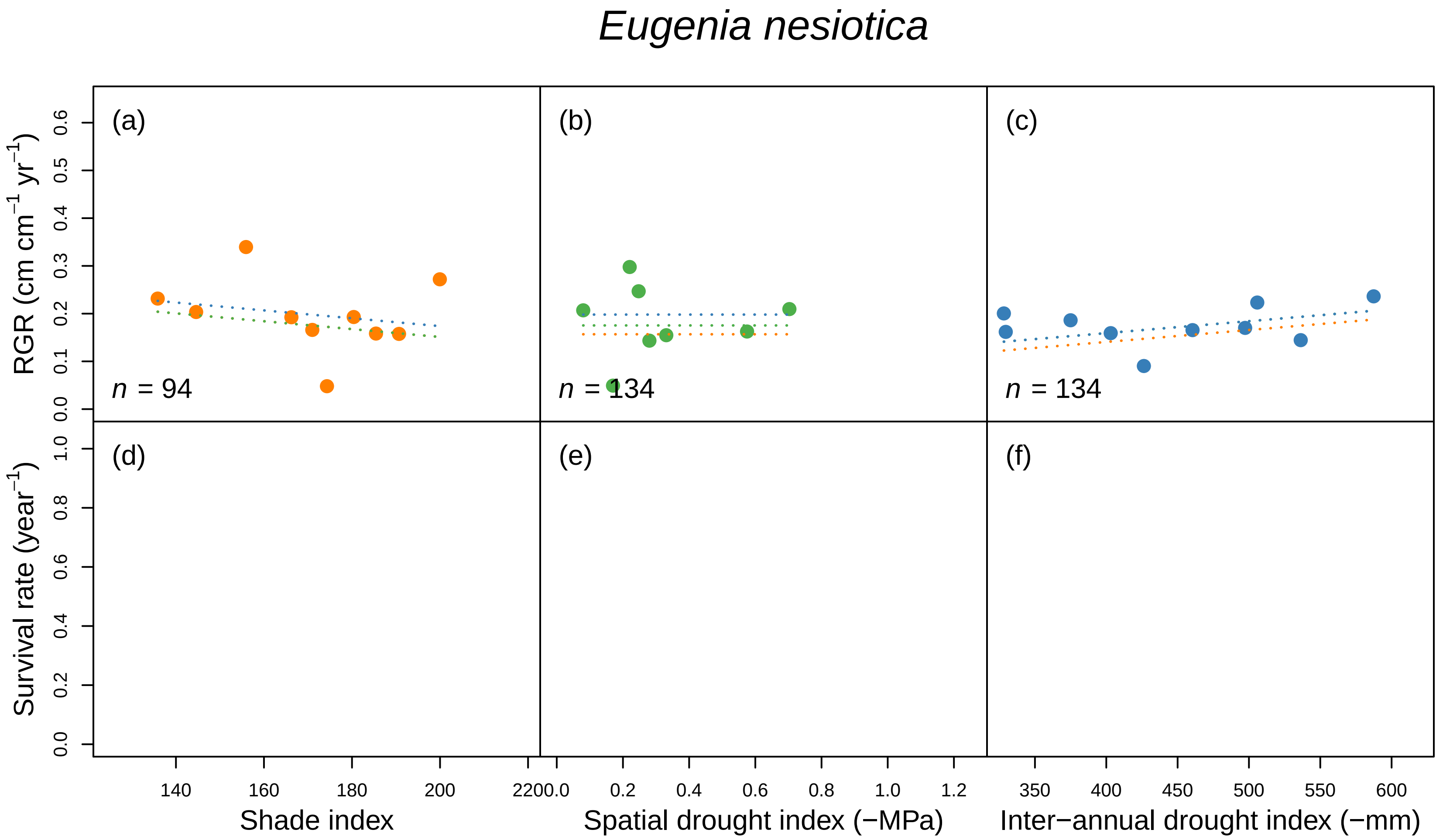

Eugenia nesiotica
(a)
(b)
(c)
(d)
(e)
(f)
n = 94
n = 134
n = 134
RGR (cm cm−1 yr−1)
Survival rate (year−1)
Shade index
Spatial drought index (−MPa)
Inter−annual drought index (−mm)

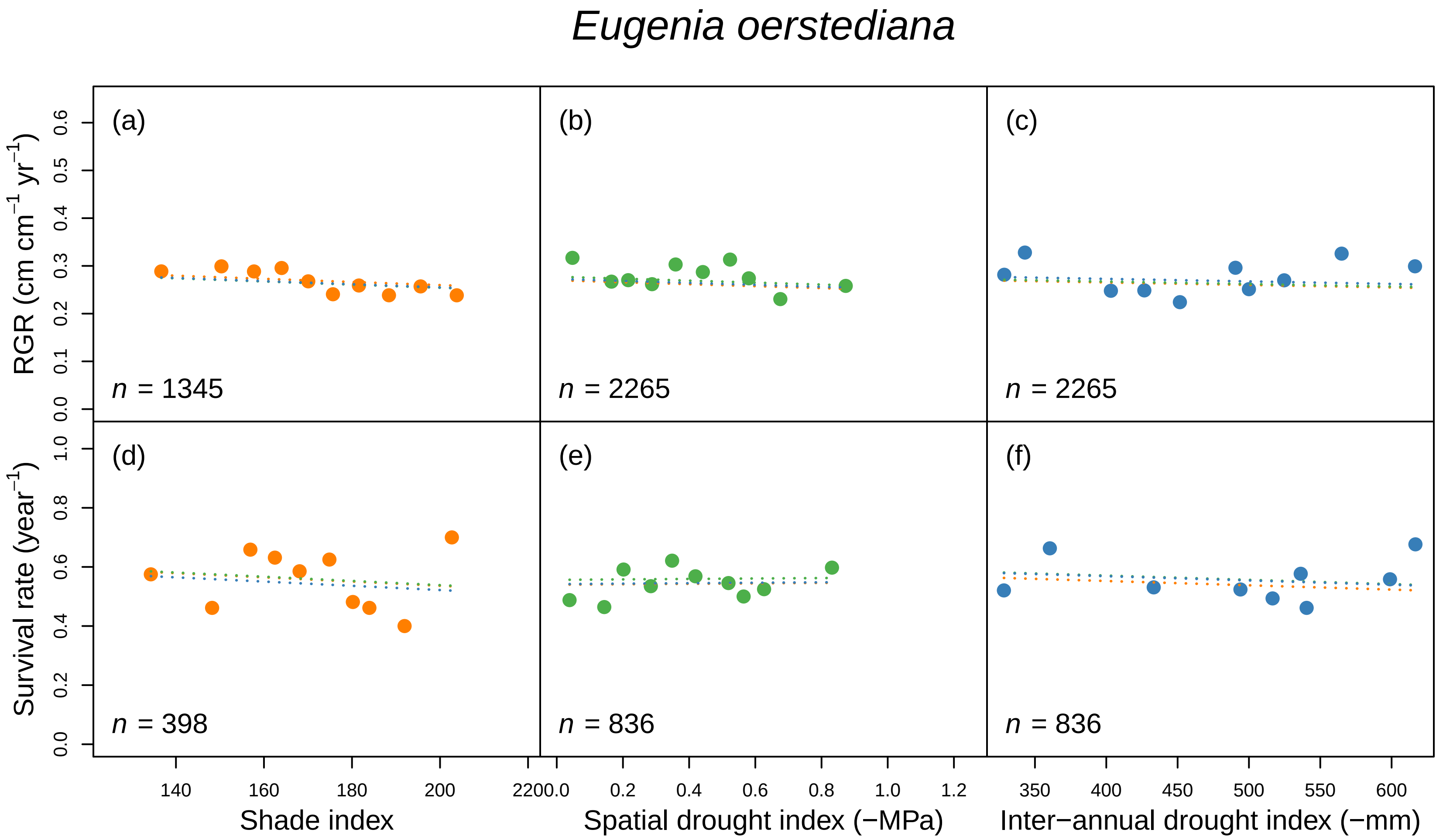
Eugenia oerstediana
RGR (cm cm−1 yr−1)
Survival rate (year−1)
(a)
(b)
(c)
(d)
(e)
(f)
n = 1345
n = 2265
n = 2265
n = 398
n = 836
n = 836
Shade index
Spatial drought index (−MPa)
Inter−annual drought index (−mm)

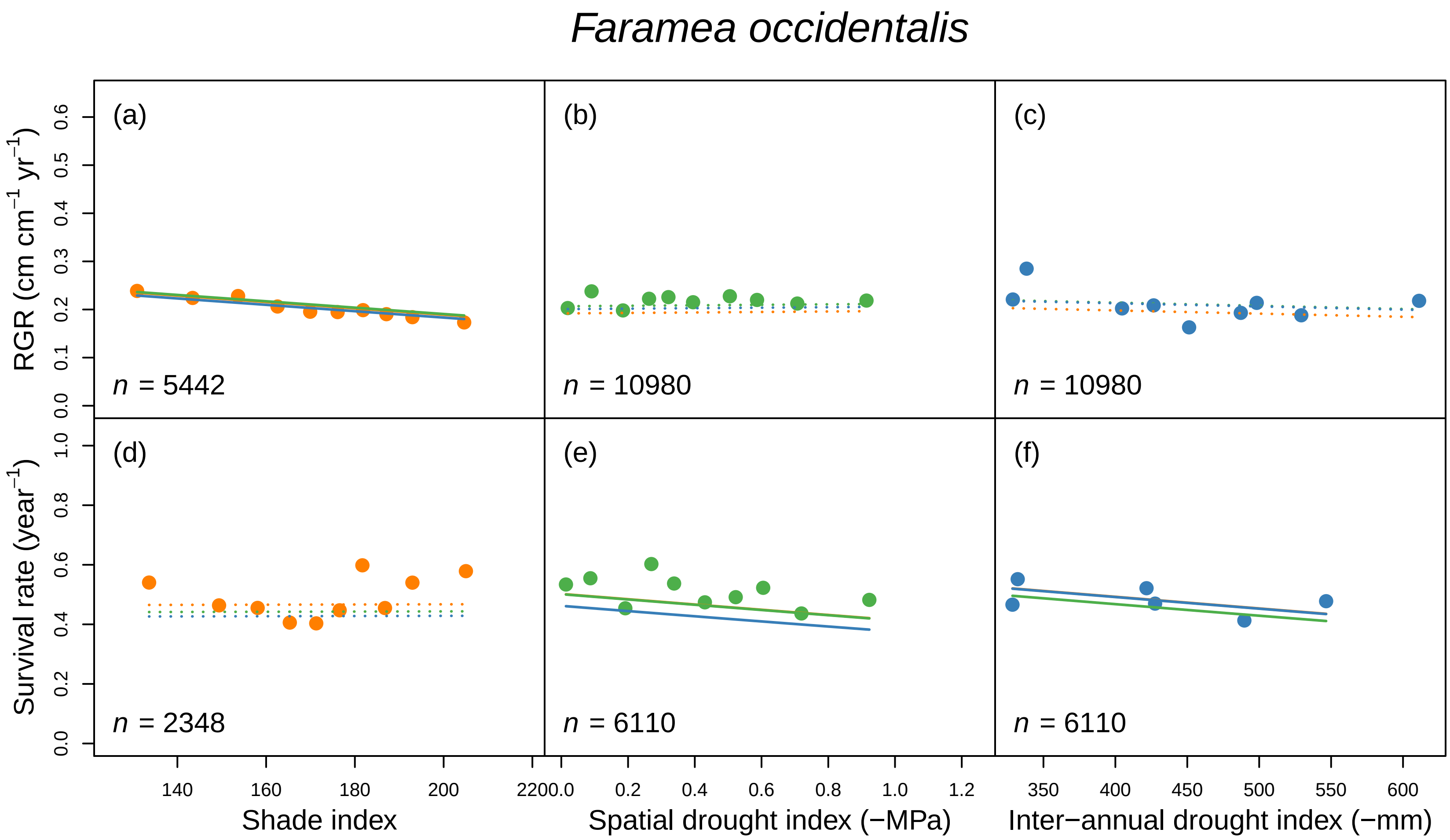
Faramea occidentalis
(a)
(b)
(c)
(d)
(e)
(f)
n = 5442
n = 10980
n = 10980
n = 2348
n = 6110
n = 6110
RGR (cm cm−1 yr−1)
Survival rate (year−1)
0.0
0.1
0.2
0.3
0.4
0.5
0.6
0.0
0.2
0.4
0.6
0.8
1.0
140
160
180
200
220
0.0
0.2
0.4
0.6
0.8
1.0
1.2
350
400
450
500
550
600
Shade index
Spatial drought index (−MPa)
Inter−annual drought index (−mm)

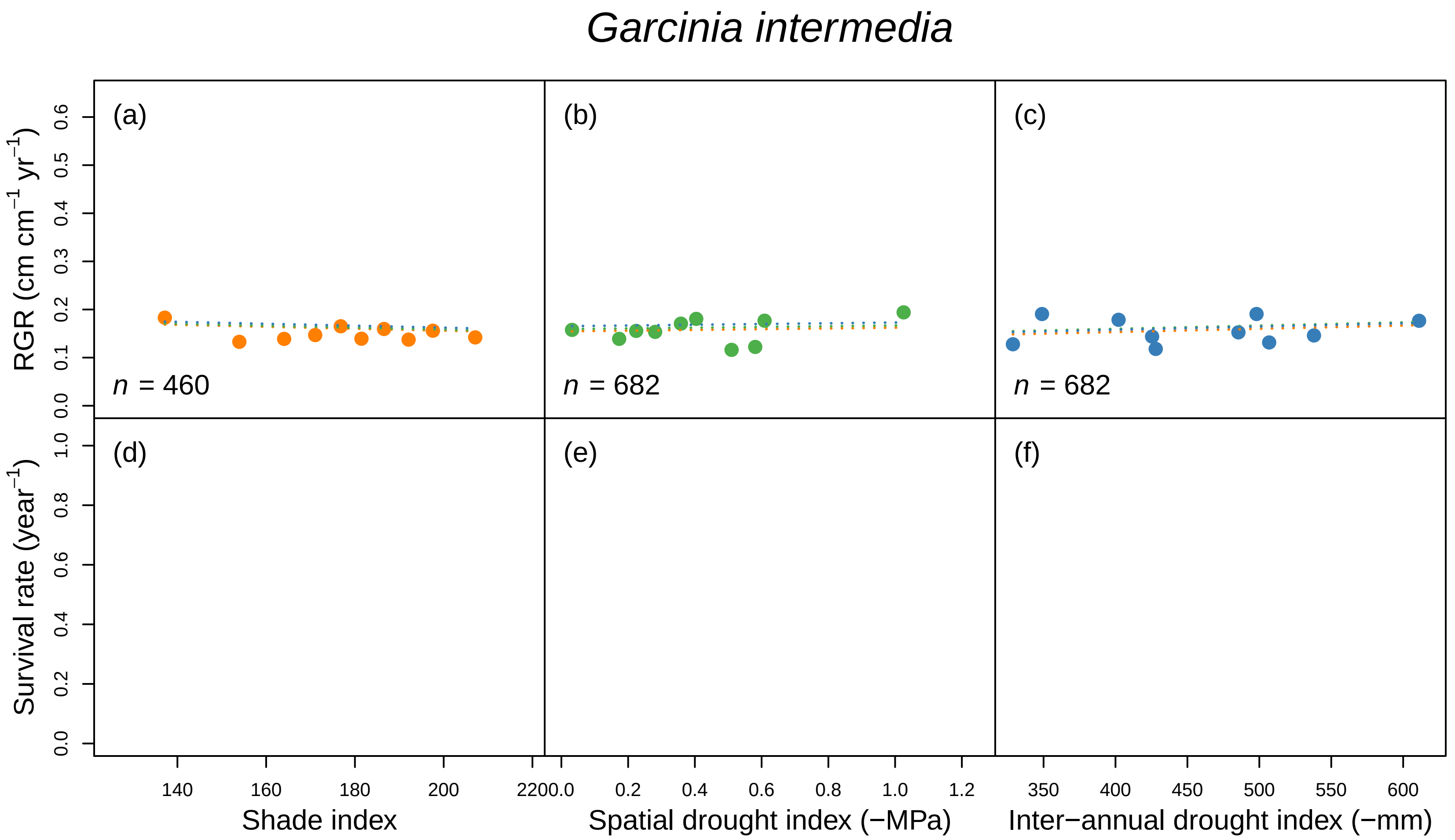
Garcinia intermedia
(a)
(b)
(c)
(d)
(e)
(f)
n = 460
n = 682
n = 682
RGR (cm cm−1 yr−1)
Survival rate (year−1)
Shade index
Spatial drought index (−MPa)
Inter−annual drought index (−mm)

# *Guapira standleyana*

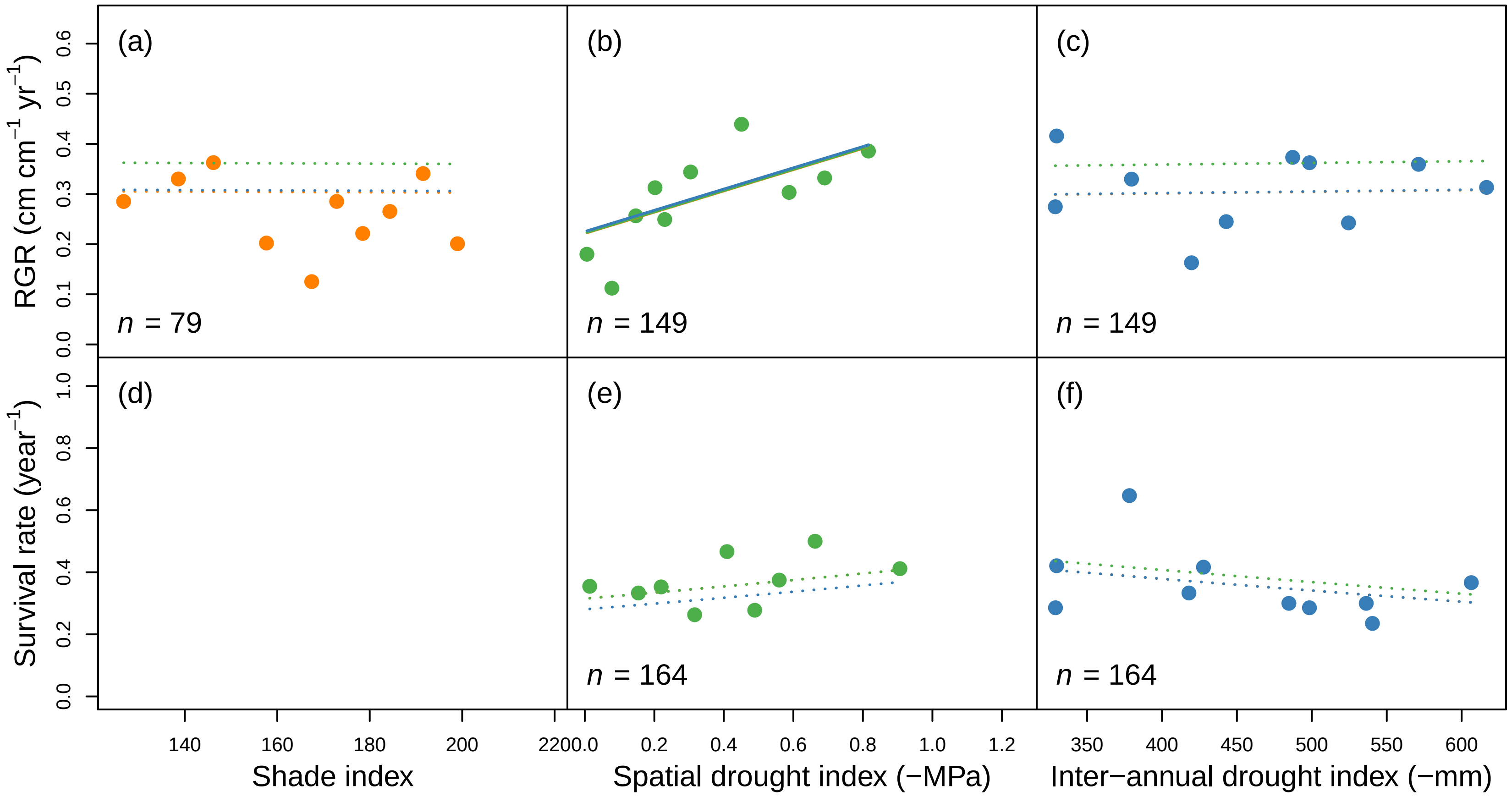

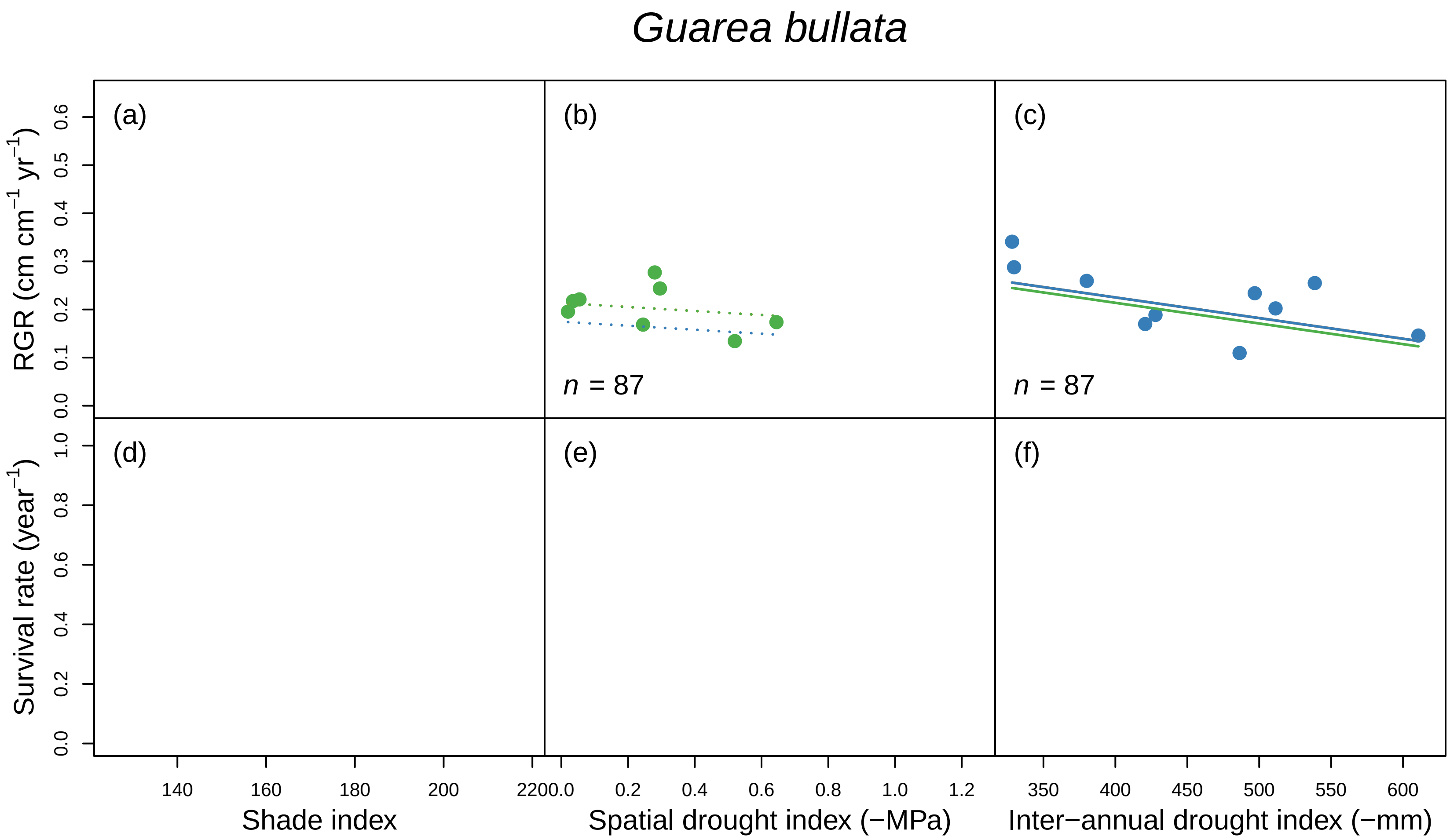

Guarea bullata
RGR (cm cm$^{-1}$ yr$^{-1}$)
Survival rate (year$^{-1}$)
(a)
(b)
(c)
(d)
(e)
(f)
$n$ = 87
$n$ = 87
0.0
0.1
0.2
0.3
0.4
0.5
0.6
0.0
0.2
0.4
0.6
0.8
1.0
140
160
180
200
220
0.0
0.2
0.4
0.6
0.8
1.0
1.2
350
400
450
500
550
600
Shade index
Spatial drought index (−MPa)
Inter−annual drought index (−mm)

# *Guarea guidonia*

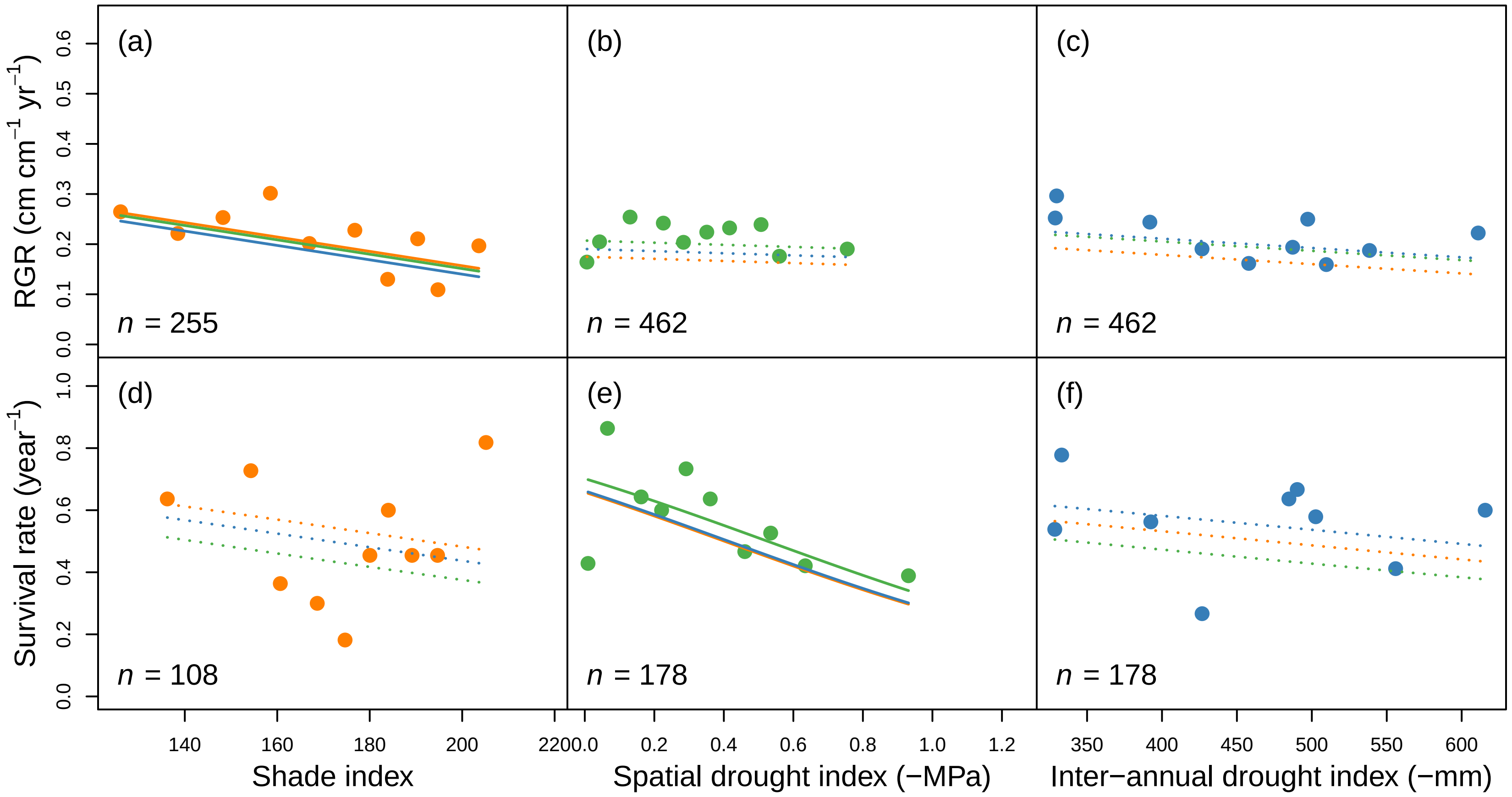

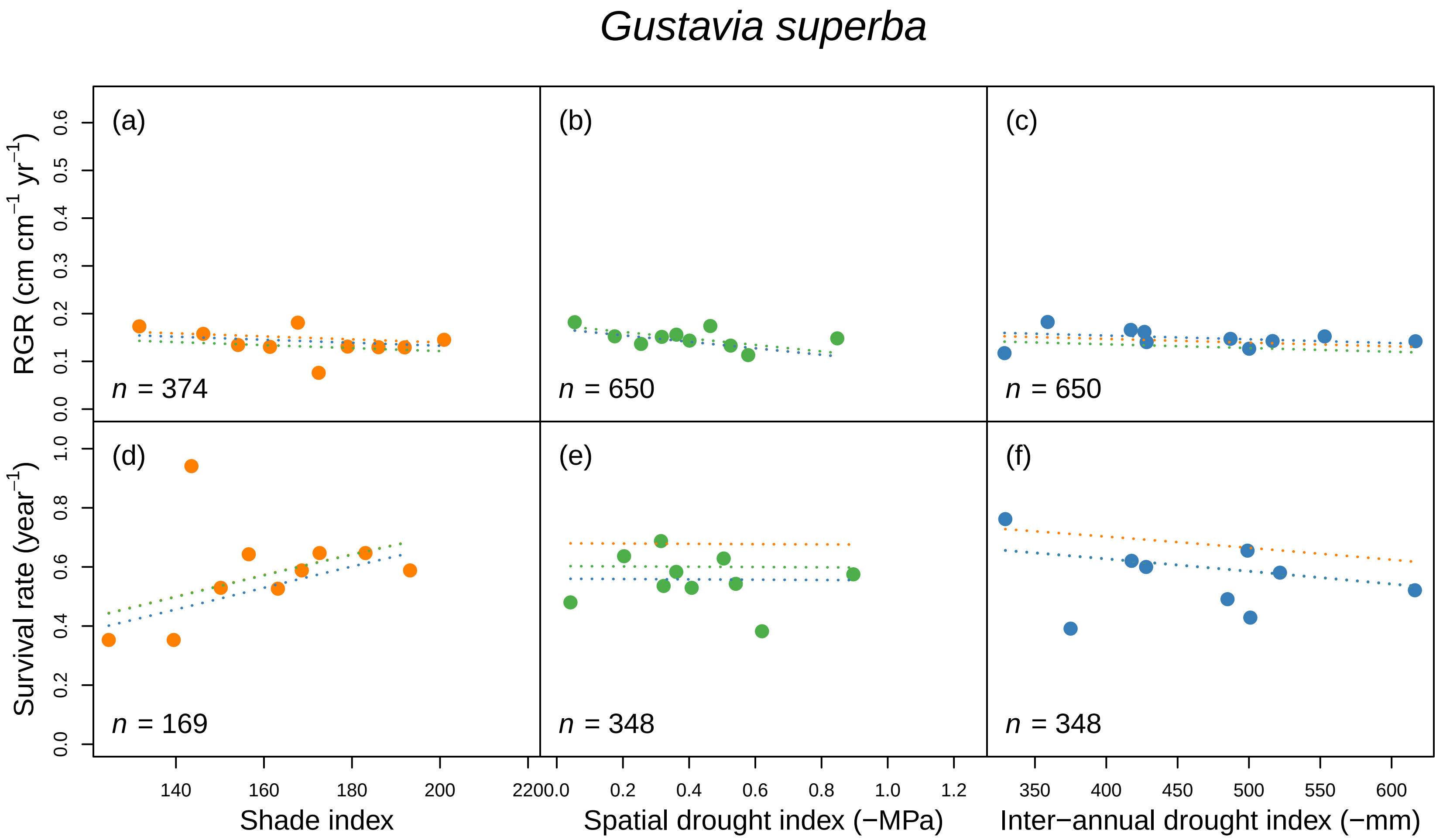

Gustavia superba
(a)
(b)
(c)
(d)
(e)
(f)
n = 374
n = 650
n = 650
n = 169
n = 348
n = 348
RGR (cm cm−1 yr−1)
Survival rate (year−1)
0.0
0.1
0.2
0.3
0.4
0.5
0.6
0.0
0.2
0.4
0.6
0.8
1.0
140
160
180
200
220
0.0
0.2
0.4
0.6
0.8
1.0
1.2
350
400
450
500
550
600
Shade index
Spatial drought index (−MPa)
Inter−annual drought index (−mm)

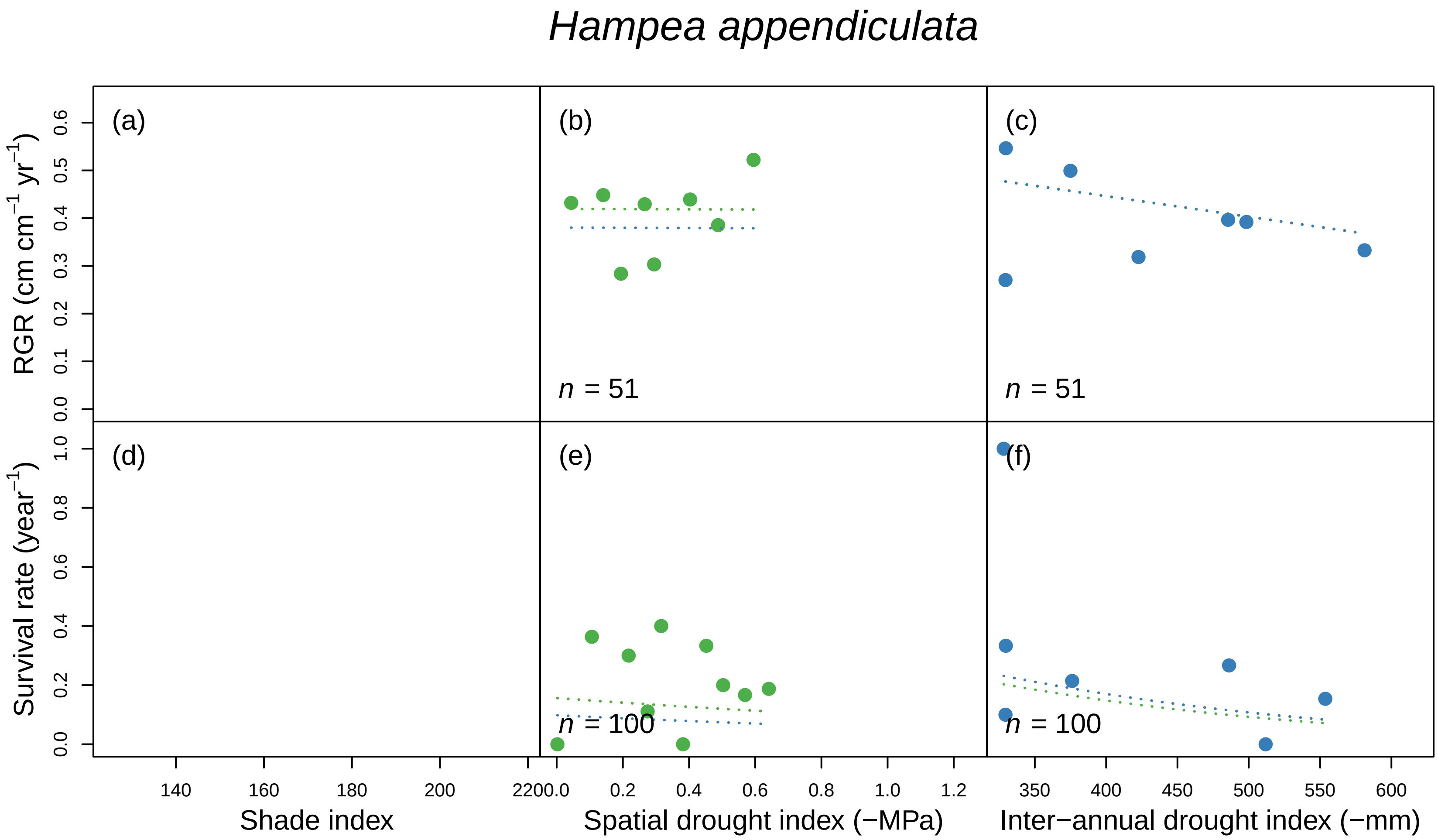

Hampea appendiculata
RGR (cm cm−1 yr−1)
Survival rate (year−1)
(a)
(b)
(c)
(d)
(e)
(f)
n = 51
n = 51
n = 100
n = 100
0.0
0.1
0.2
0.3
0.4
0.5
0.6
0.0
0.2
0.4
0.6
0.8
1.0
140
160
180
200
220
0.0
0.2
0.4
0.6
0.8
1.0
1.2
350
400
450
500
550
600
Shade index
Spatial drought index (−MPa)
Inter−annual drought index (−mm)

# *Heisteria acuminata*

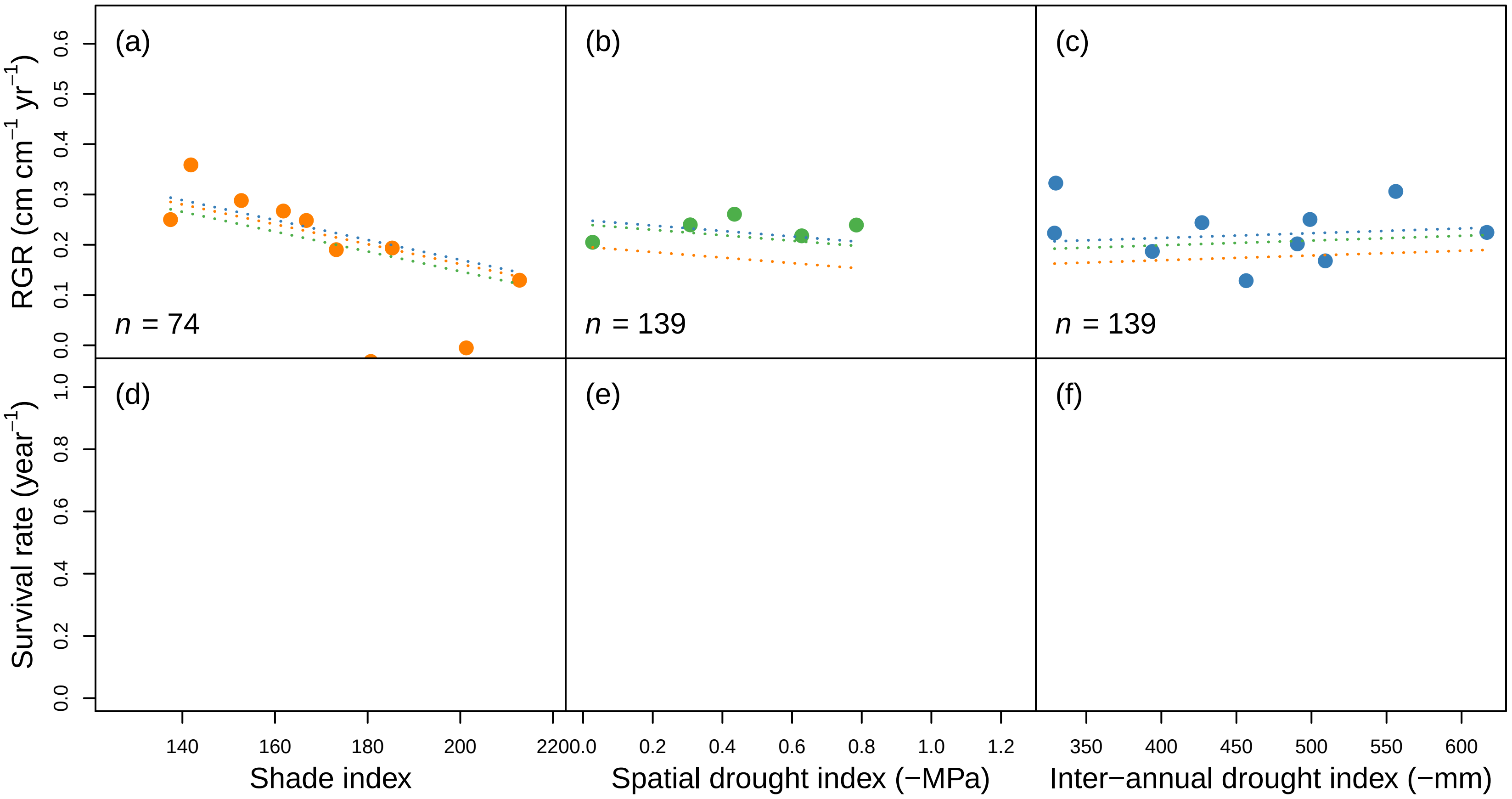

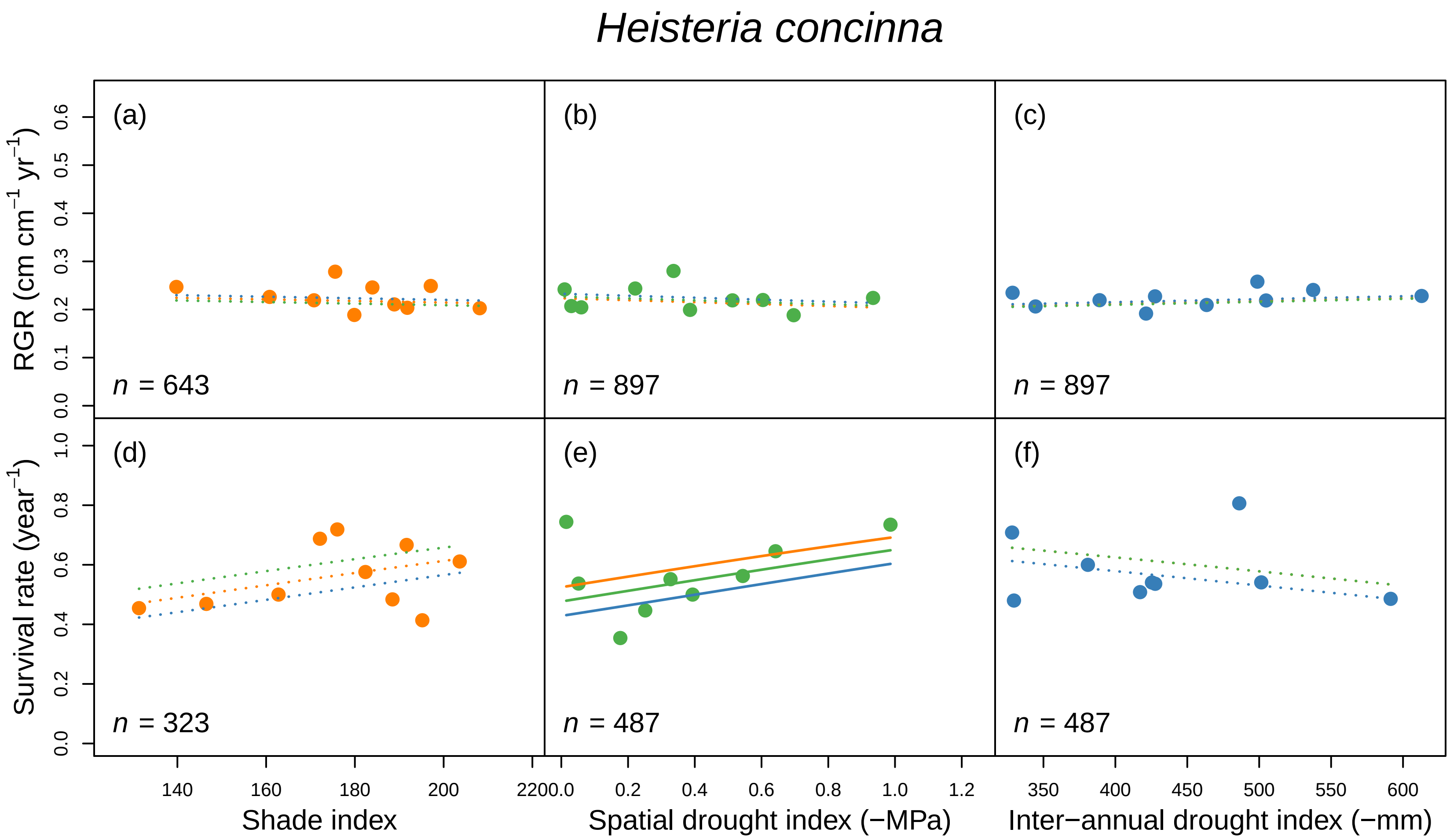

Heisteria concinna
(a)
(b)
(c)
(d)
(e)
(f)
n = 643
n = 897
n = 897
n = 323
n = 487
n = 487
RGR (cm cm−1 yr−1)
Survival rate (year−1)
0.0
0.1
0.2
0.3
0.4
0.5
0.6
0.0
0.2
0.4
0.6
0.8
1.0
140
160
180
200
220
0.0
0.2
0.4
0.6
0.8
1.0
1.2
350
400
450
500
550
600
Shade index
Spatial drought index (−MPa)
Inter−annual drought index (−mm)

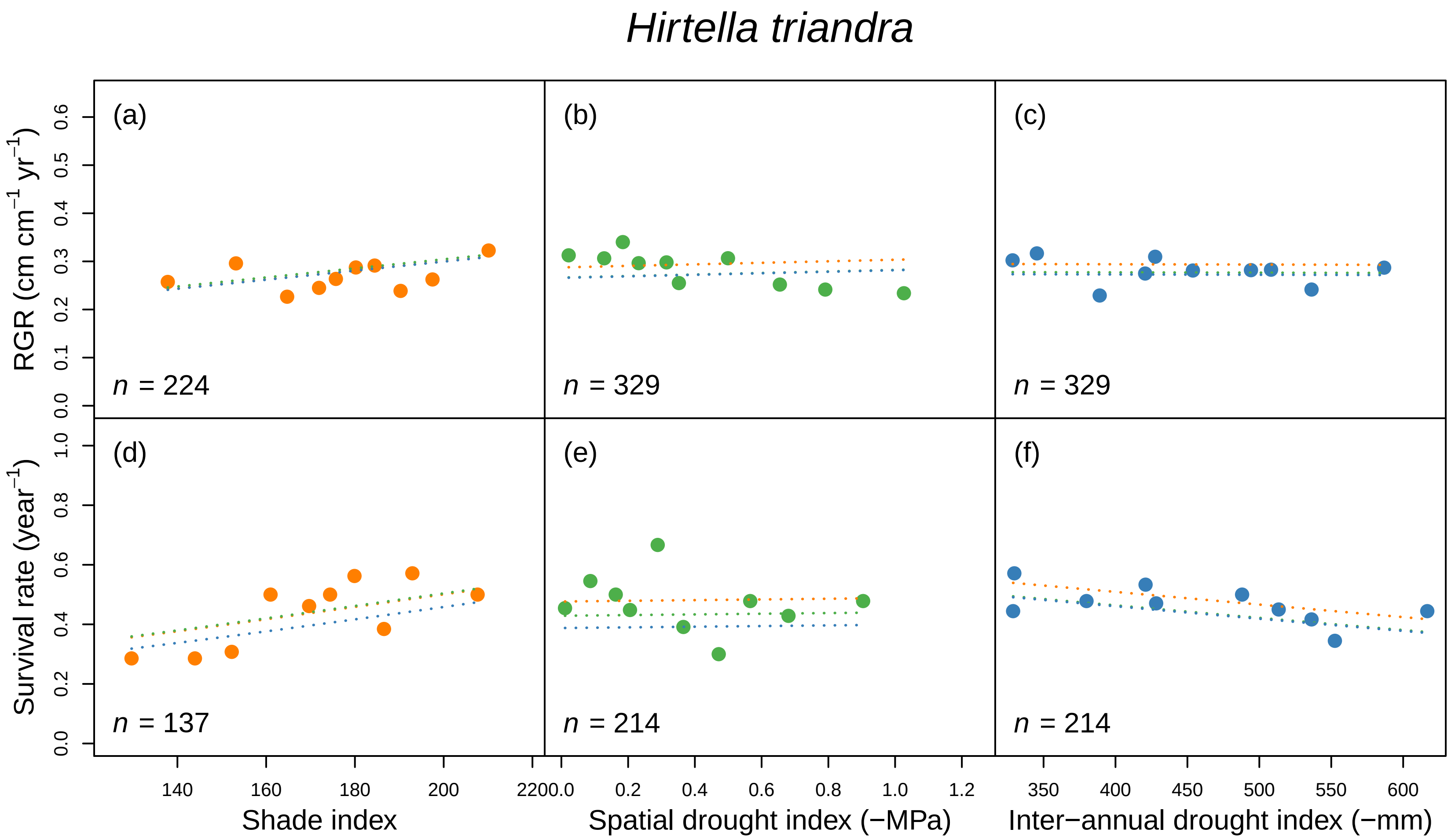

Hirtella triandra
(a)
(b)
(c)
(d)
(e)
(f)
n = 224
n = 329
n = 329
n = 137
n = 214
n = 214
RGR (cm cm$^{-1}$ yr$^{-1}$)
Survival rate (year$^{-1}$)
0.0
0.1
0.2
0.3
0.4
0.5
0.6
0.0
0.2
0.4
0.6
0.8
1.0
140
160
180
200
220
0.0
0.2
0.4
0.6
0.8
1.0
1.2
350
400
450
500
550
600
Shade index
Spatial drought index (−MPa)
Inter−annual drought index (−mm)

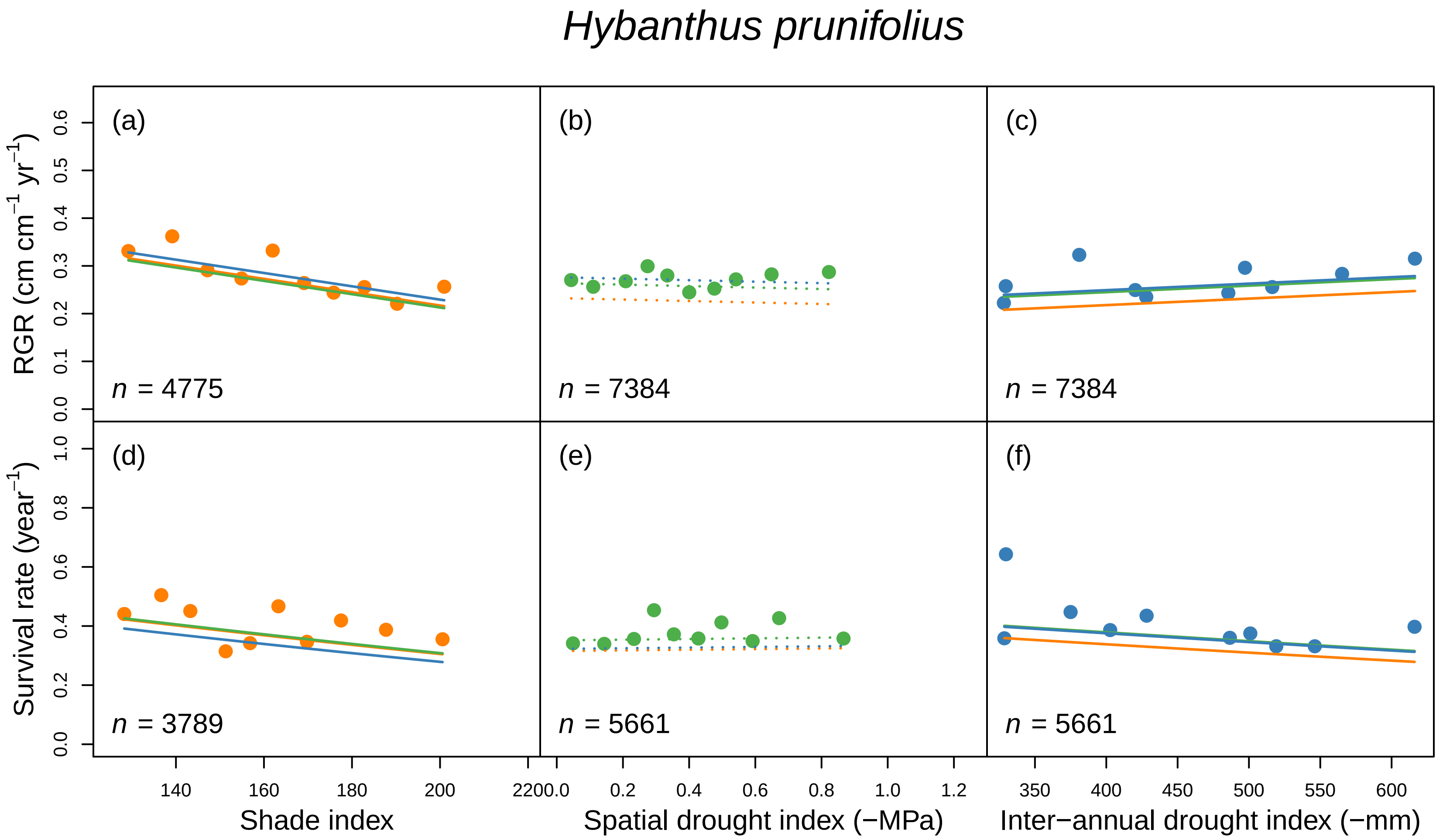

Hybanthus prunifolius
(a)
(b)
(c)
(d)
(e)
(f)
n = 4775
n = 7384
n = 7384
n = 3789
n = 5661
n = 5661
RGR (cm cm−1 yr−1)
Survival rate (year−1)
Shade index
Spatial drought index (−MPa)
Inter−annual drought index (−mm)

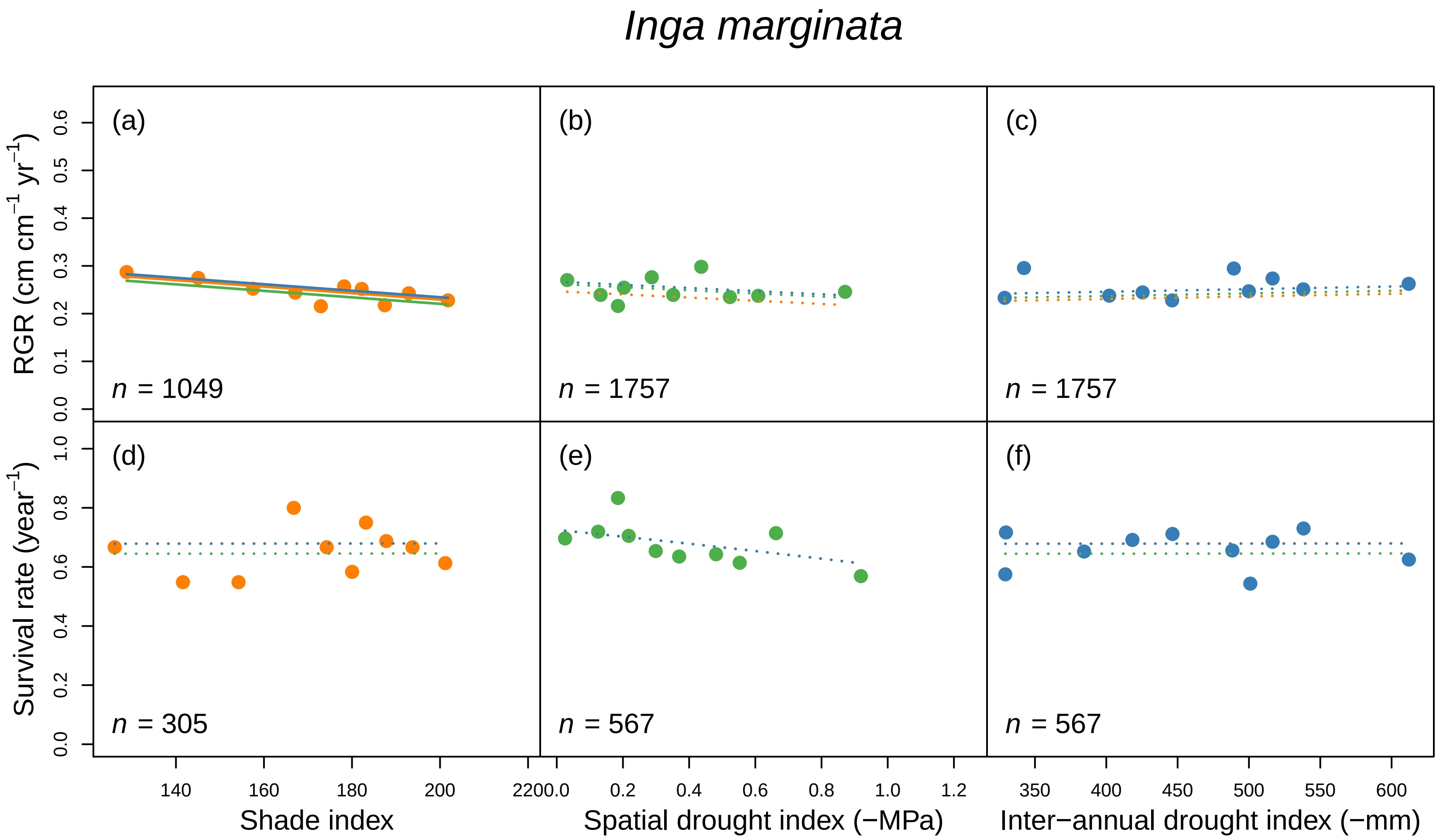
Inga marginata
RGR (cm cm$^{-1}$ yr$^{-1}$)
Survival rate (year$^{-1}$)
(a)
(b)
(c)
(d)
(e)
(f)
n = 1049
n = 1757
n = 1757
n = 305
n = 567
n = 567
Shade index
Spatial drought index (−MPa)
Inter−annual drought index (−mm)

# *Inga multijuga*

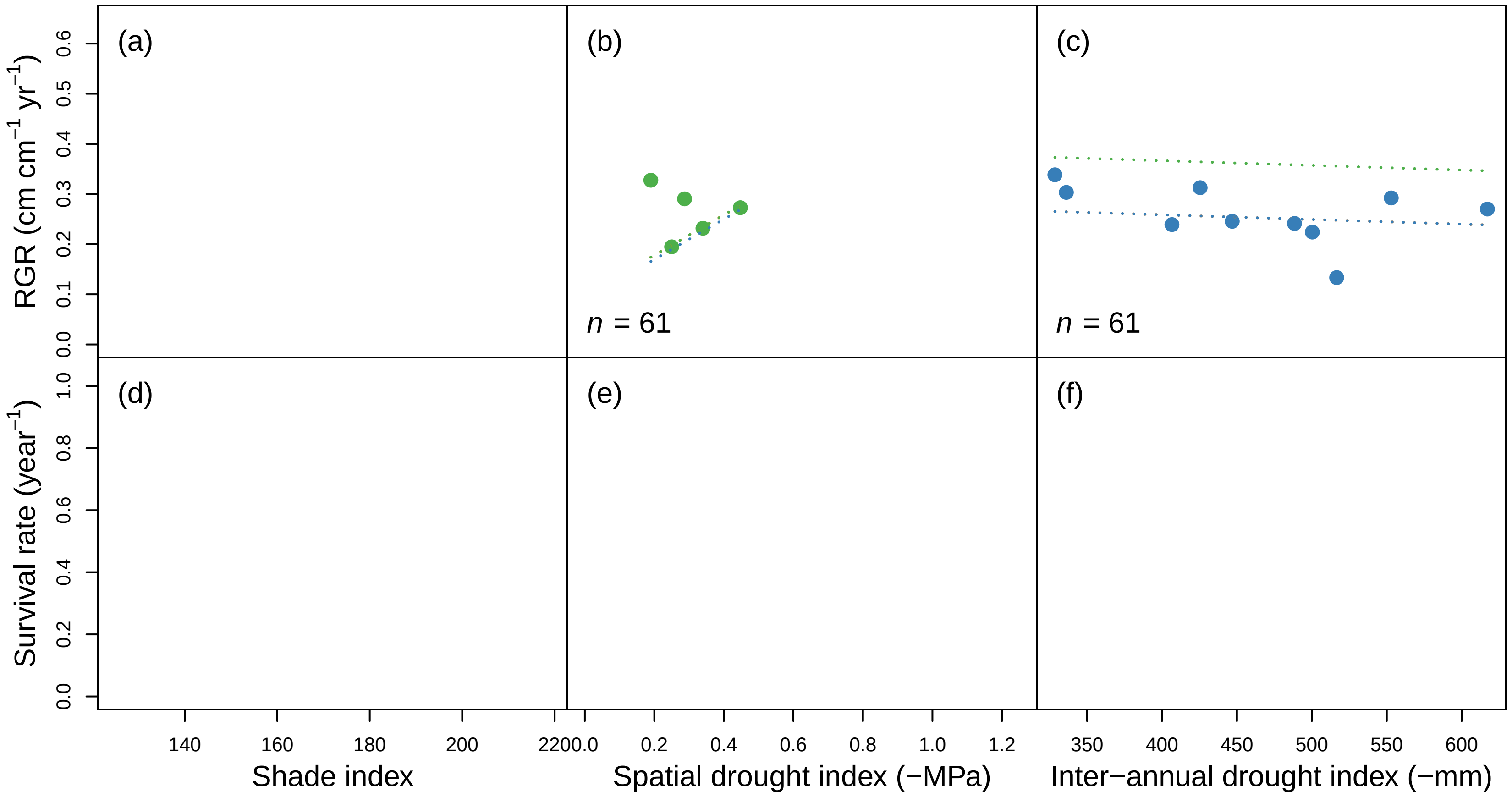

# *Inga nobilis*

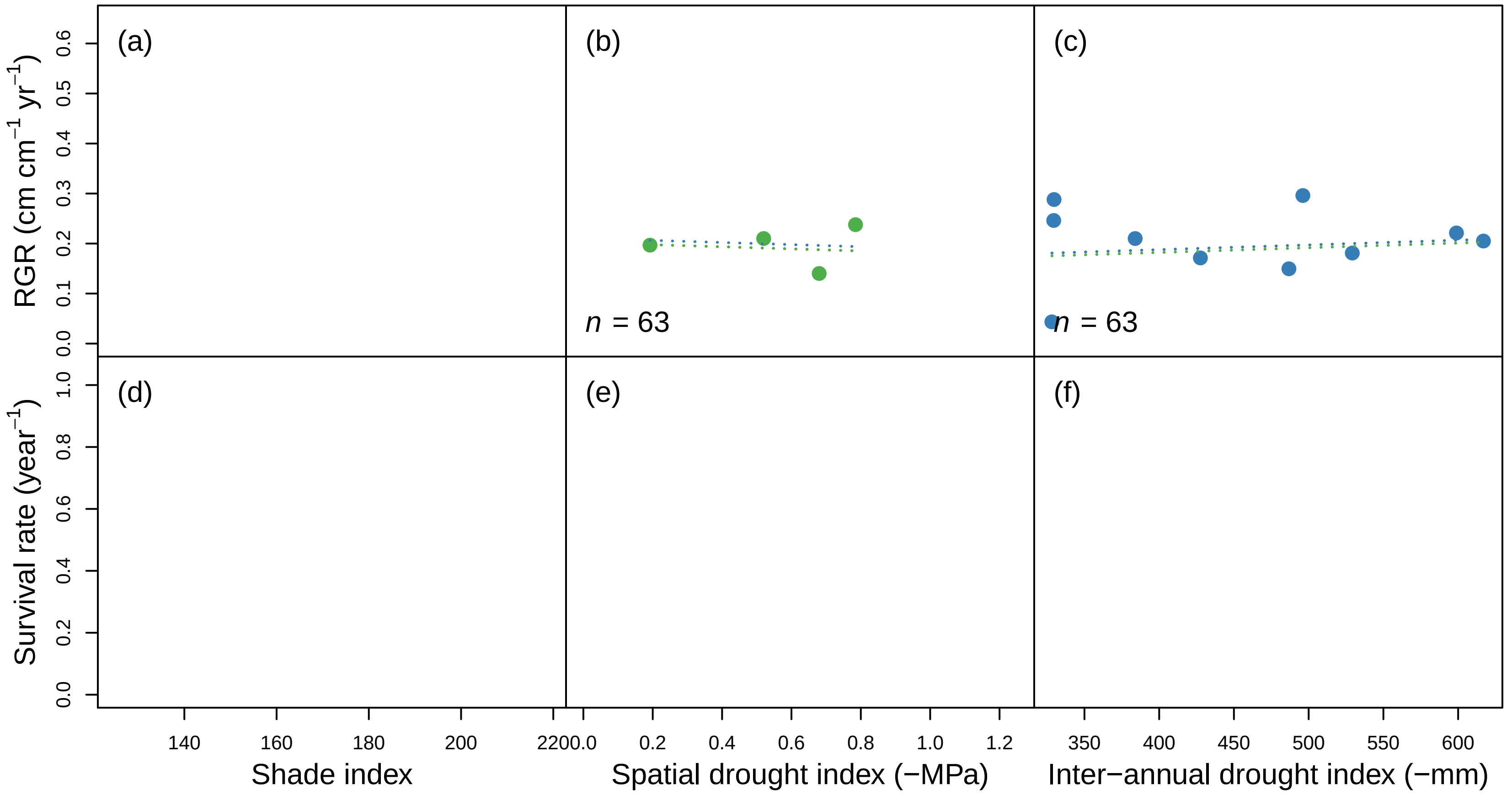

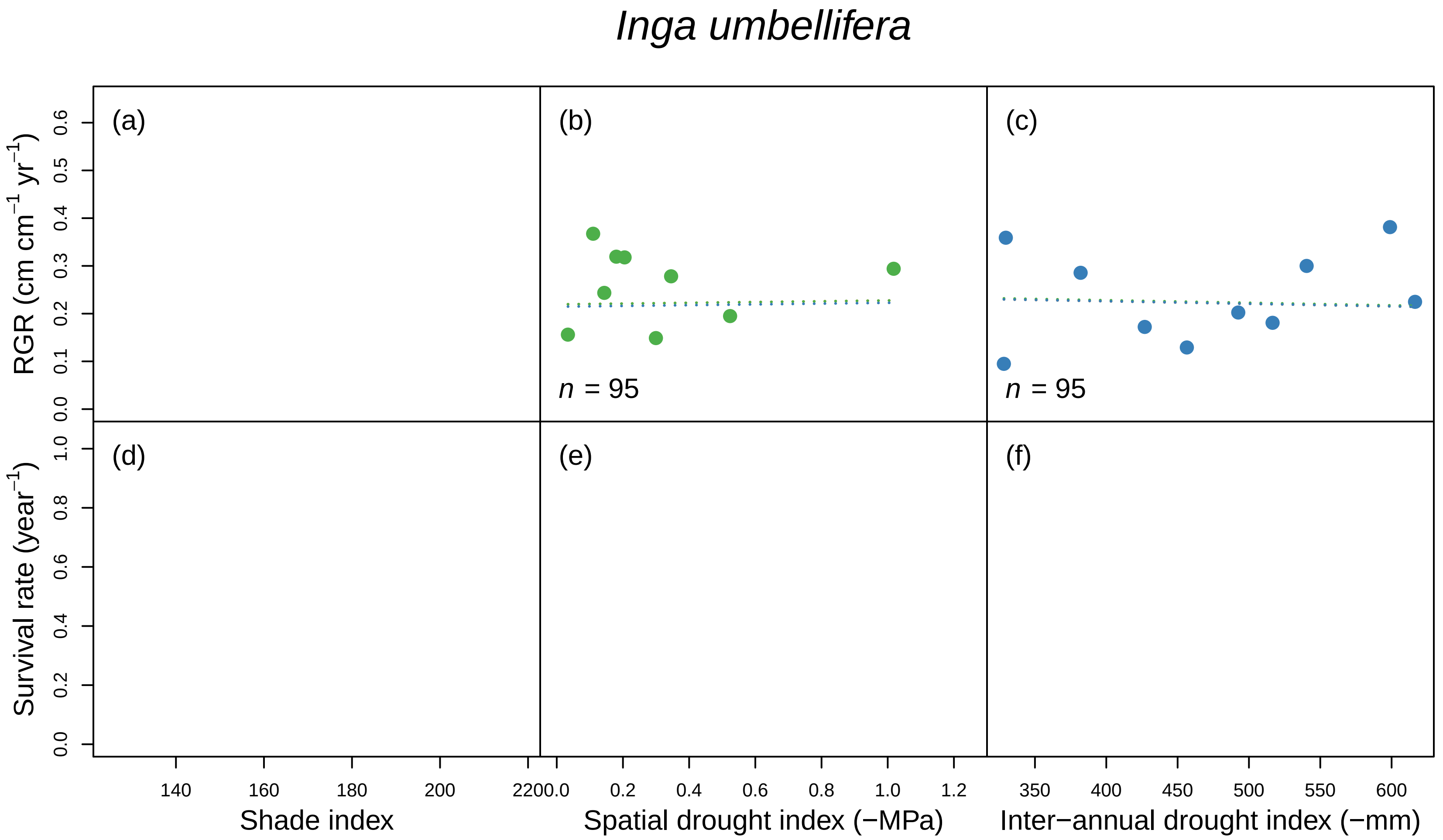

Inga umbellifera
(a)
(b)
(c)
(d)
(e)
(f)
n = 95
n = 95
RGR (cm cm−1 yr−1)
Survival rate (year−1)
0.0
0.1
0.2
0.3
0.4
0.5
0.6
0.0
0.2
0.4
0.6
0.8
1.0
140
160
180
200
220
0.0
0.2
0.4
0.6
0.8
1.0
1.2
350
400
450
500
550
600
Shade index
Spatial drought index (−MPa)
Inter−annual drought index (−mm)

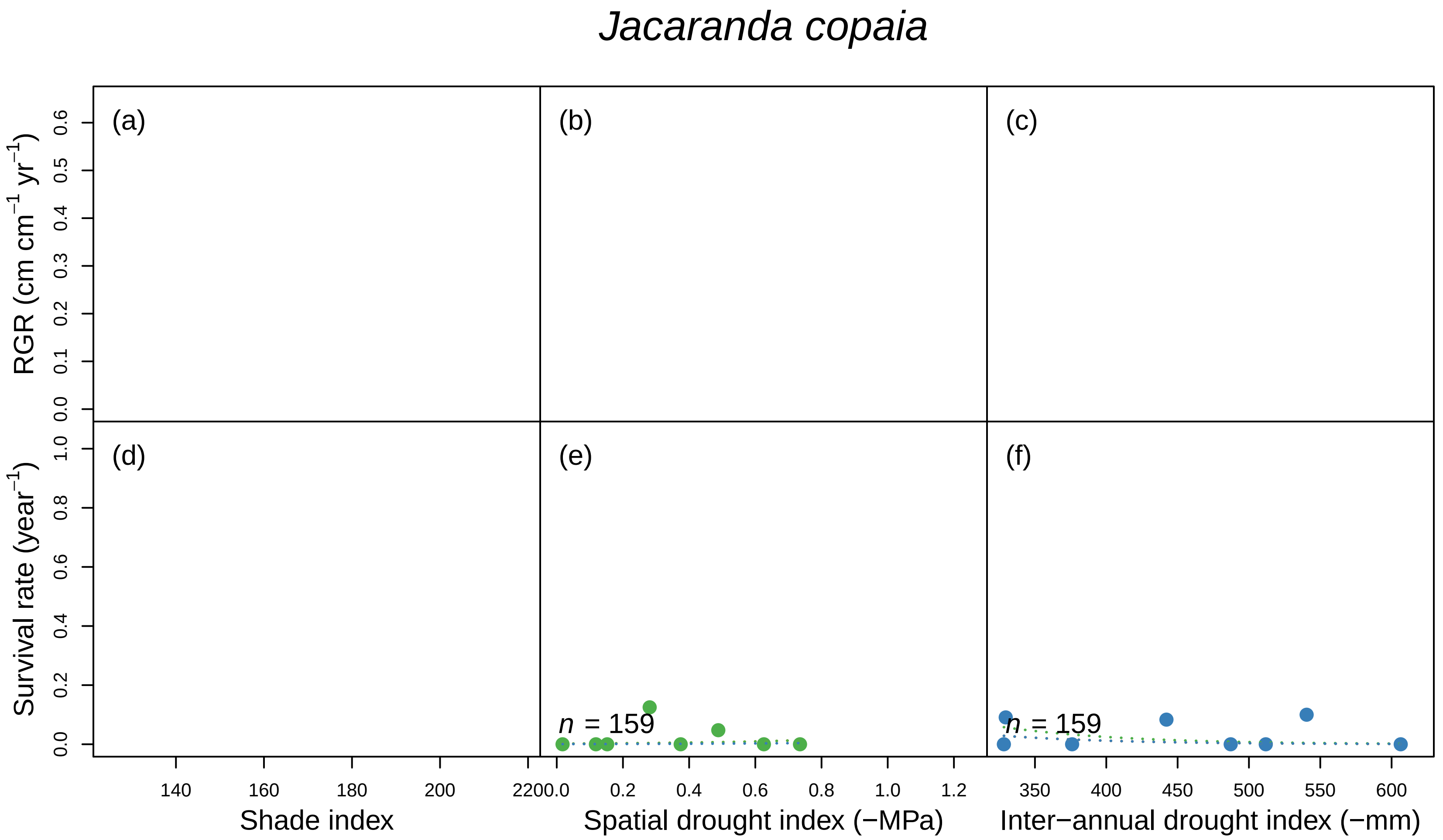
Jacaranda copaia
(a)
(b)
(c)
(d)
(e)
(f)
RGR (cm cm−1 yr−1)
Survival rate (year−1)
n = 159
n = 159
Shade index
Spatial drought index (−MPa)
Inter−annual drought index (−mm)

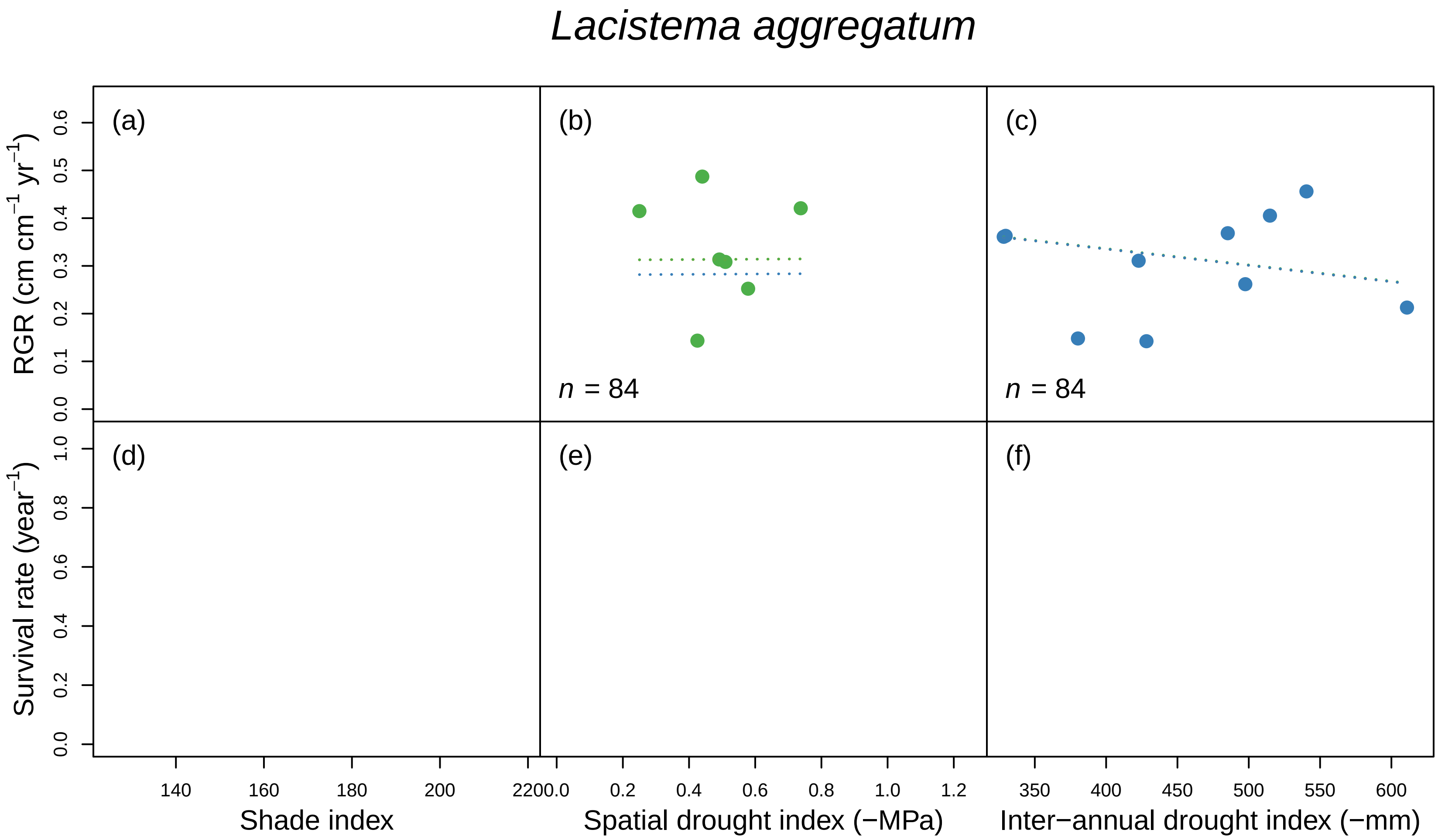
Lacistema aggregatum
RGR (cm cm−1 yr−1)
Survival rate (year−1)
(a)
(b)
(c)
(d)
(e)
(f)
n = 84
n = 84
0.0
0.1
0.2
0.3
0.4
0.5
0.6
0.0
0.2
0.4
0.6
0.8
1.0
140
160
180
200
220
0.0
0.2
0.4
0.6
0.8
1.0
1.2
350
400
450
500
550
600
Shade index
Spatial drought index (−MPa)
Inter−annual drought index (−mm)

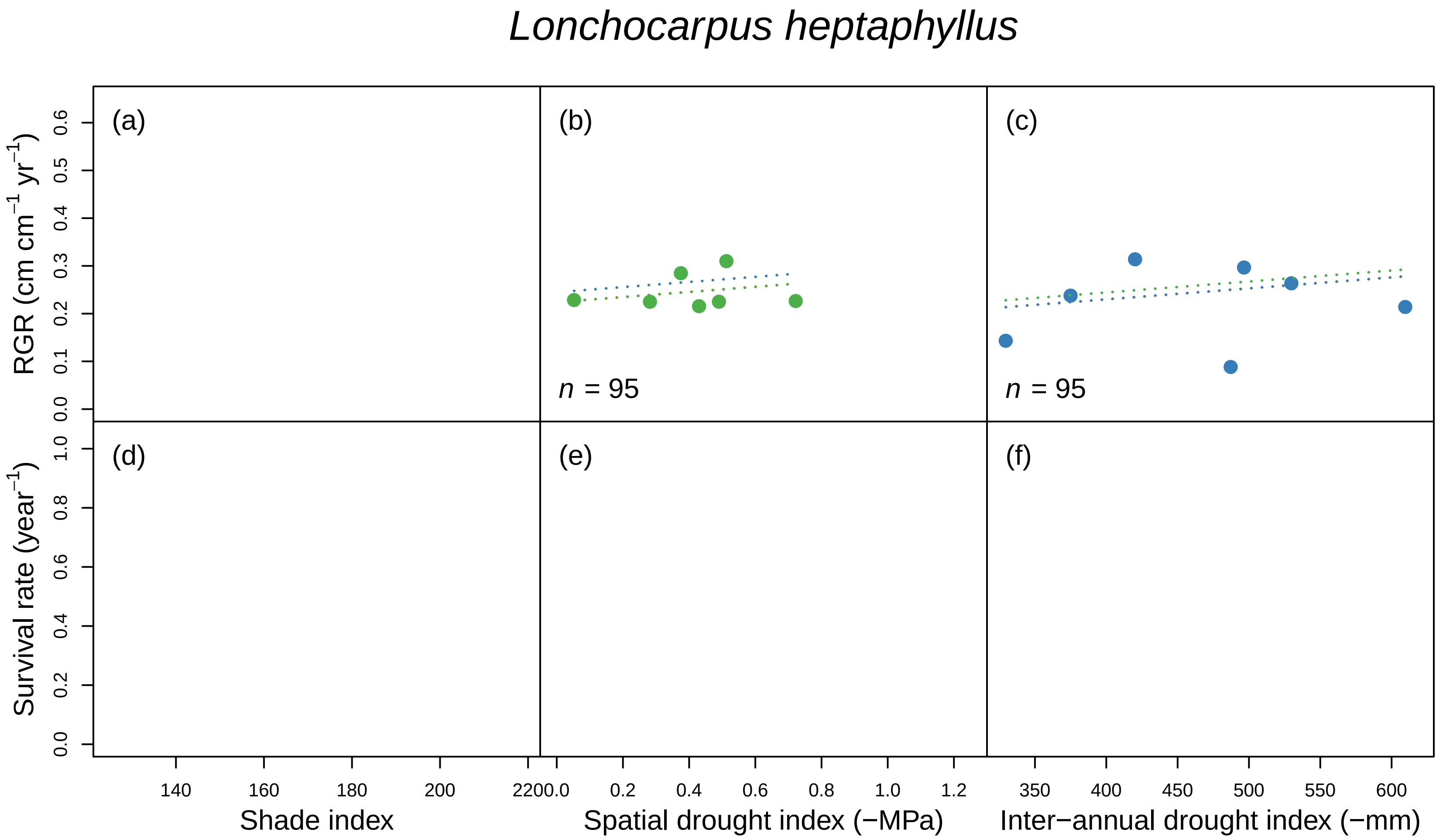

Lonchocarpus heptaphyllus
(a)
(b)
(c)
(d)
(e)
(f)
n = 95
n = 95
RGR (cm cm−1 yr−1)
Survival rate (year−1)
0.0
0.1
0.2
0.3
0.4
0.5
0.6
0.0
0.2
0.4
0.6
0.8
1.0
140
160
180
200
220
0.0
0.2
0.4
0.6
0.8
1.0
1.2
350
400
450
500
550
600
Shade index
Spatial drought index (−MPa)
Inter−annual drought index (−mm)

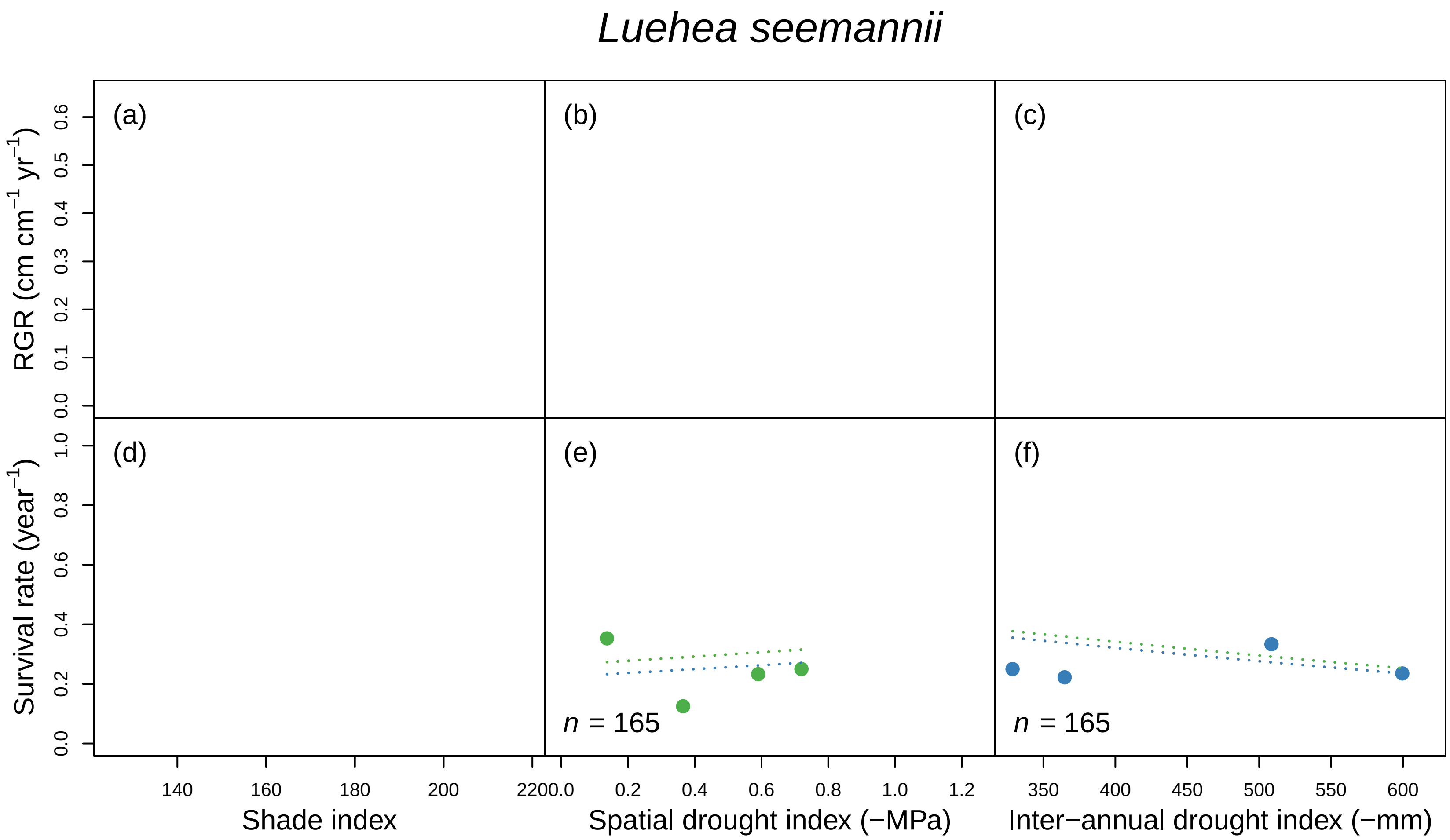

Luehea seemannii
RGR (cm cm−1 yr−1)
Survival rate (year−1)
(a)
(b)
(c)
(d)
(e)
(f)
n = 165
n = 165
0.0
0.1
0.2
0.3
0.4
0.5
0.6
0.0
0.2
0.4
0.6
0.8
1.0
140
160
180
200
220
0.0
0.2
0.4
0.6
0.8
1.0
1.2
350
400
450
500
550
600
Shade index
Spatial drought index (−MPa)
Inter−annual drought index (−mm)

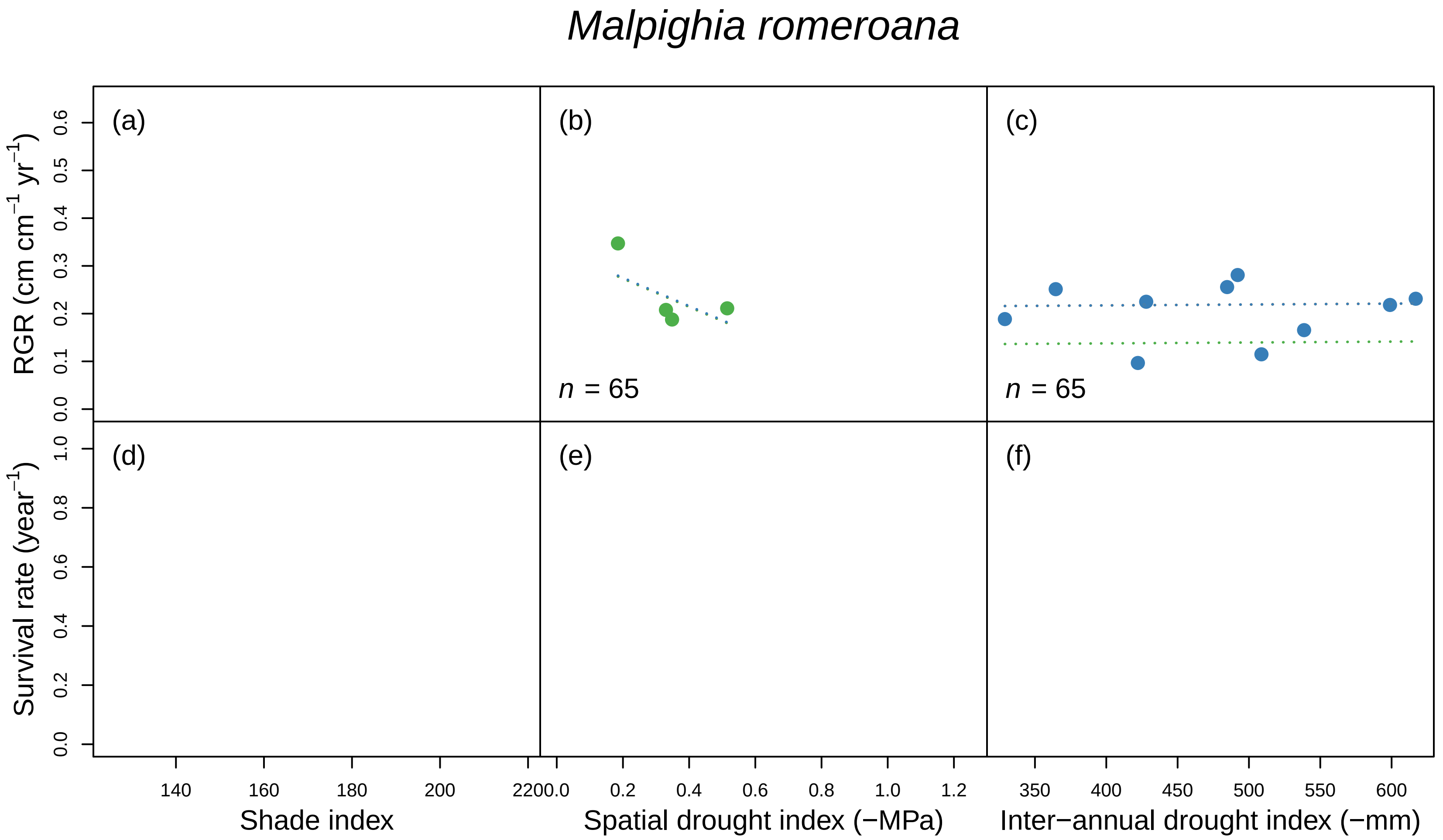
Malpighia romeroana
RGR (cm cm−1 yr−1)
Survival rate (year−1)
(a)
(b)
(c)
(d)
(e)
(f)
n = 65
n = 65
0.0
0.1
0.2
0.3
0.4
0.5
0.6
0.0
0.2
0.4
0.6
0.8
1.0
140
160
180
200
220
0.0
0.2
0.4
0.6
0.8
1.0
1.2
350
400
450
500
550
600
Shade index
Spatial drought index (−MPa)
Inter−annual drought index (−mm)

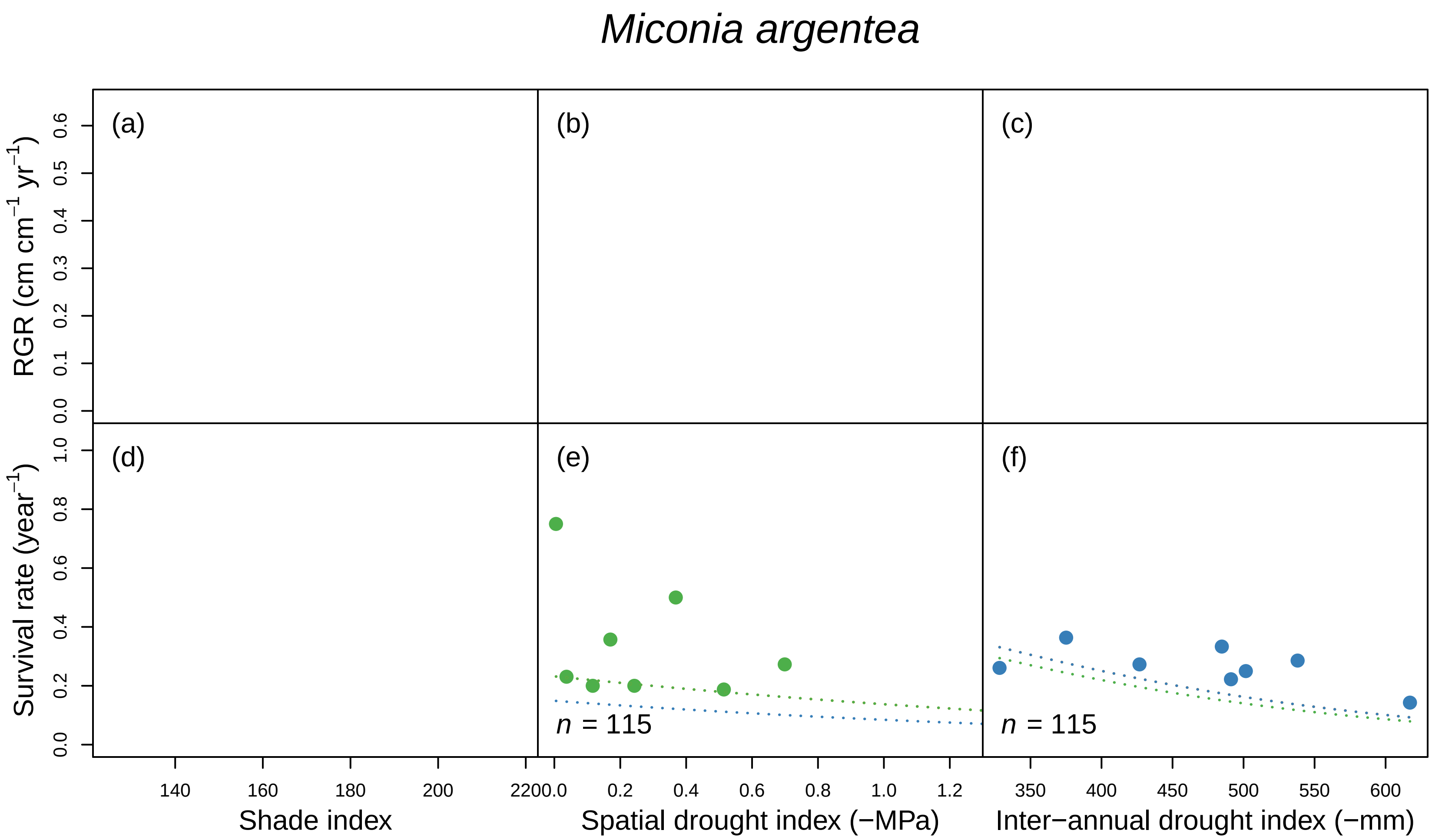

Miconia argentea
(a)
(b)
(c)
(d)
(e)
(f)
RGR (cm cm−1 yr−1)
Survival rate (year−1)
n = 115
n = 115
Shade index
Spatial drought index (−MPa)
Inter−annual drought index (−mm)

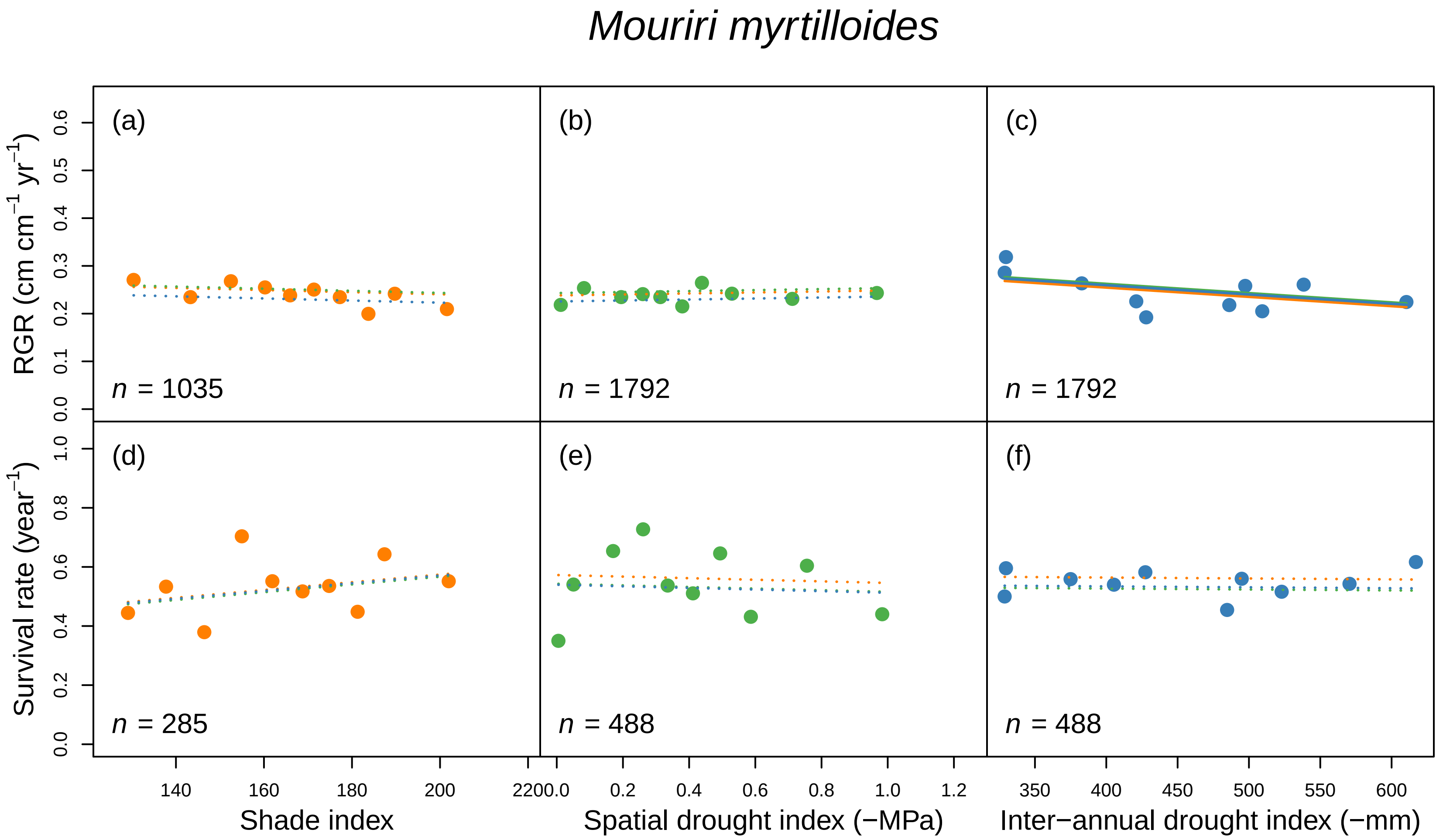
Mouriri myrtilloides
(a)
(b)
(c)
(d)
(e)
(f)
n = 1035
n = 1792
n = 1792
n = 285
n = 488
n = 488
RGR (cm cm−1 yr−1)
Survival rate (year−1)
0.0
0.1
0.2
0.3
0.4
0.5
0.6
0.0
0.2
0.4
0.6
0.8
1.0
140
160
180
200
220
0.0
0.2
0.4
0.6
0.8
1.0
1.2
350
400
450
500
550
600
Shade index
Spatial drought index (−MPa)
Inter−annual drought index (−mm)

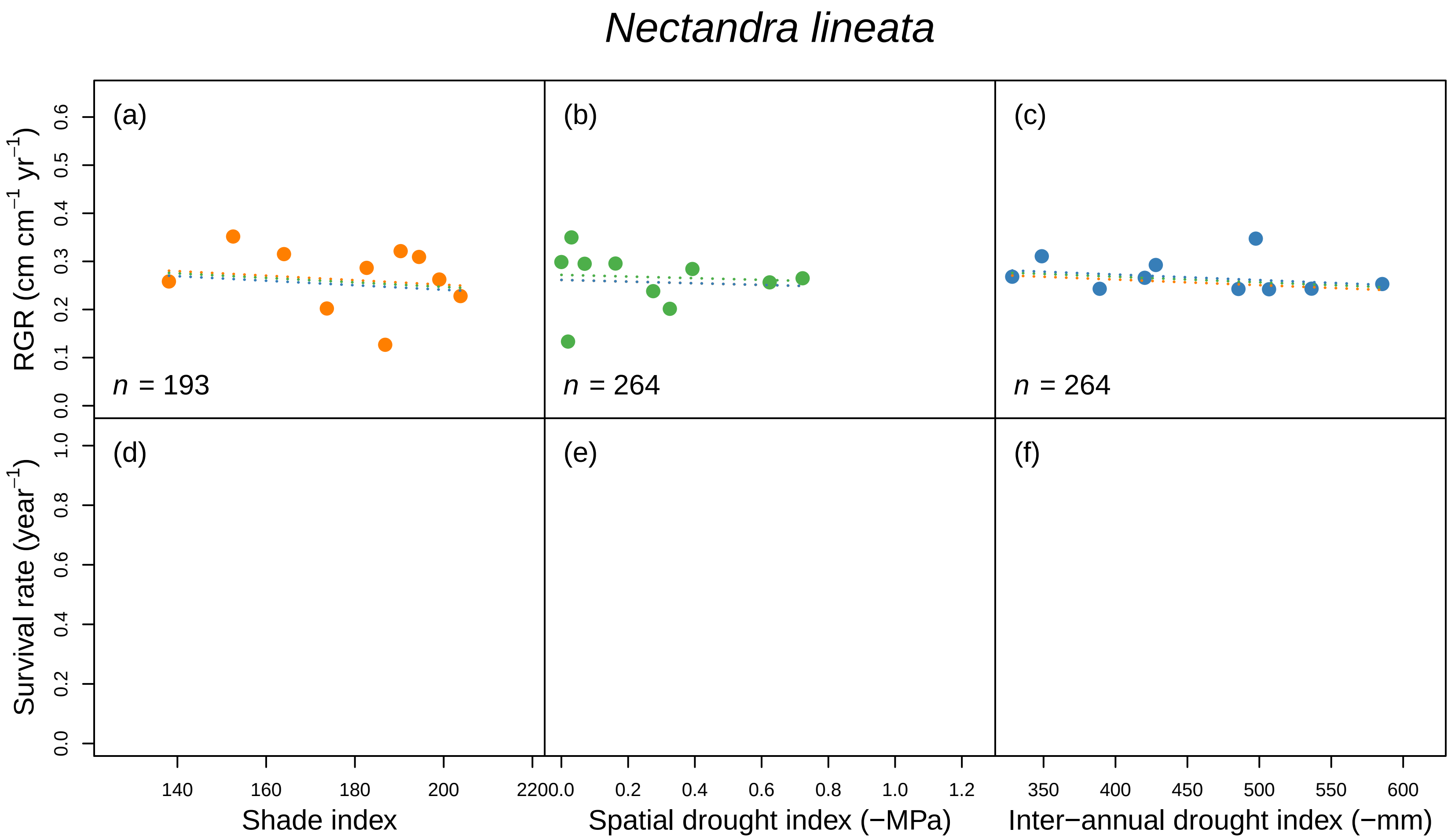

Nectandra lineata
(a)
(b)
(c)
(d)
(e)
(f)
n = 193
n = 264
n = 264
RGR (cm cm−1 yr−1)
Survival rate (year−1)
0.0
0.1
0.2
0.3
0.4
0.5
0.6
0.0
0.2
0.4
0.6
0.8
1.0
140
160
180
200
220
0.0
0.2
0.4
0.6
0.8
1.0
1.2
350
400
450
500
550
600
Shade index
Spatial drought index (−MPa)
Inter−annual drought index (−mm)

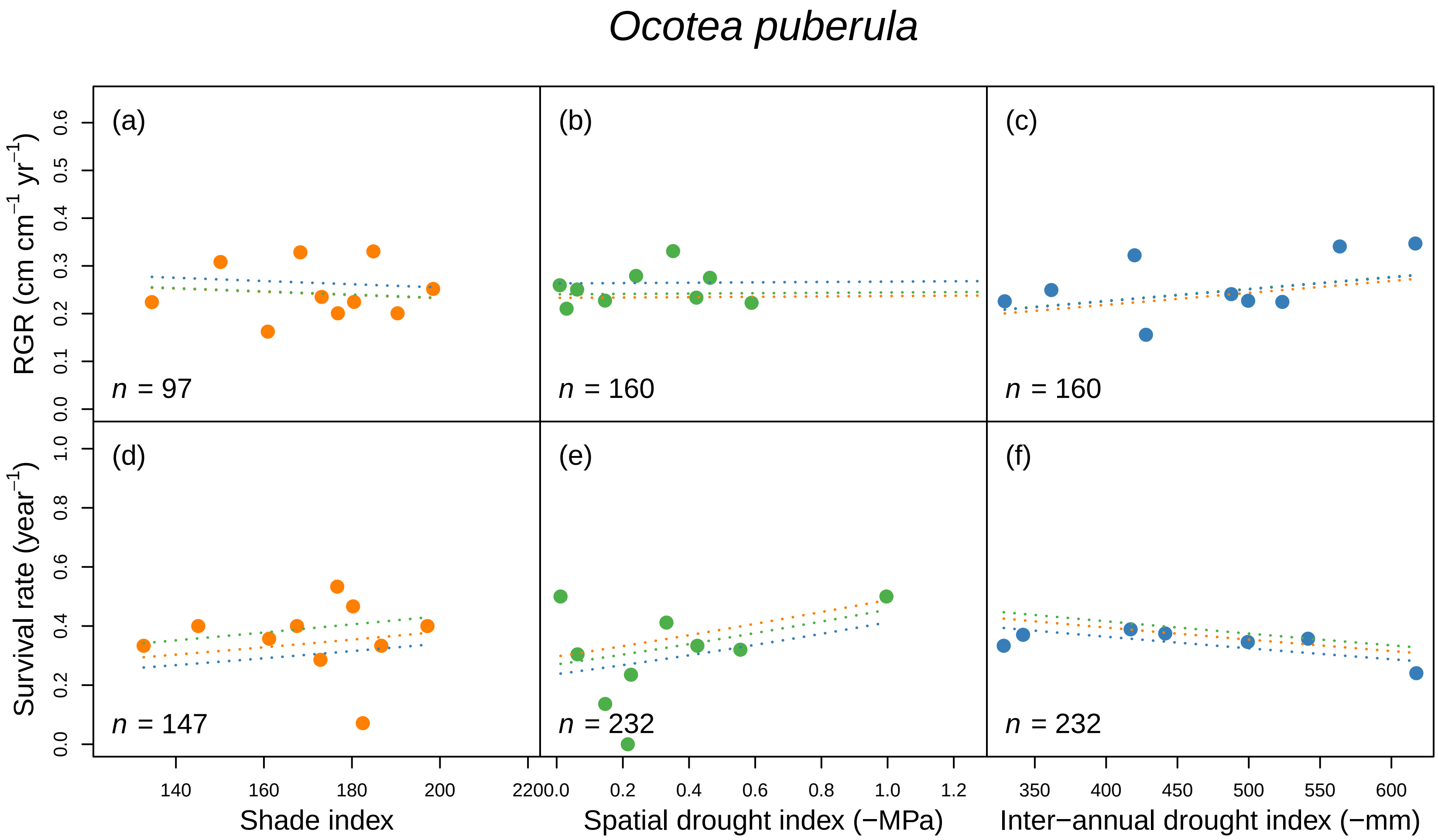

Ocotea puberula
(a)
(b)
(c)
(d)
(e)
(f)
n = 97
n = 160
n = 160
n = 147
n = 232
n = 232
RGR (cm cm−1 yr−1)
Survival rate (year−1)
Shade index
Spatial drought index (−MPa)
Inter−annual drought index (−mm)

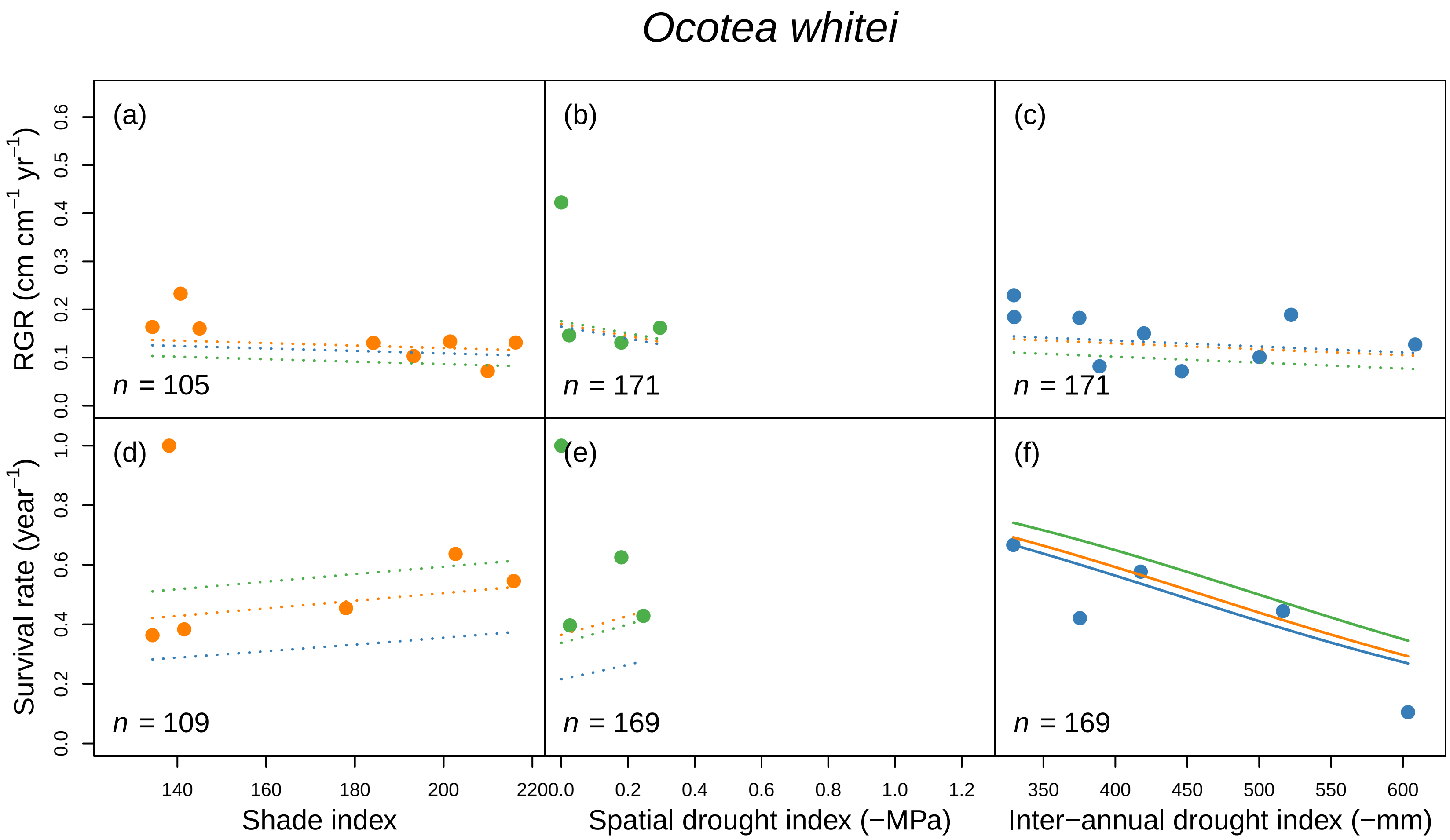
Ocotea whitei
RGR (cm cm−1 yr−1)
Survival rate (year−1)
(a)
(b)
(c)
(d)
(e)
(f)
n = 105
n = 171
n = 171
n = 109
n = 169
n = 169
0.0
0.1
0.2
0.3
0.4
0.5
0.6
0.0
0.2
0.4
0.6
0.8
1.0
140
160
180
200
220
0.0
0.2
0.4
0.6
0.8
1.0
1.2
350
400
450
500
550
600
Shade index
Spatial drought index (−MPa)
Inter−annual drought index (−mm)

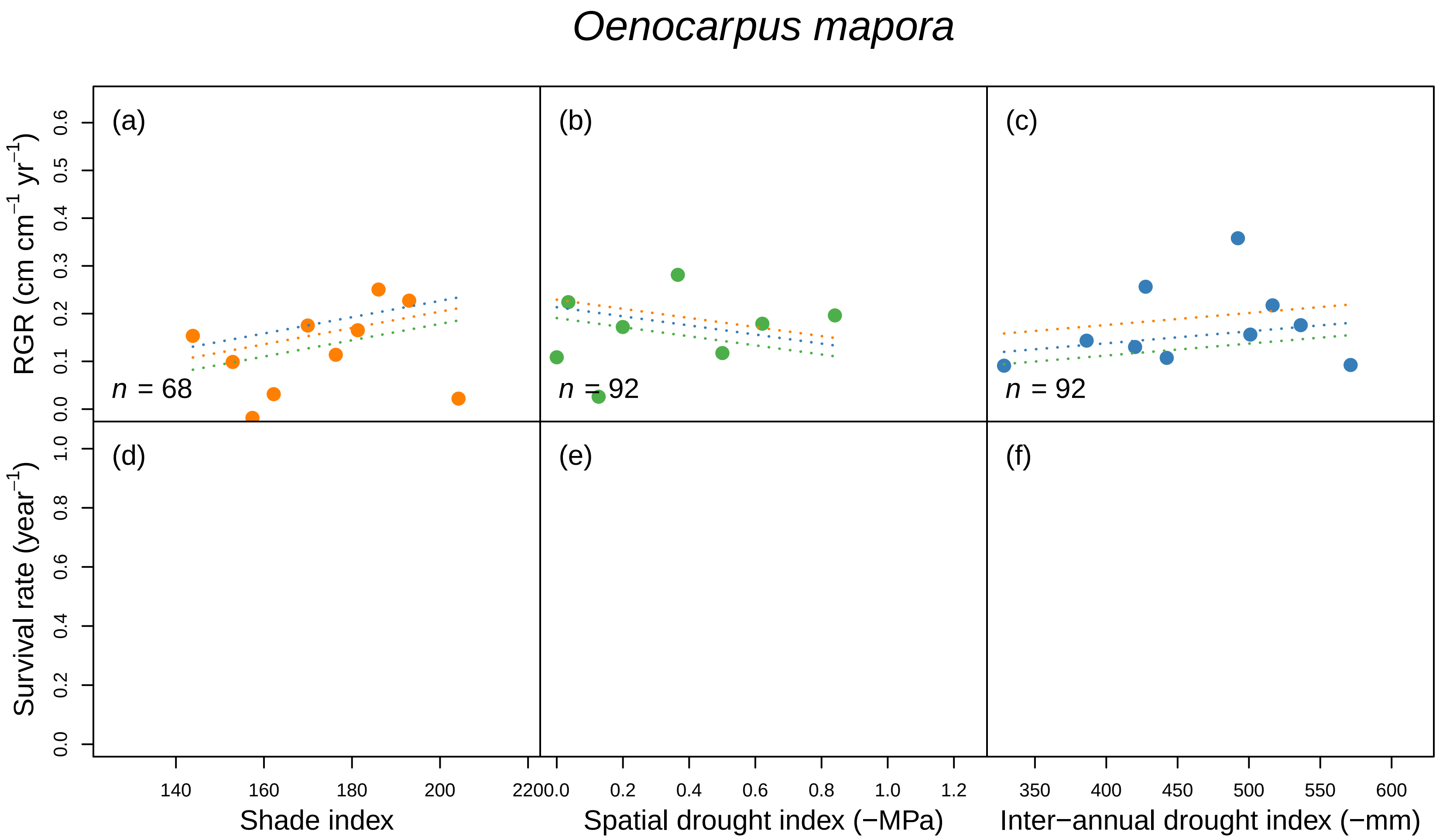

Oenocarpus mapora
(a)
(b)
(c)
(d)
(e)
(f)
n = 68
n = 92
n = 92
RGR (cm cm−1 yr−1)
Survival rate (year−1)
0.0
0.1
0.2
0.3
0.4
0.5
0.6
0.0
0.2
0.4
0.6
0.8
1.0
140
160
180
200
220
0.0
0.2
0.4
0.6
0.8
1.0
1.2
350
400
450
500
550
600
Shade index
Spatial drought index (−MPa)
Inter−annual drought index (−mm)

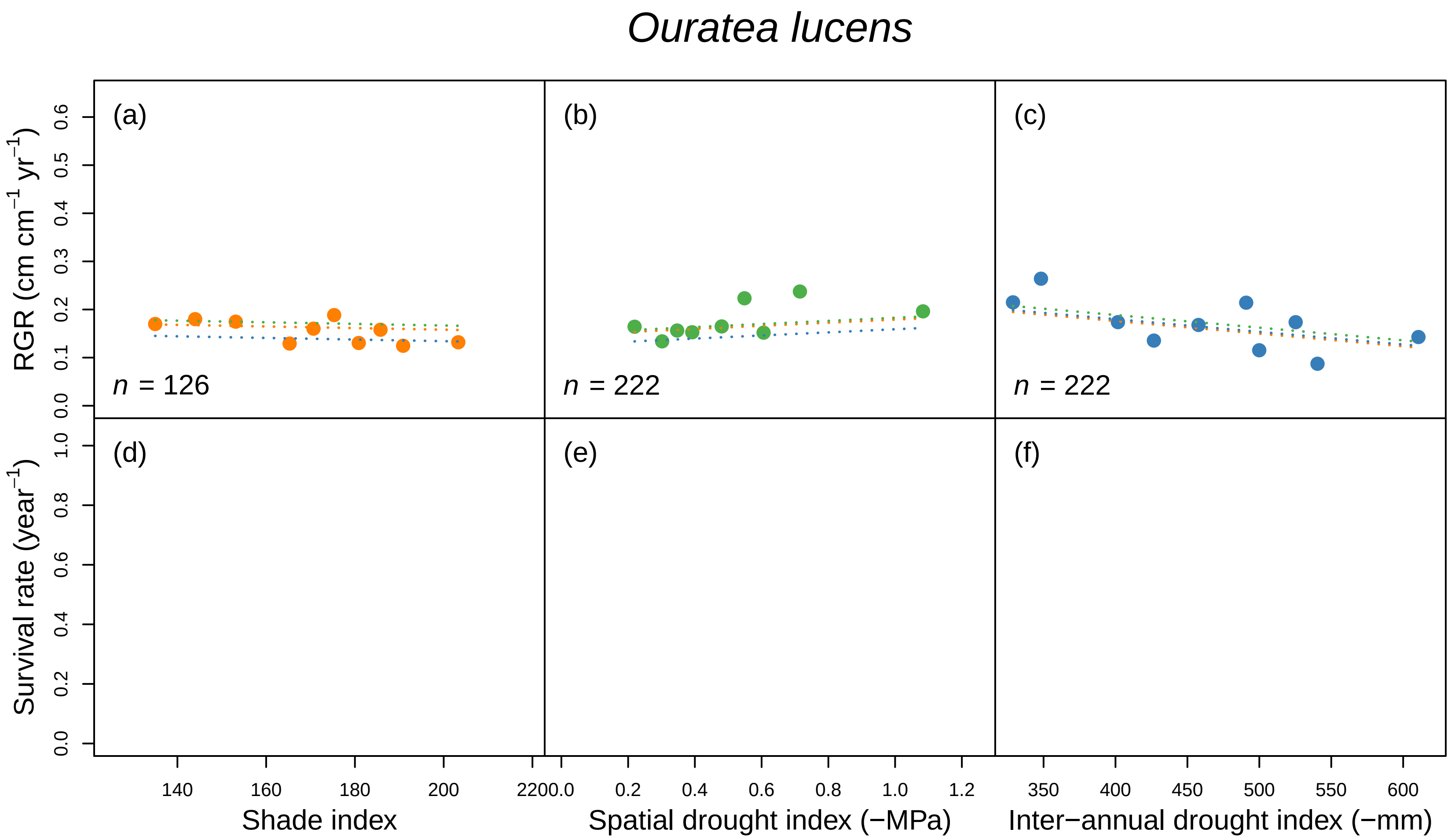

Ouratea lucens
(a)
(b)
(c)
(d)
(e)
(f)
n = 126
n = 222
n = 222
RGR (cm cm−1 yr−1)
Survival rate (year−1)
0.0
0.1
0.2
0.3
0.4
0.5
0.6
0.0
0.2
0.4
0.6
0.8
1.0
140
160
180
200
220
0.0
0.2
0.4
0.6
0.8
1.0
1.2
350
400
450
500
550
600
Shade index
Spatial drought index (−MPa)
Inter−annual drought index (−mm)

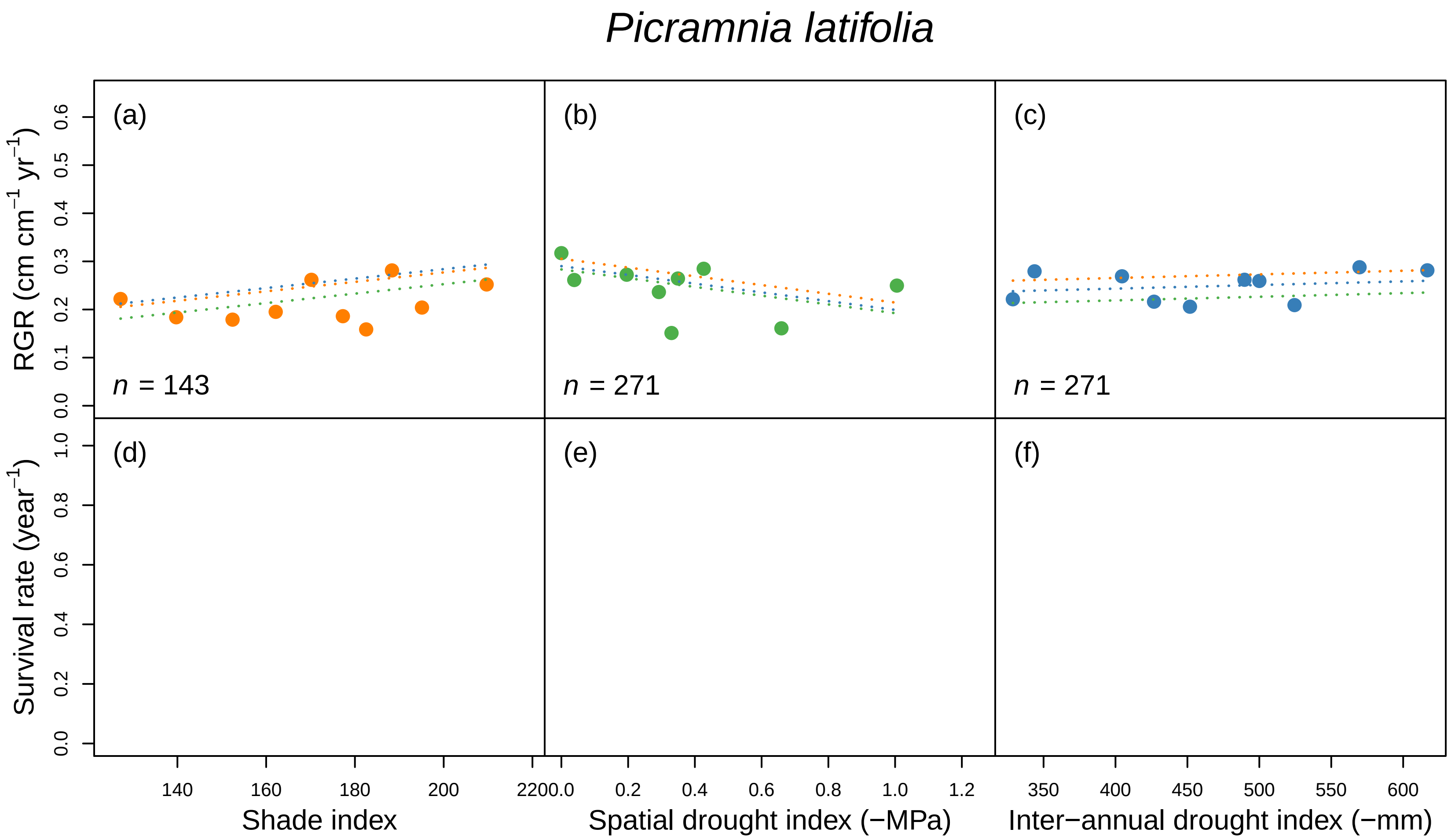

Picramnia latifolia
(a)
(b)
(c)
(d)
(e)
(f)
n = 143
n = 271
n = 271
RGR (cm cm−1 yr−1)
Survival rate (year−1)
0.0
0.1
0.2
0.3
0.4
0.5
0.6
0.0
0.2
0.4
0.6
0.8
1.0
140
160
180
200
220
0.0
0.2
0.4
0.6
0.8
1.0
1.2
350
400
450
500
550
600
Shade index
Spatial drought index (−MPa)
Inter−annual drought index (−mm)

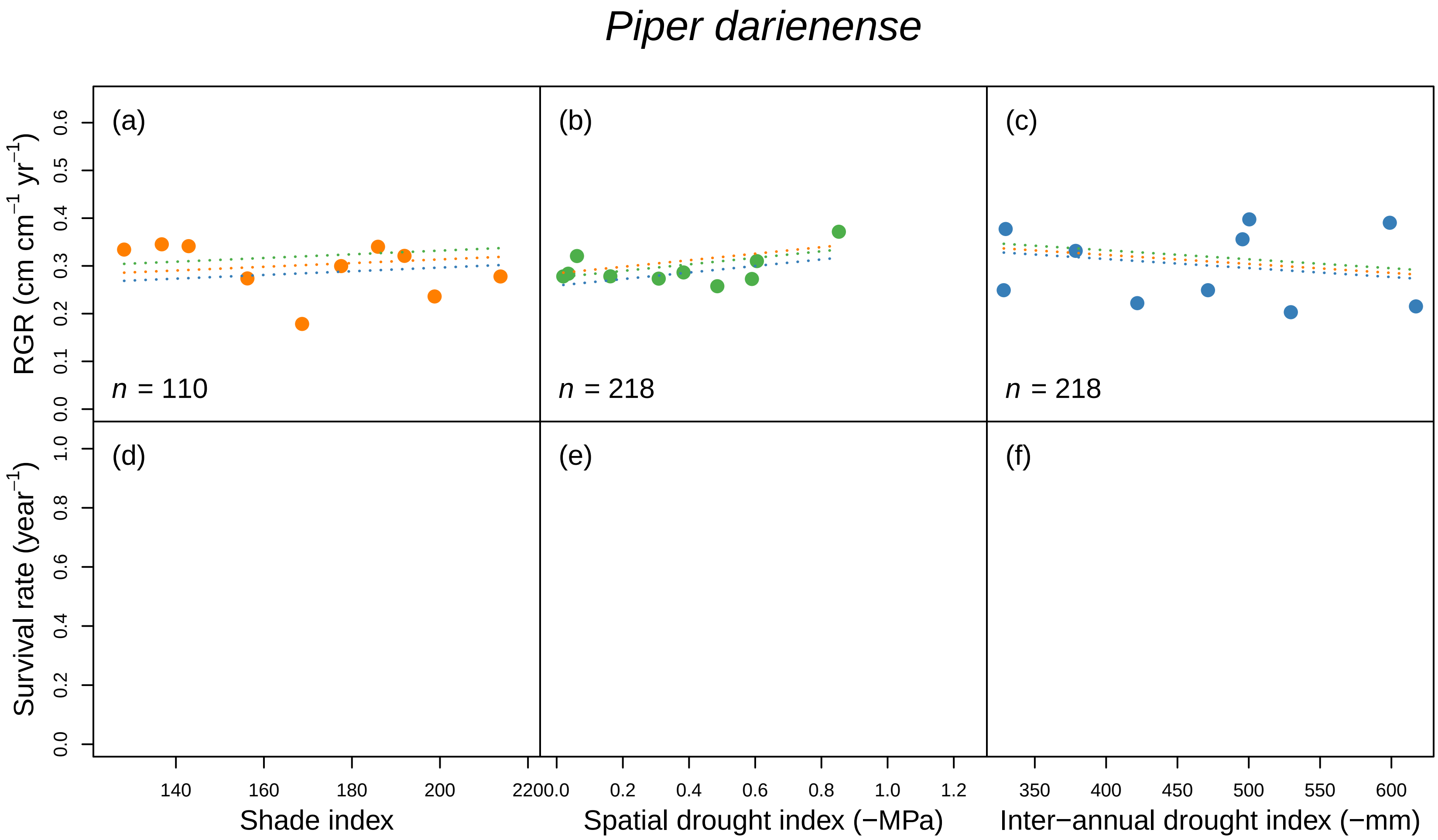

Piper darienense
RGR (cm cm−1 yr−1)
Survival rate (year−1)
(a)
(b)
(c)
(d)
(e)
(f)
n = 110
n = 218
n = 218
0.0
0.1
0.2
0.3
0.4
0.5
0.6
0.0
0.2
0.4
0.6
0.8
1.0
140
160
180
200
220
0.0
0.2
0.4
0.6
0.8
1.0
1.2
350
400
450
500
550
600
Shade index
Spatial drought index (−MPa)
Inter−annual drought index (−mm)

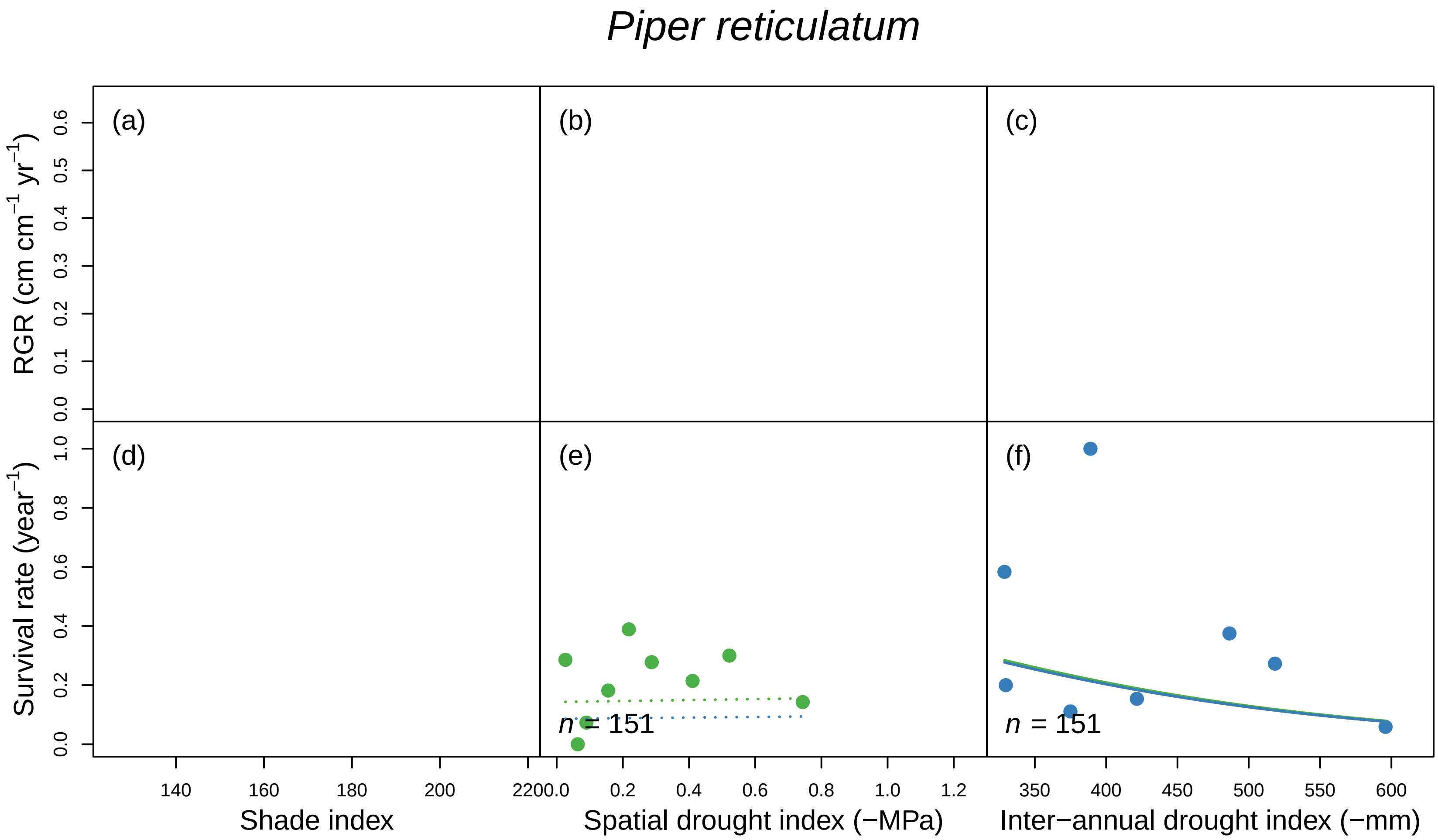

Piper reticulatum
RGR (cm cm−1 yr−1)
Survival rate (year−1)
(a)
(b)
(c)
(d)
(e)
(f)
n = 151
n = 151
0.0 0.1 0.2 0.3 0.4 0.5 0.6
0.0 0.2 0.4 0.6 0.8 1.0
140 160 180 200 220
0.0 0.2 0.4 0.6 0.8 1.0 1.2
350 400 450 500 550 600
Shade index
Spatial drought index (−MPa)
Inter−annual drought index (−mm)

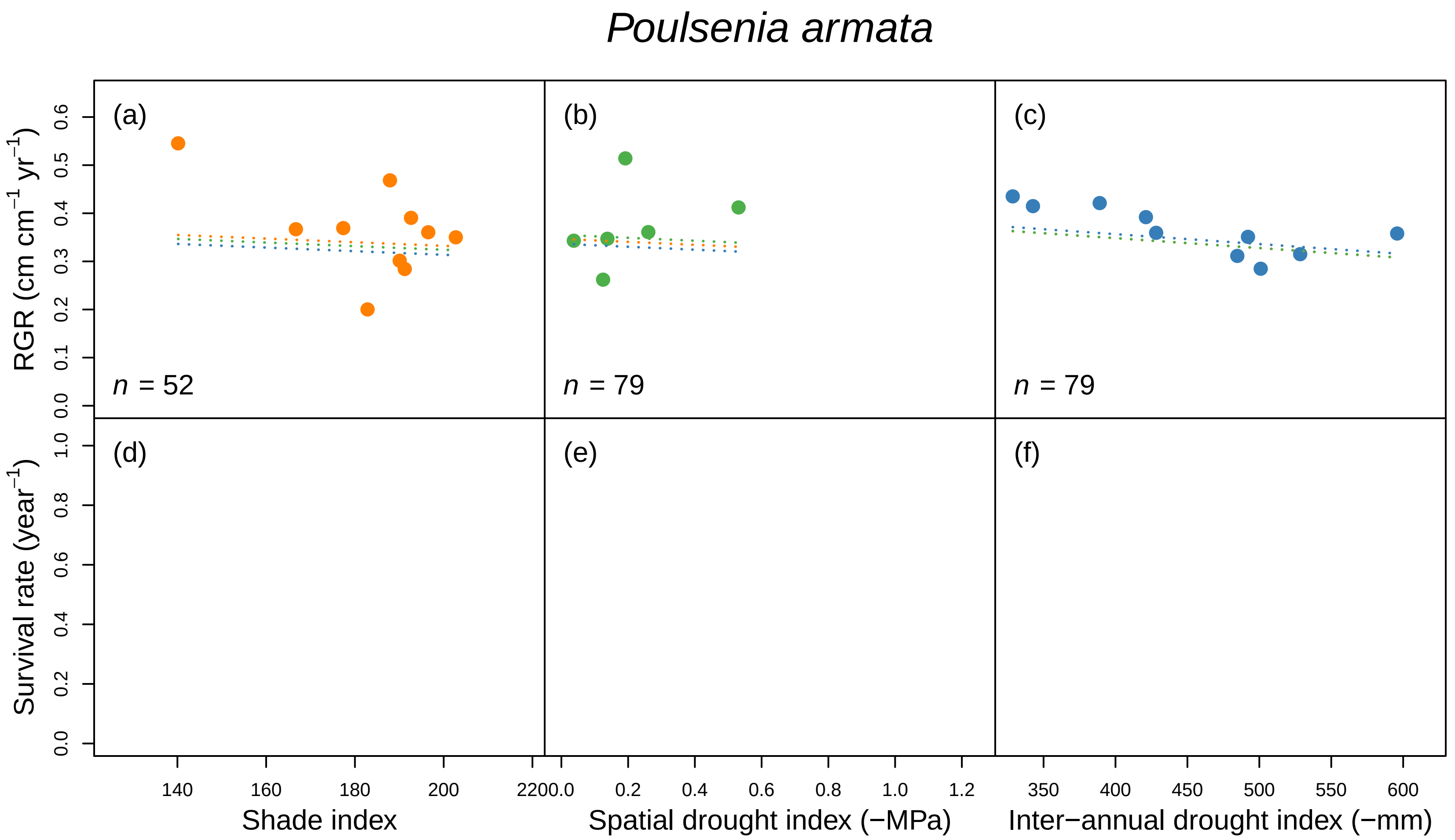

Poulsenia armata
(a)
(b)
(c)
(d)
(e)
(f)
n = 52
n = 79
n = 79
RGR (cm cm−1 yr−1)
Survival rate (year−1)
0.0
0.1
0.2
0.3
0.4
0.5
0.6
0.0
0.2
0.4
0.6
0.8
1.0
140
160
180
200
220
0.0
0.2
0.4
0.6
0.8
1.0
1.2
350
400
450
500
550
600
Shade index
Spatial drought index (−MPa)
Inter−annual drought index (−mm)

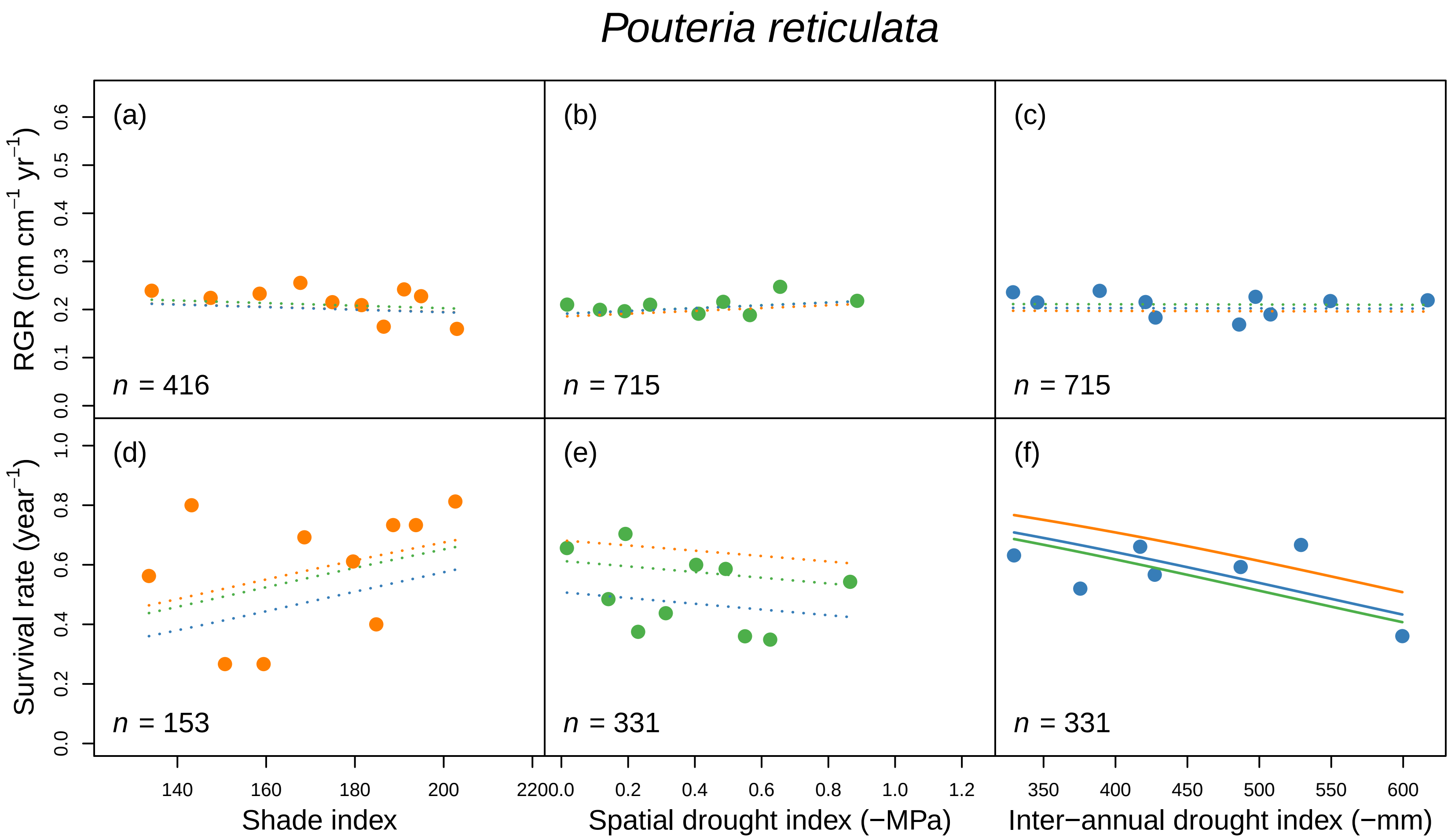
Pouteria reticulata
(a)
(b)
(c)
(d)
(e)
(f)
n = 416
n = 715
n = 715
n = 153
n = 331
n = 331
RGR (cm cm−1 yr−1)
Survival rate (year−1)
Shade index
Spatial drought index (−MPa)
Inter−annual drought index (−mm)

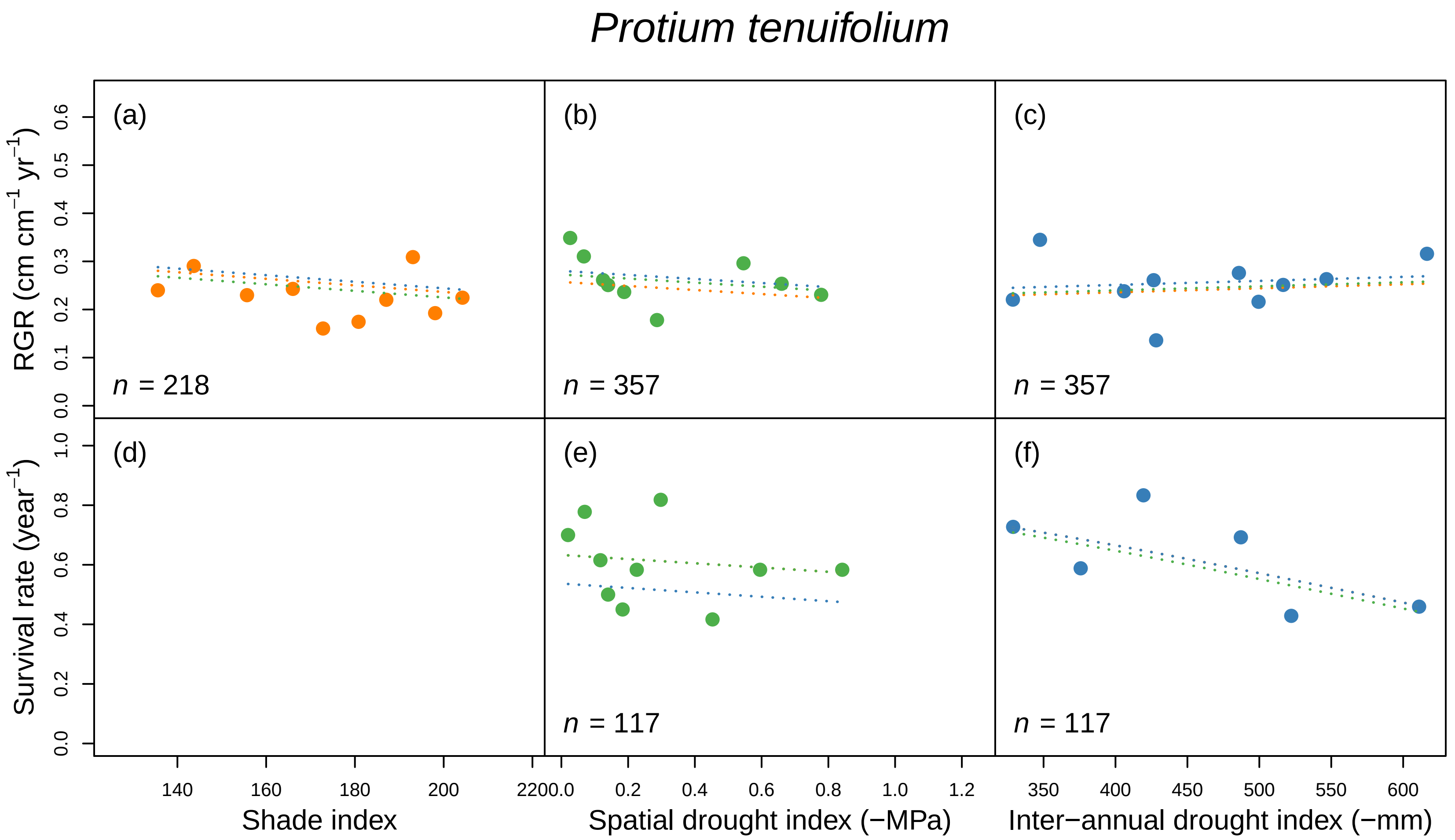
Protium tenuifolium
(a)
(b)
(c)
(d)
(e)
(f)
n = 218
n = 357
n = 357
n = 117
n = 117
RGR (cm cm−1 yr−1)
Survival rate (year−1)
Shade index
Spatial drought index (−MPa)
Inter−annual drought index (−mm)

# *Psychotria acuminata*

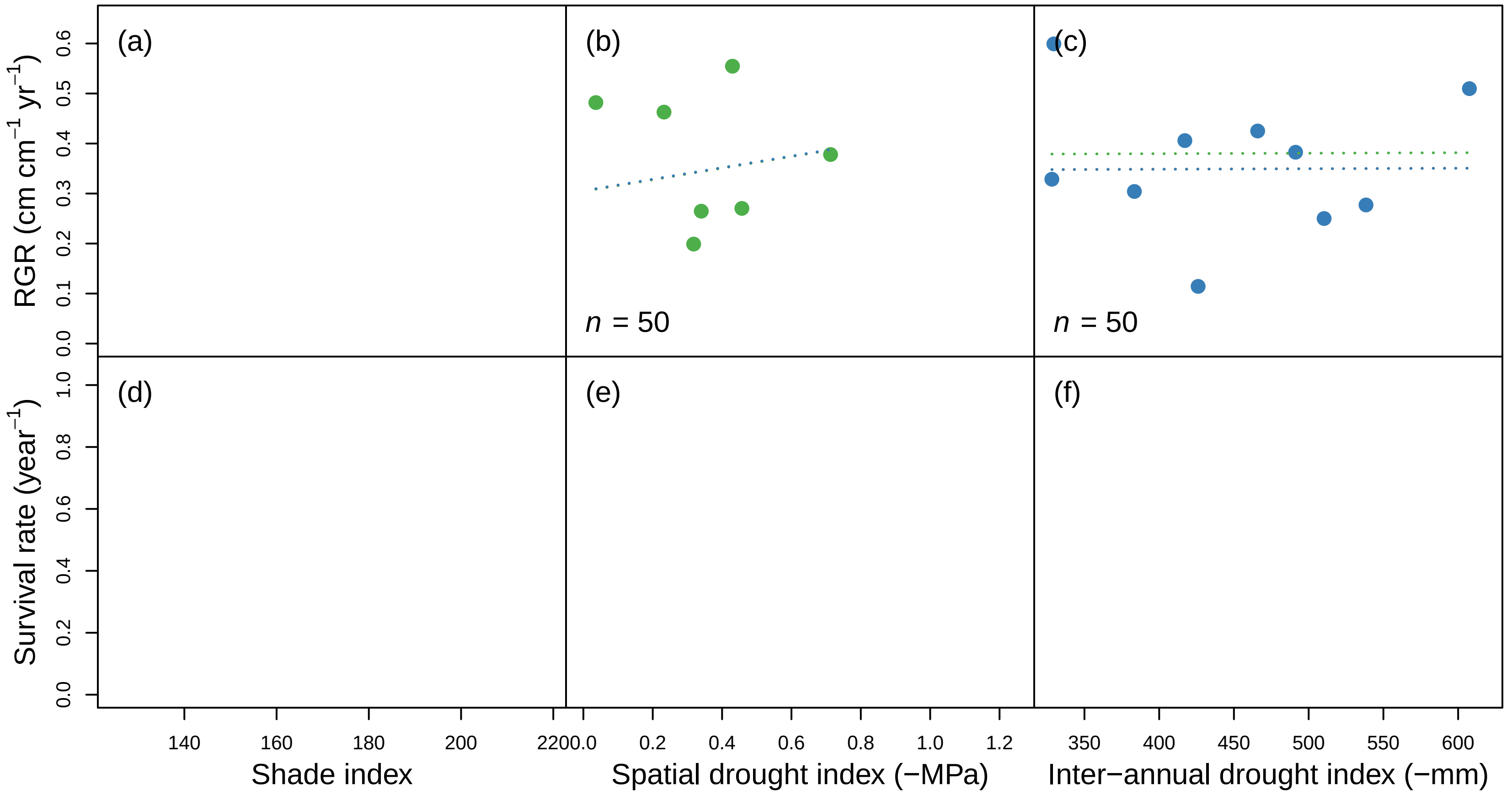

# *Psychotria deflexa*

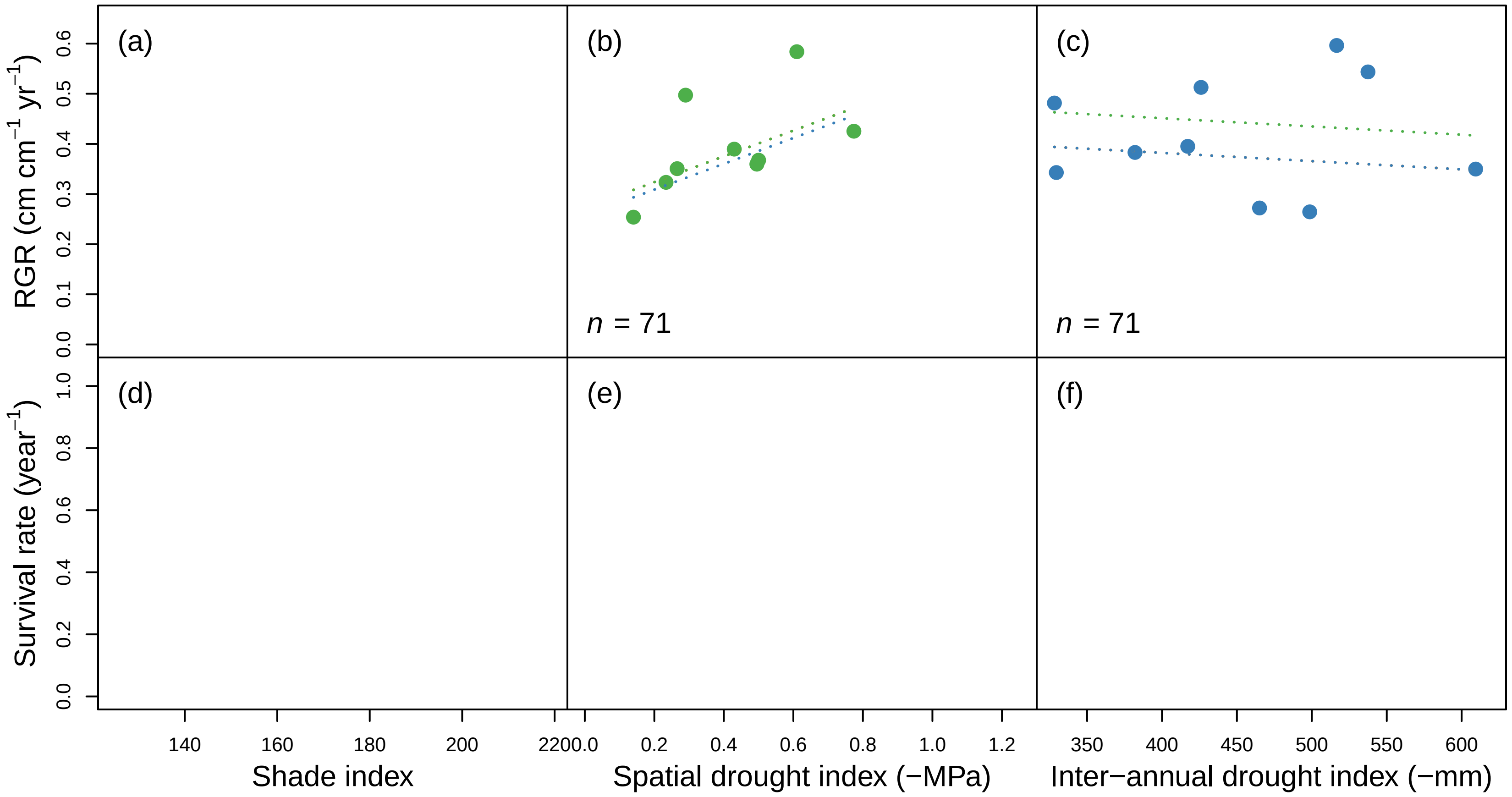

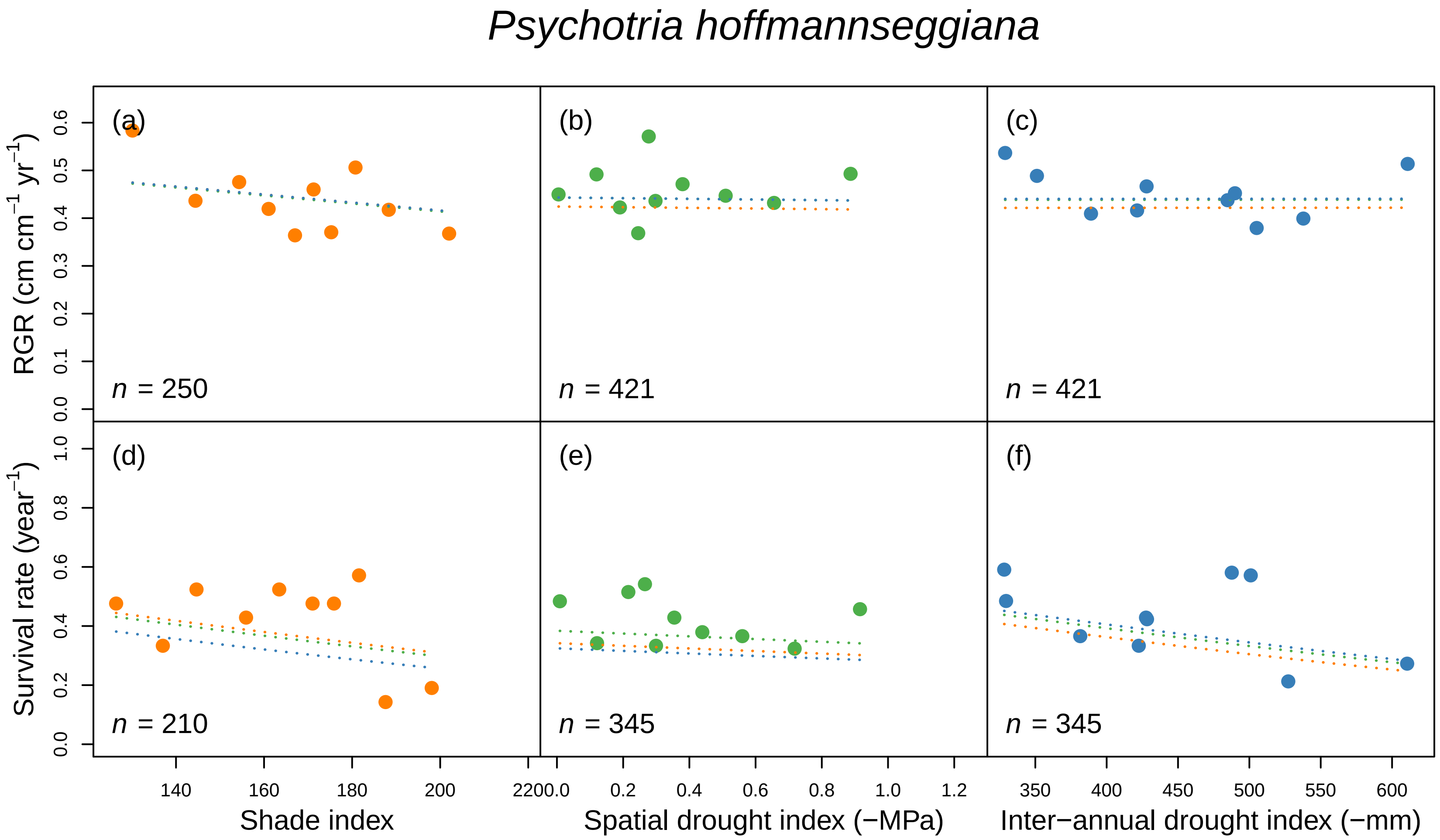

Psychotria hoffmannseggiana
RGR (cm cm−1 yr−1)
Survival rate (year−1)
(a)
(b)
(c)
(d)
(e)
(f)
n = 250
n = 421
n = 421
n = 210
n = 345
n = 345
0.0
0.1
0.2
0.3
0.4
0.5
0.6
0.0
0.2
0.4
0.6
0.8
1.0
140
160
180
200
220
0.0
0.2
0.4
0.6
0.8
1.0
1.2
350
400
450
500
550
600
Shade index
Spatial drought index (−MPa)
Inter−annual drought index (−mm)

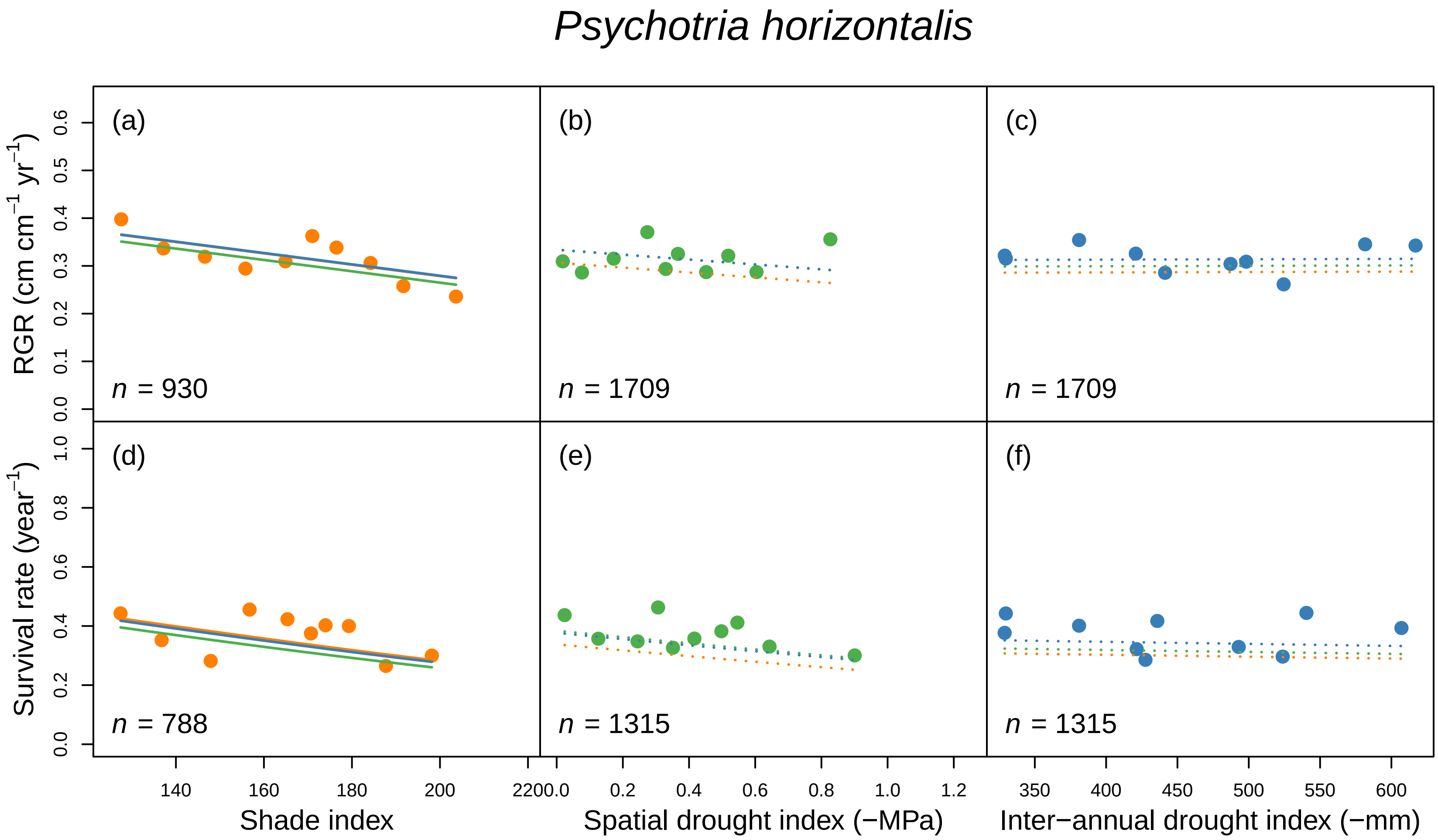
Psychotria horizontalis
RGR (cm cm−1 yr−1)
Survival rate (year−1)
(a)
(b)
(c)
(d)
(e)
(f)
n = 930
n = 1709
n = 1709
n = 788
n = 1315
n = 1315
0.0
0.1
0.2
0.3
0.4
0.5
0.6
0.0
0.2
0.4
0.6
0.8
1.0
140
160
180
200
220
0.0
0.2
0.4
0.6
0.8
1.0
1.2
350
400
450
500
550
600
Shade index
Spatial drought index (−MPa)
Inter−annual drought index (−mm)

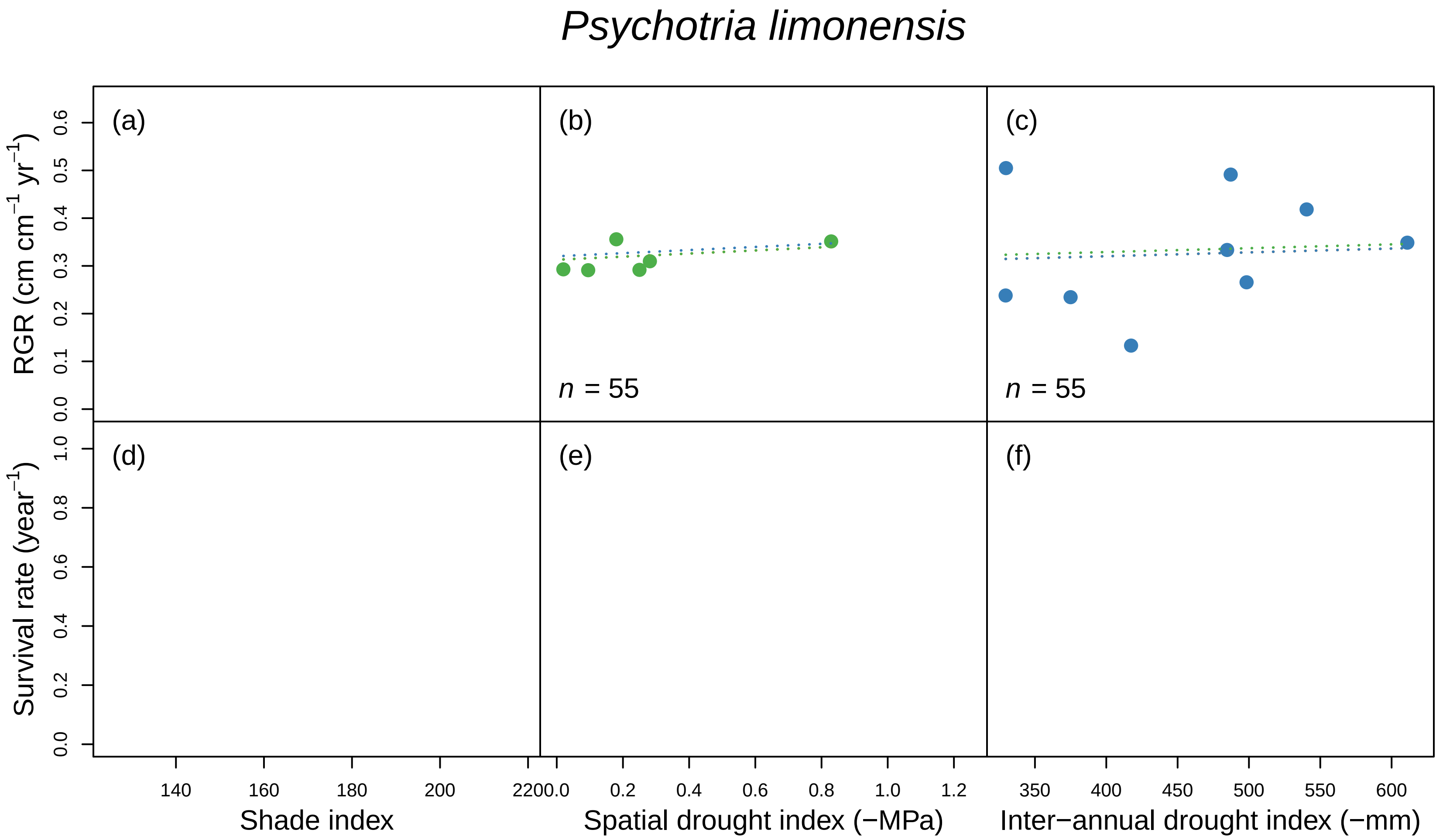

Psychotria limonensis
RGR (cm cm−1 yr−1)
0.0
0.1
0.2
0.3
0.4
0.5
0.6
(a)
(b)
(c)
n = 55
n = 55
Survival rate (year−1)
0.0
0.2
0.4
0.6
0.8
1.0
(d)
(e)
(f)
140
160
180
200
220
0.0
0.2
0.4
0.6
0.8
1.0
1.2
350
400
450
500
550
600
Shade index
Spatial drought index (−MPa)
Inter−annual drought index (−mm)

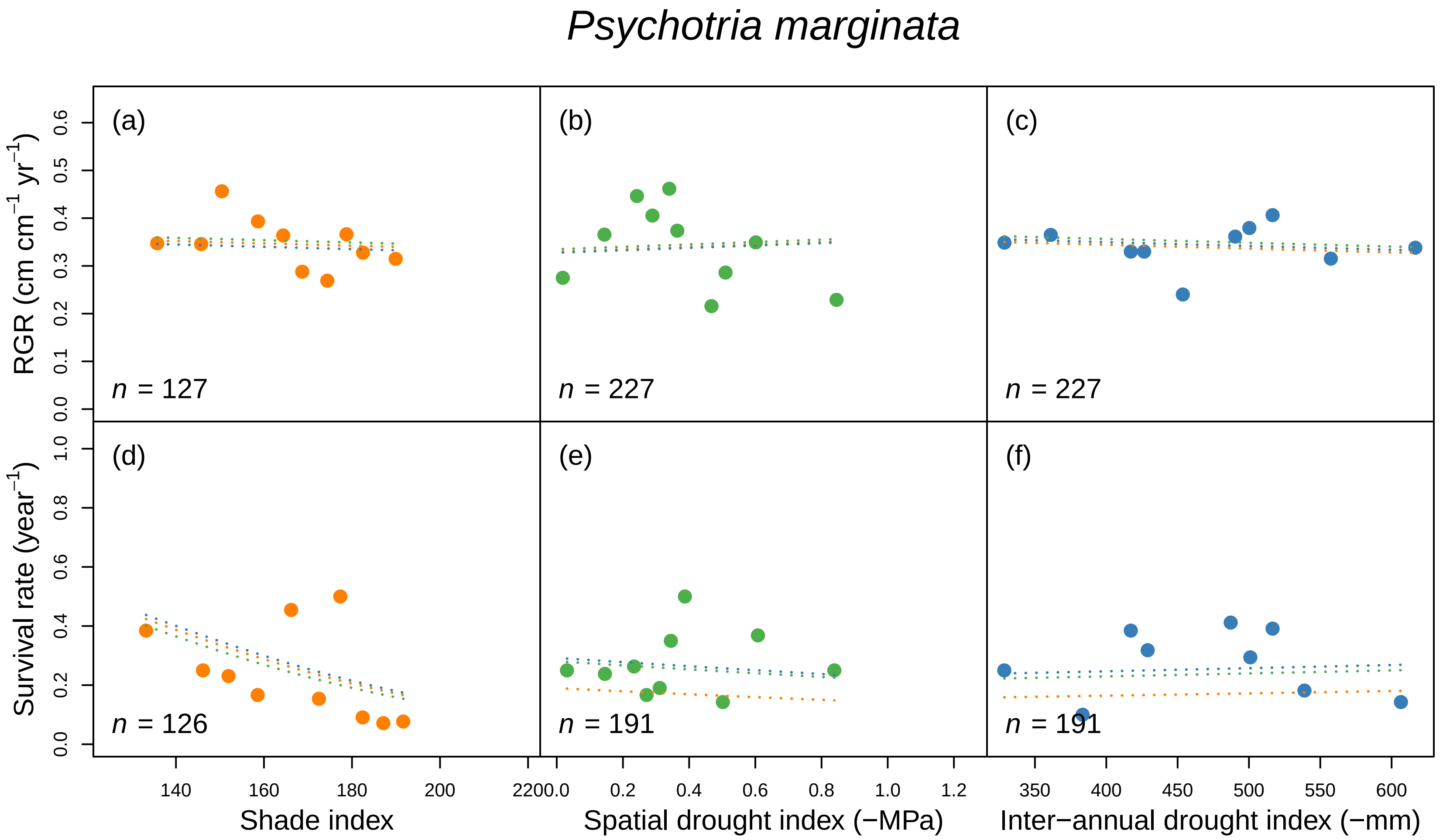

Psychotria marginata
RGR (cm cm$^{-1}$ yr$^{-1}$)
Survival rate (year$^{-1}$)
(a)
(b)
(c)
(d)
(e)
(f)
n = 127
n = 227
n = 227
n = 126
n = 191
n = 191
0.0 0.1 0.2 0.3 0.4 0.5 0.6
0.0 0.2 0.4 0.6 0.8 1.0
140 160 180 200 220
0.0 0.2 0.4 0.6 0.8 1.0 1.2
350 400 450 500 550 600
Shade index
Spatial drought index (−MPa)
Inter−annual drought index (−mm)

# *Psychotria racemosa*

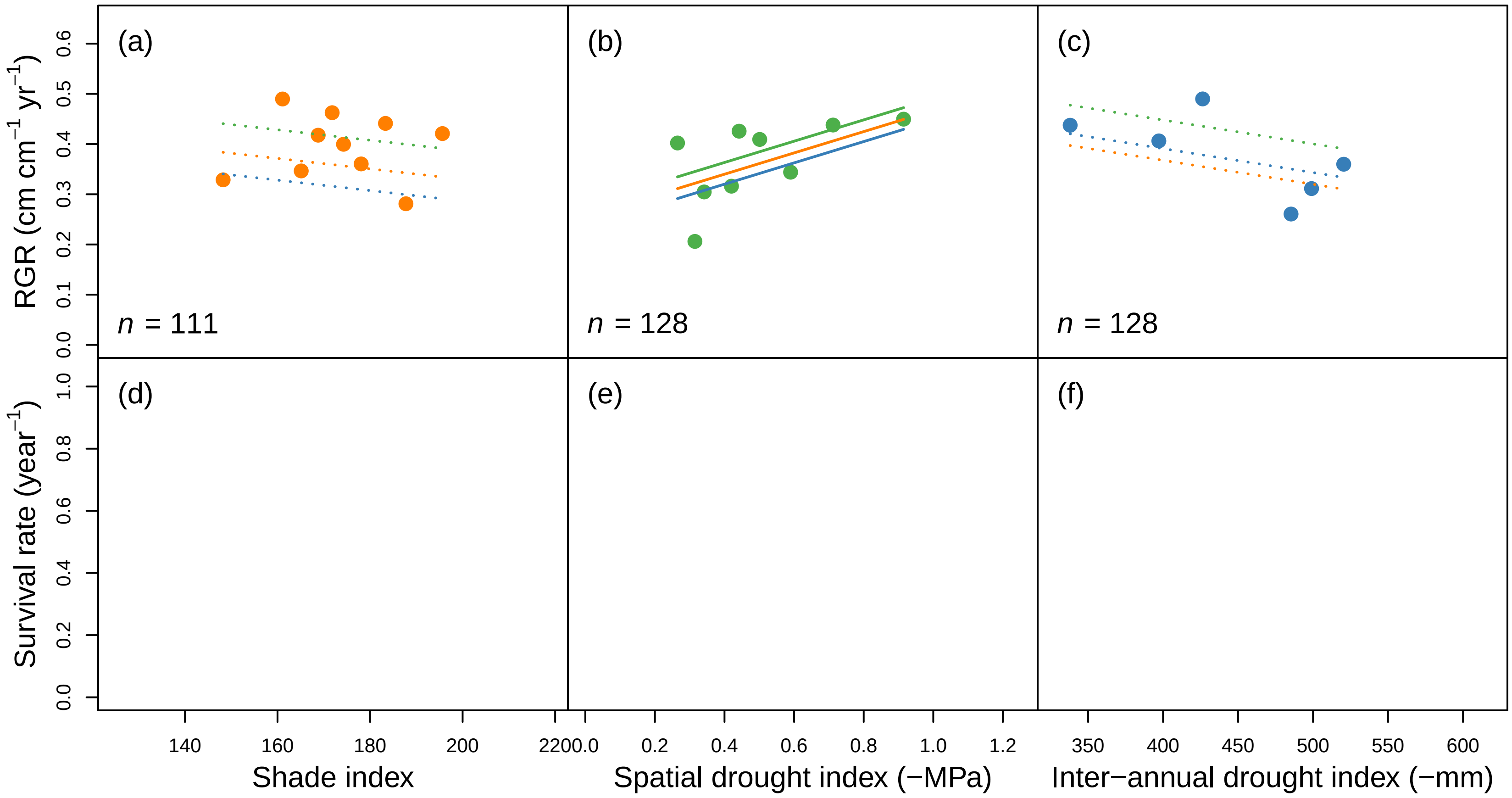

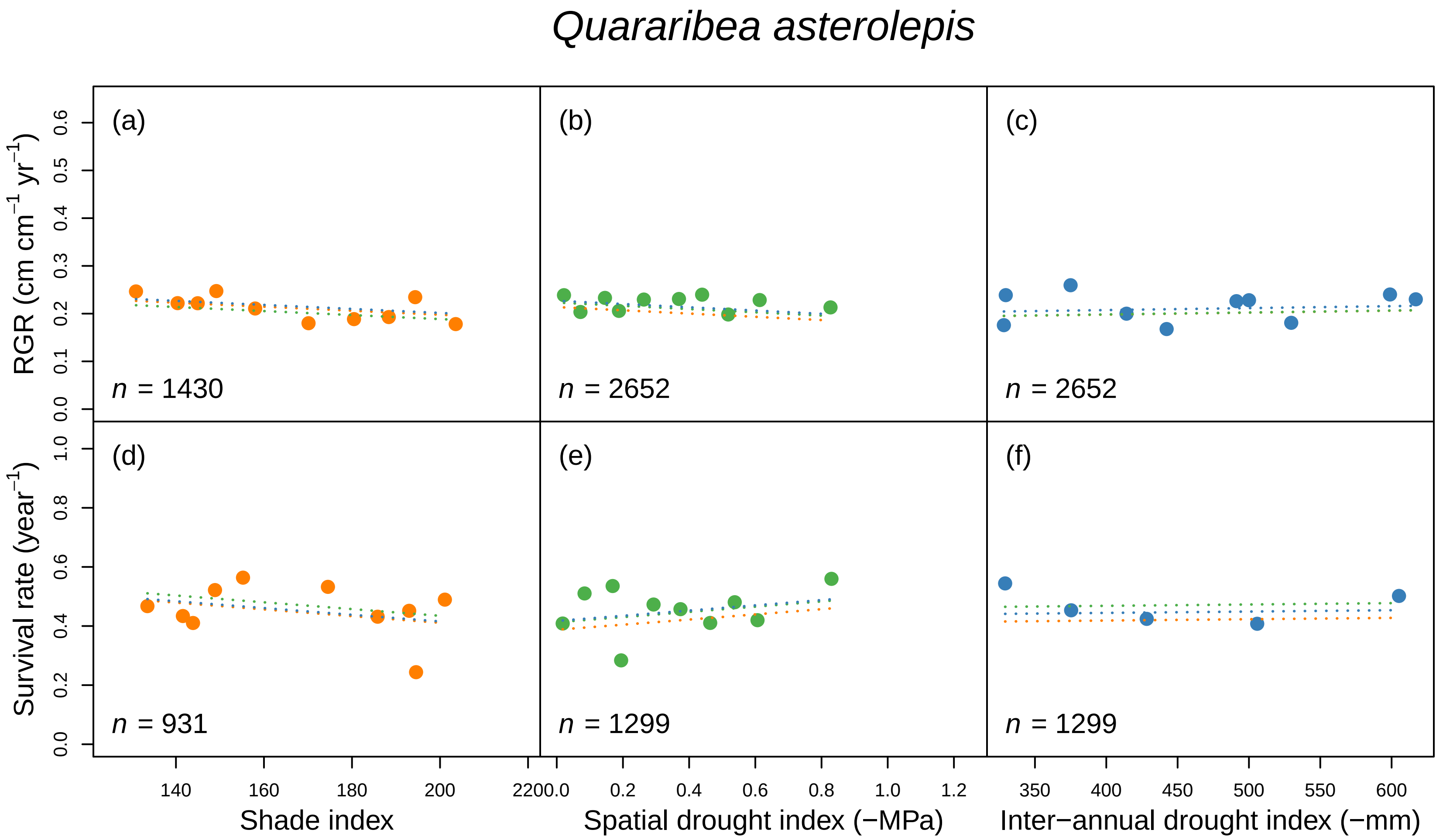
Quararibea asterolepis
RGR (cm cm−1 yr−1)
Survival rate (year−1)
(a)
(b)
(c)
(d)
(e)
(f)
n = 1430
n = 2652
n = 2652
n = 931
n = 1299
n = 1299
0.0
0.1
0.2
0.3
0.4
0.5
0.6
0.0
0.2
0.4
0.6
0.8
1.0
140
160
180
200
220
0.0
0.2
0.4
0.6
0.8
1.0
1.2
350
400
450
500
550
600
Shade index
Spatial drought index (−MPa)
Inter−annual drought index (−mm)

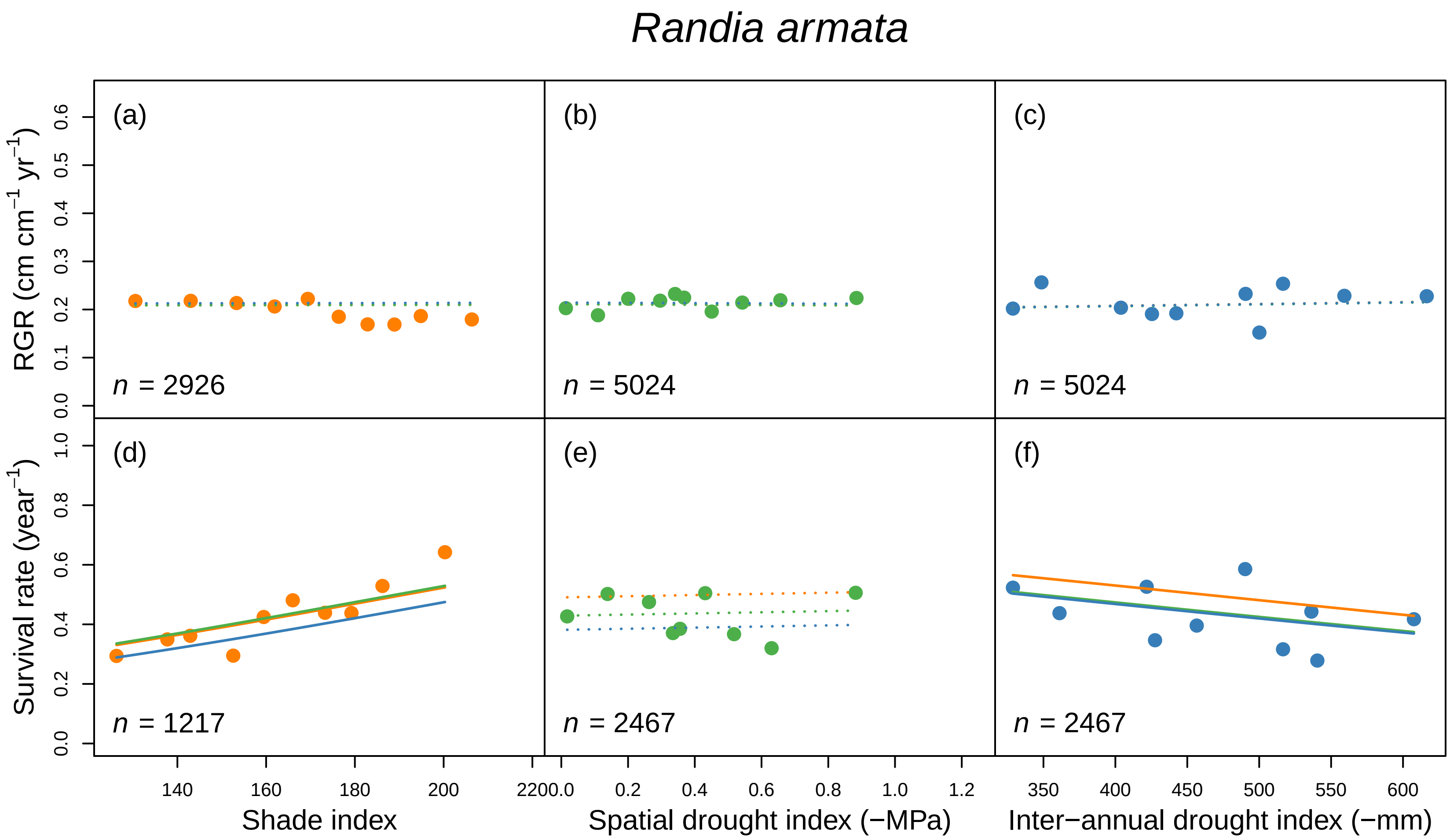

Randia armata
(a)
(b)
(c)
(d)
(e)
(f)
n = 2926
n = 5024
n = 5024
n = 1217
n = 2467
n = 2467
RGR (cm cm−1 yr−1)
Survival rate (year−1)
0.0
0.1
0.2
0.3
0.4
0.5
0.6
0.0
0.2
0.4
0.6
0.8
1.0
140
160
180
200
220
0.0
0.2
0.4
0.6
0.8
1.0
1.2
350
400
450
500
550
600
Shade index
Spatial drought index (−MPa)
Inter−annual drought index (−mm)

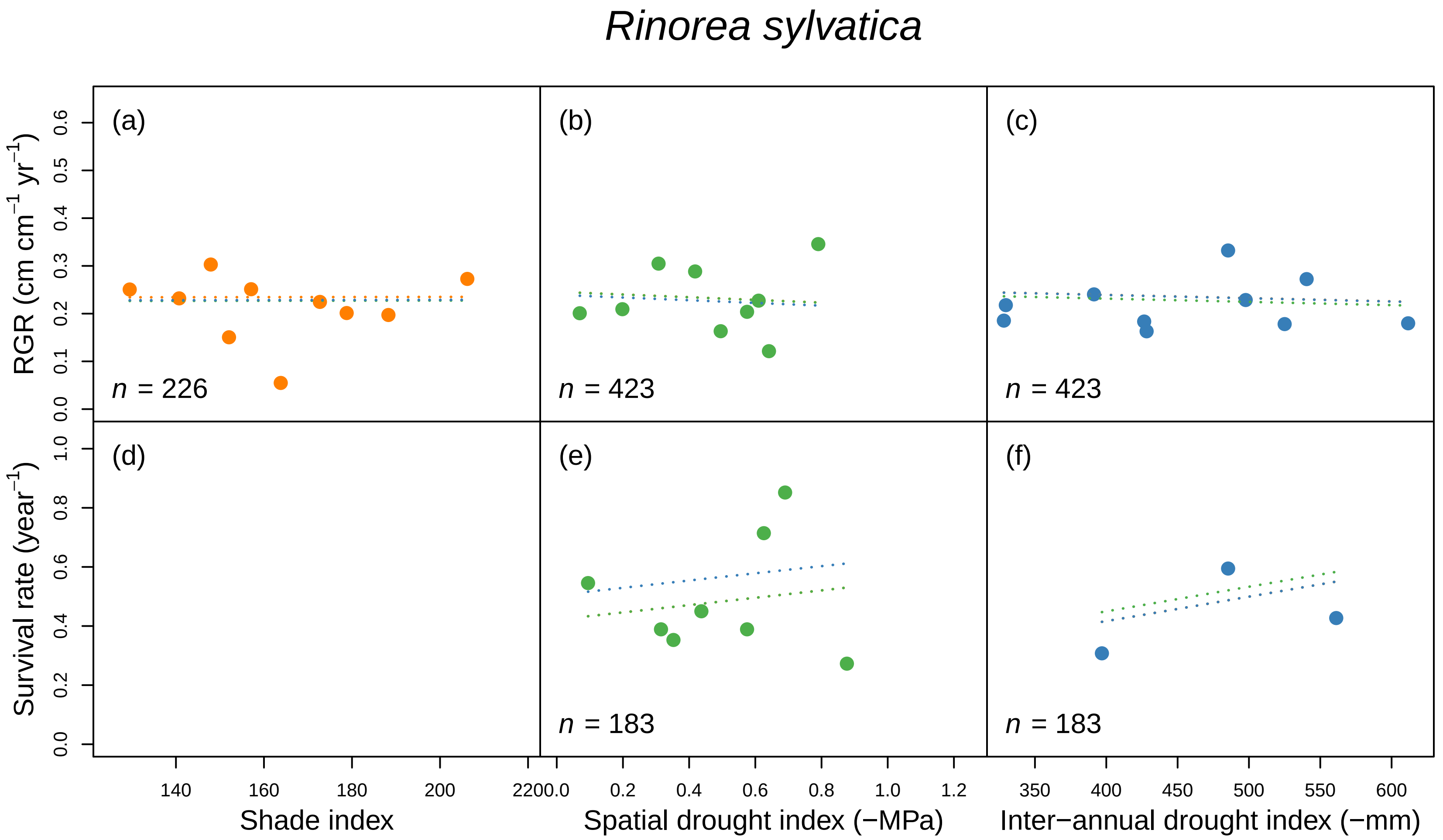
Rinorea sylvatica
(a)
(b)
(c)
(d)
(e)
(f)
n = 226
n = 423
n = 423
n = 183
n = 183
RGR (cm cm−1 yr−1)
Survival rate (year−1)
Shade index
Spatial drought index (−MPa)
Inter−annual drought index (−mm)

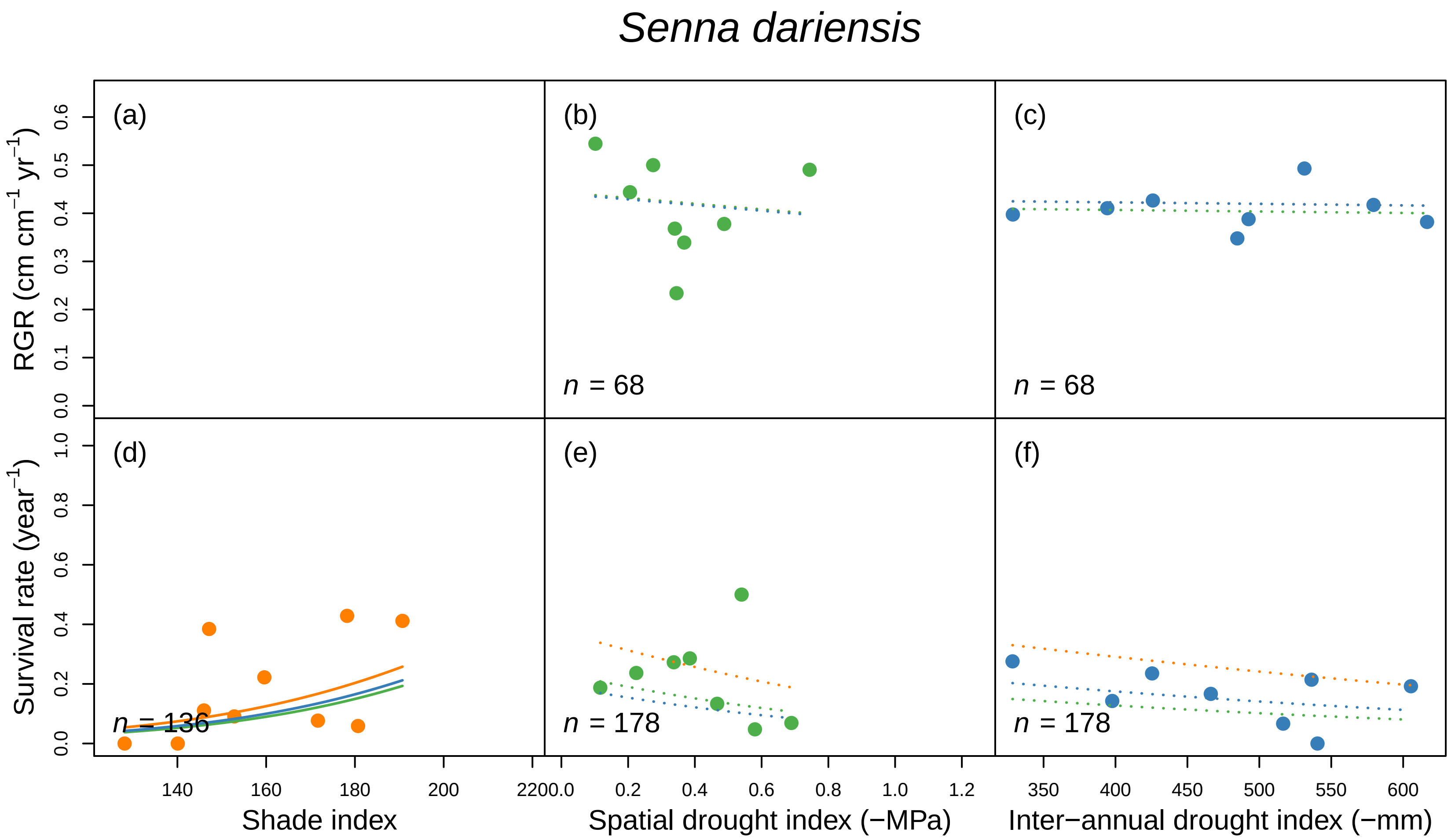
Senna dariensis
(a)
(b)
(c)
(d)
(e)
(f)
RGR (cm cm−1 yr−1)
Survival rate (year−1)
n = 68
n = 68
n = 136
n = 178
n = 178
Shade index
Spatial drought index (−MPa)
Inter−annual drought index (−mm)

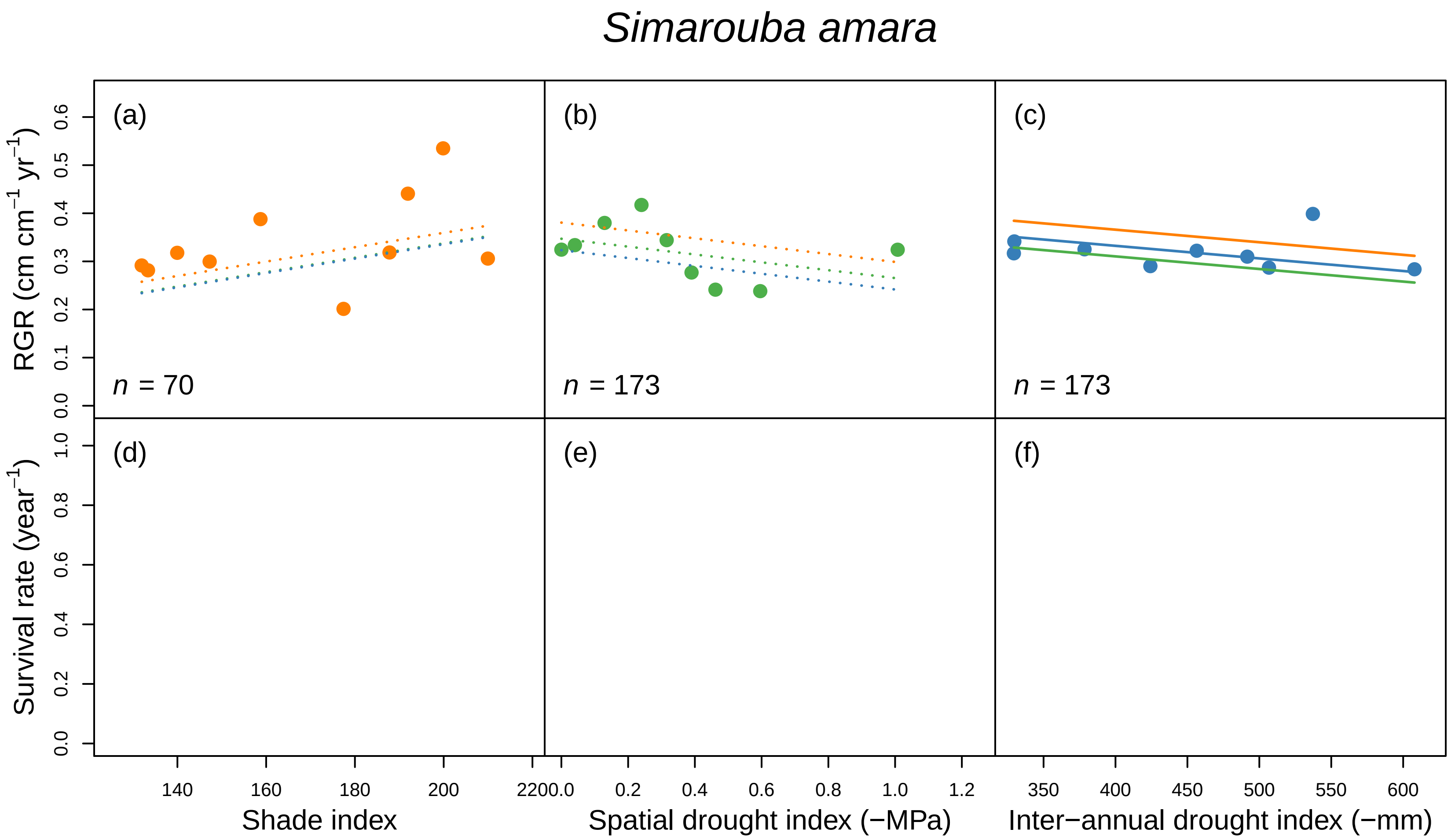

Simarouba amara
(a)
(b)
(c)
(d)
(e)
(f)
RGR (cm cm−1 yr−1)
Survival rate (year−1)
n = 70
n = 173
n = 173
0.0
0.1
0.2
0.3
0.4
0.5
0.6
0.0
0.2
0.4
0.6
0.8
1.0
140
160
180
200
220
0.0
0.2
0.4
0.6
0.8
1.0
1.2
350
400
450
500
550
600
Shade index
Spatial drought index (−MPa)
Inter−annual drought index (−mm)

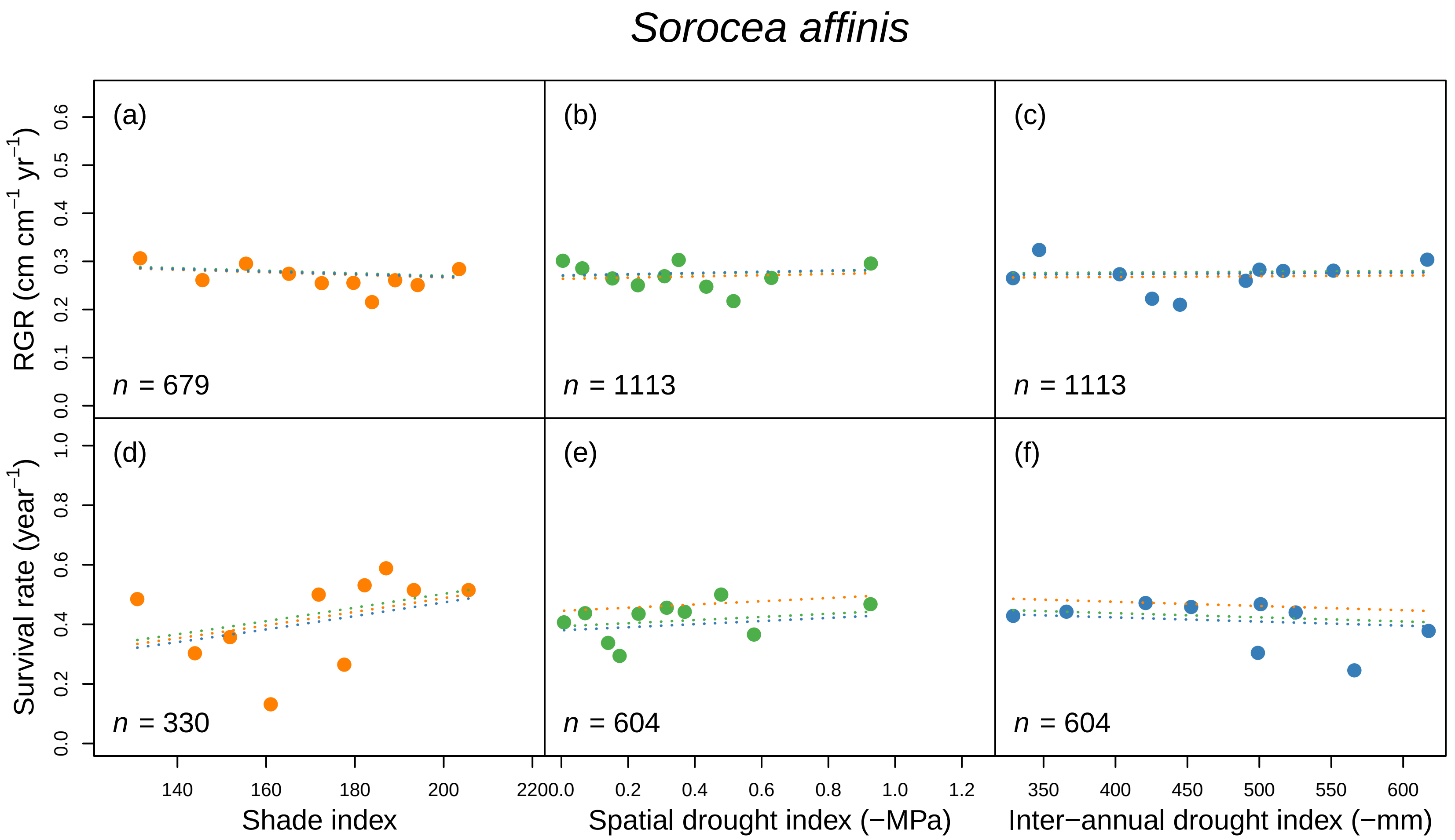

Sorocea affinis
(a)
(b)
(c)
(d)
(e)
(f)
n = 679
n = 1113
n = 1113
n = 330
n = 604
n = 604
RGR (cm cm$^{-1}$ yr$^{-1}$)
Survival rate (year$^{-1}$)
0.0
0.1
0.2
0.3
0.4
0.5
0.6
0.0
0.2
0.4
0.6
0.8
1.0
140
160
180
200
220
0.0
0.2
0.4
0.6
0.8
1.0
1.2
350
400
450
500
550
600
Shade index
Spatial drought index (−MPa)
Inter−annual drought index (−mm)

# *Strychnos brachistantha*

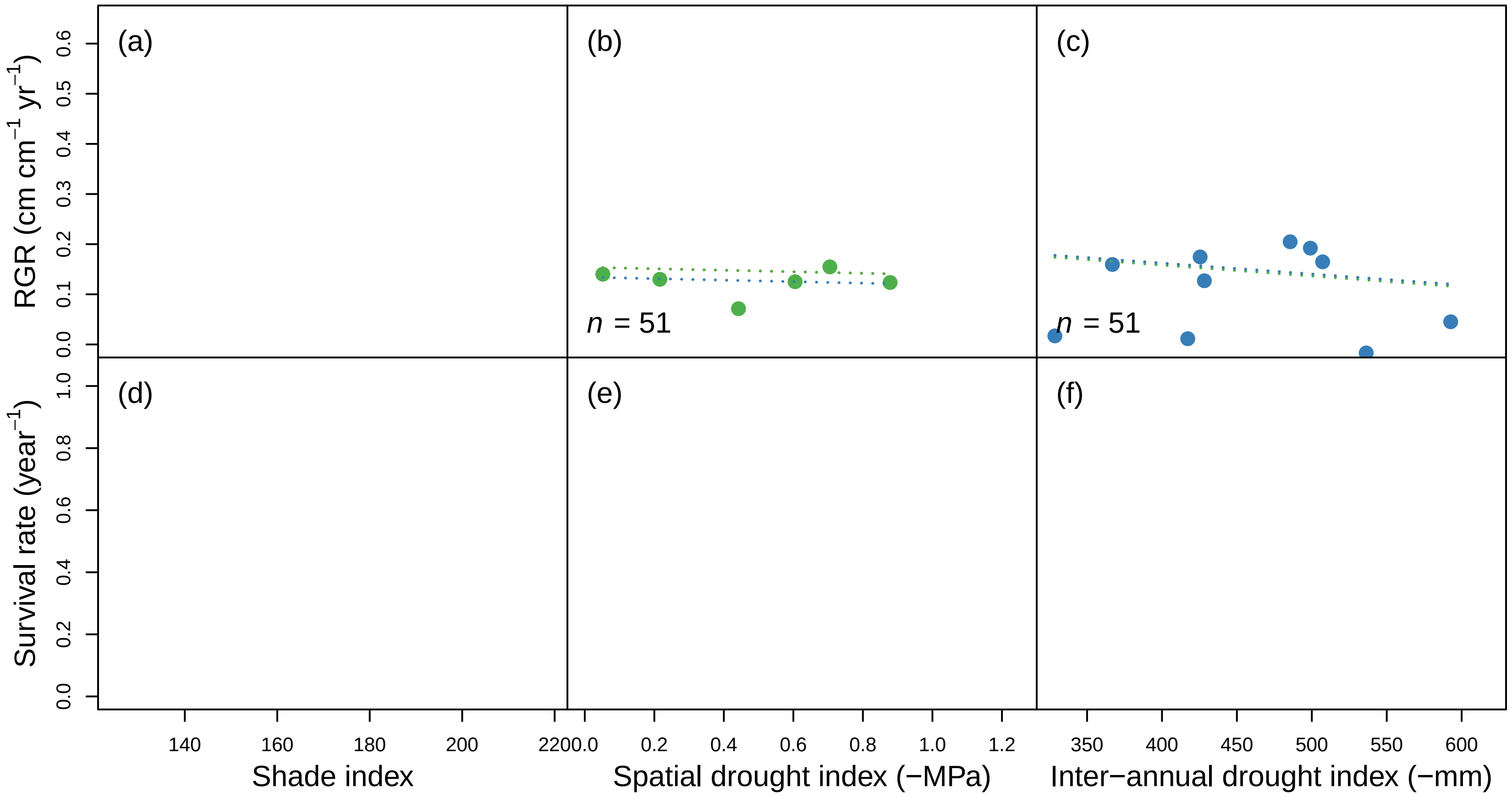

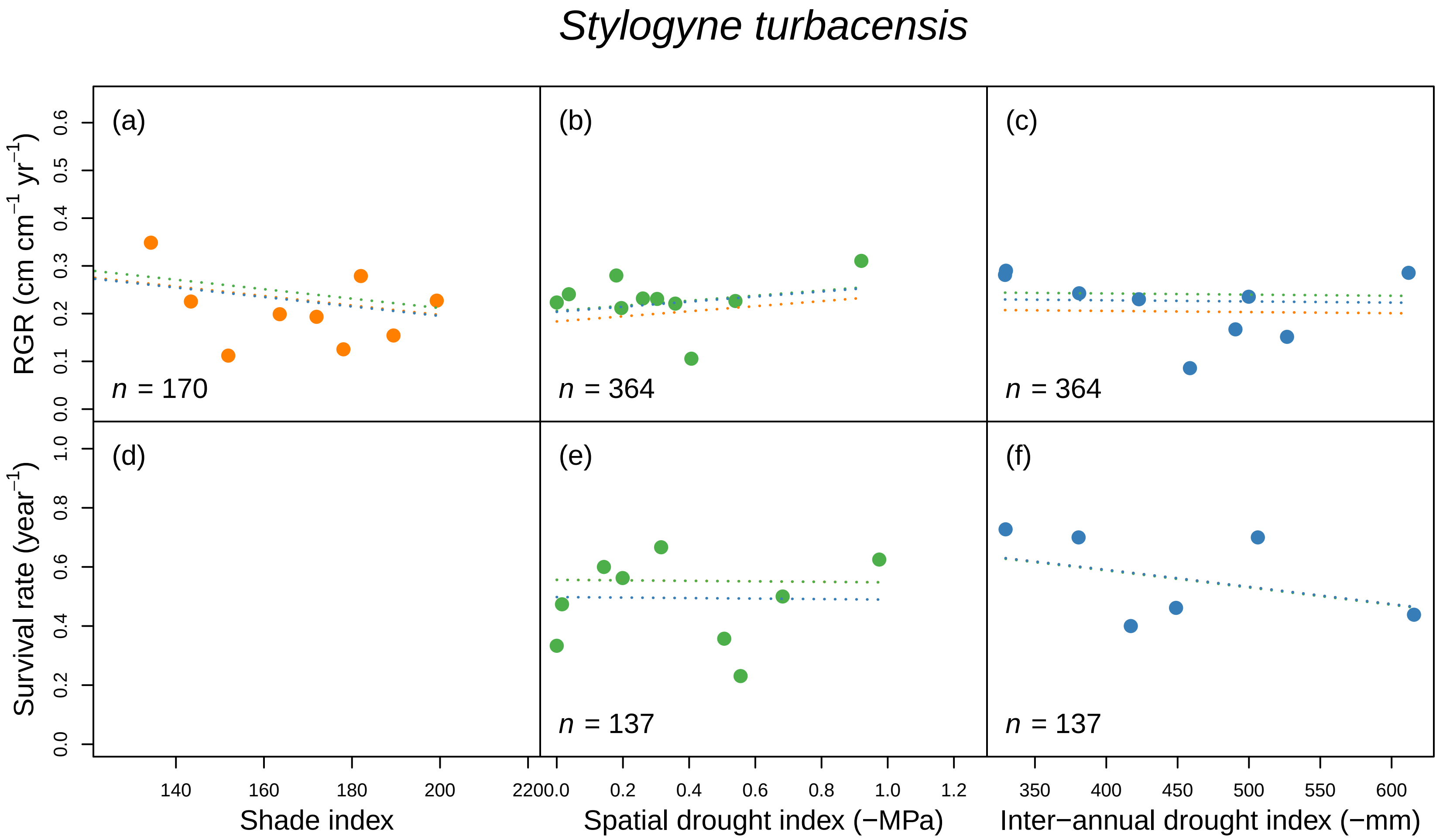
Stylogyne turbacensis
(a)
(b)
(c)
(d)
(e)
(f)
n = 170
n = 364
n = 364
n = 137
n = 137
RGR (cm cm−1 yr−1)
Survival rate (year−1)
0.0
0.1
0.2
0.3
0.4
0.5
0.6
0.0
0.2
0.4
0.6
0.8
1.0
140
160
180
200
220
0.0
0.2
0.4
0.6
0.8
1.0
1.2
350
400
450
500
550
600
Shade index
Spatial drought index (−MPa)
Inter−annual drought index (−mm)

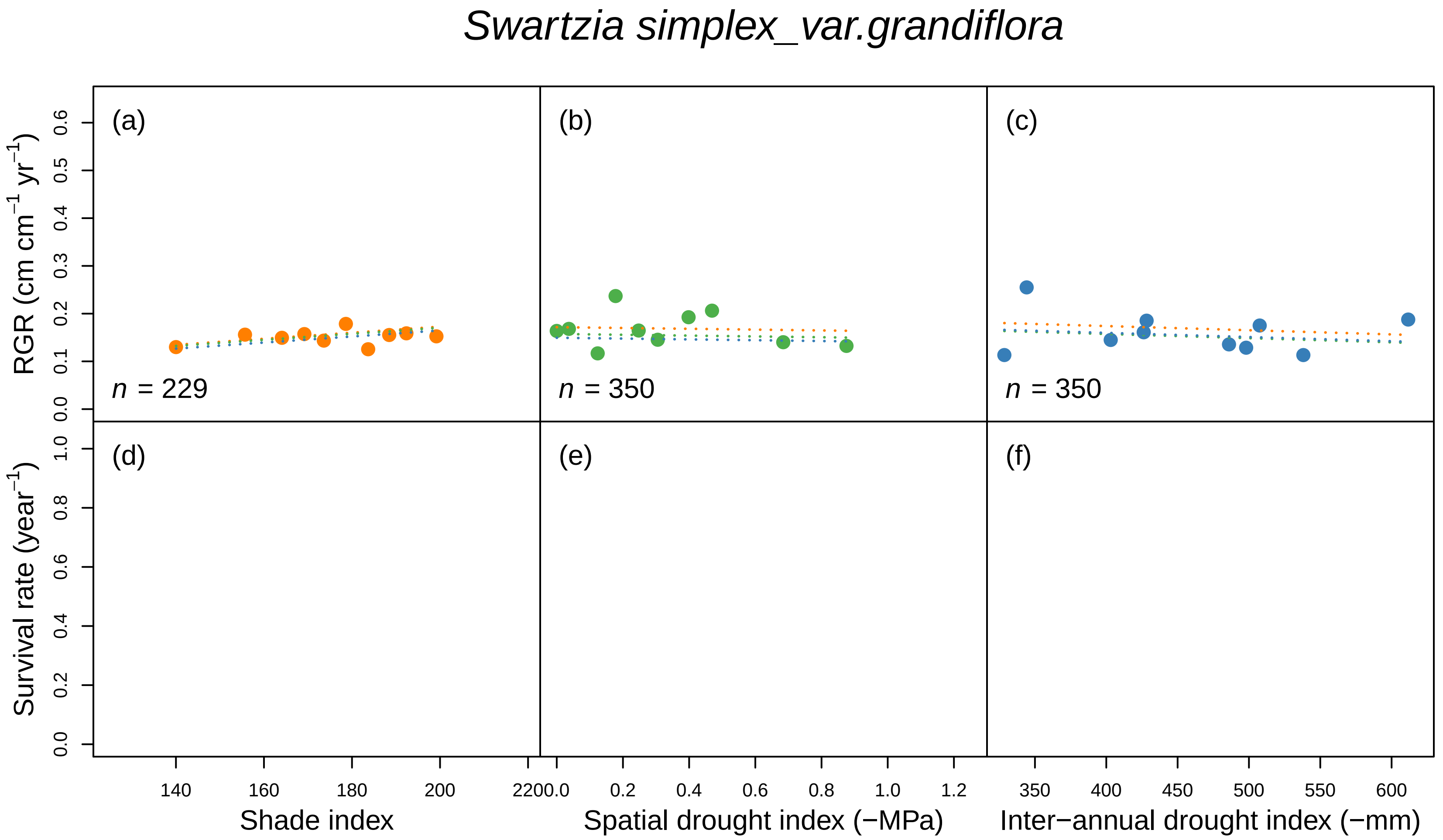

Swartzia simplex_var.grandiflora
(a)
(b)
(c)
(d)
(e)
(f)
n = 229
n = 350
n = 350
RGR (cm cm−1 yr−1)
Survival rate (year−1)
0.0
0.1
0.2
0.3
0.4
0.5
0.6
0.0
0.2
0.4
0.6
0.8
1.0
140
160
180
200
220
0.0
0.2
0.4
0.6
0.8
1.0
1.2
350
400
450
500
550
600
Shade index
Spatial drought index (−MPa)
Inter−annual drought index (−mm)

# *Swartzia simplex_var.ochnacea*

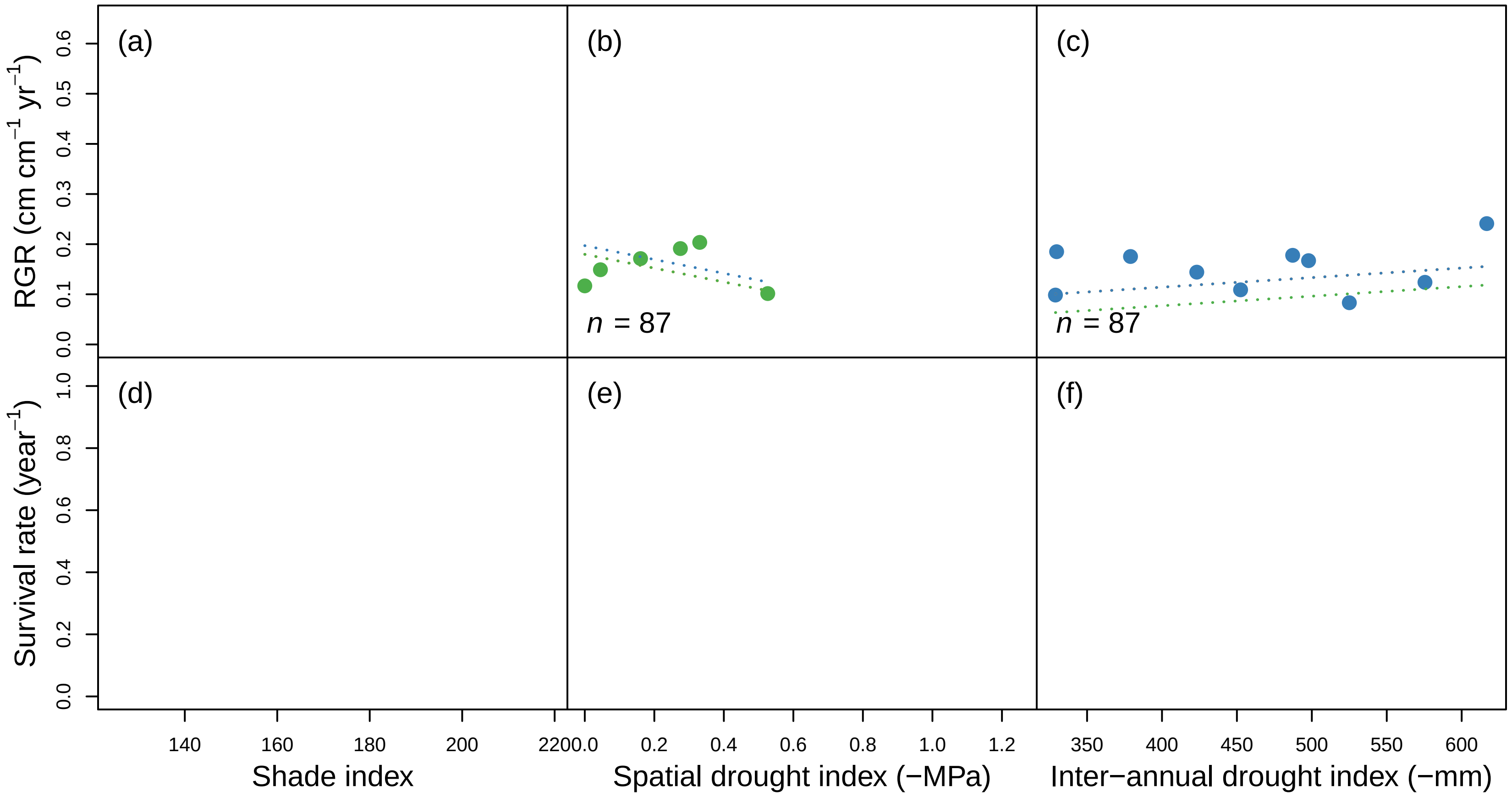

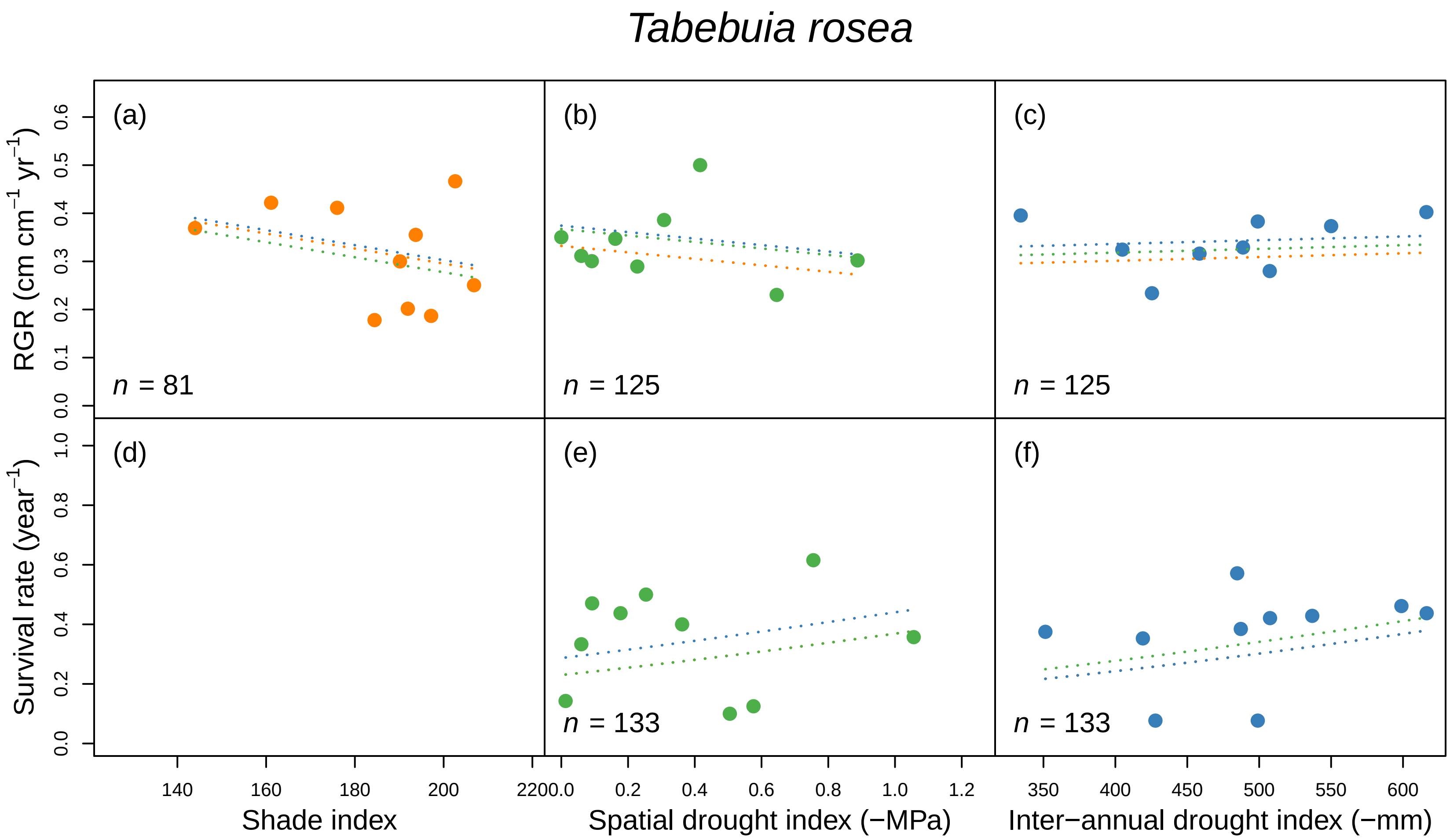
Tabebuia rosea
(a)
(b)
(c)
(d)
(e)
(f)
n = 81
n = 125
n = 125
n = 133
n = 133
RGR (cm cm−1 yr−1)
Survival rate (year−1)
0.0
0.1
0.2
0.3
0.4
0.5
0.6
0.0
0.2
0.4
0.6
0.8
1.0
140
160
180
200
220
0.0
0.2
0.4
0.6
0.8
1.0
1.2
350
400
450
500
550
600
Shade index
Spatial drought index (−MPa)
Inter−annual drought index (−mm)

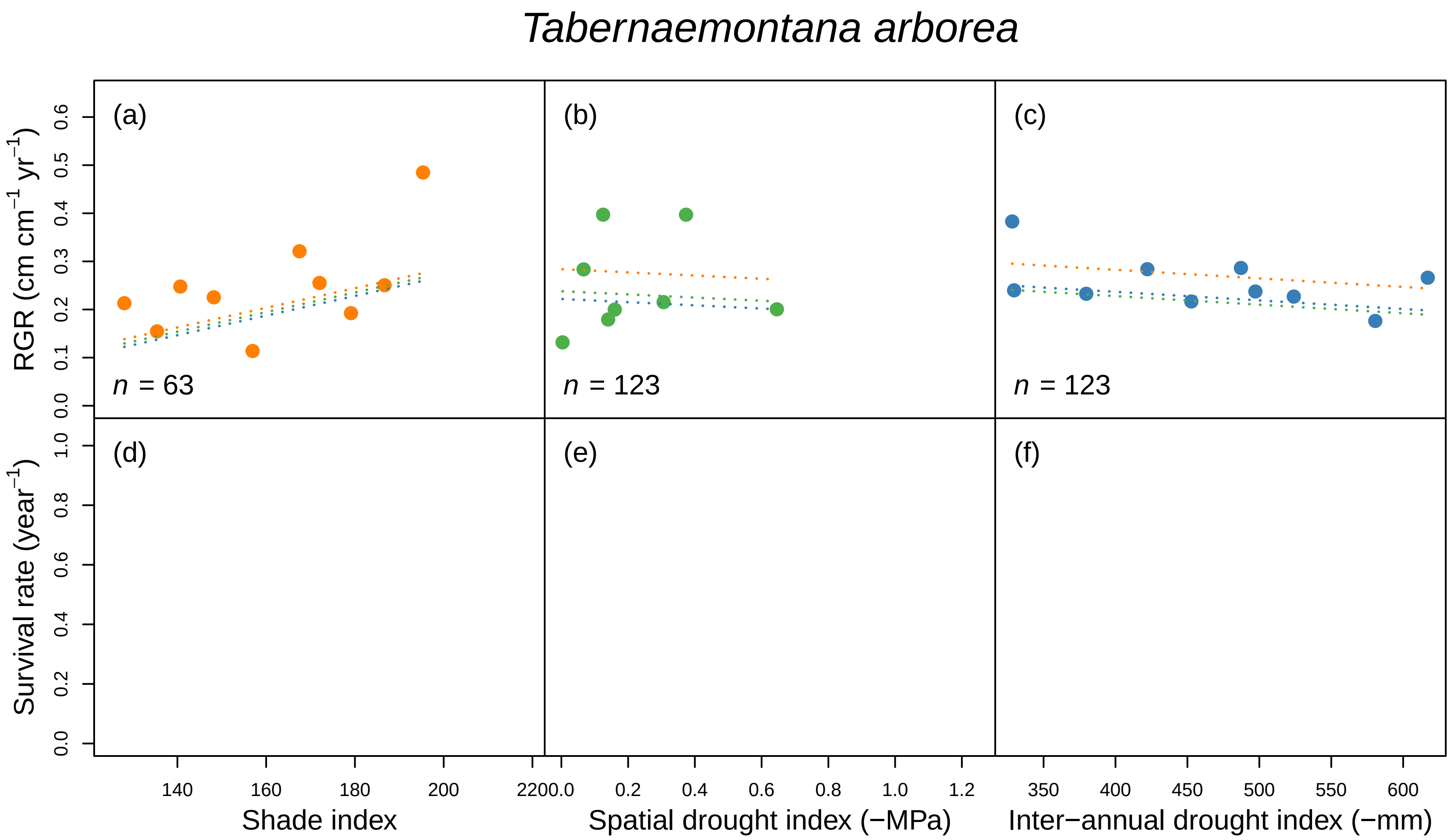
Tabernaemontana arborea
(a)
(b)
(c)
(d)
(e)
(f)
n = 63
n = 123
n = 123
RGR (cm cm−1 yr−1)
Survival rate (year−1)
0.0
0.1
0.2
0.3
0.4
0.5
0.6
0.0
0.2
0.4
0.6
0.8
1.0
140
160
180
200
220
0.0
0.2
0.4
0.6
0.8
1.0
1.2
350
400
450
500
550
600
Shade index
Spatial drought index (−MPa)
Inter−annual drought index (−mm)

# *Tachigali versicolor*

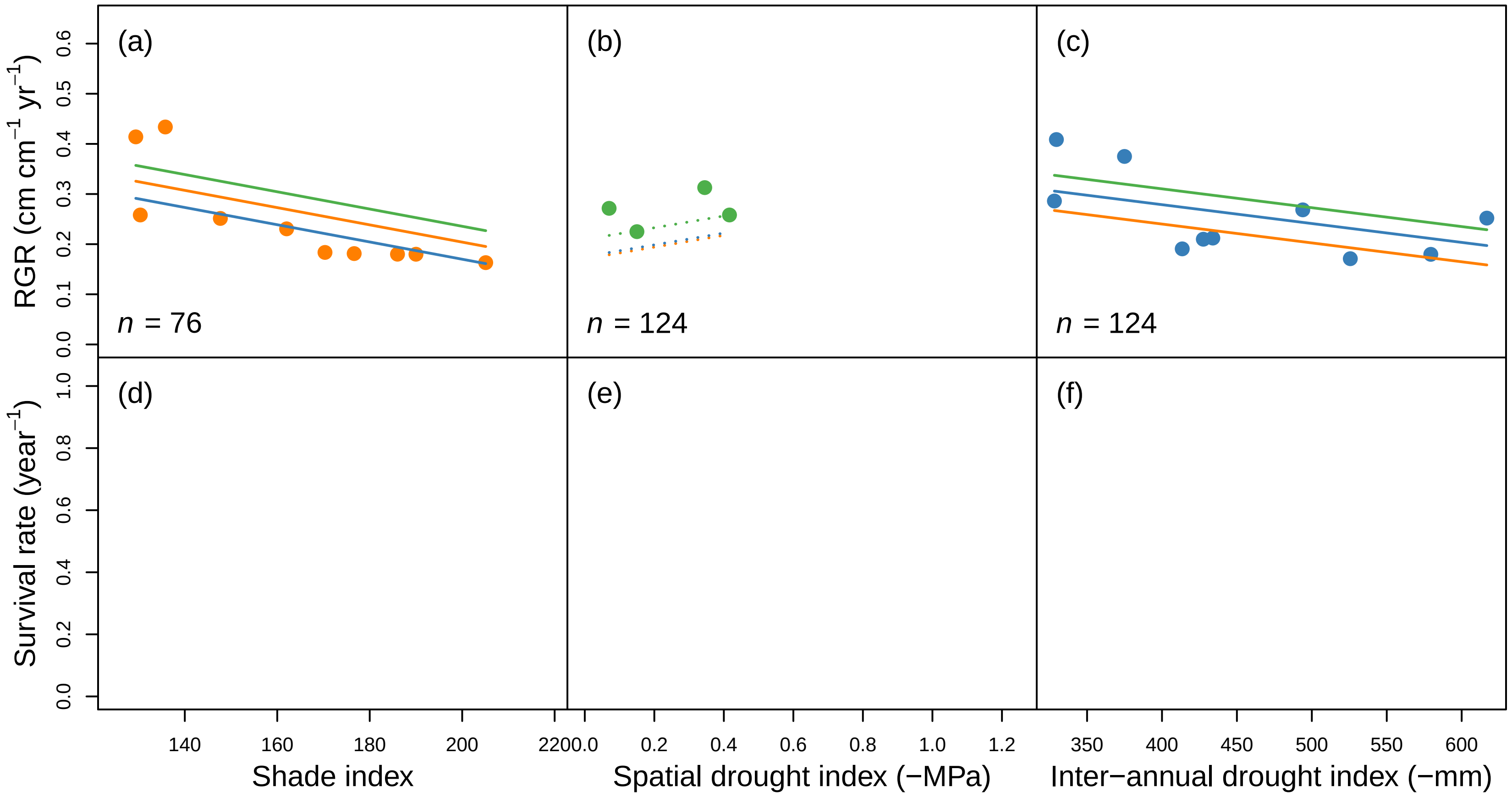

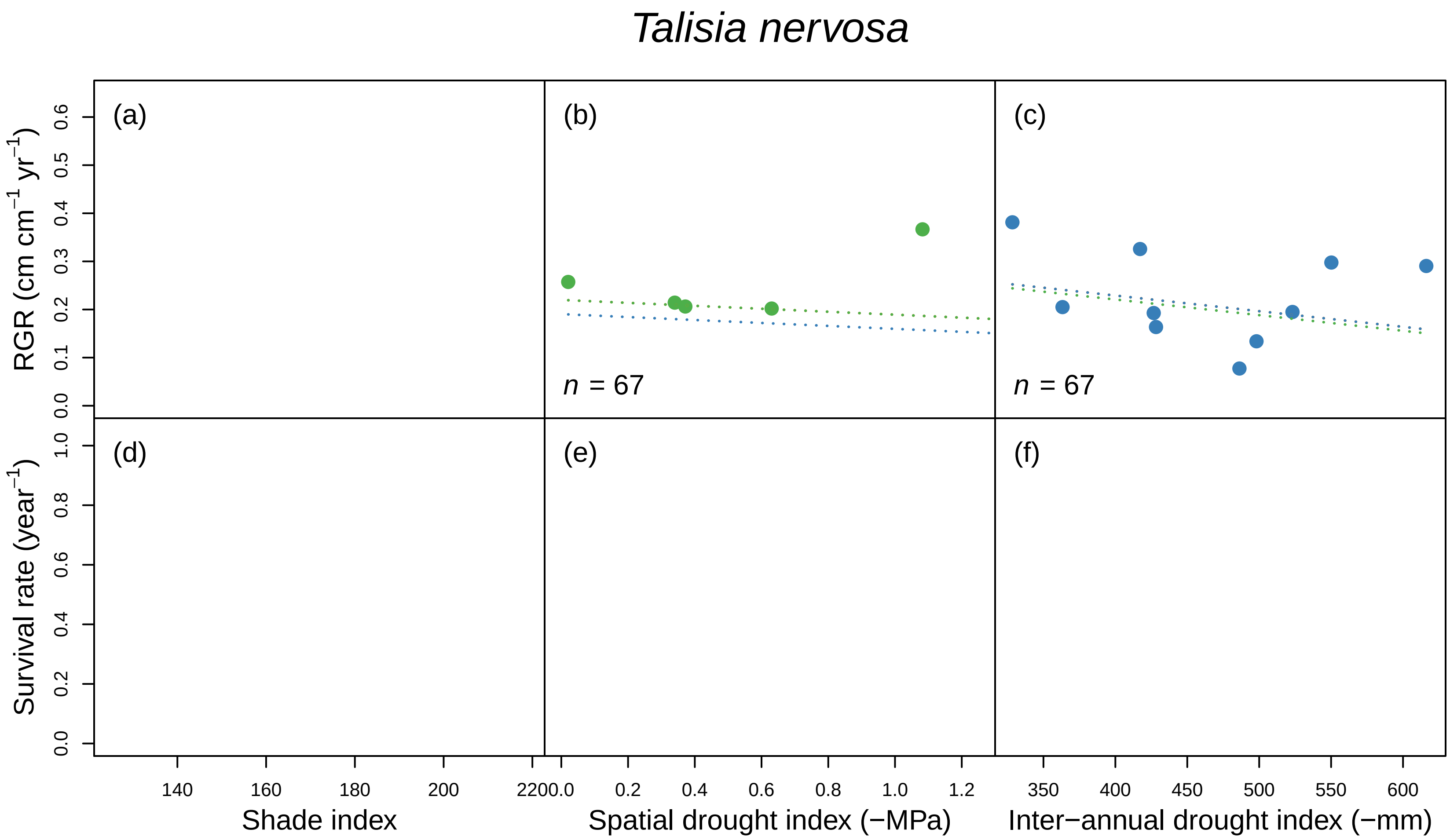

Talisia nervosa
(a)
(b)
(c)
(d)
(e)
(f)
n = 67
n = 67
RGR (cm cm−1 yr−1)
Survival rate (year−1)
0.0
0.1
0.2
0.3
0.4
0.5
0.6
0.0
0.2
0.4
0.6
0.8
1.0
140
160
180
200
220
0.0
0.2
0.4
0.6
0.8
1.0
1.2
350
400
450
500
550
600
Shade index
Spatial drought index (−MPa)
Inter−annual drought index (−mm)

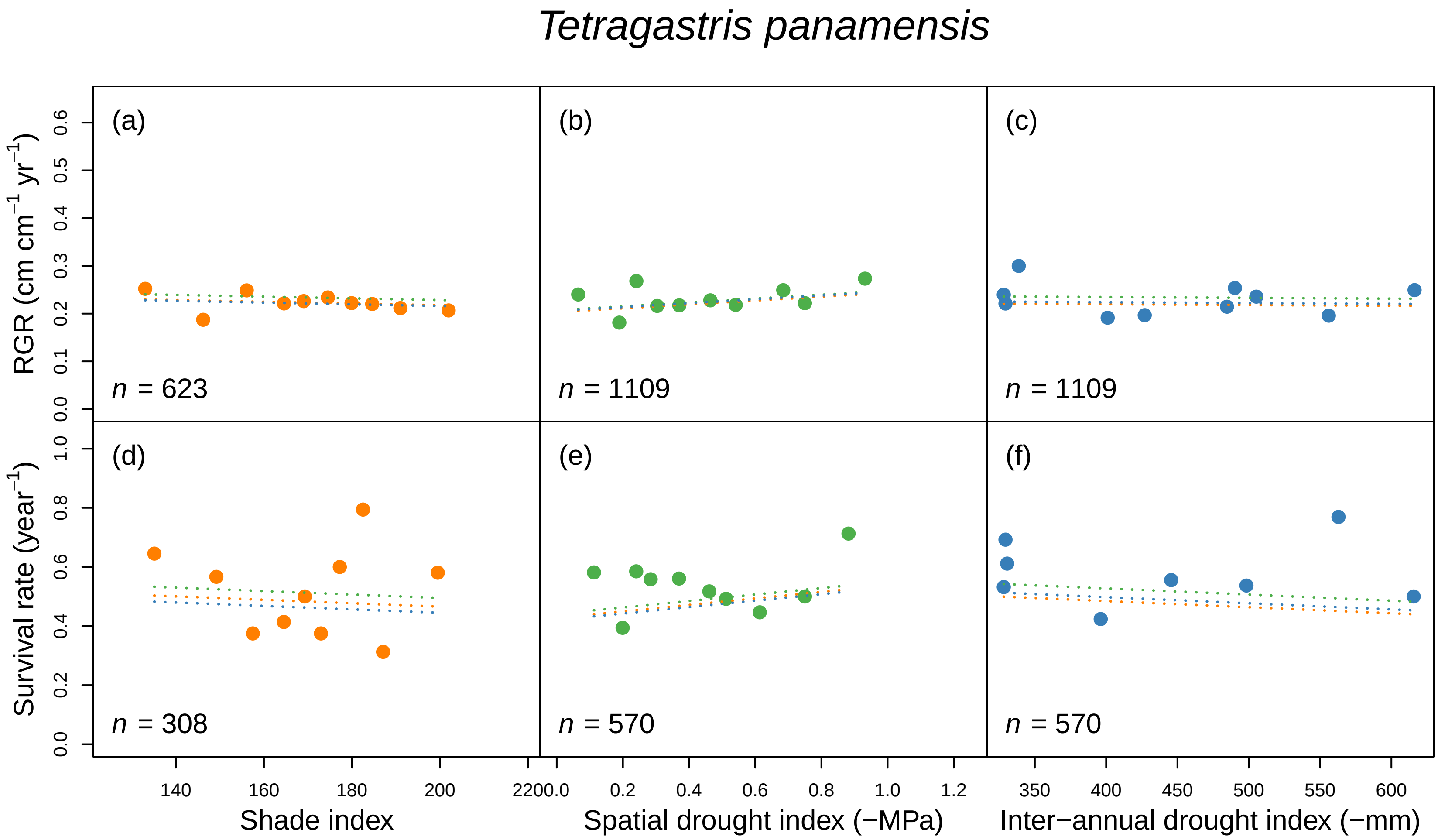

Tetragastris panamensis
(a)
(b)
(c)
(d)
(e)
(f)
n = 623
n = 1109
n = 1109
n = 308
n = 570
n = 570
RGR (cm cm−1 yr−1)
Survival rate (year−1)
Shade index
Spatial drought index (−MPa)
Inter−annual drought index (−mm)

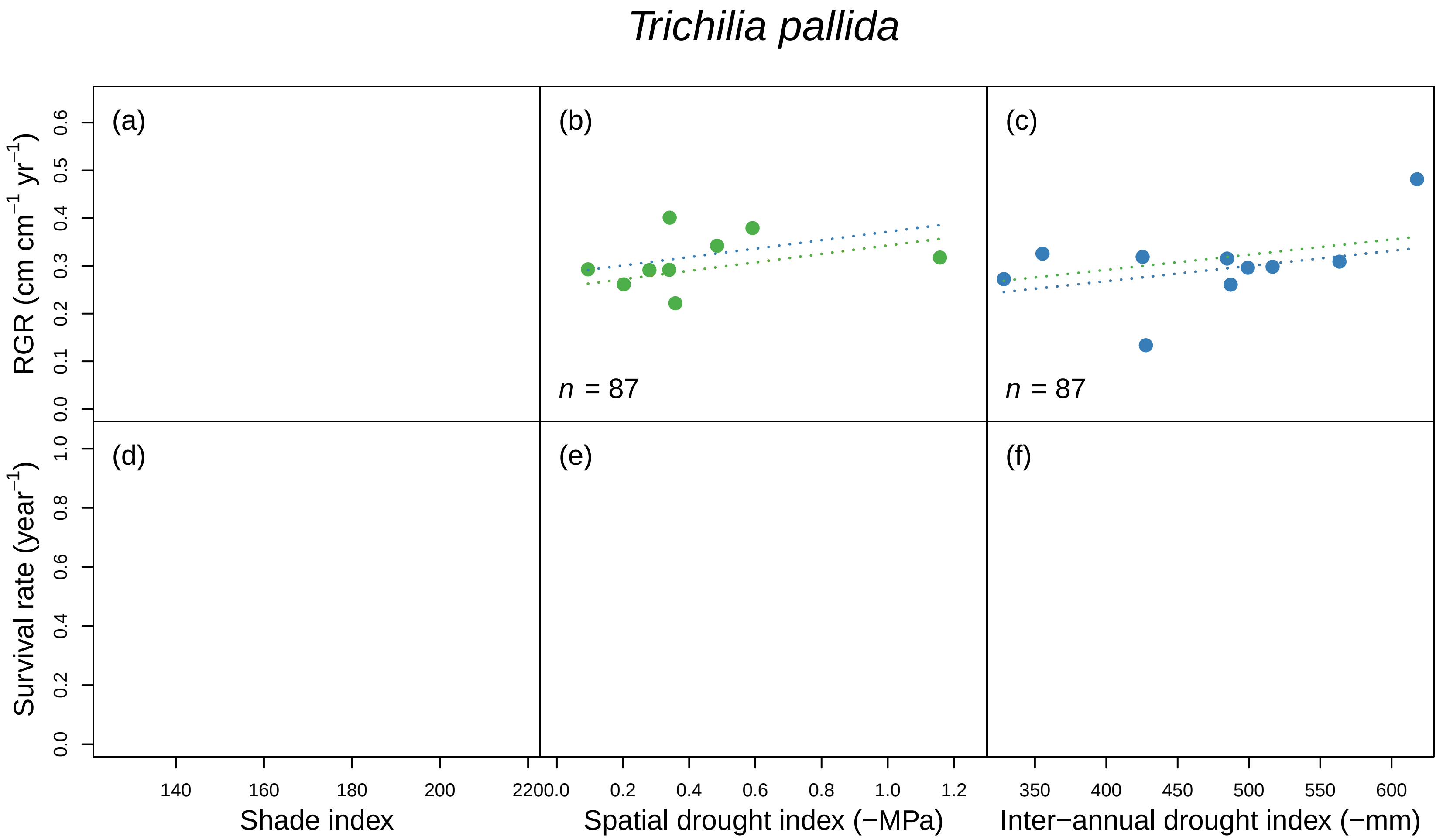
Trichilia pallida
(a)
(b)
(c)
(d)
(e)
(f)
n = 87
n = 87
RGR (cm cm−1 yr−1)
Survival rate (year−1)
0.0
0.1
0.2
0.3
0.4
0.5
0.6
0.0
0.2
0.4
0.6
0.8
1.0
140
160
180
200
220
0.0
0.2
0.4
0.6
0.8
1.0
1.2
350
400
450
500
550
600
Shade index
Spatial drought index (−MPa)
Inter−annual drought index (−mm)

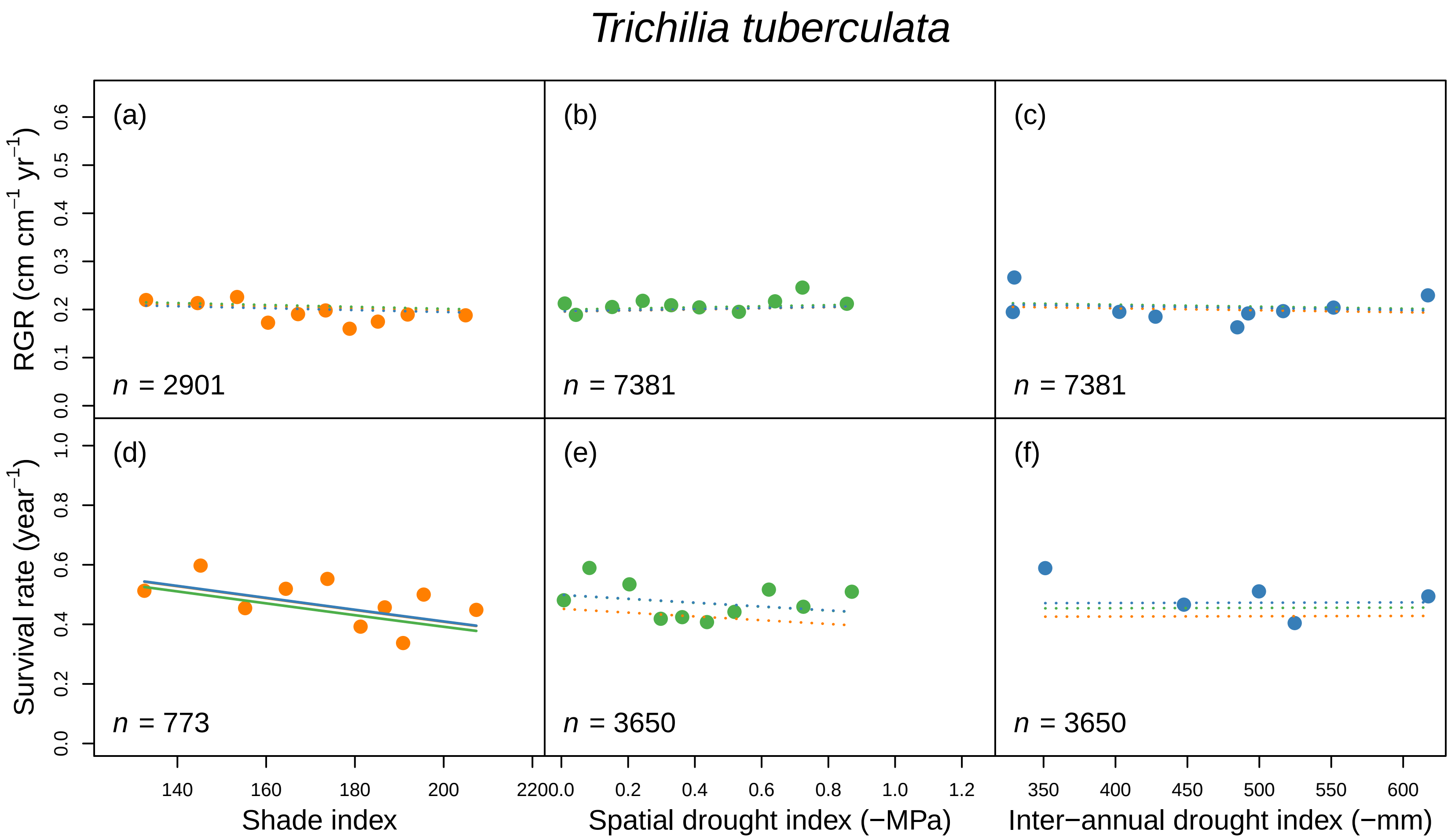

Trichilia tuberculata
(a)
(b)
(c)
(d)
(e)
(f)
n = 2901
n = 7381
n = 7381
n = 773
n = 3650
n = 3650
RGR (cm cm−1 yr−1)
Survival rate (year−1)
0.0
0.1
0.2
0.3
0.4
0.5
0.6
0.0
0.2
0.4
0.6
0.8
1.0
140
160
180
200
220
0.0
0.2
0.4
0.6
0.8
1.0
1.2
350
400
450
500
550
600
Shade index
Spatial drought index (−MPa)
Inter−annual drought index (−mm)

# *Triplaris cumingiana*

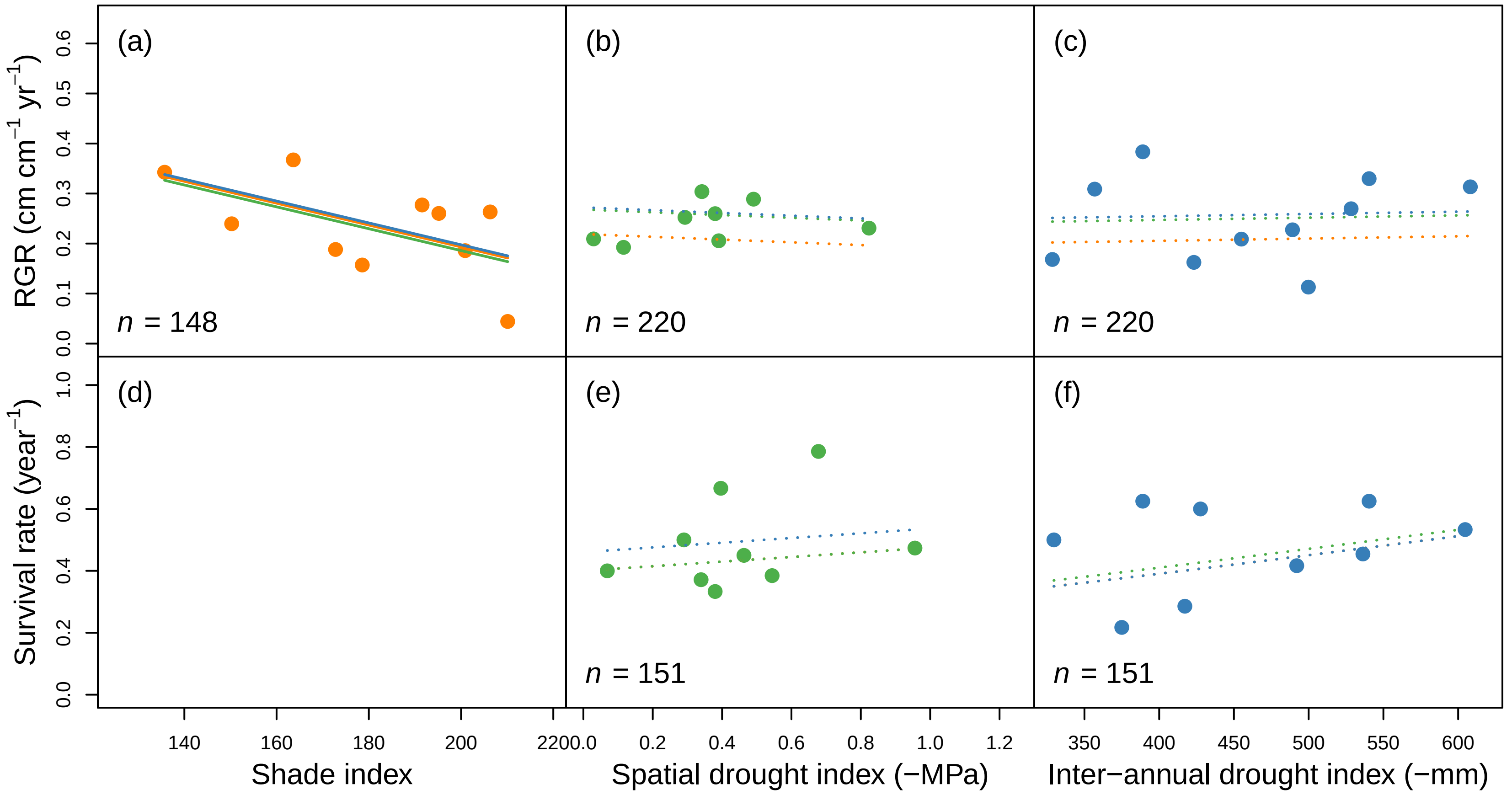

# *Trophis caucana*

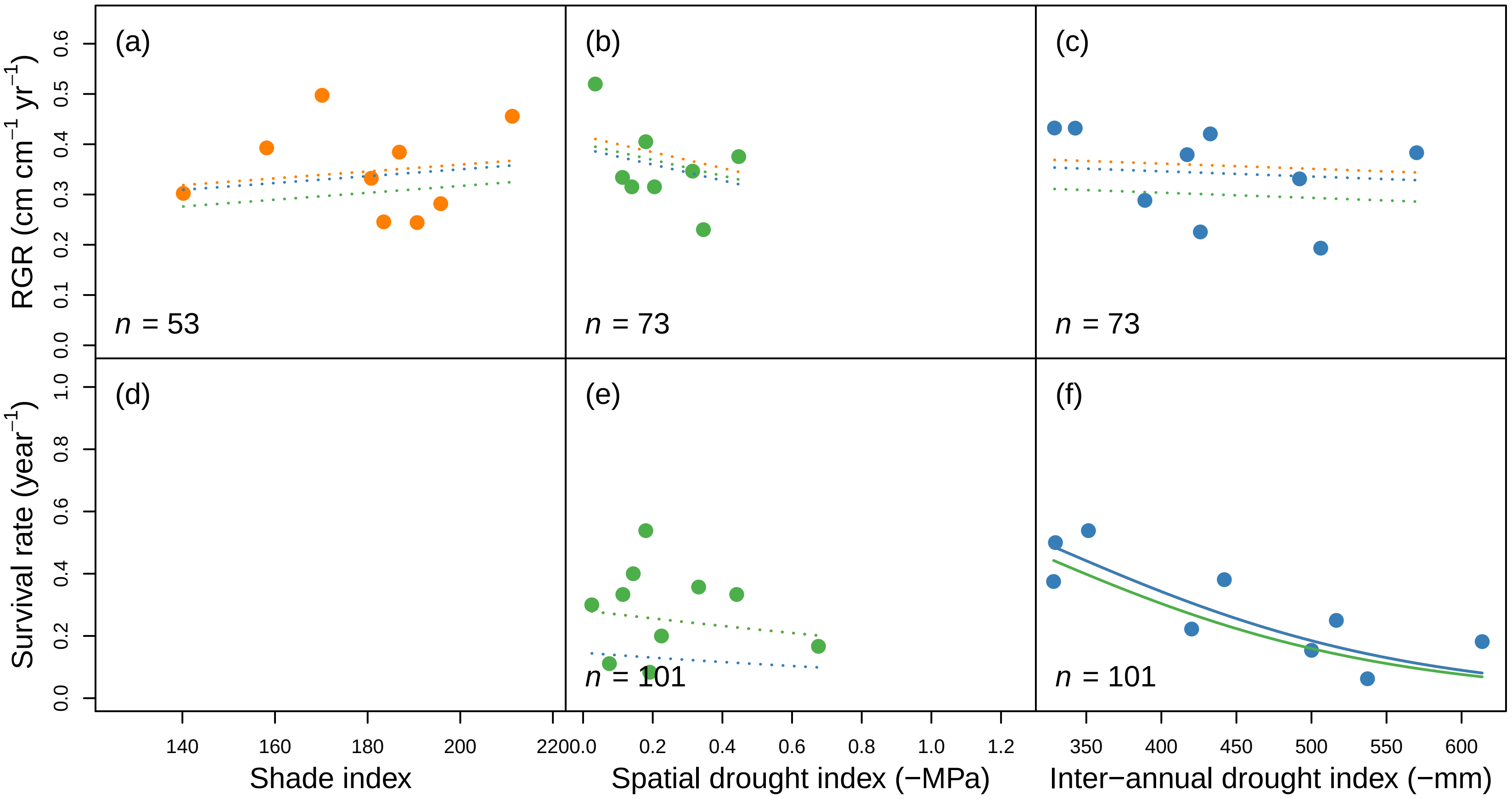

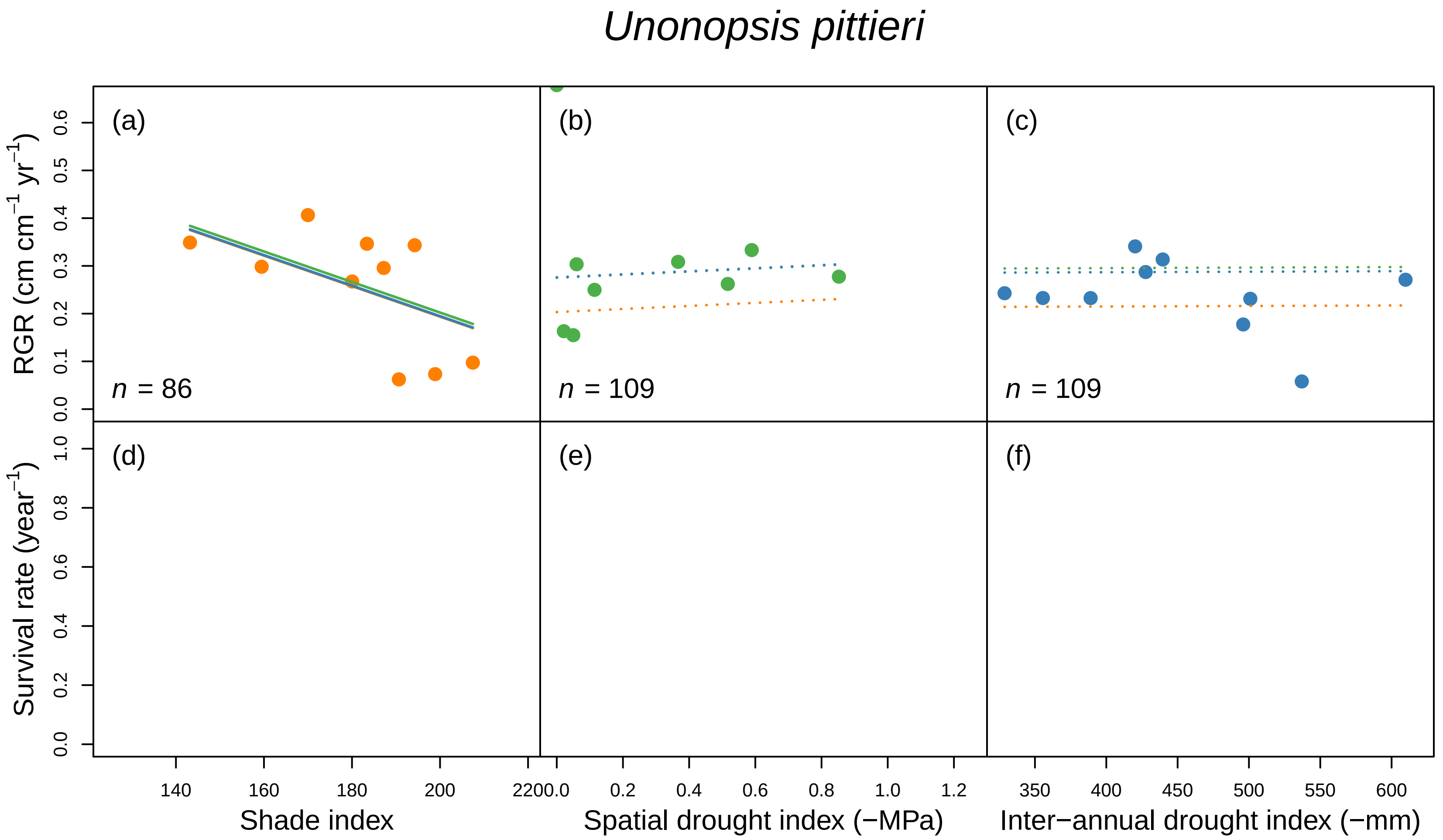
Unonopsis pittieri
(a)
(b)
(c)
(d)
(e)
(f)
n = 86
n = 109
n = 109
RGR (cm cm−1 yr−1)
Survival rate (year−1)
0.0
0.1
0.2
0.3
0.4
0.5
0.6
0.0
0.2
0.4
0.6
0.8
1.0
140
160
180
200
220
0.0
0.2
0.4
0.6
0.8
1.0
1.2
350
400
450
500
550
600
Shade index
Spatial drought index (−MPa)
Inter−annual drought index (−mm)

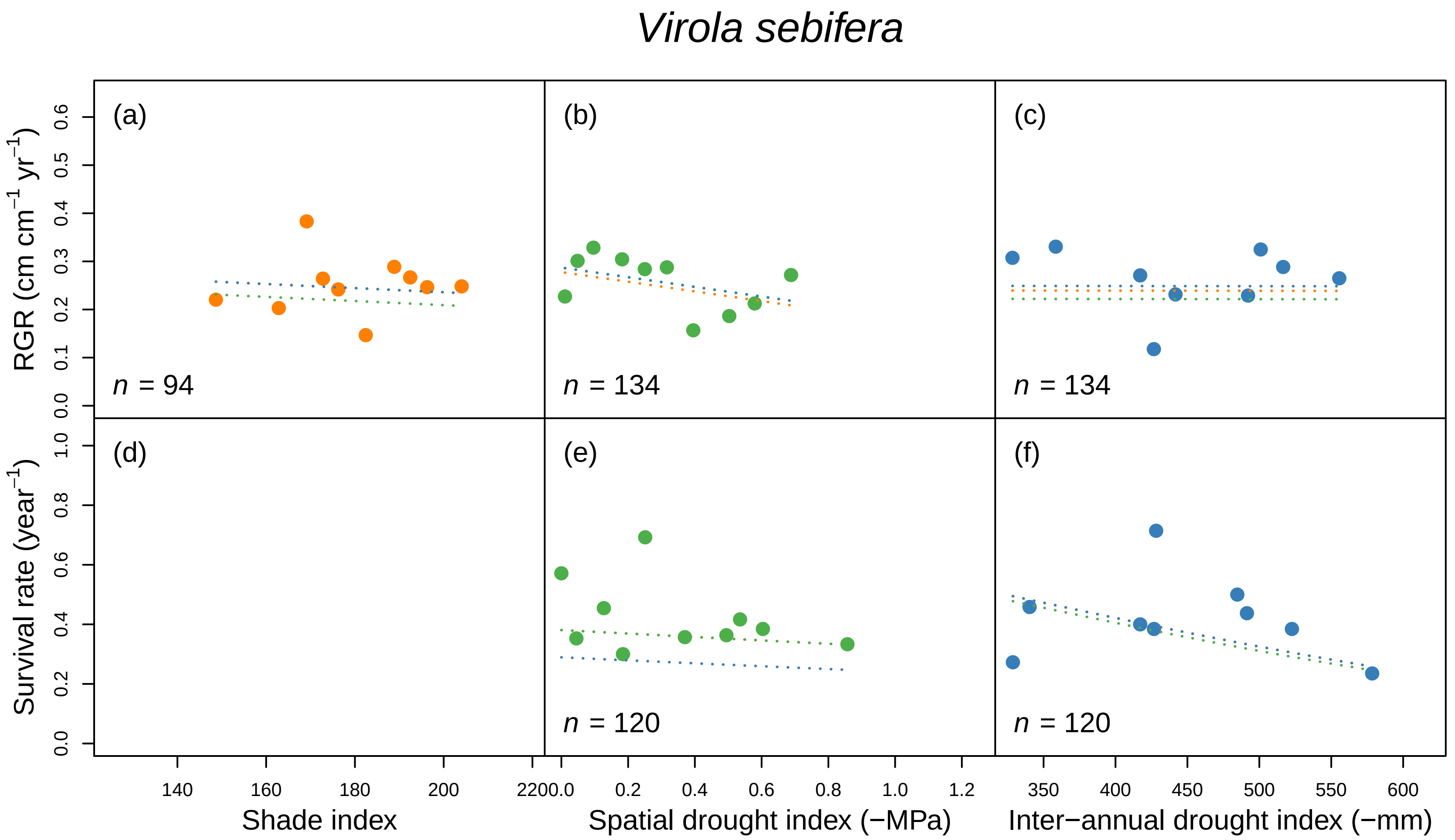

Virola sebifera
RGR (cm cm$^{-1}$ yr$^{-1}$)
Survival rate (year$^{-1}$)
(a)
(b)
(c)
(d)
(e)
(f)
n = 94
n = 134
n = 134
n = 120
n = 120
Shade index
Spatial drought index (−MPa)
Inter−annual drought index (−mm)

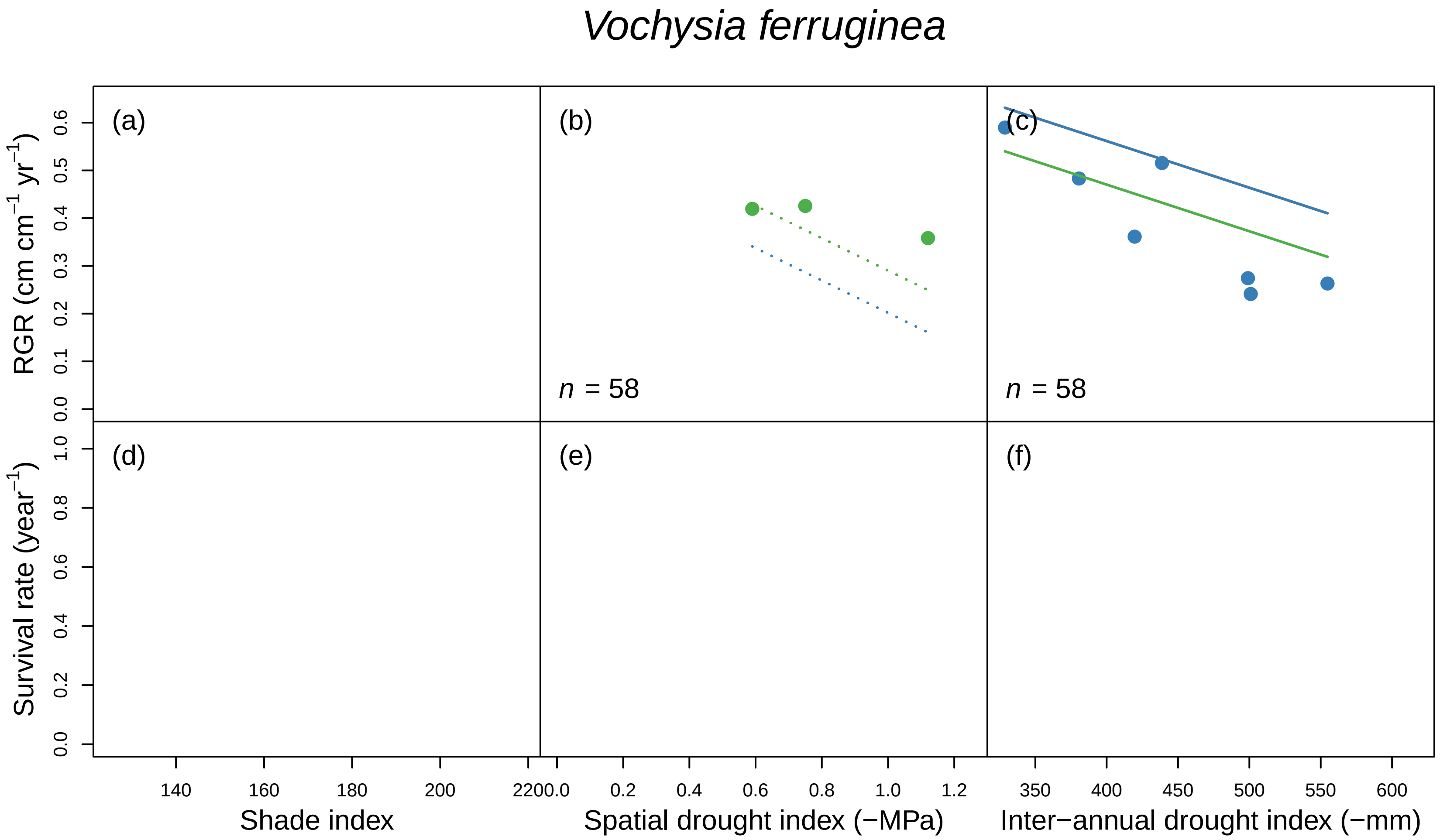

Vochysia ferruginea
RGR (cm cm−1 yr−1)
Survival rate (year−1)
(a)
(b)
(c)
(d)
(e)
(f)
n = 58
n = 58
0.0
0.1
0.2
0.3
0.4
0.5
0.6
0.0
0.2
0.4
0.6
0.8
1.0
140
160
180
200
220
0.0
0.2
0.4
0.6
0.8
1.0
1.2
350
400
450
500
550
600
Shade index
Spatial drought index (−MPa)
Inter−annual drought index (−mm)

**Table S1 Mean and 95% CI of responses in terms of relative growth rates to shade (β1), spatial drought (β2) and inter-annual drought (β3) for all species. Further shown are the number of observations of a species in years with and without shade estimates, mean RGR (β0) and the response to ln(height) (β4). Bold and underlined values indicate significant responses to shade or drought, i.e. the 95% credible interval (CI) excluded zero. Underlined values indicate that the CI of β0 or β4 excluded zero.**

| | Observations | | Mean RGR (β0) | | | Shade (β1) | | | Spatial drought (β2) | | | Int.-an. drought (β3) | | | ln(height) (β4) | | |
|---|---|---|---|---|---|---|---|---|---|---|---|---|---|---|---|---|---|
| Species | all | shade | CI- | mean | CI+ | CI- | mean | CI+ | CI- | mean | CI+ | CI- | mean | CI+ | CI- | mean | CI+ |
| *Acalypha diversifolia* | 229 | 109 | 0.264 | 0.305 | 0.346 | -0.034 | 0.013 | 0.060 | -0.038 | 0.001 | 0.042 | -0.042 | -0.007 | 0.028 | -0.088 | -0.059 | -0.032 |
| *Aegiphila panamensis* | 130 | 89 | 0.323 | 0.366 | 0.410 | -0.073 | -0.016 | 0.042 | -0.004 | 0.041 | 0.087 | -0.072 | -0.031 | 0.009 | -0.162 | -0.128 | -0.094 |
| *Alibertia edulis* | 57 | 33 | 0.219 | 0.291 | 0.363 | | | | -0.036 | 0.023 | 0.082 | -0.046 | 0.006 | 0.056 | -0.152 | -0.101 | -0.052 |
| *Allophylus psilospermus* | 50 | 37 | 0.351 | 0.424 | 0.499 | | | | -0.115 | -0.036 | 0.041 | -0.038 | 0.025 | 0.088 | -0.074 | -0.022 | 0.031 |
| *Alseis blackiana* | 687 | 392 | 0.245 | 0.273 | 0.304 | -0.024 | -0.004 | 0.016 | -0.038 | -0.006 | 0.027 | -0.015 | 0.002 | 0.018 | -0.100 | -0.083 | -0.065 |
| *Beilschmiedia pendula* | 2669 | 2079 | 0.163 | 0.181 | 0.199 | **-0.024** | **-0.016** | **-0.008** | -0.017 | -0.005 | 0.006 | -0.012 | 0.000 | 0.012 | -0.060 | -0.043 | -0.027 |
| *Brosimum alicastrum* | 202 | 118 | 0.246 | 0.290 | 0.334 | -0.058 | -0.020 | 0.019 | -0.014 | 0.015 | 0.045 | -0.042 | -0.018 | 0.006 | -0.149 | -0.101 | -0.053 |
| *Calophyllum longifolium* | 110 | 98 | 0.246 | 0.311 | 0.376 | -0.082 | -0.024 | 0.033 | -0.081 | -0.012 | 0.056 | -0.020 | 0.022 | 0.064 | -0.246 | -0.161 | -0.073 |
| *Capparis frondosa* | 1995 | 1155 | 0.180 | 0.195 | 0.210 | -0.012 | -0.001 | 0.010 | -0.017 | -0.005 | 0.006 | -0.014 | -0.002 | 0.010 | -0.120 | -0.105 | -0.090 |
| *Cassipourea elliptica* | 232 | 137 | 0.216 | 0.257 | 0.297 | **0.002** | **0.042** | **0.080** | -0.035 | 0.015 | 0.064 | **-0.062** | **-0.035** | **-0.008** | -0.131 | -0.105 | -0.080 |
| *Chrysophyllum argenteum* | 171 | 101 | 0.193 | 0.236 | 0.278 | -0.037 | -0.001 | 0.036 | -0.007 | 0.023 | 0.052 | -0.030 | -0.001 | 0.028 | -0.092 | -0.055 | -0.018 |
| *Chrysophyllum cainito* | 541 | 272 | 0.189 | 0.209 | 0.229 | -0.025 | -0.003 | 0.019 | -0.001 | 0.018 | 0.036 | -0.016 | 0.000 | 0.017 | -0.108 | -0.083 | -0.060 |
| *Cinnamomum triplinerve* | 147 | 81 | 0.224 | 0.261 | 0.299 | -0.060 | -0.010 | 0.040 | -0.039 | 0.000 | 0.039 | -0.019 | 0.009 | 0.039 | -0.058 | -0.003 | 0.051 |
| *Cordia alliodora* | 71 | 59 | 0.295 | 0.355 | 0.416 | -0.098 | -0.037 | 0.026 | -0.048 | 0.007 | 0.063 | -0.086 | -0.027 | 0.033 | -0.166 | -0.119 | -0.073 |
| *Cordia bicolor* | 92 | 63 | 0.343 | 0.389 | 0.436 | -0.085 | -0.018 | 0.051 | -0.053 | -0.005 | 0.044 | -0.060 | -0.007 | 0.044 | -0.135 | -0.080 | -0.024 |
| *Cordia lasiocalyx* | 177 | 108 | 0.308 | 0.345 | 0.385 | -0.037 | -0.006 | 0.025 | -0.043 | -0.015 | 0.013 | -0.012 | 0.013 | 0.037 | -0.125 | -0.093 | -0.061 |
| *Coussarea curvigemmia* | 210 | 129 | 0.192 | 0.225 | 0.257 | -0.070 | -0.034 | 0.003 | -0.044 | -0.014 | 0.017 | -0.015 | 0.013 | 0.042 | -0.086 | -0.054 | -0.023 |
| *Croton billbergianus* | 66 | 32 | 0.477 | 0.541 | 0.604 | | | | -0.067 | 0.001 | 0.071 | -0.063 | -0.018 | 0.027 | -0.112 | -0.073 | -0.035 |
| *Cupania rufescens* | 56 | 29 | 0.125 | 0.200 | 0.277 | | | | -0.079 | -0.019 | 0.043 | -0.047 | -0.002 | 0.043 | -0.190 | -0.082 | 0.027 |
| *Desmopsis panamensis* | 893 | 577 | 0.245 | 0.264 | 0.283 | -0.015 | 0.003 | 0.021 | -0.012 | 0.004 | 0.019 | -0.025 | -0.008 | 0.008 | -0.073 | -0.059 | -0.046 |
| *Drypetes standleyi* | 278 | 134 | 0.284 | 0.319 | 0.355 | -0.025 | 0.010 | 0.046 | -0.037 | -0.011 | 0.014 | -0.030 | -0.006 | 0.017 | -0.101 | -0.074 | -0.047 |
| *Erythroxylum macrophyllum* | 146 | 78 | 0.160 | 0.205 | 0.251 | -0.062 | -0.008 | 0.046 | -0.021 | 0.036 | 0.093 | -0.001 | 0.033 | 0.068 | -0.101 | -0.051 | -0.001 |
| *Eugenia coloradoensis* | 112 | 56 | 0.260 | 0.312 | 0.367 | -0.072 | -0.017 | 0.038 | -0.037 | -0.004 | 0.029 | -0.022 | 0.010 | 0.042 | -0.153 | -0.109 | -0.066 |
| *Eugenia galalonensis* | 306 | 217 | 0.249 | 0.279 | 0.309 | -0.048 | -0.015 | 0.017 | -0.034 | -0.004 | 0.026 | -0.022 | 0.002 | 0.027 | -0.078 | -0.056 | -0.035 |
| *Eugenia nesiotica* | 134 | 94 | 0.290 | 0.359 | 0.428 | -0.066 | -0.019 | 0.030 | -0.065 | 0.000 | 0.065 | -0.012 | 0.023 | 0.057 | -0.175 | -0.131 | -0.087 |

| | | | | | | | | | | | | | | | | | |
|---|---|---|---|---|---|---|---|---|---|---|---|---|---|---|---|---|---|
| *Eugenia oerstediana* | 2265 | 1345 | 0.279 | 0.294 | 0.308 | -0.020 | -0.007 | 0.005 | -0.017 | -0.006 | 0.006 | -0.017 | -0.005 | 0.007 | -0.111 | -0.100 | -0.089 |
| *Faramea occidentalis* | 10980 | 5442 | 0.185 | 0.196 | 0.207 | **-0.021** | **-0.015** | **-0.009** | -0.004 | 0.001 | 0.007 | -0.016 | -0.006 | 0.004 | -0.075 | -0.069 | -0.064 |
| *Garcinia intermedia* | 682 | 460 | 0.147 | 0.168 | 0.189 | -0.024 | -0.005 | 0.015 | -0.015 | 0.002 | 0.019 | -0.012 | 0.006 | 0.024 | -0.028 | -0.013 | 0.001 |
| *Guapira standleyana* | 149 | 79 | 0.211 | 0.261 | 0.309 | -0.048 | -0.001 | 0.047 | **0.018** | **0.057** | **0.097** | -0.031 | 0.003 | 0.036 | -0.184 | -0.131 | -0.079 |
| *Guarea bullata* | 87 | 49 | 0.123 | 0.185 | 0.247 | | | | -0.077 | -0.011 | 0.052 | **-0.075** | **-0.039** | **-0.002** | -0.013 | 0.032 | 0.076 |
| *Guarea guidonia* | 462 | 255 | 0.183 | 0.207 | 0.231 | **-0.054** | **-0.032** | **-0.011** | -0.028 | -0.006 | 0.017 | -0.035 | -0.017 | 0.002 | -0.084 | -0.061 | -0.038 |
| *Gustavia superba* | 650 | 374 | 0.139 | 0.158 | 0.178 | -0.029 | -0.007 | 0.015 | -0.039 | -0.018 | 0.002 | -0.024 | -0.007 | 0.011 | -0.070 | -0.048 | -0.026 |
| *Hampea appendiculata* | 51 | 27 | 0.330 | 0.399 | 0.468 | | | | -0.090 | -0.001 | 0.087 | -0.104 | -0.039 | 0.027 | -0.140 | -0.086 | -0.033 |
| *Heisteria acuminata* | 139 | 74 | 0.181 | 0.228 | 0.273 | -0.089 | -0.044 | 0.001 | -0.051 | -0.015 | 0.021 | -0.022 | 0.009 | 0.040 | -0.147 | -0.101 | -0.056 |
| *Heisteria concinna* | 897 | 643 | 0.191 | 0.212 | 0.233 | -0.023 | -0.004 | 0.016 | -0.020 | -0.005 | 0.009 | -0.011 | 0.006 | 0.022 | -0.079 | -0.055 | -0.032 |
| *Hirtella triandra* | 329 | 224 | 0.321 | 0.348 | 0.376 | -0.006 | 0.021 | 0.048 | -0.017 | 0.004 | 0.026 | -0.024 | -0.001 | 0.022 | -0.135 | -0.110 | -0.085 |
| *Hybanthus prunifolius* | 7384 | 4775 | 0.212 | 0.224 | 0.236 | **-0.039** | **-0.031** | **-0.024** | -0.012 | -0.004 | 0.004 | **0.002** | **0.012** | **0.023** | -0.089 | -0.083 | -0.077 |
| *Inga marginata* | 1757 | 1049 | 0.246 | 0.262 | 0.278 | **-0.030** | **-0.015** | **-0.001** | -0.023 | -0.009 | 0.006 | -0.008 | 0.005 | 0.019 | -0.076 | -0.063 | -0.051 |
| *Inga multijuga* | 61 | 33 | 0.198 | 0.280 | 0.361 | | | | -0.045 | 0.108 | 0.267 | -0.056 | -0.008 | 0.040 | -0.105 | -0.061 | -0.018 |
| *Inga nobilis* | 63 | 34 | 0.128 | 0.205 | 0.281 | | | | -0.055 | -0.006 | 0.043 | -0.030 | 0.009 | 0.046 | -0.088 | -0.030 | 0.028 |
| *Inga umbellifera* | 95 | 46 | 0.183 | 0.239 | 0.294 | | | | -0.040 | 0.002 | 0.044 | -0.039 | -0.005 | 0.030 | -0.113 | -0.048 | 0.012 |
| *Lacistema aggregatum* | 84 | 49 | 0.284 | 0.372 | 0.462 | | | | -0.119 | 0.001 | 0.123 | -0.080 | -0.031 | 0.019 | -0.118 | -0.082 | -0.046 |
| *Lonchocarpus heptaphyllus* | 95 | 47 | 0.231 | 0.274 | 0.318 | | | | -0.026 | 0.014 | 0.055 | -0.015 | 0.021 | 0.057 | -0.101 | -0.058 | -0.017 |
| *Malpighia romeroana* | 65 | 35 | 0.170 | 0.243 | 0.316 | | | | -0.254 | -0.080 | 0.095 | -0.043 | 0.002 | 0.047 | -0.176 | -0.109 | -0.043 |
| *Mouriri myrtilloides* | 1792 | 1035 | 0.297 | 0.314 | 0.331 | -0.019 | -0.005 | 0.009 | -0.009 | 0.003 | 0.014 | **-0.031** | **-0.018** | **-0.004** | -0.093 | -0.082 | -0.071 |
| *Nectandra lineata* | 264 | 193 | 0.245 | 0.280 | 0.314 | -0.042 | -0.011 | 0.021 | -0.036 | -0.005 | 0.027 | -0.037 | -0.011 | 0.016 | -0.058 | -0.025 | 0.008 |
| *Ocotea puberula* | 160 | 97 | 0.201 | 0.239 | 0.279 | -0.058 | -0.008 | 0.042 | -0.025 | 0.001 | 0.027 | -0.011 | 0.023 | 0.056 | -0.090 | -0.052 | -0.015 |
| *Ocotea whitei* | 171 | 105 | 0.156 | 0.268 | 0.380 | -0.032 | -0.006 | 0.021 | -0.090 | -0.034 | 0.022 | -0.037 | -0.011 | 0.015 | -0.146 | -0.084 | -0.023 |
| *Oenocarpus mapora* | 92 | 68 | -0.158 | -0.007 | 0.142 | -0.033 | 0.038 | 0.109 | -0.077 | -0.026 | 0.025 | -0.031 | 0.023 | 0.076 | -0.149 | -0.082 | -0.015 |
| *Ouratea lucens* | 222 | 126 | 0.138 | 0.177 | 0.216 | -0.038 | -0.004 | 0.030 | -0.023 | 0.009 | 0.040 | -0.048 | -0.024 | 0.000 | -0.104 | -0.071 | -0.039 |
| *Picramnia latifolia* | 271 | 143 | 0.251 | 0.281 | 0.312 | -0.006 | 0.022 | 0.050 | -0.052 | -0.024 | 0.002 | -0.015 | 0.007 | 0.029 | -0.097 | -0.072 | -0.048 |
| *Piper darienense* | 218 | 110 | 0.175 | 0.224 | 0.272 | -0.035 | 0.009 | 0.053 | -0.019 | 0.018 | 0.056 | -0.051 | -0.017 | 0.016 | -0.137 | -0.100 | -0.063 |
| *Poulsenia armata* | 79 | 52 | 0.221 | 0.309 | 0.398 | -0.080 | -0.008 | 0.065 | -0.095 | -0.008 | 0.080 | -0.066 | -0.019 | 0.028 | -0.166 | -0.112 | -0.059 |
| *Pouteria reticulata* | 715 | 416 | 0.206 | 0.225 | 0.245 | -0.024 | -0.006 | 0.012 | -0.008 | 0.008 | 0.024 | -0.015 | 0.000 | 0.014 | -0.069 | -0.045 | -0.022 |
| *Protium tenuifolium* | 357 | 218 | 0.281 | 0.309 | 0.338 | -0.039 | -0.015 | 0.009 | -0.038 | -0.011 | 0.015 | -0.011 | 0.008 | 0.026 | -0.088 | -0.068 | -0.049 |
| *Psychotria acuminata* | 50 | 30 | 0.461 | 0.547 | 0.631 | | | | -0.050 | 0.031 | 0.109 | -0.055 | 0.001 | 0.056 | -0.188 | -0.143 | -0.098 |
| *Psychotria deflexa* | 71 | 48 | 0.357 | 0.426 | 0.496 | | | | -0.006 | 0.069 | 0.145 | -0.065 | -0.015 | 0.034 | -0.130 | -0.091 | -0.050 |

| | | | | | | | | | | | | | | | | | | | |
|---|---|---|---|---|---|---|---|---|---|---|---|---|---|---|---|---|---|---|---|
| *Psychotria hoffmannseggiana* | 421 | 250 | 0.445 | 0.469 | 0.494 | -0.049 | -0.019 | 0.012 | -0.025 | -0.002 | 0.021 | -0.022 | 0.000 | 0.023 | -0.108 | -0.093 | -0.079 |
| *Psychotria horizontalis* | 1709 | 930 | 0.193 | 0.213 | 0.233 | **-0.042** | **-0.027** | **-0.011** | -0.028 | -0.014 | 0.001 | -0.013 | 0.001 | 0.015 | -0.132 | -0.120 | -0.109 |
| *Psychotria limonensis* | 55 | 20 | 0.297 | 0.371 | 0.445 | | | | -0.048 | 0.009 | 0.065 | -0.036 | 0.007 | 0.051 | -0.102 | -0.055 | -0.008 |
| *Psychotria marginata* | 227 | 127 | 0.282 | 0.321 | 0.361 | -0.052 | -0.005 | 0.040 | -0.036 | 0.007 | 0.049 | -0.035 | -0.007 | 0.020 | -0.103 | -0.081 | -0.059 |
| *Psychotria racemosa* | 128 | 111 | 0.347 | 0.401 | 0.456 | -0.097 | -0.023 | 0.051 | **0.004** | **0.057** | **0.109** | -0.107 | -0.043 | 0.019 | -0.130 | -0.095 | -0.061 |
| *Quararibea asterolepis* | 2652 | 1430 | 0.221 | 0.235 | 0.249 | -0.020 | -0.009 | 0.001 | -0.020 | -0.009 | 0.002 | -0.008 | 0.004 | 0.015 | -0.115 | -0.103 | -0.091 |
| *Randia armata* | 5024 | 2926 | 0.132 | 0.145 | 0.159 | -0.008 | 0.000 | 0.008 | -0.009 | -0.001 | 0.008 | -0.007 | 0.003 | 0.014 | -0.160 | -0.149 | -0.138 |
| *Rinorea sylvatica* | 423 | 226 | 0.208 | 0.236 | 0.264 | -0.027 | 0.000 | 0.028 | -0.036 | -0.008 | 0.021 | -0.027 | -0.006 | 0.014 | -0.106 | -0.085 | -0.064 |
| *Senna dariensis* | 68 | 41 | 0.327 | 0.385 | 0.443 | | | | -0.090 | -0.016 | 0.059 | -0.049 | -0.003 | 0.045 | -0.151 | -0.104 | -0.056 |
| *Simarouba amara* | 173 | 70 | 0.344 | 0.384 | 0.424 | -0.005 | 0.034 | 0.072 | -0.050 | -0.022 | 0.006 | **-0.048** | **-0.024** | **0.000** | -0.105 | -0.073 | -0.042 |
| *Sorocea affinis* | 1113 | 679 | 0.231 | 0.250 | 0.268 | -0.023 | -0.006 | 0.011 | -0.011 | 0.003 | 0.018 | -0.014 | 0.001 | 0.016 | -0.118 | -0.101 | -0.083 |
| *Strychnos brachistantha* | 51 | 37 | 0.032 | 0.186 | 0.342 | | | | -0.078 | -0.004 | 0.069 | -0.088 | -0.020 | 0.048 | -0.358 | -0.084 | 0.187 |
| *Stylogyne turbacensis* | 364 | 170 | 0.113 | 0.151 | 0.189 | -0.053 | -0.022 | 0.008 | -0.012 | 0.014 | 0.041 | -0.026 | -0.002 | 0.022 | -0.127 | -0.098 | -0.070 |
| *Swartzia simplex var.grandiflora* | 350 | 229 | 0.199 | 0.235 | 0.273 | -0.018 | 0.014 | 0.046 | -0.030 | -0.002 | 0.025 | -0.030 | -0.008 | 0.014 | -0.148 | -0.109 | -0.072 |
| *Swartzia simplex var.ochnacea* | 87 | 45 | 0.168 | 0.249 | 0.333 | | | | -0.105 | -0.037 | 0.031 | -0.015 | 0.017 | 0.050 | -0.194 | -0.129 | -0.066 |
| *Tabebuia rosea* | 125 | 81 | 0.311 | 0.362 | 0.413 | -0.090 | -0.035 | 0.019 | -0.059 | -0.018 | 0.021 | -0.040 | 0.007 | 0.054 | -0.106 | -0.065 | -0.026 |
| *Tabernaemontana arborea* | 123 | 63 | 0.248 | 0.305 | 0.361 | -0.004 | 0.046 | 0.095 | -0.066 | -0.009 | 0.047 | -0.049 | -0.016 | 0.016 | -0.111 | -0.075 | -0.041 |
| *Tachigali versicolor* | 124 | 76 | 0.269 | 0.335 | 0.399 | **-0.076** | **-0.039** | **-0.001** | -0.058 | 0.032 | 0.122 | **-0.060** | **-0.034** | **-0.007** | -0.102 | -0.066 | -0.030 |
| *Talisia nervosa* | 67 | 41 | 0.164 | 0.232 | 0.300 | | | | -0.040 | -0.008 | 0.024 | -0.076 | -0.029 | 0.016 | -0.164 | -0.090 | -0.014 |
| *Tetragastris panamensis* | 1109 | 623 | 0.238 | 0.254 | 0.271 | -0.018 | -0.004 | 0.010 | 0.000 | 0.011 | 0.022 | -0.014 | -0.002 | 0.011 | -0.059 | -0.045 | -0.032 |
| *Trichilia pallida* | 87 | 40 | 0.216 | 0.275 | 0.331 | | | | -0.024 | 0.024 | 0.072 | -0.015 | 0.029 | 0.072 | -0.148 | -0.086 | -0.025 |
| *Trichilia tuberculata* | 7381 | 2901 | 0.183 | 0.194 | 0.206 | -0.012 | -0.004 | 0.003 | -0.003 | 0.003 | 0.009 | -0.014 | -0.004 | 0.006 | -0.060 | -0.053 | -0.047 |
| *Triplaris cumingiana* | 220 | 148 | 0.124 | 0.171 | 0.217 | **-0.080** | **-0.049** | **-0.018** | -0.042 | -0.007 | 0.027 | -0.023 | 0.004 | 0.032 | -0.203 | -0.152 | -0.102 |
| *Trophis caucana* | 73 | 53 | 0.186 | 0.274 | 0.362 | -0.047 | 0.015 | 0.078 | -0.144 | -0.043 | 0.058 | -0.066 | -0.009 | 0.048 | -0.205 | -0.136 | -0.067 |
| *Unonopsis pittieri* | 109 | 86 | 0.297 | 0.355 | 0.414 | **-0.133** | **-0.072** | **-0.010** | -0.039 | 0.009 | 0.056 | -0.047 | 0.001 | 0.049 | -0.268 | -0.194 | -0.121 |
| *Virola sebifera* | 134 | 94 | 0.208 | 0.251 | 0.294 | -0.070 | -0.010 | 0.050 | -0.074 | -0.027 | 0.020 | -0.047 | 0.000 | 0.046 | -0.112 | -0.051 | 0.009 |
| *Vochysia ferruginea* | 58 | 47 | 0.378 | 0.508 | 0.640 | | | | -0.187 | -0.091 | 0.004 | **-0.160** | **-0.089** | **-0.017** | -0.170 | -0.109 | -0.047 |

**Table S2 Mean and 95% CI of responses in terms of first-year survival to shade (β1), spatial drought (β2) and inter-annual drought (β3) for all species. Further shown are the number of observations of a species in years with and without shade estimates, the survival constant (β0) and the response to ln(height) (β4). Bold and underlined values indicate significant responses to shade or drought, i.e. the 95% credible interval (CI) excluded zero. Underlined values indicate that the CI of β0 or β4 excluded zero.**

| | Observations | | Survival constant (β0) | | | Shade (β1) | | | Spatial drought (β2) | | | Int.-an. drought (β3) | | | ln(height) (β4) | | |
|---|---|---|---|---|---|---|---|---|---|---|---|---|---|---|---|---|---|
| Species | all | shade | CI- | mean | CI+ | CI- | mean | CI+ | CI- | mean | CI+ | CI- | mean | CI+ | CI- | mean | CI+ |
| *Acalypha diversifolia* | 104 | 56 | 0.445 | 1.105 | 1.844 | | | | **-0.963** | **-0.484** | **-0.031** | **-1.620** | **-1.002** | **-0.429** | 0.389 | 0.798 | 1.266 |
| *Aegiphila panamensis* | 172 | 143 | -1.183 | -0.697 | -0.213 | -0.304 | 0.277 | 0.867 | -0.189 | 0.224 | 0.644 | -0.539 | -0.026 | 0.459 | 0.604 | 1.246 | 1.939 |
| *Apeiba membranacea* | 119 | 75 | -2.852 | -1.917 | -1.091 | | | | -1.068 | -0.336 | 0.331 | -0.719 | 0.051 | 0.798 | 0.229 | 0.861 | 1.577 |
| *Beilschmiedia pendula* | 1782 | 1428 | -2.086 | -1.694 | -1.321 | -0.050 | 0.071 | 0.191 | **-0.279** | **-0.141** | **-0.002** | -0.172 | -0.002 | 0.171 | 0.702 | 0.894 | 1.092 |
| *Capparis frondosa* | 218 | 131 | 0.424 | 0.767 | 1.118 | -0.383 | 0.040 | 0.475 | -0.437 | -0.088 | 0.274 | -0.689 | -0.312 | 0.065 | 0.448 | 0.943 | 1.475 |
| *Cecropia insignis* | 166 | 115 | -2.835 | -2.188 | -1.627 | -0.249 | 0.502 | 1.321 | -0.210 | 0.257 | 0.698 | **-1.952** | **-1.056** | **-0.237** | 0.218 | 0.568 | 0.953 |
| *Chrysophyllum cainito* | 220 | 91 | -0.030 | 0.431 | 0.909 | | | | **0.065** | **0.396** | **0.727** | -0.295 | 0.079 | 0.476 | -0.336 | 0.207 | 0.755 |
| *Cinnamomum triplinerve* | 101 | 44 | -1.570 | -0.809 | -0.103 | | | | -0.388 | 0.081 | 0.525 | -0.332 | 0.115 | 0.578 | -0.223 | 0.453 | 1.218 |
| *Coussarea curvigemmia* | 129 | 87 | -0.741 | -0.321 | 0.100 | | | | -0.422 | -0.056 | 0.301 | -0.779 | -0.125 | 0.523 | -0.534 | 0.322 | 1.218 |
| *Dendropanax arboreus* | 180 | 105 | -2.667 | -1.234 | 0.150 | -0.663 | -0.008 | 0.650 | -0.736 | -0.211 | 0.273 | **-1.241** | **-0.632** | **-0.078** | -0.058 | 0.868 | 1.830 |
| *Desmopsis panamensis* | 333 | 258 | 0.408 | 0.719 | 1.042 | -0.637 | -0.317 | 0.002 | -0.240 | 0.039 | 0.318 | -0.210 | 0.145 | 0.503 | 0.697 | 1.121 | 1.584 |
| *Eugenia oerstediana* | 836 | 398 | 0.046 | 0.230 | 0.415 | -0.320 | -0.066 | 0.183 | -0.165 | 0.008 | 0.183 | -0.243 | -0.059 | 0.121 | 0.434 | 0.699 | 0.973 |
| *Faramea occidentalis* | 6110 | 2348 | -0.431 | -0.302 | -0.176 | -0.104 | 0.003 | 0.111 | **-0.172** | **-0.096** | **-0.019** | **-0.277** | **-0.157** | **-0.037** | 0.753 | 0.842 | 0.933 |
| *Guapira standleyana* | 164 | 82 | -0.959 | -0.601 | -0.248 | | | | -0.222 | 0.120 | 0.454 | -0.543 | -0.164 | 0.203 | -0.391 | 0.033 | 0.455 |
| *Guarea guidonia* | 178 | 108 | -0.441 | -0.031 | 0.375 | -0.674 | -0.201 | 0.282 | **-0.768** | **-0.439** | **-0.120** | -0.524 | -0.182 | 0.164 | 0.031 | 0.706 | 1.405 |
| *Gustavia superba* | 348 | 169 | -0.451 | -0.096 | 0.264 | -0.024 | 0.337 | 0.709 | -0.265 | -0.006 | 0.257 | -0.469 | -0.176 | 0.116 | 0.295 | 0.578 | 0.860 |
| *Hampea appendiculata* | 100 | 65 | -2.460 | -1.616 | -0.882 | | | | -0.913 | -0.166 | 0.590 | -1.362 | -0.531 | 0.282 | 0.450 | 1.604 | 2.840 |
| *Heisteria concinna* | 487 | 323 | -0.319 | -0.042 | 0.228 | -0.057 | 0.192 | 0.442 | **0.002** | **0.193** | **0.385** | -0.426 | -0.195 | 0.038 | 0.234 | 0.637 | 1.042 |
| *Hirtella triandra* | 214 | 137 | -1.742 | -1.203 | -0.700 | -0.215 | 0.193 | 0.602 | -0.313 | 0.012 | 0.331 | -0.513 | -0.170 | 0.172 | 0.864 | 1.360 | 1.898 |
| *Hybanthus prunifolius* | 5661 | 3789 | -0.117 | 0.016 | 0.149 | **-0.256** | **-0.163** | **-0.072** | -0.074 | 0.014 | 0.100 | **-0.244** | **-0.129** | **-0.014** | 0.935 | 1.044 | 1.154 |
| *Inga marginata* | 567 | 305 | 0.117 | 0.371 | 0.629 | -0.261 | 0.001 | 0.264 | -0.351 | -0.151 | 0.051 | -0.226 | 0.002 | 0.230 | 0.426 | 0.772 | 1.138 |
| *Jacaranda copaia* | 159 | 86 | -6.137 | -4.062 | -2.537 | | | | -0.902 | 0.738 | 2.577 | -3.335 | -1.230 | 0.608 | -0.179 | 1.083 | 2.435 |
| *Luehea seemannii* | 165 | 27 | -2.191 | -1.433 | -0.763 | | | | -0.656 | 0.093 | 0.878 | -0.779 | -0.214 | 0.358 | 0.202 | 0.529 | 0.889 |
| *Miconia argentea* | 115 | 87 | -1.226 | -0.662 | -0.109 | | | | -0.704 | -0.173 | 0.303 | -1.203 | -0.546 | 0.071 | 0.320 | 0.638 | 0.993 |
| *Mouriri myrtilloides* | 488 | 285 | -0.784 | -0.506 | -0.235 | -0.151 | 0.121 | 0.395 | -0.218 | -0.029 | 0.162 | -0.238 | -0.013 | 0.211 | 1.218 | 1.659 | 2.119 |
| *Ocotea puberula* | 232 | 147 | -1.110 | -0.762 | -0.422 | -0.310 | 0.132 | 0.584 | -0.058 | 0.220 | 0.492 | -0.483 | -0.174 | 0.129 | -0.016 | 0.729 | 1.502 |

| | | | | | | | | | | | | | | | | | |
|---|---|---|---|---|---|---|---|---|---|---|---|---|---|---|---|---|---|
| *Ocotea whitei* | 169 | 109 | -6.022 | -3.775 | -1.713 | -0.250 | 0.118 | 0.491 | -0.424 | 0.360 | 1.136 | **-1.095** | **-0.616** | **-0.164** | 0.603 | 1.247 | 1.945 |
| *Piper reticulatum* | 151 | 65 | -1.904 | -0.987 | -0.112 | | | | -0.541 | 0.035 | 0.585 | **-1.057** | **-0.573** | **-0.127** | -0.059 | 0.388 | 0.842 |
| *Pouteria reticulata* | 331 | 153 | -1.585 | -0.764 | 0.000 | -0.060 | 0.302 | 0.671 | -0.373 | -0.105 | 0.158 | **-0.689** | **-0.429** | **-0.173** | 0.312 | 0.914 | 1.554 |
| *Protium tenuifolium* | 117 | 46 | -0.913 | -0.041 | 0.785 | | | | -0.534 | -0.080 | 0.358 | -0.842 | -0.397 | 0.025 | -0.270 | 0.534 | 1.444 |
| *Psychotria hoffmannseggiana* | 345 | 210 | -0.231 | 0.093 | 0.434 | -0.503 | -0.182 | 0.132 | -0.304 | -0.054 | 0.189 | -0.556 | -0.260 | 0.031 | 0.562 | 0.862 | 1.172 |
| *Psychotria horizontalis* | 1315 | 788 | 0.173 | 0.447 | 0.730 | **-0.377** | **-0.200** | **-0.025** | -0.264 | -0.124 | 0.016 | -0.193 | -0.031 | 0.130 | 0.599 | 0.759 | 0.926 |
| *Psychotria marginata* | 191 | 126 | -0.493 | 0.286 | 1.072 | -1.059 | -0.511 | 0.037 | -0.523 | -0.095 | 0.325 | -0.351 | 0.056 | 0.461 | 0.349 | 0.772 | 1.215 |
| *Quararibea asterolepis* | 1299 | 931 | -0.745 | -0.559 | -0.379 | -0.268 | -0.105 | 0.052 | -0.055 | 0.096 | 0.246 | -0.148 | 0.018 | 0.186 | 0.696 | 0.883 | 1.076 |
| *Randia armata* | 2467 | 1217 | -0.173 | -0.036 | 0.107 | **0.107** | **0.248** | **0.390** | -0.088 | 0.021 | 0.133 | **-0.335** | **-0.197** | **-0.061** | 0.667 | 0.830 | 0.995 |
| *Rinorea sylvatica* | 183 | 83 | -0.142 | 0.435 | 1.013 | | | | -0.289 | 0.134 | 0.555 | -0.245 | 0.334 | 0.939 | 0.561 | 1.173 | 1.852 |
| *Senna dariensis* | 178 | 136 | -1.576 | -1.029 | -0.485 | **0.034** | **0.662** | **1.342** | -1.032 | -0.372 | 0.263 | -0.789 | -0.255 | 0.277 | 0.640 | 1.416 | 2.282 |
| *Sorocea affinis* | 604 | 330 | -0.462 | -0.252 | -0.042 | -0.033 | 0.213 | 0.460 | -0.123 | 0.058 | 0.240 | -0.268 | -0.057 | 0.156 | 0.375 | 0.677 | 0.997 |
| *Stylogyne turbacensis* | 137 | 55 | -0.061 | 0.668 | 1.443 | | | | -0.325 | -0.009 | 0.308 | -0.593 | -0.234 | 0.121 | -0.135 | 0.434 | 1.029 |
| *Tabebuia rosea* | 133 | 76 | -2.079 | -1.403 | -0.776 | | | | -0.192 | 0.181 | 0.560 | -0.300 | 0.298 | 0.927 | 0.924 | 1.658 | 2.461 |
| *Tetragastris panamensis* | 570 | 308 | -1.965 | -1.392 | -0.836 | -0.359 | -0.053 | 0.247 | -0.074 | 0.118 | 0.315 | -0.270 | -0.083 | 0.100 | 0.705 | 1.143 | 1.612 |
| *Trichilia tuberculata* | 3650 | 773 | -0.298 | -0.153 | -0.008 | **-0.342** | **-0.184** | **-0.023** | -0.159 | -0.070 | 0.019 | -0.130 | 0.004 | 0.137 | 0.877 | 1.014 | 1.155 |
| *Triplaris cumingiana* | 151 | 93 | -0.337 | 0.105 | 0.549 | | | | -0.324 | 0.082 | 0.489 | -0.165 | 0.246 | 0.661 | 0.605 | 1.322 | 2.102 |
| *Trophis caucana* | 101 | 75 | -1.944 | -1.164 | -0.439 | | | | -0.934 | -0.177 | 0.548 | **-1.437** | **-0.832** | **-0.274** | -0.474 | 0.261 | 1.012 |
| *Virola sebifera* | 120 | 67 | -2.239 | -1.423 | -0.688 | | | | -0.535 | -0.068 | 0.389 | -0.934 | -0.413 | 0.084 | 0.526 | 1.323 | 2.219 |
